# Ruthenium(II)-Catalyzed Homocoupling of α-Carbonyl Sulfoxonium Ylides Under Mild Conditions: Methodology Development and Mechanistic DFT Study

**DOI:** 10.3389/fchem.2020.00648

**Published:** 2020-09-16

**Authors:** Maosheng Zhang, Jinrong Zhang, Zhenfang Teng, Jianhui Chen, Yuanzhi Xia

**Affiliations:** ^1^College of Chemistry and Materials Engineering, Wenzhou University, Wenzhou, China; ^2^Information Technology Center, Wenzhou University, Wenzhou, China

**Keywords:** C-H activation, mechanism, DFT calculations, ruthenium—catalyst, sulfoxonium ylide

## Abstract

A mild ruthenium(II)-catalyzed homocoupling of α-carbonyl sulfoxonium ylides was developed and the detailed mechanism was understood based on DFT calculations in the current report. The catalytic system utilizes the α-carbonyl sulfoxonium ylide as both the directing group for *ortho*-sp^2^ C-H activation and the acylmethylating reagent for C-C coupling. Various substituents are compatible in the transformation and a variety of isocoumarin derivatives were synthesized at room temperature without any protection. The theoretical results disclosed that the full catalytic cycle contains eight elementary steps, and in all the cationic Ru(II) monomer is involved as the catalytic active species. The acid additive is responsible for protonation of the ylide carbon prior to the intramolecular nucleophilic addition and C-C bond cleavage. Interestingly, the intermediacy of free acylmethylation intermediate or its enol isomer is not necessary for the transformation.

**Graphical Abstract:**
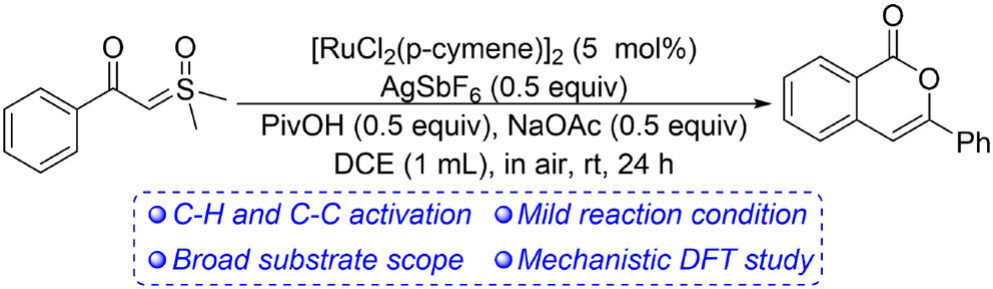
Ru(II)-catalyzed homocoupling of alpha-carbonyl sulfoxonium ylides.

## Introduction

**Scheme 1:**
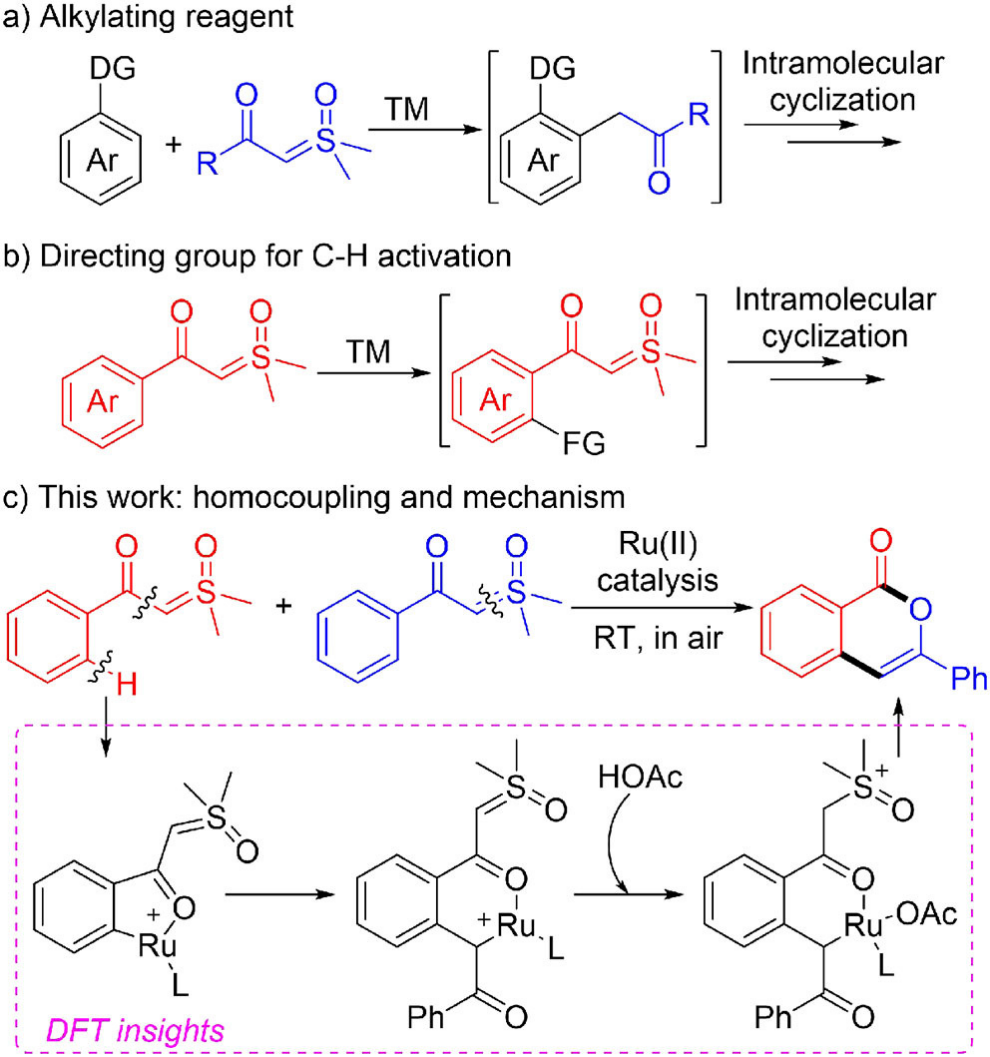
Applications of α-carbonyl sulfoxonium ylides under transition metal catalysis.

Under transition metal catalysis, the sulfoxonium ylides have found wide applications in synthetic chemistry (Li et al., 1997). These species could be used as efficient carbene precursors by elimination of dimethyl sulfoxide (DMSO) by activation of the ylide C-S bond with metal (Bayer and Vaitla, 2018; Cheng et al., 2018). This strategy has recently found important applications in transition metal-catalyzed C-H activation reactions (Scheme 1) (Gulias and Mascarenas, 2016; Wang et al., 2016; Sambiagio et al., 2018), as sulfoxonium ylides possess the advantages of easy availability of starting materials and safe operation in reactions compared with the alternative approach with diazo precursors (Davies and Manning, 2008; Xia et al., 2017; Clare et al., 2019; Wen et al., 2019; Zhou et al., 2020). In this context, since the initial independent reports by the Li (Xu et al., 2017a) and Aïssa (Barday et al., 2017) groups, interesting acylmethylation methods with sulfoxonium ylides as the acylmethylating reagents have been developed under the catalysis of rhodium (You et al., 2018; Xu et al., 2019; Yu J. et al., 2019; Tian et al., 2020), ruthenium (Karishma et al., 2019; Li H. et al., 2019; Fu et al., 2020), and other transition metals (Ji et al., 2018; Li C. et al., 2019). Notably, tandem intramolecular annulations of the *in-situ* generated acylmethylation products were achieved for novel constructions of naphthols (Chen et al., 2018; Cui et al., 2019; Lai et al., 2019; Luo et al., 2019; Lv et al., 2019; Shen et al., 2019; Wu C. et al., 2019; Xie et al., 2019; Zhang et al., 2019; Wu et al., 2020), indoles (Hu et al., 2018a; Xiao et al., 2018; Zhou et al., 2018; Wang and Xu, 2019), and other heterocyclic compounds (Hoang and Ellman, 2018; Hoang et al., 2018; Hu et al., 2018b, 2019; Liang et al., 2018; Shi et al., 2018; Xie et al., 2018a; Xie H. et al., 2018; Xu et al., 2018; Cai et al., 2019; Chen P. et al., 2019; Huang et al., 2019; Liu et al., 2019; Nie et al., 2019; Zhang et al., 2020) of biological and pharmacological importance (Scheme 1A). In these cases, diverse reactivity of the sulfoxonium ylides were observed as they may serve as C1 or C2 synthons depending on the reaction condition and substrate structure (Chen et al., 2018; Hoang and Ellman, 2018; Hoang et al., 2018; Hu et al., 2018a,b, 2019; Liang et al., 2018; Shi et al., 2018; Xiao et al., 2018; Xie et al., 2018b, 2019; Xie H. et al., 2018; Xu et al., 2018; Zhou et al., 2018; Cai et al., 2019; Chen P. et al., 2019; Cui et al., 2019; Huang et al., 2019; Lai et al., 2019; Liu et al., 2019; Luo et al., 2019; Lv et al., 2019; Nie et al., 2019; Shen et al., 2019; Wang and Xu, 2019; Wu C. et al., 2019; Zhang et al., 2019, 2020; Wu et al., 2020).

Except for the application of sulfoxonium ylides as the coupling partner, novel methodologies using α-carbonyl sulfoxonium ylides as the directing group for Rh(III)-catalyzed C-H activation were reported recently (Scheme 1B) (Xu et al., 2017b; Chen X. et al., 2019; Hanchate et al., 2019; Lou et al., 2019; Wang et al., 2019; Wu X. et al., 2019; Yu Y. et al., 2019; Kommagalla et al., 2020).

In most cases the sulfoxonium ylide functioned as a traceless bifunctional directing group, which were removed in terms of DMSO elimination during the course of annulation with alkynes (Xu et al., 2017b; Hanchate et al., 2019; Yu Y. et al., 2019), anthranils (Wu X. et al., 2019), allenoates (Lou et al., 2019), and alkenes (Kommagalla et al., 2020). However, when using oxa/azabicyclic olefins as coupling partners, chemo-divergent couplings were achieved by the Li group (Wang et al., 2019), and the sulfoxonium ylide moiety was retained in the C-H alkylation product that controlled by the introduction of PivOH. The retention of the sulfoxonium ylide was also found in a recent work by Fan and coworkers (Chen X. et al., 2019), in which the naphthalenone derivatives were synthesized from Rh(III)-catalyzed cascade reactions of sulfoxonium ylides with α-diazocarbonyl compounds.

As a continuation of our interest in synthetic and mechanistic study of transition metal-catalyzed C-H activations (Xu et al., 2012; Gao et al., 2015; Guo and Xia, 2015; Guo et al., 2015; Zhou et al., 2015; Chen et al., 2016; Wang et al., 2017, 2018; Pan et al., 2018; Xie et al., 2018a; Xie H. et al., 2018), in the current report we present a combined experimental and theoretical study of ruthenium(II)-catalyzed homocoupling of α-carbonyl sulfoxonium ylides, affording a variety of isocoumarin derivatives under mild conditions (Scheme 1C). (Liang et al., 2018; Xu et al., 2018; Huang et al., 2019; Zhou et al., 2019; Wen et al., 2020; Zhu et al., 2020). DFT calculations (Shan et al., 2016, 2018; Yu et al., 2017; Lian et al., 2019; Ling et al., 2019) suggested that the reaction is realized by a formal [3+3] annulation initiated by Ru(II)-catalyzed C-H activation. (Ackermann, 2011; Davies et al., 2017; Nareddy et al., 2017). It was found that the formation of a free *ortho*-acylmethylated intermediate is not essential for the final cyclization via C-O coupling, and the important roles of Ru(II) and acid additive for promoting the intramolecular nucleophilic substitution were disclosed.

## Results and Discussion

We initiated the investigation by optimizing the reaction conditions for the homocoupling of α-carbonyl sulfoxonium ylide **1a** to form isocoumarin **2a** (Table 1) under Ru(II) catalysis. It was found that 36% of the NMR yield of **2a** could be obtained when the reaction was catalyzed by 5 mol% of [RuCl_2_(*p*-cymene)]_2_ with 0.2 equivalent AgOAc and 1 equivalent KOAc in trifluroethanol solution under air atmosphere at room temperature (entry 1). No reaction was observed if the catalyst was changed to RuCl_2_(PPh_3_)_3_, [Cp^*^Rh(CH_3_CN)_3_SbF_6_]_2_, or [Cp^*^RhCl_2_]_2_ (entry 2). Similar or worse yields resulted if the AgOAc is replaced by other silver salts (entries 3–6).

**Table 1:**
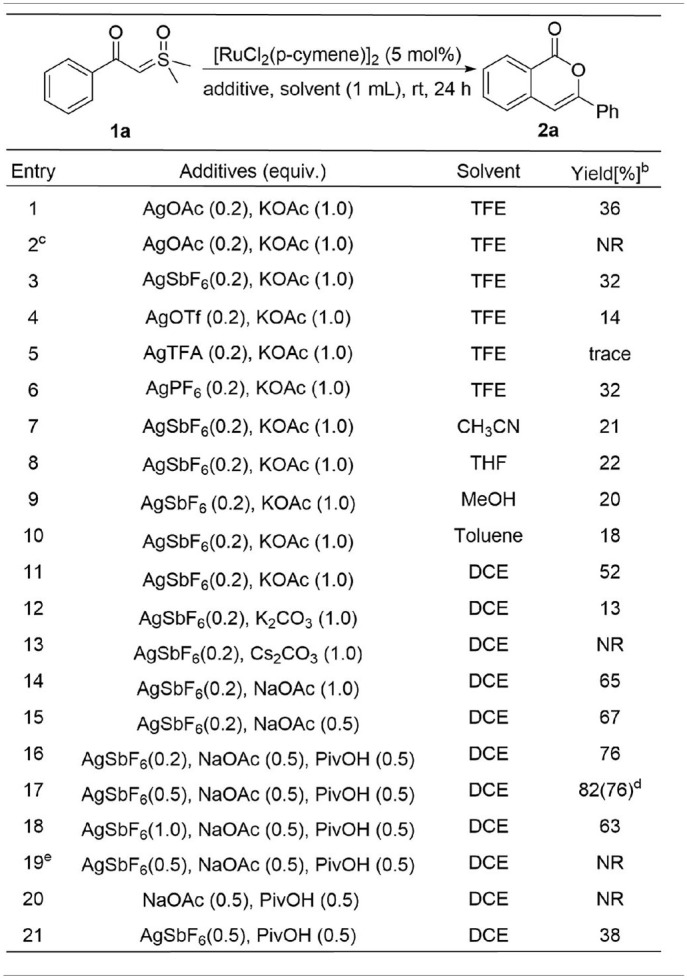
Optimization of reaction conditions*^a^*.

a *Reaction condition: **1a** (0.2 mmol), [RuCl_2_(p-cymene)]_2_ (5 mol%), AgSbF_6_ (0.5 equiv), PivOH (0.5 equiv), NaOAc (0.5 equiv), DCE (1 mL), rt, 24 h*.

b *Determined by ^1^H NMR with mesitylene as the internal standard*.

c Using RuCl_2_(PPh_3_)_3_, Cp^*^Rh(CH_3_CN)_3_(SbF_6_)_2_, or [Cp^*^RhCl_2_]_2_ instead of [RuCl_2_(p-cymene)]_2_.

d *Isolated yield in parenthesis*.

e *Without [RuCl_2_(p-cymene)]_2_*.

Among different solvents screened with AgSbF_6_ as the silver additive (entries 7–11), dichloroethane was found to be the most effective to afford a 52% yield in **2a**. Based on this result, we changed the KOAc additive to K_2_CO_3_ and Cs_2_CO_3_ but no positive result was obtained (entries 12–13). However, improvement of the yield to 65% could be achieved when using 1 equivalent NaOAc instead of KOAc (entry 14), and a similar yield was obtained if the amount of NaOAc was reduced to 0.5 equivalent (entry 15). A better reaction was found by adding 0.5 equivalent pivalic acid to the system (entry 16), and an 82% NMR yield of **2a** was obtained by increasing the AgSbF_6_ to 0.5 equivalent (entry 17). However, the yield would decrease if the AgSbF_6_ was increased to 1 equivalent (entry 18). Control experiments showed that both the Ru(II) and silver salt are essential to the homocoupling (entries 19–20), and the efficiency of the reaction would be dramatically reduced in the absence of NaOAc (entry 21).

With the optimal conditions in hand, the scope of this ruthenium(II)-catalyzed homocoupling protocol with respect to different α-carbonyl sulfoxonium ylide derivatives was investigated (Table 2). While the **2a** was isolated in a 76% yield in reaction of **1a**, the substitution of electron-donating methyl, ethyl, and *t-*butyl groups at the *para* position of the benzene ring was found to have positive effects on the efficiency, affording the corresponding isocoumarins **2b**-**d** in good yields. However, other substrates with other electron donating groups, including chloromethyl, methoxyl, phenyl, trifluoromethoxyl, and trifluoromethylthio, resulted in slightly lower yields of products **2e**-**2i**. The *meta* methyl group in **1j** does not have notable influence on the formation of **2j**, however, substrate **1k**, having an *ortho* methoxyl group, delivered the **2k** in moderate yield, probably due to the steric effect of the substituent in this case. When both *meta* positions of **1l** are substituted by methyl groups, a 58% yield of **2l** was isolated.

**Table 2:**
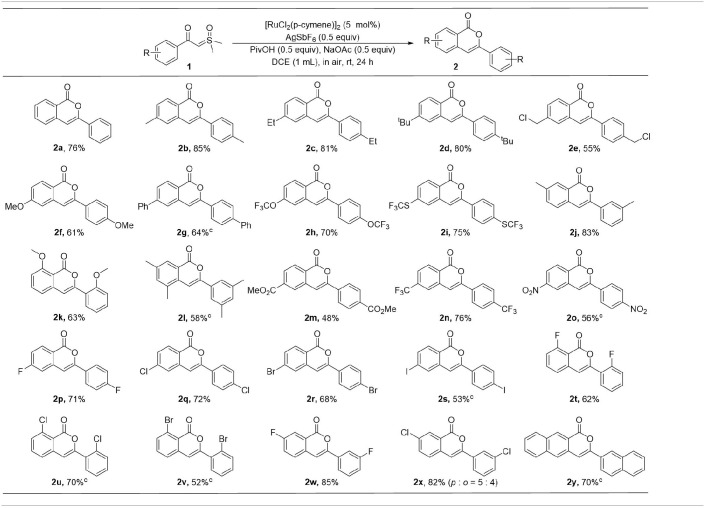
Variation of α-carbonyl sulfoxonium ylides^a^^,^^b^.

a *Reaction conditions: **1a** (0.2 mmol), [RuCl_2_(p-cymene)]_2_ (5 mol%), AgSbF_6_ (0.5 equiv), PivOH (0.5 equiv), NaOAc (0.5 equiv), DCE (1 mL), air, rt, 24 h*.

b *Isolated yields*.

c *Reactions were carried out at 80 °C with a 10 mol% catalyst*.

The effects of electron-withdrawing group on the reactions were also investigated. When the α-carbonyl sulfoxonium ylides were substituted by ester, trifluoromethyl, or nitro group at the *para* position, the desired products were obtained in 48–76% of yields (**2m**-**2o**). Various halides could be tolerated in the reactions, delivering the products in moderate to good yields (**2p**-**2x**). While the *meta*-fluoro-substituted precursor **1w** underwent *para* C-H activation selectively, interestingly, poor selectivity was observed in the reaction with the chloro-containing analog **1x**, forming an 82% yield of isolable products **2x-*p***and **2x-*o***in 5:4 ratio. The toleration of halogens could be useful for further functionalization of the products. In addition, ylide **1y** containing the naphthalene ring was also compatible, affording a 70% yield of the **2y**.

**Scheme 2:**
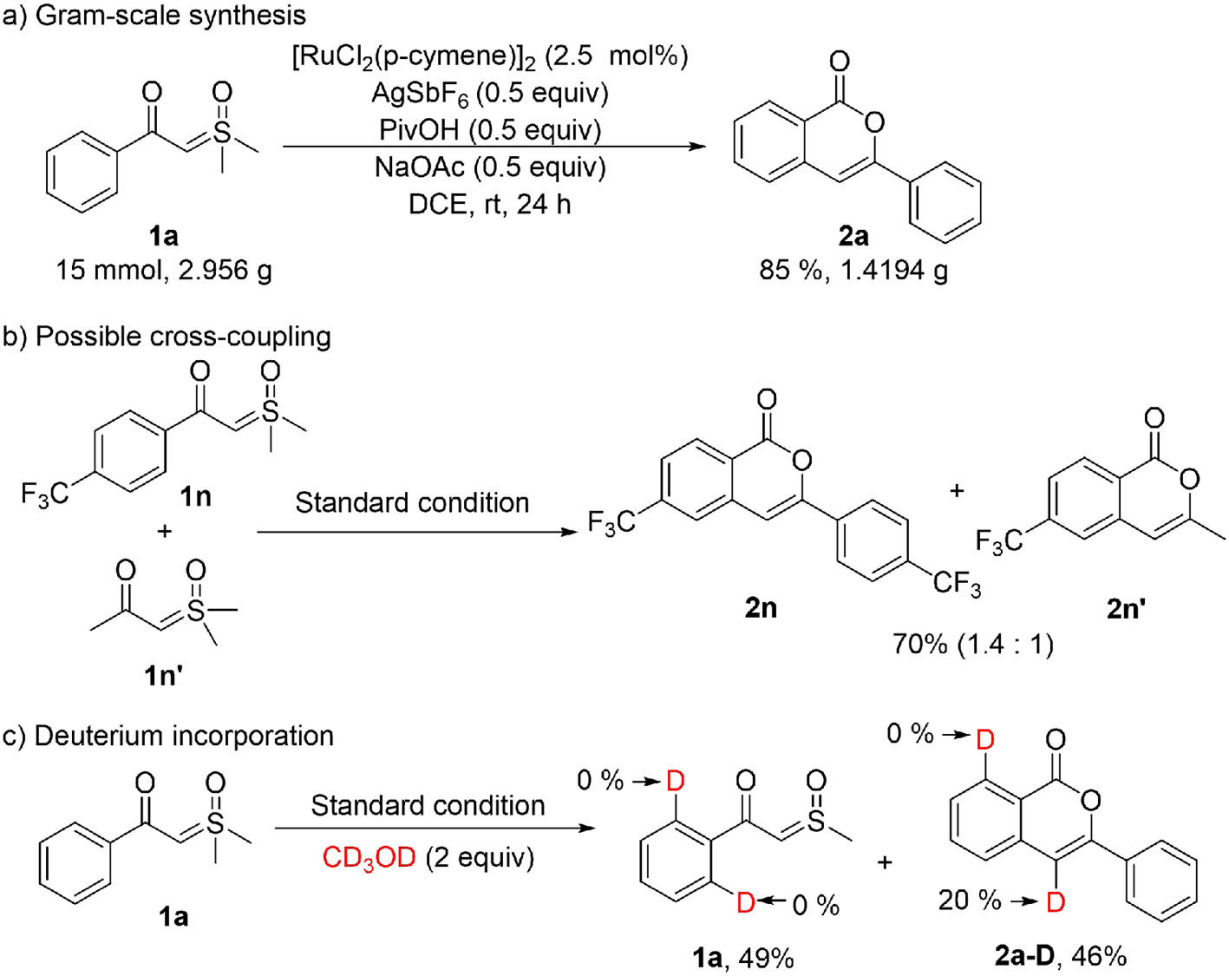
Synthetic application and control experiments.

To show the synthetic application of the catalytic homo-coupling, a gram-scale synthesis of **2a** was performed, and a high yield was achieved with a reduced loading of the Ru(II) catalyst (Scheme 2A). The cross-coupling between aromatic and alkyl α-carbonyl sulfoxonium ylides was tested by the reaction of an equimolar mixture of **1n** and **1n'** under standard conditions (Scheme 2B), which resulted in **2n** and **2n'** in a 1.4:1 ratio, indicating that introducing an alkyl group at C4 of the isocoumarin is possible (more examples are given in the SI). To probe the reaction mechanism, a deuterium labeling experiment was carried out with **1a** in presence of 2 equiv of CD_3_OD (Scheme 2C). After 4 h, a 49% yield of **2a-D** was isolated, in which deuterium incorporation only occurred at C4, but no deuterium incorporation was observed in the recovered **1a**. This indicated that the C-H activation step should be irreversible under the current conditions.

**Figure 1:**
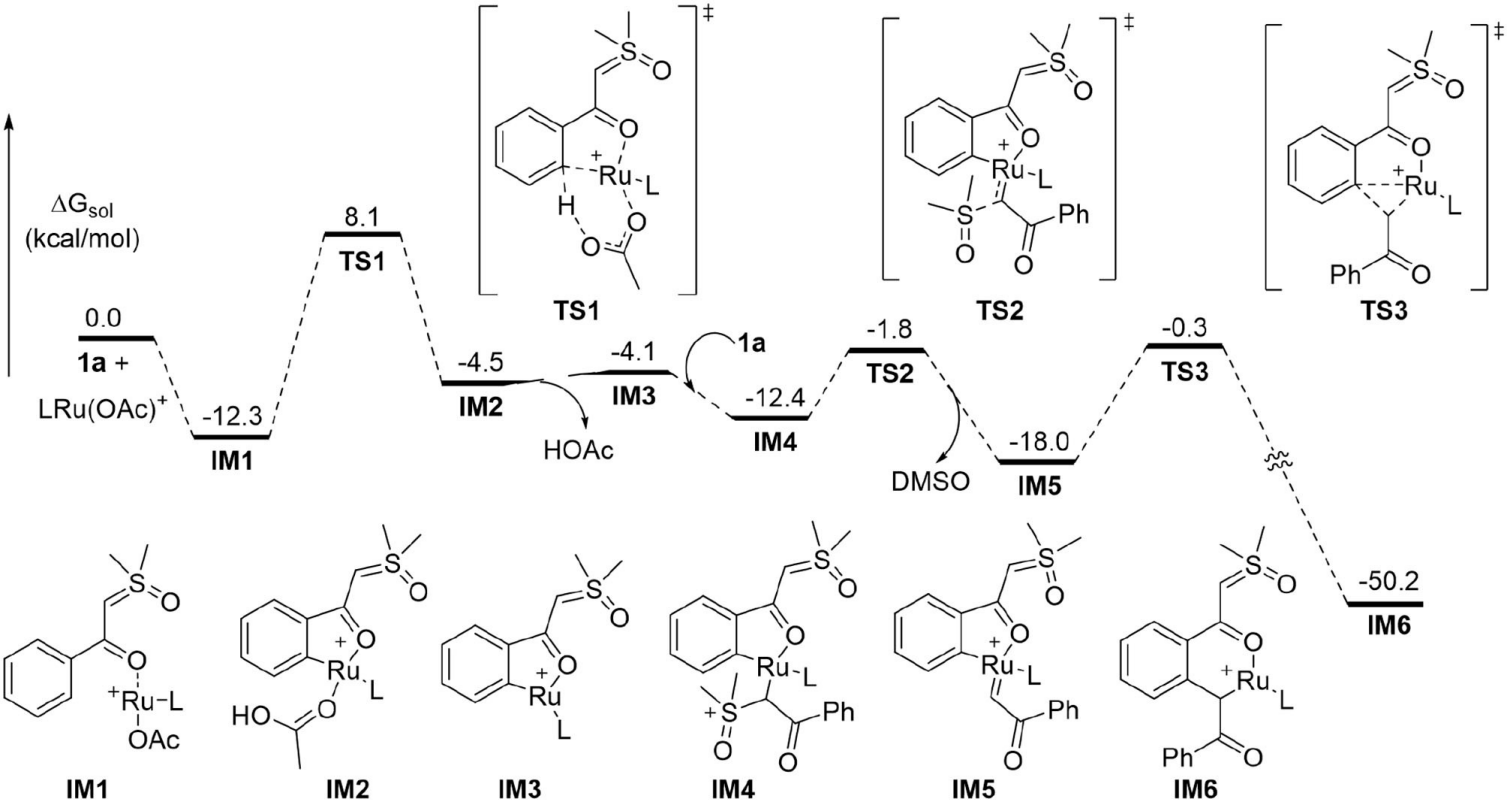
DFT Results for the Formation of Six-membered Ruthenacycle (L = *p*-cymene).

**Figure 2:**
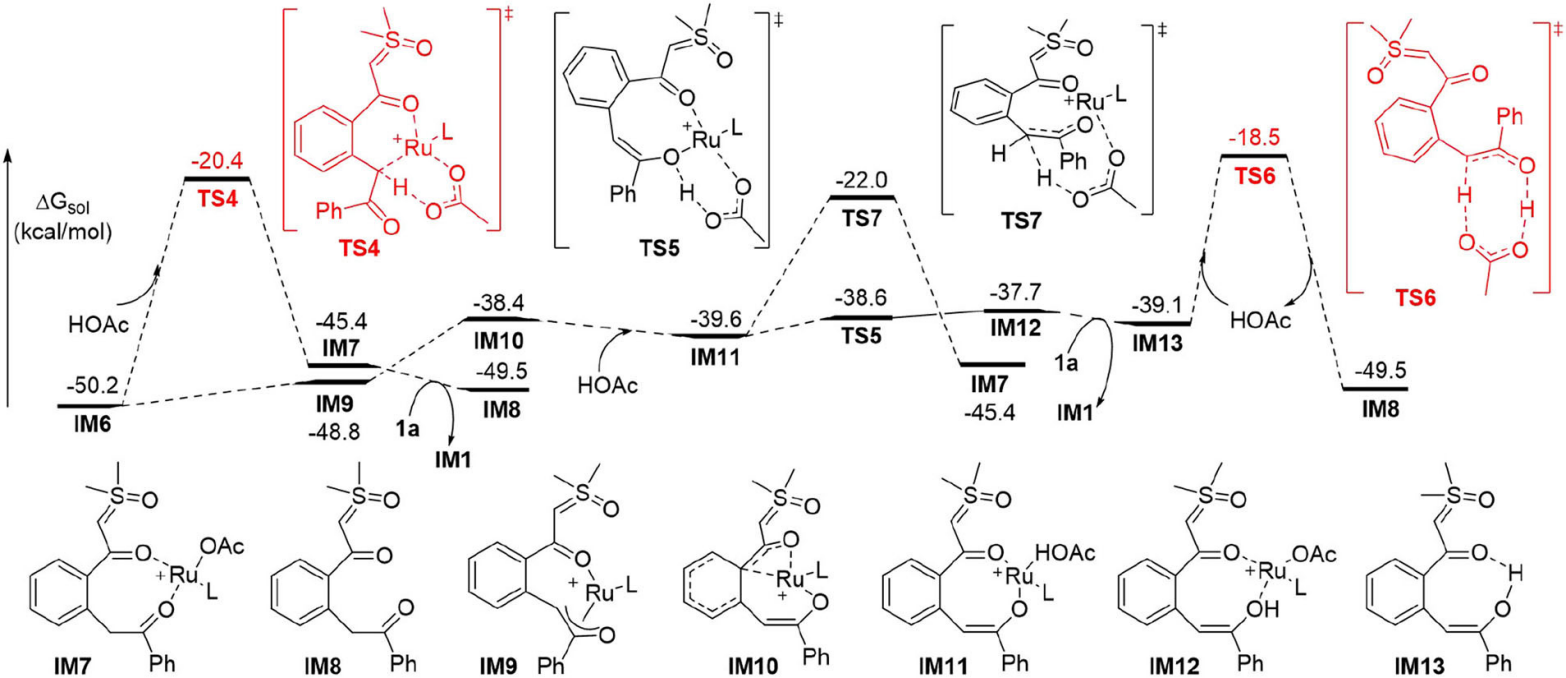
DFT Results for the Generation of the Acylmethylation Intermediate (L = *p*-cymene).

**Figure 3:**
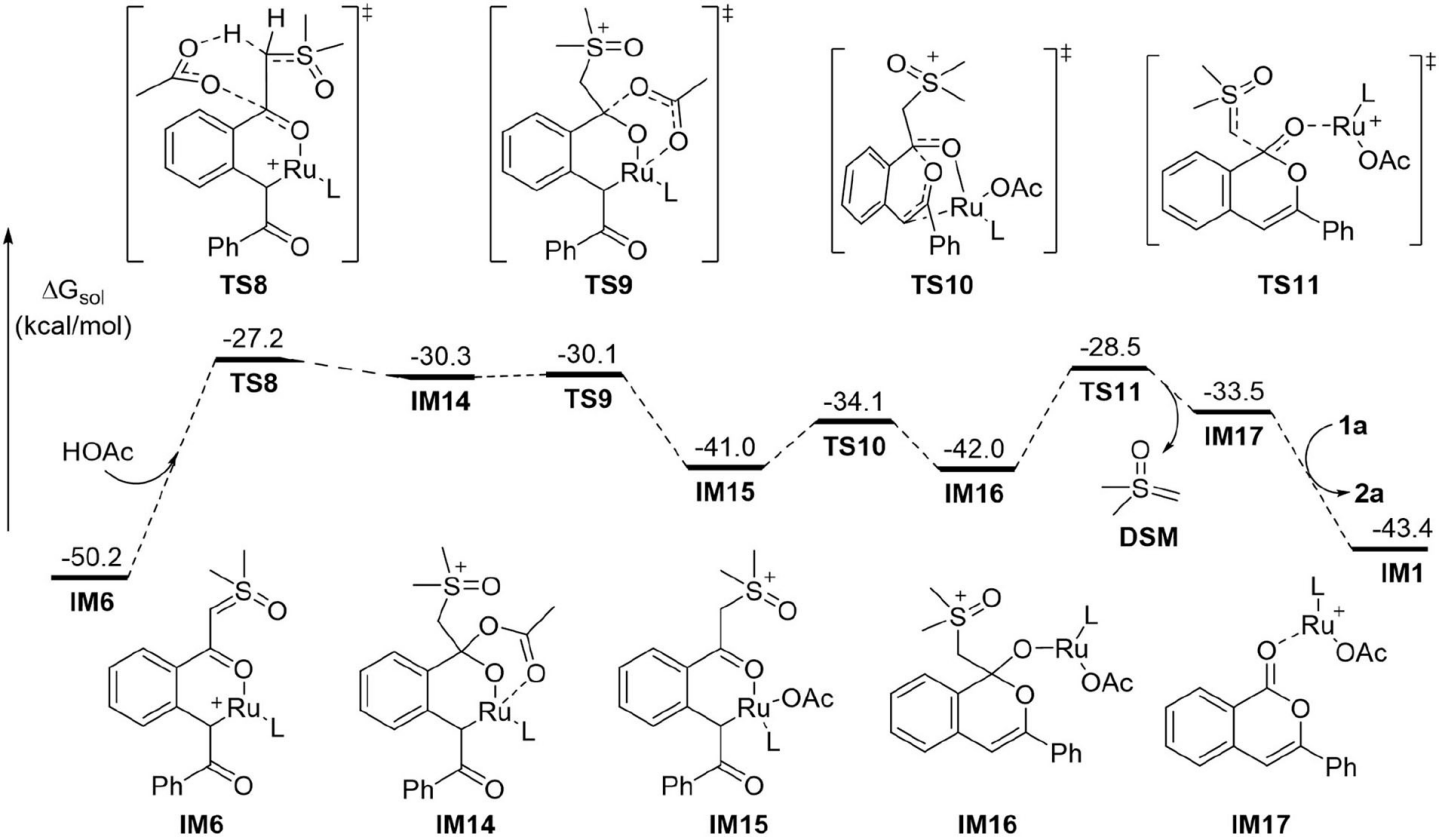
DFT Results for the Formation of 2a from IM6 (L = *p*-cymene).

To better understand the experimental results, DFT calculations were carried out to highlight the details of the transformation (Figures 1–3) (Hou et al., 2017; Jiang J. et al., 2019; Shu et al., 2019). According to the theoretical results, the reactant complex **IM1** formed exergonically from substrate **1a** and cationic monomeric LRu(OAc)^+^ (L = *p*-cymene), which was produced in the catalytic system of [RuCl_2_(*p*-cymene)]_2_, AgSbF_6_, and NaOAc (Figure 1) (Xie et al., 2018a; Xie H. et al., 2018). Calculations found that if the neutral complex LRu(OAc)_2_ was used, one anionic ligand should be dissociated from the Ru(II) to form a stable reactant complex, indicating a generation of cationic species is more favorable. From **IM1**, the *ortho*-C-H cleavage directed by the carbonyl functionality occurs via the CMD process (**TS1**) with an activation barrier of 20.4 kcal/mol and leads to metallated intermediate **IM2** endergonically. **IM3** is formed by releasing HOAc prior to the incorporation of another **1a** to form complex **IM4** through interaction between the ylide carbon and the Ru(II). From the latter intermediate, the C-S cleavage via **TS2** becomes facile with a small barrier of 10.6 kcal/mol. This step forms Ru-carbene intermediate **IM5** slightly exergonically and eliminates DMSO concurrently. The migratory insertion of the carbene moiety into the Ru-C bond requires a barrier of 17.7 kcal/mol via **TS3**. The profile in Figure 1 disclosed that **TS2** and **TS3** are much lower in energy than **TS1** and the formation of the six-membered ruthenocycle **IM6** is highly exergonic, suggesting that the C-H activation step is irreversible and is consistent with the deuterium-labeling experiment.

It was generally proposed that the cyclic product was formed by the first generation of an acylmethylation intermediate in similar cascade reactions. (Chen et al., 2018; Hoang and Ellman, 2018; Hoang et al., 2018; Hu et al., 2018a,b, 2019; Liang et al., 2018; Shi et al., 2018; Xiao et al., 2018; Xie et al., 2018b, 2019; Xie H. et al., 2018; Xu et al., 2018; Zhou et al., 2018; Cai et al., 2019; Chen P. et al., 2019; Cui et al., 2019; Huang et al., 2019; Lai et al., 2019; Liu et al., 2019; Luo et al., 2019; Lv et al., 2019; Nie et al., 2019; Shen et al., 2019; Wang and Xu, 2019; Wu C. et al., 2019; Zhang et al., 2019, 2020; Wu et al., 2020). Further transformations from **IM6** were explored theoretically to confirm whether the acylmethylation intermediate (**IM8**) is key in the formation of **2a** (Figure 2). It was found that the direct protodemetallation of **IM6** with HOAc is relatively difficult to achieve with a barrier of 29.8 kcal/mol via **TS4**, albeit the formation of **IM8** is thermodynamically possible via a ligand displacement of complex **IM7** with **1a**. The possible involvement of an enol intermediate was also studied. The η^3^ oxallyl complex **IM9** and O-bound enolate complex **IM10** are 1.4 and 11.8 kcal/mol higher in energy than C-bound enolate complex **IM6**, respectively. The high energy of **IM10** is probably due to the strong interaction between the Ru(II) and the phenyl group, which leads to a puckered structure and dearomatization of the phenyl ring. The complexation of HOAc with **IM10** forms **IM11** by H-bonding, the proton transfer from HOAc to the enolate oxygen is very facile with a barrier of 1.0 kcal/mol via **TS5** and generates the complex **IM12** slightly endergonically. The free enol intermediate **IM13**, 11.1 kcal/mol higher in energy than **IM6**, could be released by the incorporation of another **1a**, from which the reactant complex **IM1** is regenerated. Tautomerism between **IM13** and **IM8** could be possible via an intramolecular process involving HOAc as the proton shuttle as shown in **TS6** with an activation barrier of 20.6 kcal/mol, while tautomerism by intramolecular 1,3-H shift requires a much higher barrier of 48.9 kcal/mol from **IM13** (See **SI** for more details). However, the energy of **TS6** is 31.7 kcal/mol above that of the global minimum **IM6**^1^. It was supposed that the protonation of the α carbon of **IM11** could be another possible pathway to complex **IM7**. This could be realized via **TS7**, but a relatively high activation barrier of 28.2 kcal/mol is still required from **IM6**.

While the above results indicated that the generation of acylmethylation intermediate **IM8** should be difficult under current conditions^1^, we found the pathway initiated by protonation of the anionic ylide carbon in **IM6** by HOAc is the most energetically favorable (Figure 3). Accordingly, the barrier for the protonation via **TS8** is 23.0 kcal/mol, leading endergonically to **IM14** in which the acetate is associated with both the carbonyl carbon and the Ru atom. **IM14** undergoes a very facile C-O dissociation via **TS9** to form **IM15**, from which the intramolecular nucleophilic addition via **TS10** requires a small barrier of 6.9 kcal/mol and forms the C-O bond of the 6-membered heterocycle in intermediate **IM16**. In the following step, C-C bond cleavage occurs via **TS11** with a barrier of 13.5 kcal/mol, this generates product complex **IM17** and eliminates dimethylsulfoxonium methylide (DSM) concurrently. In the last step the formation of product **2a** and regeneration of reactant complex **IM1** could be realized by a ligand exchange reaction of **IM17** with **1a**. Thus, the protonation of the ylide carbon by HOAc via **TS8** is the most difficult step in the whole reaction. This explains why the acid additive is required for promoting the reaction.

**Figure 4:**
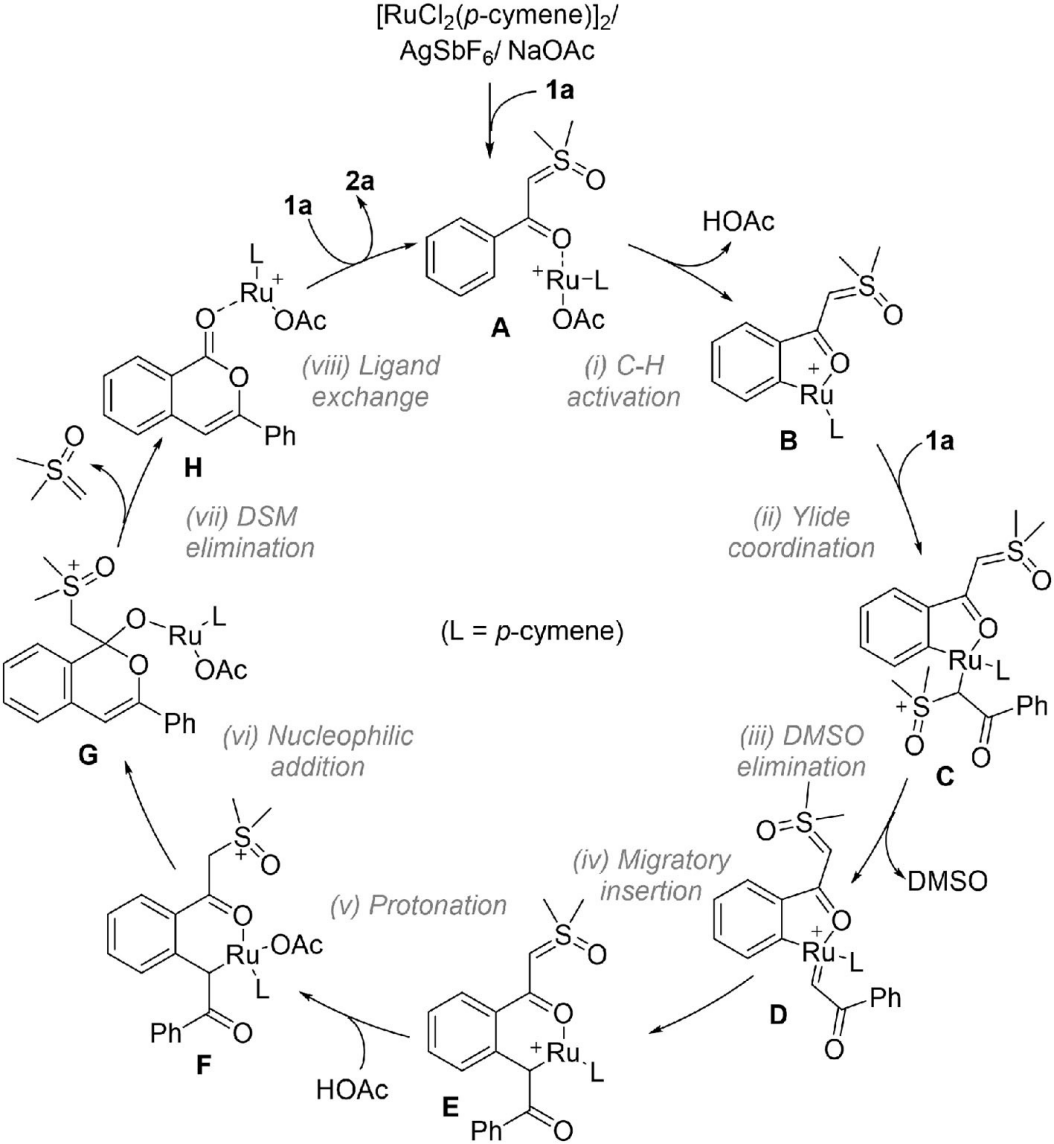
Proposed catalytic cycle based on DFT results.

Based on the above results, the full catalytic cycle for the transformation contains eight elementary steps as shown in Figure 4. Upon the formation of cationic reactant complex **A**, the first step is the acetate-assisted C-H activation to form a five-membered ruthenacycle **B**. The incorporation of another **1a** by ylide coordination generates σ-complex **C**, which undergoes DMSO elimination to form carbene intermediate **D**. From this, C-C bond formation by migratory insertion generates a six-membered ruthenocycle **E**. Then, protonation of the ylide carbon by HOAc leads to intermediate **F**. The following step is an intramolecular nucleophilic addition which creates **G**, from which the DSM elimination by C-C cleavage occurs to deliver product complex **H**. In the last step, releasing isocoumarin product **2a** and regenerating complex **A** is completed by a ligand exchange.

## Conclusion

In conclusion, we have established a mild ruthenium(II)-catalyzed homocoupling of α-carbonyl sulfoxonium ylides and carried out a detailed mechanistic investigation using DFT calculations. The methodology enables the efficient synthesis of a variety of isocoumarin derivatives under air conditions at room temperature. Theoretical results uncovered that the Ru(II) catalyst is involved in all steps of C-H activation, C-C coupling, C-O formation, and C-C cleavage, and the intermediacy of free acylmethylation intermediate or its enol isomer was not necessary for the intramolecular nucleophilic cyclization process. The mechanistic information could have implications for better understanding related tandem reactions in other catalytic systems.

## Experimental Section

### Computational Details

All DFT calculations were carried out with the Gaussian 09 suite of computational programs (Frisch et al., 2013). The geometries of all stationary points were optimized using the B3LYP hybrid functional (Lee et al., 1988; Becke, 1993a,b) at the basis set level of 6-31G(d) for all atoms except for Ru, which was described by the relativistic effective core potential basis set of Lanl2dz. Frequencies were analytically computed at the same level of theory to obtain the free energies and to confirm whether the structures were minima (no imaginary frequency) or transition states (only one imaginary frequency). The solvent effect of toluene was evaluated by using the SMD polarizable continuum model by carrying out single point calculations at the M06/6-311+G(d,p) (SDD fur Ru) level (Zhao and Truhlar, 2008a,b). All transition state structures were confirmed to connect the proposed reactants and products by intrinsic reaction coordinate (IRC) calculations. All the energies given in the text are relative free energies corrected with solvation effects.

## Materials and Methods

Commercially available materials were used as received, unless otherwise noted. ^1^H NMR and ^13^C NMR spectra were measured on a Bruker-400 or Bruker-500 instrument, using CDCl_3_ as the solvent with tetramethylsilane (TMS) as an internal standard at room temperature. Chemical shifts are given in δ relative to TMS, the coupling constants *J* are given in Hz. Melting points were measured on an X4 melting point apparatus and uncorrected. HRMS analysis was measured on a Bruker micrOTOF-Q II instrument (ESI) or a Waters GCT Premier instrument (EI-TOF).

### Typical Procedure for the Synthesis of α-Carbonyl Sulfoxonium Ylides 1

Under N_2_, trimethylsulfur iodide (3.3 g, 15 mmol, 3 equiv) was suspended in dry THF (25 mL) in a flame-dried 100 mL round bottom flask that was protected from light with aluminum foil. Potassium *tert*-butoxide (2.24 g, 20 mmol, 4 equiv) was added and the mixture was stirred at reflux for 2 h. After cooling to room temperature, benzoyl chloride (5 mmol, 1 equiv) in THF (10 mL) was added. The mixture was stirred at reflux for another hour and then filtered at room temperature through a plug of celite before all volatiles were removed under vacuum. Purification by flash chromatography afforded sulfur ylide **1a**.

**2-(dimethyl(oxo)-λ^6^-sulfanylidene)-1-phenylethan-1-one(1a)** (Xiao et al., 2018). ^1^H NMR (400 MHz, CDCl_3_) δ 7.83–7.76 (m, 2H), 7.47–7.35 (m, 3H), 5.01 (s, 1H), 3.50 (s, 6H).

**2-(dimethyl(oxo)-λ^6^-sulfanylidene)-1-(p-tolyl)ethan-1-one(1b)** (Xiao et al., 2018). ^1^H NMR (500 MHz, CDCl_3_) δ 7.70 (d, *J* = 6.0 Hz, 2H), 7.19 (d, *J* = 6.0 Hz, 2H), 4.95 (s, 1H), 3.53–3.49 (m, 6H), 2.37 (d, *J* = 5.5 Hz, 3H).

**2-(dimethyl(oxo)-λ^6^-sulfanylidene)-1-(4-ethylphenyl)ethan-1-one(1c)** (Xiao et al., 2018). ^1^H NMR (400 MHz, CDCl_3_) δ 7.72 (d, *J* = 8.0 Hz, 2H), 7.22 (d, *J* = 8.0 Hz, 2H), 4.96 (s, 1H), 3.50 (s, 6H), 2.67 (q, *J* = 7.6 Hz, 2H), 1.24 (t, *J* = 7.6 Hz, 3H).

**1-(4-(tert-butyl)phenyl)-2-(dimethyl(oxo)-λ^6^-sulfanylidene)ethan-1-one(1d)** (Neuhaus et al., 2018). ^1^H NMR (400 MHz, CDCl_3_) δ 7.74 (d, *J* = 8.0 Hz, 2H), 7.41 (d, *J* = 8.4 Hz, 2H), 4.95 (s, 1H), 3.51 (s, 6H), 1.33 (s, 9H).

**2-(dimethyl(oxo)-λ^6^-sulfanylidene)-1-(4-methoxyphenyl)ethan-1-one(1e)** (Xiao et al., 2018). ^1^H NMR (400 MHz, CDCl_3_) δ 7.77 (d, *J* = 8.0 Hz, 2H), 6.89 (d, *J* = 8.0 Hz, 2H), 4.91 (s, 1H), 3.84 (d, *J* = 0.8 Hz, 3H), 3.51 (d, *J* = 0.8 Hz, 6H).

**1-(4-(chloromethyl)phenyl)-2-(dimethyl(oxo)-λ^6^-sulfanylidene)ethan-1-one(1f)**. White solid (m.p. = 137.3- 138.5°C). ^1^H NMR (500 MHz, CDCl_3_) δ 7.75 (d, *J* = 8.0 Hz, 2H), 7.42 (d, *J* = 8.5 Hz, 2H), 5.81 (d, *J* = 17.5 Hz, 1H), 5.30 (d, *J* = 11.0 Hz, 1H), 4.99 (s, 1H), 3.50 (s, 6H); ^13^C NMR (125 MHz, CDCl_3_) δ 181.8, 139.8, 138.2, 136.3, 128.3, 126.9, 126.8, 125.9, 115.0, 68.4, 42.4. HRMS (ESI-TOF) calculated for C_11_H_13_ClO_2_SNa [M+Na] 267.0217; found 267.0231.

**1-([1,1'-biphenyl]-4-yl)-2-(dimethyl(oxo)-λ^6^-sulfanylidene)ethan-1-one(1g)** (Jiang H. F. et al., 2019) ^1^H NMR (400 MHz, CDCl_3_) δ 7.87 (d, *J* = 8.8 Hz, 2H), 7.64–7.61 (m, 4H), 7.47–7.42 (m, 2H), 7.38–7.34 (m, 1H), 5.03 (s, 1H), 3.53 (s, 6H).

**2-(dimethyl(oxo)-λ^6^-sulfanylidene)-1-(4-(trifluoromethoxy)phenyl)ethan-1-one(1h)** (Jiang H. F. et al., 2019). ^1^H NMR (400 MHz, CDCl_3_) δ 7.88-7.70 (m, 2H), 7.22 (d, *J* = 8.8 Hz, 2H), 4.97 (s, 1H), 3.52 (s, 6H).

**2-(dimethyl(oxo)-λ^6^-sulfanylidene)-1-(4-((trifluoromethyl)thio)phenyl)ethan-1-one(1i)** (Vaitla et al., 2017). ^1^H NMR (400 MHz, CDCl_3_) δ 7.82 (d, *J* = 8.0 Hz, 2H), 7.67 (d, *J* = 8.0 Hz, 2H), 5.01 (s, 1H), 3.53 (s, 6H).

**2-(dimethyl(oxo)-λ^6^-sulfanylidene)-1-(m-tolyl)ethan-1-one(1j)** (Jiang H. F. et al., 2019). ^1^H NMR (400 MHz, CDCl_3_) δ 7.63 (s, 1H), 7.58 (d, *J* = 7.2 Hz, 1H), 7.33-7.17 (m, 2H), 4.99 (s, 1H), 3.50 (s, 6H), 2.38 (s, 3H).

**2-(dimethyl(oxo)-λ^6^-sulfanylidene)-1-(2-methoxyphenyl)ethan-1-one(1k)** (Vaitla et al., 2017). ^1^H NMR (400 MHz, CDCl_3_) δ 7.91–7.88 (m, 1H), 7.41-7.31 (m, 1H), 7.02–6.99 (m, 1H), 6.92 (d, *J* = 8.4 Hz, 1H), 5.32 (s, 1H), 3.89 (s, 3H), 3.52 (s, 6H).

**2-(dimethyl(oxo)-λ^6^-sulfanylidene)-1-(3,5-dimethylphenyl)ethan-1-one(1l)** (Neuhaus et al., 2018). ^1^H NMR (400 MHz, CDCl_3_) δ 7.42 (s, 2H), 7.07 (s, 1H), 4.96 (s, 1H), 3.50 (s, 6H), 2.34 (s, 6H).

**methyl 4-(2-(dimethyl(oxo)-λ^6^-sulfanylidene)acetyl)benzoate(1m)** (Phelps et al., 2016). ^1^H NMR (400 MHz, CDCl_3_) δ 8.06 (d, *J* = 8.4 Hz, 2H), 7.84 (d, *J* = 8.4 Hz, 2H), 5.04 (s, 1H), 3.93 (s, 3H), 3.54 (s, 6H).

**2-(dimethyl(oxo)-λ^6^-sulfanylidene)-1-(4-(trifluoromethyl)phenyl)ethan-1-one(1n)** (Jiang H. F. et al., 2019). ^1^H NMR (400 MHz, CDCl_3_) δ 7.89 (d, *J* = 8.0 Hz, 2H), 7.65 (d, *J* = 8.4 Hz, 2H), 5.02 (s, 1H), 3.54 (s, 6H).

**2-(dimethyl(oxo)-λ^6^-sulfanylidene)-1-(4-nitrophenyl)ethan-1-one(1o)**(Vaitla et al., 2017). ^1^H NMR (400 MHz, CDCl_3_) δ 8.25 (d, *J* = 8.4 Hz, 2H), 7.93 (d, *J* = 8.4 Hz, 2H), 5.04 (s, 1H), 3.55 (s, 6H).

**2-(dimethyl(oxo)-l6-sulfanylidene)-λ^6^-(4-fluorophenyl)ethan-1-one(1p)** (Jiang H. F. et al., 2019). ^1^H NMR (400 MHz, CDCl_3_) δ 7.85–7.67 (m, 2H), 7.11-6.97 (m, 2H), 4.93 (s, 1H), 3.51 (s, 6H).

**1-(4-chlorophenyl)-2-(dimethyl(oxo)-λ^6^-sulfanylidene)ethan-1-one(1q)** (Xiao et al., 2018) ^1^H NMR (400 MHz, CDCl_3_) δ 7.73 (d, *J* = 8.4 Hz, 2H), 7.36 (d, *J* = 8.4 Hz, 2H), 4.96 (s, 1H), 3.51 (s, 6H).

**1-(4-bromophenyl)-2-(dimethyl(oxo)-λ^6^-sulfanylidene)ethan-1-one(1r)** (Vaitla et al., 2017). ^1^H NMR (400 MHz, CDCl_3_) δ 7.65 (d, *J* = 8.4 Hz, 2H), 7.51 (d, *J* = 8.4 Hz, 2H), 4.99 (s, 1H), 3.51 (s, 6H).

**2-(dimethyl(oxo)-λ^6^-sulfanylidene)-1-(4-iodophenyl)ethan-1-one(1s)** (Jiang H. F. et al., 2019). ^1^H NMR (400 MHz, CDCl_3_) δ 7.84–7.62 (m, 2H), 7.58–7.42 (m, 2H), 4.95 (s, 1H), 3.51 (d, *J* = 1.2 Hz, 6H).

**2-(dimethyl(oxo)-λ^6^-sulfanylidene)-1-(2-fluorophenyl)ethan-1-one(1t)** (Neuhaus et al., 2018). ^1^H NMR (500 MHz, CDCl_3_) δ 7.92–7.89 (m, 1H), 7.40–7.35 (m, 1H), 7.21–7.17 (m, 1H), 7.08–7.03 (m, 1H), 5.17 (s, 1H), 3.53 (s, 6H).

**1-(2-chlorophenyl)-2-(dimethyl(oxo)-λ^6^-sulfanylidene)ethan-1-one(1u)** (Xiao et al., 2018). ^1^H NMR (400 MHz, CDCl_3_) δ 7.56–7.38 (m, 1H), 7.41–7.30 (m, 1H), 7.29–7.23 (m, 2H), 4.76 (s, 1H), 3.53 (s, 6H).

**1-(2-bromophenyl)-2-(dimethyl(oxo)-λ^6^-sulfanylidene)ethan-1-one(1v)** (Vaitla et al., 2017). ^1^H NMR (400 MHz, CDCl_3_) δ 7.56–7.53 (m, 1H), 7.45–7.41 (m, 1H), 7.34–7.24 (m, 1H), 7.21–716 (m, 1H), 4.67 (s, 1H), 3.54 (s, 6H).

**2-(dimethyl(oxo)-λ^6^-sulfanylidene)-1-(3-fluorophenyl)ethan-1-one(1w)** (Jiang H. F. et al., 2019). ^1^H NMR (400 MHz, CDCl_3_) δ 7.57–7.48 (m, 2H), 7.35 (d, *J* = 6.0 Hz, 1H), 7.15–7.10 (m, 1H), 4.97 (s, 1H), 3.52 (s, 6H).

**1-(3-chlorophenyl)-2-(dimethyl(oxo)-λ^6^-sulfanylidene)ethan-1-one(1x)** (Jiang H. F. et al., 2019). ^1^H NMR (400 MHz, CDCl_3_) δ 7.78–7.76 (m, 1H), 7.67–7.64 (m, 1H), 7.41–7.38 (m, 1H), 7.34–7.29 (m, 1H), 4.98 (s, 1H), 3.51 (s, 6H).

**2-(dimethyl(oxo)-λ^6^-sulfanylidene)-1-(naphthalen-2-yl)ethan-1-one(1y)** (Phelps et al., 2016). ^1^H NMR (400 MHz, CDCl_3_) δ 8.33 (s, 1H), 8.01–7.75 (m, 4H), 7.58–7.42 (m, 2H), 5.13 (s, 1H), 3.56 (s, 6H).

**1-(dimethyl(oxo)-λ^6^-sulfanylidene)-3,3-dimethylbutan-2-one(1z)** (Xiao et al., 2018). ^1^H NMR (400 MHz, CDCl_3_) δ 4.46 (s, 1H), 3.39 (d, *J* = 0.8 Hz, 6H), 1.12 (d, *J* = 1.2 Hz, 9H).

**1-(dimethyl(oxo)-λ^6^-sulfanylidene)propan-2-one(1aa)** (Barday et al., 2017). ^1^H NMR (400 MHz, CDCl_3_) δ 4.40 (s, 1H), 3.40 (s, 6H), 1.95 (s, 3H).

### General Procedure for the Synthesis of Isocoumarins 2

α-carbonyl sulfoxonium ylide (0.2 mmol), [RuCl_2_(p-cymene)]_2_ (0.01 mmol), NaOAc (0.1 mmol), PivOH (0.1 mmol), AgSbF_6_(0.1 mmol), and DCE (1 mL) were added to a 10 mL Schlenk tube charged with a magnetic stirring bar under air atmosphere. The reaction was stirred at room temperature for 24 h. The mixture was then pumped through a suction funnel and through silica gel and washed with mixed EA and PE. The filtrate was concentrated under reduced pressure and purified by flash chromatography on silica gel to create the target homocoupling product (**2**).

**3-phenyl-1H-isochromen-1-one(2a)** (Nandi et al., 2013). Yield: 76% (0.0169 g, 0.152 mmol), white solid. ^1^H NMR (500 MHz, CDCl_3_) δ 8.29 (d, *J* = 8.0 Hz, 1H), 7.87 (d, *J* = 7.5 Hz, 2H), 7.72–7.68 (m, 1H), 7.49–7.40 (m, 5H), 6.93 (s, 1H); ^13^C NMR (125 MHz, CDCl_3_) δ 162.2, 153.6, 137.5, 134.8, 132.0, 129.9, 129.6, 128.8, 128.1, 125.9, 125.2, 120.5, 101.8.

**6-methyl-3-(p-tolyl)-1H-isochromen-1-one(2b)** (Nandi et al., 2013). Yield: 85% (0.0211 g, 0.170 mmol), white solid. ^1^H NMR (500 MHz, CDCl_3_) δ 8.17 (d, *J* = 8.0 Hz, 1H), 7.75 (d, *J* = 8.5 Hz, 2H), 7.31–7.13 (m, 4H), 6.82 (s, 1H), 2.47 (s, 3H), 2.39 (s, 3H); ^13^C NMR (125 MHz, CDCl_3_) δ 162.4, 153.9, 145.8, 140.1, 137.8, 129.6, 129.5, 129.4, 129.3, 125.8, 125.2, 118.1, 101.0, 21.9, 21.3.

**6-ethyl-3-(4-ethylphenyl)-1H-isochromen-1-one(2c)** (Zhou et al., 2019). Yield: 69% (0.0192 g, 0.138 mmol), white solid. ^1^H NMR (500 MHz, CDCl_3_) δ 8.19 (d, *J* = 8.0 Hz, 1H), 7.79 (d, *J* = 8.0 Hz, 2H), 7.38–7.19 (m, 4H), 6.86 (s, 1H), 2.76 (q, *J* = 8.0 Hz, 2H), 2.69 (q, *J* = 7.5 Hz, 2H), 1.30 (t, *J* = 7.5 Hz, 3H), 1.26 (t, *J* = 7.5 Hz, 3H); ^13^C NMR (125 MHz, CDCl_3_) δ 162.4, 153.9, 151.9, 146.4, 137.9, 129.7, 129.6, 128.3, 128.2, 125.2, 124.6, 118.3, 101.2, 29.2, 28.7, 15.2, 14.9.

**6-(tert-butyl)-3-(4-(tert-butyl)phenyl)-1H-isochromen-1-one(2d)** (Zhou et al., 2019). Yield: 80% (0.0268 g, 0.160mmol), white solid. ^1^H NMR (500 MHz, CDCl_3_) δ 8.23 (d, *J* = 8.5 Hz, 1H), 7.82 (d, *J* = 8.5 Hz, 2H), 7.55–7.52 (m, 1H), 7.48 (d, *J* = 8.5 Hz, 3H), 6.93 (s, 1H), 1.39 (s, 9H), 1.35 (s, 9H); ^13^C NMR (125 MHz, CDCl_3_) δ 162.4, 158.8, 153.8, 153.3, 137.7, 129.43, 129.37, 125.9, 125.7, 125.0, 122.2, 118.1, 101.6, 35.4, 34.8, 31.2, 31.0.

**6-methoxy-3-(4-methoxyphenyl)-1H-isochromen-1-one(2e)** (Zhou et al., 2019). Yield: 60% (0.0169 g, 0.120 mmol), white solid. ^1^H NMR (500 MHz, CDCl_3_) δ 8.17 (d, *J* = 8.5 Hz, 1H), 7.78 (d, *J* = 8.5 Hz, 2H), 7.04–6.89 (m, 3H), 6.81 (d, *J* = 2.0 Hz, 1H), 6.74 (s, 1H), 3.90 (s, 3H), 3.85 (s, 3H); ^13^C NMR (125 MHz, CDCl_3_) δ 164.7, 162.1, 161.1, 154.2, 140.2, 131.7, 126.8, 124.6, 116.1, 114.2, 113.3, 107.6, 100.2, 55.6, 55.3.

**6-(chloromethyl)-3-(4-(chloromethyl)phenyl)-1H-isochromen-1-one(2f)**. Yield: 55% (0.0176 g, 0.110 mmol), white solid (m.p. = 99.8–101.8°C). ^1^H NMR (500 MHz, CDCl_3_) δ 8.24 (d, *J* = 8.0 Hz, 1H), 7.83 (d, *J* = 8.5 Hz, 2H), 7.56–7.39 (m, 4H), 6.92 (s, 1H), 5.96 (d, *J* = 17.5 Hz, 1H), 5.83 (d, *J* = 17.5 Hz, 1H), 5.49 (d, *J* = 11.0 Hz, 1H), 5.34 (d, *J* = 11.0 Hz, 1H); ^13^C NMR (125 MHz, CDCl_3_) δ 162.0, 153.7, 143.9, 139.2, 137.9, 136.0, 135.7, 131.2, 130.0, 126.6, 125.7, 125.4, 123.6, 119.6, 118.0, 115.3, 101.7. HRMS (ESI-TOF) calculated for C_17_H_12_Cl_2_O_2_ Na [M+Na] 341.0107; found 341.0111.

**3-([1,1'-biphenyl]-4-yl)-6-phenyl-1H-isochromen-1-one(2g)**. Yield: 64% (0.0239 g, 0.128 mmol), faint yellow solid (m.p. = 222.8–224.5°C). ^1^H NMR (400 MHz, DMSO) δ 8.20 (d, J = 8.4 Hz, 1H), 7.96 (d, J = 6.4 Hz, 3H), 7.91–7.65 (m, 7H), 7.61–7.33 (m, 7H); ^13^C NMR (125 MHz, CDCl_3_) δ 162.2, 153.8, 147.8, 142.8, 140.1, 139.5, 138.1, 130.9, 130.3, 129.1, 128.9, 128.7, 127.9, 127.5, 127.4, 127.2, 127.1, 125.7, 124.2, 119.3, 101.9. HRMS (ESI-TOF) calculated for C_27_H_19_O_2_ [M+H] 375.1380; found 375.1361.

**6-(trifluoromethoxy)-3-(4-(trifluoromethoxy)phenyl)-1H-isochromen-1-one(2h)**. Yield: 70% (0.0273 g, 0.140 mmol), white solid (m.p. = 138–140°C). ^1^H NMR (500 MHz, CDCl_3_) δ 8.35 (d, *J* = 9.5 Hz, 1H), 7.91 (d, *J* = 8.5 Hz, 2H), 7.32 (d, *J* = 7.0 Hz, 4H), 6.92 (s, 1H); ^13^C NMR (125 MHz, CDCl_3_) δ 160.7, 154.1, 153.8, 150.7, 139.3, 132.5, 130.0, 127.1, 1121.1, 120.5, 120.4 (q, *J* = 256.9 Hz), 120.3 (q, *J* = 158.5 Hz), 118.6, 116.3, 101.5; ^19^F NMR (470 MHz, CDCl_3_) δ −57.53 (s), −57.79 (s). HRMS (ESI-TOF) calculated for C_17_H_9_F_6_O_2_ [M+H] 391.0400; found 391.0391.

**6-((trifluoromethyl)thio)-3-(4-((trifluoromethyl)thio)phenyl)-1H-isochromen-1-one(2i)**. Yield: 75% (0.0617 g, 0.150 mmol), white solid (m.p. = 140–143°C). ^1^H NMR (500 MHz, CDCl_3_) δ 8.34 (d, *J* = 8.5 Hz, 1H), 7.92 (d, *J* = 8.5 Hz, 2H), 7.81 (s, 1H), 7.77–7.72 (m, 3H), 7.02 (s, 1H); ^13^C NMR (125 MHz, CDCl_3_) δ 160.8, 153.6, 137.8, 136.4, 134.3, 133.8, 133.0, 132.5, 130.9, 129.4 (q, *J* = 306.5 Hz), 129.1 (q, *J* = 307.0 Hz), 126.9, 126.2, 121.9, 102.2; ^19^F NMR (470 MHz, CDCl_3_) δ −41.09 (s), −42.14 (s). HRMS (ESI-TOF) calculated for C_17_H_8_F_6_O_2_S_2_Na [M+Na] 444.9762; found 444.9765.

**7-methyl-3-(m-tolyl)-1H-isochromen-1-one(2j)** (Nandi et al., 2013). Yield: 83% (0.0206 g, 0.166 mmol), white solid. ^1^H NMR (500 MHz, CDCl_3_) δ 8.09 (s, 1H), 7.69 (s, 1H), 7.64 (d, *J* = 8.0 Hz, 1H), 7.54–7.49 (m, 1H), 7.38 (d, *J* = 8.0 Hz, 1H), 7.34–7.30 (m, 1H), 7.21 (d, *J* = 7.5 Hz, 1H), 6.89 (s, 1H), 2.45 (s, 3H), 2.41 (s, 3H); ^13^C NMR (125 MHz, CDCl_3_) δ 162.5, 153.0, 138.5, 138.4, 136.1, 135.1, 132.0, 130.5, 129.3, 128.6, 125.8, 125.7, 122.2, 120.4, 101.6, 21.4, 21.3.

**8-methoxy-3-(2-methoxyphenyl)-1H-isochromen-1-one(2k)** (Neuhaus et al., 2018). Yield: 63% (0.0178 g, 0.126 mmol), white solid. ^1^H NMR (500 MHz, CDCl_3_) δ 7.97–7.94 (m, 1H), 7.59–7.55 (m, 1H), 7.38–7.29 (m, 1H), 7.26 (s, 1H), 7.07–6.93 (m, 3H), 6.89 (d, J = 8.0 Hz, 1H), 3.98 (s, 3H), 3.92 (s, 3H); ^13^C NMR (125 MHz, CDCl_3_) δ 161.4, 159.1, 157.2, 150.6, 140.9, 135.4, 130.6, 128.7, 120.6, 120.5, 118.3, 111.2, 109.6, 109.3, 106.9, 56.1, 55.5.

**3-(3,5-dimethylphenyl)-5,7-dimethyl-1H-isochromen-1-one(2l)** (Zhou et al., 2019). Yield: 58% (0.0273 g, 0.140 mmol), faint yellow solid. ^1^H NMR (500 MHz, CDCl_3_) δ 7.99 (s, 1H), 7.51 (s, 2H), 7.38 (s, 1H), 7.05 (s, 1H), 7.01 (s, 1H), 2.53 (s, 3H), 2.43 (s, 3H), 2.39 (s, 6H); ^13^C NMR (125 MHz, CDCl_3_) δ 163.0, 152.8, 138.4, 137.7, 137.1, 133.9, 133.4, 132.3, 131.5, 127.3, 123.0, 120.6, 98.4, 21.3, 21.3, 18.7.

**Methyl 3-(4-(methoxycarbonyl)phenyl)-1-oxo-1H-isochromene-6-carboxylate(2m)**. Yield: 48% (0.0162 g, 0.096 mmol), faint yellow solid (m.p. = 267.4–268.5°C). ^1^H NMR (400 MHz, CDCl_3_) δ 8.39 (d, *J* = 8.4 Hz, 1H), 8.23 (s, 1H), 8.15 (d, *J* = 8.4 Hz, 3H), 7.97 (d, *J* = 8.4 Hz, 2H), 7.12 (s, 1H), 4.01 (s, 3H), 3.96 (s, 3H); ^13^C NMR (125 MHz, CDCl_3_) δ 166.3, 165.7, 161.1, 153.3, 137.1, 136.0, 135.6, 131.6, 130.2, 130.1, 128.9, 127.8, 125.2, 123.7, 103.2, 52.8, 52.3. HRMS (ESI-TOF) calculated for C_19_H_15_O_6_ [M+H] 339.0863; found 391.0866.

**6-(trifluoromethyl)-3-(4-(trifluoromethyl)phenyl)-1H-isochromen-1-one(2n)** (Zhou et al., 2019). Yield: 76% (0.0272 g, 0.156 mmol), white solid. ^1^H NMR (500 MHz, CDCl_3_) δ 8.44 (d, J = 8.0 Hz, 1H), 8.00 (d, J = 8.0 Hz, 2H), 7.81 (s, 1H), 7.77–7.73 (m, 3H), 7.10 (s, 1H); ^13^C NMR (125 MHz, CDCl_3_) δ 160.6, 153.5, 137.3, 136.6 (q, *J* = 32.5 Hz), 134.7, 132.2 (q, *J* = 32.5 Hz), 130.8, 126.0 (q, *J* = 3.8 Hz), 125.7, 125.0 (q, *J* = 3.8 Hz), 123.7(q, *J* = 270.6 Hz), 123.4 (q, *J* = 3.8 Hz), 123.2 (q, *J* = 271.6 Hz), 123.1, 102.6; ^19^F NMR (470 MHz, CDCl_3_) δ −62.97 (s), −63.54 (s).

**6-nitro-3-(4-nitrophenyl)-1H-isochromen-1-one(2o)**. Yield: 56% (0.0175 g, 0.112 mmol), yellow solid (m.p. = 255.1–255.2°C). ^1^H NMR (500 MHz, DMSO) δ 8.56 (d, *J* = 2.0 Hz, 1H), 8.39–8.36 (m, 3H), 8.34–8.31 (m, 1H), 8.11 (d, *J* = 9.0 Hz, 2H), 7.95 (s, 1H); ^13^C NMR (125 MHz, DMSO) δ 159.6, 151.7, 151.3, 148.1, 137.7, 136.9, 131.1, 126.2, 124.6, 124.3, 123.0, 121.9, 104.7. HRMS (ESI-TOF) calculated for C_15_H_9_N_2_O_6_ [M+H] 313.0455; found 313.0443.

**6-fluoro-3-(4-fluorophenyl)-1H-isochromen-1-one(2p)** (Zhou et al., 2019). Yield: 71% (0.0183 g, 0.142 mmol), white solid. ^1^H NMR (500 MHz, CDCl_3_) δ 8.34–8.31 (m, 1H), 7.89–7.85 (m, 2H), 7.20–7.13 (m, 4H), 6.84 (s, 1H); ^13^C NMR (125 MHz, CDCl_3_) δ 166.81 (d, J = 255.0 Hz), 164.02 (d, J = 250.0 Hz), 161.1, 154.1, 140.15 (d, J = 11.3 Hz), 133.06 (d, J = 10.0 Hz), 127.89 (d, J = 3.8 Hz), 127.52 (d, J = 8.8 zHz), 116.88 (d, J = 2.5 Hz), 116.49 (d, J = 23.3 Hz), 116.08 (d, J = 22.5 Hz), 111.47 (d, J = 22.5 Hz), 101.0; ^19^F NMR (470 MHz, CDCl_3_) δ −101.66 (s), −109.51 (s).

**6-chloro-3-(4-chlorophenyl)-1H-isochromen-1-one(2q)** (Zhou et al., 2019). Yield: 72% (0.0210 g, 0.144 mmol), white solid. ^1^H NMR (500 MHz, CDCl_3_) δ 8.23 (d, *J* = 8.5 Hz, 1H), 7.80 (d, *J* = 9.0 Hz, 2H), 7.49–7.43 (m, 4H), 6.85 (s, 1H); ^13^C NMR (125 MHz, CDCl_3_) δ 161.2, 153.9, 141.7, 138.7, 136.5, 131.4, 130.1, 129.2, 128.8, 126.7, 125.5, 118.8, 101.0.

**6-bromo-3-(4-bromophenyl)-1H-isochromen-1-one(2r)**. Yield: 69% (0.0218 g, 0.138 mmol), white solid (m.p. = 245.8–246.7°C). ^1^H NMR (500 MHz, CDCl_3_) δ 8.15 (d, *J* = 8.0 Hz, 1H), 7.73 (d, *J* = 8.5 Hz, 2H), 7.67–7.66 (m, 1H), 7.64–7.58 (m, 3H), 6.86 (s, 1H); ^13^C NMR (125 MHz, CDCl_3_) δ 161.3, 153.9, 138.7, 132.2, 131.7, 131.4, 130.5, 130.4, 128.6, 126.8, 124.9, 119.2, 100.9. HRMS (ESI-TOF) calculated for C_15_H_9_Br_2_O_2_ [M+H] 378.8964; found 378.8967.

**6-iodo-3-(4-iodophenyl)-1H-isochromen-1-one(2s)**. Yield: 53% (0.0251 g, 0.106 mmol), white solid (m.p. = 272.2–273.4°C). ^1^H NMR (400 MHz, CDCl_3_) δ 7.97 (d, *J* = 8.8 Hz, 1H), 7.90 (s, 1H), 7.84–7.80 (m, 3H), 7.58 (d, *J* = 7.6 Hz, 2H), 6.84 (s, 1H); ^13^C NMR (125 MHz, CDCl_3_) δ 161.6, 153.9, 138.5, 138.2, 137.6, 134.9 131.2, 131.0, 126.8, 119.8, 103.3, 100.7, 96.7. HRMS (ESI-TOF) calculated for C_15_H_8_I_2_O_2_Na [M+Na] 496.8506; found 496.8513.

**8-fluoro-3-(2-fluorophenyl)-1H-isochromen-1-one(2t)** (Zhou et al., 2019). Yield: 62% (0.0160 g, 0.124 mmol), white solid. ^1^H NMR (500 MHz, CDCl_3_) δ 8.03–7.99 (m, 1H), 7.71–7.66 (m, 1H), 7.44–7.36 (m, 1H), 7.32–7.24 (m, 2H), 7.22–7.13 (m, 3H); ^13^C NMR (125 MHz, CDCl_3_) δ 162.91 (d, *J* = 266.3 Hz), 160.13 (d, *J* = 251.3 Hz), 157.4, 149.1, 140.0, 136.19 (d, *J* = 10.0 Hz), 131.47 (d, *J* = 8.8 Hz), 128.6, 124.64 (d, *J* = 3.8 Hz), 122.23 (d, *J* = 3.8 Hz), 119.71 (d, *J* = 10.0 Hz), 116.43 (d, *J* = 22.5 Hz), 115.63 (d, *J* = 21.3 Hz), 109.63 (d, *J* = 7.5 Hz), 106.48 (dd, *J* = 15.0, 2.9 Hz); ^19^F NMR (470 MHz, CDCl_3_) δ−107.01 (s),−111.73 (s).

**8-chloro-3-(2-chlorophenyl)-1H-isochromen-1-one(2u)** (Zhou et al., 2019). Yield: 70% (0.0204 g, 0.140 mmol), faint yellow solid. ^1^H NMR (500 MHz, CDCl_3_) δ 7.74–7.71 (m, 1H), 7.62–7.53 (m, 2H), 7.51–7.48 (m, 1H), 7.42–7.35 (m, 3H), 6.96 (s, 1H); ^13^C NMR (125 MHz, CDCl_3_) δ 158.7, 152.1, 140.0, 137.2, 134.6, 132.4, 131.4, 131.1, 130.9, 130.7, 130.6, 127.1, 125.2, 117.8, 107.4.

**8-bromo-3-(2-bromophenyl)-1H-isochromen-1-one(2v)**. Yield: 52% (0.0198 g, 0.104 mmol), white solid (m.p. = 122–123°C). ^1^H NMR (500 MHz, CDCl_3_) δ 7.82–7.78 (m, 1H), 7.69 (d, *J* = 8.0 Hz, 1H), 7.65–7.62 (m, 1H), 7.52–7.48 (m, 1H), 7.45–7.40 (m, 2H), 7.32–7.28 (m, 1H), 6.85 (s, 1H); ^13^C NMR (125 MHz, CDCl_3_) δ 159.0, 153.5, 140.0, 135.2, 134.6, 133.9, 133.3, 131.2, 130.9, 127.6, 125.9, 125.1, 121.8, 119.1, 107.3. HRMS (ESI-TOF) calculated for C_15_H_9_Br_2_O_2_ [M+H] 378.8964; found 378.8963.

**7-fluoro-3-(3-fluorophenyl)-1H-isochromen-1-one(2w)** (Zhou et al., 2019). Yield: 84% (0.0217 g, 0.168 mmol), white solid. ^1^H NMR (500 MHz, CDCl_3_) δ 8.10 (d, *J* = 7.0 Hz, 1H), 7.67 (d, *J* = 8.0 Hz, 1H), 7.62–7.56 (m, 1H), 7.60–7.57 (m, 3H), 7.19–7.10 (m, 2H); ^13^C NMR (125 MHz, CDCl_3_) δ 163.09 (d, J = 246.3 Hz), 160.52 (d, J = 3.8 Hz), 157.34 (d, J = 251.3Hz), 152.9, 133.87 (d, J = 8.8 Hz), 130.50 (d, J = 8.8 Hz), 128.81 (d, J = 7.5 Hz), 126.29 (d, J = 17.5 Hz), 125.40 (d, J = 3.8 Hz), 122.07 (d, J = 3.8 Hz), 120.98 (d, J = 2.5 Hz), 120.36 (d, J = 20.0 Hz), 117.18 (d, J = 21.3 Hz), 112.43 (d, J = 23.8 Hz), 95.07 (d, J = 5.0 Hz); ^19^F NMR (470 MHz, CDCl_3_) δ −111.76 (s), −120.69 (s).

**7-chloro-3-(3-chlorophenyl)-1H-isochromen-1-one(2x-*p*)**. Yield: 45% (0.0131 g, 0.090 mmol), white solid (m.p. = 190.8–195.2°C). ^1^H NMR (400 MHz, CDCl_3_) δ 8.29 (s, 1H), 7.86 (s, 1H), 7.78–7.64 (m, 2H), 7.47 (d, *J* = 8.4 Hz, 1H), 7.41 (d, *J* = 5.2 Hz, 2H), 6.95 (s, 1H); ^13^C NMR (125 MHz, CDCl_3_) δ 160.7, 152.5, 135.5, 135.4, 135.2, 134.4, 133.4, 130.2, 129.3, 127.6, 125.4, 123.3, 121.9, 101.9; HRMS (ESI-TOF) calculated for C_15_H_9_Cl_2_O_2_ [M+H] 290.9974; found 290.9970.

**5-chloro-3-(3-chlorophenyl)-1H-isochromen-1-one(2x-*o*)**. Yield: 36% (0.0104 g, 0.072 mmol), white solid (m.p. = 183°C). ^1^H NMR (500 MHz, CDCl_3_) δ 8.24 (d, J = 8.0 Hz, 1H), 7.91 (s, 1H), 7.83–7.75 (m, 2H), 7.49–7.38 (m, 3H), 7.32 (s, 1H); ^13^C NMR (125 MHz, CDCl_3_) δ 161.0, 153.1, 135.2, 133.5, 130.8, 130.4, 130.2, 128.7, 128.5, 125.6, 123.6, 122.2, 98.8. HRMS (ESI-TOF) calculated for C_15_H_9_Cl_2_O_2_ Na [M+H] 290.9974; found 290.9970.

**3-(naphthalen-2-yl)-1H-benzo[g]isochromen-1-one(2y)**. Yield: 70% (0.0226 g, 0.140 mmol), faint yellow solid (m.p. = 214.0–217.6 °C). ^1^H NMR (500 MHz, CDCl_3_) δ 8.93 (s, 1H), 8.46 (s, 1H), 7.96–7.79 (m, 7H), 7.67–7.61 (m, 1H), 7.55–7.49 (m, 3H), 7.18 (s, 1H); ^13^C NMR (125 MHz, CDCl_3_) δ 162.6, 152.0, 136.7, 133.8, 133.3, 132.5, 132.3, 132.0, 129.8, 129.5, 129.2, 128.8, 128.6, 127.73, 127.67, 127.1, 126.8, 126.7, 125.1, 124.4, 122.0, 119.1, 102.4. HRMS (ESI-TOF) calculated for C_23_H_14_O_2_Na [M+Na] 345.0886; found 345.0892.

## Data Availability Statement

The raw data supporting the conclusions of this article will be made available by the authors, without undue reservation.

## Author Contributions

YX designed the research. MZ and JZ carried out the experiments. MZ carried out DFT calculations and wrote the SI. All authors contributed to results discussion and manuscript preparation.

## Conflict of Interest

The authors declare that the research was conducted in the absence of any commercial or financial relationships that could be construed as a potential conflict of interest.

## Footnotes

^1^It should be noted that the enol **IM13** could be formed in the system as it is in equilibrium with **IM6** according to the relative low energies for related intermediates and TS in Figure 2, and in experiments the enol-keto tautomerism is very comment. However, further transformations from free **IM8** and **IM13** are less favorable as compared with the pathway in Figure 3. Details are given in the Supplementary Material.

## References

[B1] Ackermann L. (2011). Carboxylate-assisted transition-metal-catalyzed c-h bond functionalizations: mechanism and scope. Chem. Rev. 111, 1315–1345. 10.1021/cr100412j 21391562

[B2] Barday M. Janot C. Halcovitch N. R. Muir J. Aissa C. (2017). Cross-coupling of alpha-carbonyl sulfoxonium ylides with C-H bonds. Angew. Chem. Int. Ed. 56, 13117–13121. 10.1002/anie.201706804 28853234

[B3] Bayer A. Vaitla J. (2018). Sulfoxonium ylide derived metal carbenoids in organic synthesis. Synthesis 51, 612–628. 10.1055/s-0037-1610328

[B4] Becke A. D. (1993a). Density-functional thermochemistry. III. The role of exact exchange. J. Chem. Phys. 98, 5648–5652. 10.1063/1.464913

[B5] Becke A. D. (1993b). A new mixing of hartree–fock and local density-functional theories. J. Chem. Phys. 98, 1372–1377. 10.1063/1.464304

[B6] Cai L. Zhu X. Chen J. Lin A. Yao H. (2019). Rh(iii)-Catalyzed C–H activation/annulation of salicylaldehydes with sulfoxonium ylides for the synthesis of chromones. Org. Chem. Front. 6, 3688–3692. 10.1039/C9QO00830F

[B7] Chen G. Zhang X. Jia R. Li B. Fan X. (2018). Selective synthesis of benzo[a]carbazoles and indolo[2,1-a]-isoquinolines via Rh(III)-catalyzed C–H functionalizations of 2-arylindoles with sulfoxonium ylides. Adv. Synth. Catal. 360, 3781–3787. 10.1002/adsc.201800622

[B8] Chen J. Guo W. Xia Y. (2016). Computational revisit to the β-carbon elimination step in Rh(III)-catalyzed C-H activation/cycloaddition reactions of N-phenoxyacetamide and cyclopropenes. J. Org. Chem. 81, 2635–2638. 10.1021/acs.joc.6b00003 26889719

[B9] Chen P. Nan J. Hu Y. Ma Q. Ma Y. (2019). Ru(II)-catalyzed/NH2-assisted selective alkenyl C-H [5 + 1] annulation of alkenylanilines with sulfoxonium ylides to quinolines. Org. Lett. 21, 4812–4815. 10.1021/acs.orglett.9b01702 31192612

[B10] Chen X. Wang M. Zhang X. Fan X. (2019). Rh(III)-catalyzed cascade reactions of sulfoxonium ylides with α-diazocarbonyl compounds: an access to highly functionalized naphthalenones. Org. Lett. 21, 2541–2545. 10.1021/acs.orglett.9b00340 30958678

[B11] Cheng J. Wu X. Sun S. Yu J.-T. (2018). Recent applications of α-carbonyl sulfoxonium ylides in rhodium- and iridium-catalyzed C–H functionalizations. Synlett. 30, 21–29. 10.1055/s-0037-1610263

[B12] Clare D. Dobson B. C. Inglesby P. A. Aïssa C. (2019). Chemospecific cyclizations of a-carbonyl sulfoxonium ylides on aryls and heteroaryls. Angew. Chem. Int. Ed. 58, 16198–16202. 10.1002/anie.201910821 31507055 PMC6856693

[B13] Cui X.-F. Ban Z.-H. Tian W.-F. Hu F.-P. Zhou X.-Q. Ma H.-J.. (2019). Ruthenium-catalyzed synthesis of indole derivatives from N-aryl-2-aminopyridines and alpha-carbonyl sulfoxonium ylides. Org. Biomol. Chem. 17, 240–243. 10.1039/C8OB02818D 30534708

[B14] Davies H. M. Manning J. R. (2008). Catalytic C-H functionalization by metal carbenoid and nitrenoid insertion. Nature. 451, 417–424. 10.1038/nature06485 18216847 PMC3033428

[B15] Davies L. Macgregor S. A. McMullin C. L. (2017). Computational studies of carboxylate-assisted C-H activation and functionalization at group 8-10 transition metal centers. Chem. Rev. 117, 8649–8709. 10.1021/acs.chemrev.6b00839 28530807

[B16] Frisch M. J. Trucks G. W. Schlegel H. B. Scuseria G. E. Cheeseman J. R. Scalmani G. Gaussian 09, Revision, D. 01. Wallingford, CT: Gaussian, Inc (2013).

[B17] Fu Y. Wang Z. Zhang Q. Li Z. Liu H. Bi X. (2020). Ru(II)-catalyzed C6-selective C–H acylmethylation of pyridones using sulfoxonium ylides as carbene precursors. RSC Adv. 10, 6351–6355. 10.1039/C9RA10749E PMC9049633 35496007

[B18] Gao P. Guo W. Xue J. Zhao Y. Yuan Y. Xia Y.. (2015). Iridium(III)-catalyzed direct arylation of C-H bonds with diaryliodonium salts. J. Am. Chem. Soc. 137, 12231–12240. 10.1021/jacs.5b06758 26348796

[B19] Gulias M. Mascarenas J. L. (2016). Metal-catalyzed annulations through activation and cleavage of C-H bonds. Angew. Chem. Int. Ed. 55, 11000–11019. 10.1002/anie.201511567 27329931

[B20] Guo W. Xia Y. (2015). Mechanistic understanding of the divergent reactivity of cyclopropenes in Rh(III)-catalyzed C-H activation/cycloaddition reactions of N-phenoxyacetamide and N-pivaloxybenzamide. J. Org. Chem. 80, 8113–8121. 10.1021/acs.joc.5b01201 26218720

[B21] Guo W. Zhou T. Xia Y. (2015). Mechanistic understanding of the aryl-dependent ring formations in Rh(III)-catalyzed C-H activation/cycloaddition of benzamides and methylenecyclopropanes by DFT calculations. Organometallics 34, 3012–3020. 10.1021/acs.organomet.5b0031725982708

[B22] Hanchate V. Kumar A. Prabhu K. R. (2019). Synthesis of naphthols by Rh(III)-catalyzed domino C-H activation, annulation, and lactonization using sulfoxonium ylide as a traceless directing group. Org. Lett. 21, 8424–8428. 10.1021/acs.orglett.9b03182 31596098

[B23] Hoang G. L. Ellman J. A. (2018). Rhodium(III)-catalyzed C-H functionalization of C-alkenyl azoles with sulfoxonium ylides for the synthesis of bridgehead N-fused [5,6]-bicyclic heterocycles. Tetrahedron 74, 3318–3324. 10.1016/j.tet.2018.03.062 29988985 PMC6034689

[B24] Hoang G. L. Streit A. D. Ellman J. A. (2018). Three-component coupling of aldehydes, aminopyrazoles, and sulfoxonium ylides via rhodium(III)-catalyzed imidoyl C–H activation: synthesis of pyrazolo[1,5-a]pyrimidines. J. Org. Chem. 83, 15347–15360. 10.1021/acs.joc.8b02606 30525637 PMC6467769

[B25] Hou C. Jiang J. Liu Y. Zhao C. Ke Z. (2017). When bifunctional catalyst encounters dual MLC modes: DFT study on the mechanistic preference in Ru-PNNH pincer complex catalyzed dehydrogenative coupling reaction. ACS Catal. 7, 786–795. 10.1021/acscatal.6b02505

[B26] Hu P. Zhang Y. Liu B. Li X. (2018b). Facile construction of hydrogenated azepino[3,2,1-hi]Indoles by Rh(iii)-catalyzed C–H activation/[5 + 2] Annulation of N-cyanoacetylindolines with sulfoxonium ylides. Org. Chem. Front. 5, 3263–3266. 10.1039/C8QO00861B

[B27] Hu P. Zhang Y. Xu Y. Yang S. Liu B. Li X. (2018a). Construction of (Dihydro)naphtho[1,8-bc]pyrans via Rh(III)-catalyzed twofold C-H activation of benzoylacetonitriles. Org. Lett. 20, 2160–2163. 10.1021/acs.orglett.8b00420 29607647

[B28] Hu S. Du S. Yang Z. Ni L. Chen Z. (2019). Synthesis of multi-substituted dihydropyrazoles by copper-mediated [4+1] cycloaddition reaction of n-sulfonylhydrazones and sulfoxonium ylides. Adv. Synth. Catal. 361, 3124–3136. 10.1002/adsc.201900212

[B29] Huang Y. Lyu X. Song H. Wang Q. (2019). Sulfoxonium ylides as carbene precursors: rhodium(III)-catalyzed sequential C–H functionalization, selective enol oxygen-atom nucleophilic addition, and hydrolysis. Adv. Synth. Catal. 361, 5272–5276. 10.1002/adsc.201900861

[B30] Ji S. Yan K. Li B. Wang B. (2018). Cp^*^Co(III)-catalyzed C-H acylmethylation of arenes by employing sulfoxonium ylides as carbene precursors. Org. Lett. 20, 5981–5984. 10.1021/acs.orglett.8b02796 30207478

[B31] Jiang H. F. Zhang H. Xiong W. F. (2019). Iridium-catalyzed three-component coupling reaction of carbon dioxide, amines, and sulfoxonium ylides. Org. Lett. 21, 1125–1129. 10.1021/acs.orglett.9b00072 30714384

[B32] Jiang J. Liu H. Cao L. Zhao C. Liu Y. Ackermann L. (2019). Metallacyclopropene, or metallallylcarbenoid? RuCatalyzed annulation between benzoic acid and alkyne. ACS Catal. 9, 9387–9392. 10.1021/acscatal.9b02952

[B33] Karishma P. Agarwal D. S. Laha B. Mandal S. K. Sakhuja R. (2019). Ruthenium catalyzed C-H acylmethylation of N-arylphthalazine-1,4-diones with alpha-carbonyl sulfoxonium ylides: highway to diversely functionalized phthalazino-fused cinnolines. Chem. Asian J. 14, 4274–4288. 10.1002/asia.201901250 31613428

[B34] Kommagalla Y. Ando S. Chatani N. (2020). Rh(III)-catalyzed reaction of alpha-carbonyl sulfoxonium ylides and alkenes: synthesis of indanones via [4 + 1] cycloaddition. Org. Lett. 22, 1375–1379. 10.1021/acs.orglett.9b04664 32009402

[B35] Lai R. Wu X. Lv S. Zhang C. He M. Chen Y. (2019). Synthesis of indoles and quinazolines via additive-controlled selective C-H activation/annulation of N-arylamidines and sulfoxonium ylides. Chem. Commun. 55, 4039–4042. 10.1039/C9CC01146C 30865745

[B36] Lee C. Yang W. Parr R. G. (1988). Development of the colle-salvetti correlation-energy formula into a functional of the electron density. Phys. Rev. B. 37, 785–789. 10.1103/PhysRevB.37.785 9944570

[B37] Li A.-H. Dai L.-X. Aggarwal V. K. (1997). Asymmetric ylide reactions: epoxidation, cyclopropanation, aziridination, olefination, and rearrangement. Chem. Rev. 97, 2341–2372. 10.1021/cr960411r 11848902

[B38] Li C. Li M. Zhong W. Jin Y. Li J. Wu W.. (2019). Palladium-catalyzed oxidative allylation of sulfoxonium ylides: regioselective synthesis of conjugated dienones. Org. Lett. 21, 872–875. 10.1021/acs.orglett.8b03606 30726098

[B39] Li H. Wu C. Liu H. Wang J. (2019). Ruthenium(II)-catalyzed C-H acylmethylation between (hetero)arenes and alpha-Cl ketones/sulfoxonium ylides. J. Org. Chem. 84, 13262–13275. 10.1021/acs.joc.9b01013 31310527

[B40] Lian B. Zhang L. Fang D.-C. (2019). A computational study on ruthenium-catalyzed [4 + 1] annulation via C–H activation: the origin of selectivity and the role of the internal oxidizing group. Org. Chem. Front. 6, 2600–2606. 10.1039/C9QO00154A

[B41] Liang Y.-F. Yang L. Rogge T. Ackermann L. (2018). Ruthenium(IV) intermediates in C–H activation/annulation by weak O-coordination. Chem. Eur. J. 24, 16548–16552. 10.1002/chem.201804734 30251441

[B42] Ling B. Liu Y. Jiang Y.-Y. Liu P. Bi S. (2019). Mechanistic insights into the ruthenium-catalyzed [4 + 1] annulation of benzamides and propargyl alcohols by DFT studies. Organometallics. 38, 1877–1886. 10.1021/acs.organomet.8b00769

[B43] Liu C.-F. Liu M. Dong L. (2019). Iridium(III)-catalyzed tandem annulation synthesis of pyrazolo[1,2-alpha]cinnolines from pyrazolones and sulfoxonium ylides. J. Org. Chem. 84, 409–416. 10.1021/acs.joc.8b02582 30521336

[B44] Lou J. Wang Q. Zhou Y.-G. Yu Z. (2019). Rhodium(III)-catalyzed annulative coupling of sulfoxonium ylides and allenoates: an arene C-H activation/cyclopropanation cascade. Org. Lett. 21, 9217–9222. 10.1021/acs.orglett.9b03589 31689112

[B45] Luo Y. Guo L. Yu X. Ding H. Wang H. Wu Y. (2019). Cp^*^Ir^III^-catalyzed [3+2] annulations of N-aryl-2-aminopyrimidines with sulfoxonium ylides to access 2-alkyl indoles through C-H bond activation. Eur. J. Org. Chem. 2019, 3203–3207. 10.1002/ejoc.201900495

[B46] Lv N. Chen Z. Liu Z. Zhang Y. (2019). Redox-neutral rhodium(III)-catalyzed annulation of arylhydrazines with sulfoxonium ylides to synthesize 2-arylindoles. J. Org. Chem. 84, 13013–13021. 10.1021/acs.joc.9b01815 31436098

[B47] Nandi D. Ghosh D. Chen S.-J. Kuo B.-C. Wang N. M. Lee H. M. (2013). One-step synthesis of isocoumarins and 3-benzylidenephthalides via ligandless Pd-catalyzed oxidative coupling of benzoic acids and vinylarenes. J. Org. Chem. 78, 3445–3451. 10.1021/jo400174w 23506132

[B48] Nareddy P. Jordan F. Szostak M. (2017). Recent developments in ruthenium-catalyzed C–H arylation: array of mechanistic manifolds. ACS Catal. 7, 5721–5745. 10.1021/acscatal.7b01645

[B49] Neuhaus J. D. Pinto A. Maulide N. (2018). A catalytic cross-olefination of diazo compounds with sulfoxonium ylides. Angew. Chem. Int. Ed. 57, 16215–16218. 10.1002/anie.201809934 30264529 PMC6283242

[B50] Nie R. Lai R. Lv S. Xu Y. Guo L. Wang Q.. (2019). Water-mediated C-H activation of arenes with secure carbene precursors: the reaction and its application. Chem. Commun. 55, 11418–11421. 10.1039/C9CC05804D 31482875

[B51] Pan J.-L. Xie P. Chen C. Hao Y. Liu C. Bai H.-Y.. (2018). Rhodium(III)-catalyzed redox-neutral cascade [3 + 2] annulation of N-phenoxyacetamides with propiolates via C-H functionalization/isomerization/lactonization. Org. Lett. 20, 7131–7136. 10.1021/acs.orglett.8b03082 30407015

[B52] Phelps A. M. Schomaker J. M. Shekhar S. (2016). Ligand-controlled synthesis of azoles via Ir-catalyzed reactions of sulfoxonium ylides with 2-amino heterocycles. J. Org. Chem. 81, 4158–4169. 10.1021/acs.joc.6b00497 27104299

[B53] Sambiagio C. Schonbauer D. Blieck R. Dao-Huy T. Pototschnig G. Schaaf P.. (2018). A comprehensive overview of directing groups applied in metal-catalysed C-H functionalisation chemistry. Chem. Soc. Rev. 47, 6603–6743. 10.1039/C8CS00201K 30033454 PMC6113863

[B54] Shan C. Luo X. Qi X. Liu S. Li Y. Lan Y. (2016). Mechanism of ruthenium-catalyzed direct arylation of C–H bonds in aromatic amides: a computational study. Organometallics. 35, 1440–1445. 10.1021/acs.organomet.6b00064

[B55] Shan C. Zhong K. Qi X. Xu D. Qu L.-B. Bai R. (2018). Long distance unconjugated agostic-assisted 1,5-H shift in a Ru-mediated alder-ene type reaction: mechanism and stereoselectivity. Org. Chem. Front. 5, 3178–3185. 10.1039/C8QO00699G

[B56] Shen Z. Cui X. Wu Y. (2019). Rhodium(III)-catalyzed intermolecular cyclization of anilines with sulfoxonium ylides toward indoles. Chin. Chem. Lett. 30, 1374–1378. 10.1016/j.cclet.2019.01.033

[B57] Shi X. Wang R. Zeng X. Zhang Y. Hu H. Xie C. (2018). Ruthenium(II)-catalyzed oxidant-free coupling/cyclization of benzimidates and sulfoxonium ylides to form substituted isoquinolines. Adv. Synth. Catal. 360, 4049–4053. 10.1002/adsc.201800844

[B58] Shu S. Huang M. Jiang J. Qu L.-B. Liu Y. Ke Z. (2019). Catalyzed or non-catalyzed: chemoselectivity of Ru-catalyzed acceptorless dehydrogenative coupling of alcohols and amines via metal–ligand bond cooperation and (de)aromatization. Catal. Sci. Technol. 9, 2305–2314. 10.1039/C9CY00243J

[B59] Tian Y. Kong X.-Q. Niu J. Huang Y.-B. Wu Z.-H. Xu B. (2020). Rhodium-catalyzed regioselective C(sp2)–H bond activation reactions of N-(Hetero)aryl-7-azaindoles and cross-coupling with α-carbonyl sulfoxonium ylides. Tetrahedron Lett. 61, 10.1016/j.tetlet.2020.151627

[B60] Vaitla J. Bayer A. Hopmann K. H. (2017). Synthesis of indoles and pyrroles utilizing iridium carbenes generated from sulfoxonium ylides. Angew. Chem. Int. Ed. 56, 4277–4281. 10.1002/anie.201610520 28319303

[B61] Wang F. Yu S. Li X. (2016). Transition metal-catalysed couplings between arenes and strained or reactive rings: combination of C-H activation and ring scission. Chem. Soc. Rev. 45, 6462–6477. 10.1039/C6CS00371K 27711636

[B62] Wang P. Xu Y. Sun J. Li X. (2019). Rhodium(III)-catalyzed chemo-divergent couplings of sulfoxonium ylides with oxa/azabicyclic olefins. Org. Lett. 21, 8459–8463. 10.1021/acs.orglett.9b03226 31588755

[B63] Wang X. Xie P. Qiu R. Zhu L. Liu T. Li Y.. (2017). Nickel-catalysed direct alkylation of thiophenes via double C(sp^3^)-H/C(sp^2^)-H bond cleavage: the importance of KH_2_PO_4_. Chem. Commun. 53, 8316–8319. 10.1039/C7CC04252C 28686241

[B64] Wang Z. Xie P. Xia Y. (2018). Recent progress in Ru(II)-catalyzed C-H activations with oxidizing directing groups. Chin. Chem. Lett. 29, 47–53. 10.1016/j.cclet.2017.06.018

[B65] Wang Z. Xu H. (2019). Rhodium-catalyzed C–H activation/cyclization of enaminones with sulfoxonium ylides toward polysubstituted naphthalenes. Tetrahedron Lett. 60, 664–667. 10.1016/j.tetlet.2019.01.051

[B66] Wen S. Chen Y. Zhao Z. Ba D. Lv W. Cheng G. (2020). Ruthenium(II)-catalyzed construction of isocoumarins via dual C-H/C-C activation of sulfoxonium ylides. J. Org. Chem. 85, 1216–1223. 10.1021/acs.joc.9b02520 31808689

[B67] Wen S. Lv W. Ba D. Liu J. Cheng G. (2019). Ruthenium(ii)-catalyzed chemoselective deacylative annulation of 1,3-diones with sulfoxonium ylides via C-C bond activation. Chem. Sci. 10, 9104–9108. 10.1039/C9SC03245B 31827753 PMC6889838

[B68] Wu C. Zhou J. He G. Li H. Yang Q. Wang R. (2019). Ruthenium(ii)-catalyzed selective C–H bond activation of imidamides and coupling with sulfoxonium ylides: an efficient approach for the synthesis of highly functional 3-ketoindoles. Org. Chem. Front. 6, 1183–1188. 10.1039/C9QO00048H

[B69] Wu X. Xiao Y. Sun S. Yu J.-T. Cheng J. (2019). Rhodium-catalyzed reaction of sulfoxonium ylides and anthranils toward indoloindolones via a (4 + 1) annulation. Org. Lett. 21, 6653–6657. 10.1021/acs.orglett.9b02249 31424223

[B70] Wu Y. Pi C. Cui X. Wu Y. (2020). Rh(III)-catalyzed tandem acylmethylation/nitroso migration/cyclization of n-nitrosoanilines with sulfoxonium ylides in one pot: approach to 3-nitrosoindoles. Org. Lett. 22, 361–364. 10.1021/acs.orglett.9b03768 31895572

[B71] Xia Y. Qiu D. Wang J. (2017). Transition-metal-catalyzed cross-couplings through carbene migratory insertion. Chem. Rev. 117, 13810–13889. 10.1021/acs.chemrev.7b00382 29091413

[B72] Xiao Y. Xiong H. Sun S. Yu J. Cheng J. (2018). Rh(iii)-catalyzed dual C-H functionalization of 3-(1H-indol-3-yl)-3-oxopropanenitriles with sulfoxonium ylides or diazo compounds toward polysubstituted carbazoles. Org. Biomol. Chem. 16, 8715–8718. 10.1039/C8OB02145G 30411773

[B73] Xie H. Lan J. Gui J. Chen F. Jiang H. Zeng R. W. (2018). (II)-catalyzed coupling-cyclization of sulfoximines with alpha-carbonyl sulfoxonium ylides as an approach to 1,2-benzothiazines. Adv. Synth. Catal. 360, 3534–3543. 10.1002/adsc.201800753

[B74] Xie P. Guo W. Chen D. Xia Y. (2018a). Multiple pathways for C-H cleavage in cationic Cp^*^Rh(III)-catalyzed C-H activation without carboxylate assistance: a computational study. Catal. Sci. Technol. 8, 4005–4009. 10.1039/C8CY00870A

[B75] Xie P. Jia M. Xu X.-H. Chen F. Xia Y. (2018b). Mechanistic DFT study on rhodium(III)-catalyzed double C-H activation for oxidative annulations of 2-substituted imidazoles and alkynes. Asian J. Org. Chem. 7, 586–591. 10.1002/ajoc.201700625

[B76] Xie W. Chen X. Shi J. Li J. Liu R. (2019). Synthesis of 1-aminoindole derivatives via Rh(iii)-catalyzed annulation reactions of hydrazines with sulfoxonium ylides. Org. Chem. Front. 6, 2662–2666. 10.1039/C9QO00524B

[B77] Xu G. D. Huang K. L. Huang Z. Z. (2019). Rh(III)-catalyzed aldehydic C–H functionalization reaction between salicylaldehydes and sulfoxonium ylides Adv. Synth. Catal. 361, 3318–3323. 10.1002/adsc.201900276

[B78] Xu L. Zhu Q. Huang G. Cheng B. Xia Y. (2012). Computational elucidation of the internal oxidant-controlled reaction pathways in Rh(III)-catalyzed aromatic C-H functionalization. J. Org. Chem. 77, 3017–3024. 10.1021/jo202431q 22204386

[B79] Xu Y. Yang X. Zhou X. Kong L. Li X. (2017b). Rhodium(III)-catalyzed synthesis of naphthols via C-H activation of sulfoxonium ylides. Org. Lett. 19, 4307–4310. 10.1021/acs.orglett.7b01974 28783365

[B80] Xu Y. Zheng G. Yang X. Li X. (2018). Rhodium(iii)-catalyzed chemodivergent annulations between N-methoxybenzamides and sulfoxonium ylides via C-H activation. Chem. Commun. 54, 670–673. 10.1039/C7CC07753J 29303167

[B81] Xu Y. Zhou X. Zheng G. Li X. (2017a). Sulfoxonium ylides as a carbene precursor in Rh(III)-catalyzed C-H acylmethylation of arenes. Org. Lett. 19, 5256–5259. 10.1021/acs.orglett.7b02531 28901774

[B82] You C. Pi C. Wu Y. Cui X. (2018). Rh(III)-catalyzed selective C8–H acylmethylation of quinoline N-oxides. Adv. Synth. Catal. 360, 4068–4072. 10.1002/adsc.201800659

[B83] Yu J. Wen S. Ba D. Lv W. Chen Y. Cheng G. (2019). Rhodium(III)-catalyzed regioselective C3–H acylmethylation of [2,2′-bipyridine]-6-carboxamides with sulfoxonium ylides. Org. Lett. 21, 6366–6369. 10.1021/acs.orglett.9b02253 31361496

[B84] Yu J.-L. Zhang S.-Q. Hong X. (2017). mechanisms and origins of chemo- and regioselectivities of ru(II)-catalyzed decarboxylative C-H alkenylation of aryl carboxylic acids with alkynes: a computational study. J. Am. Chem. Soc. 139, 7224–7243. 10.1021/jacs.7b00714 28498678

[B85] Yu Y. Wu Q. Liu D. Yu L. Tan Z. Zhu G. (2019). Synthesis of 1-naphthols via Cp^*^Co(III)-catalyzed C–H activation and cyclization of sulfoxonium ylides with alkynes. Org. Chem. Front. 6, 3868–3873. 10.1039/C9QO00994A

[B86] Zhang J. Wang X. Chen D. Kang Y. Ma Y. Szostak M. (2020). Synthesis of C6-substituted isoquinolino[1,2-b]quinazolines via Rh(III)-catalyzed C-H annulation with sulfoxonium ylides. J. Org. Chem. 85, 3192–3201. 10.1021/acs.joc.9b03065 31944108

[B87] Zhang L. Chen J. Jin L. X. Zheng Jiang X. Yu C. (2019). Synthesis of 2-substituted indoles by iridium(III)-catalyzed C–H functionalization of N-phenylpyridin-2-amines. Tetrahedron Lett. 60, 1053–1056. 10.1016/j.tetlet.2019.03.027

[B88] Zhao Y. Truhlar D. G. (2008a). The M06 suite of density functionals for main group thermochemistry, thermochemical kinetics, noncovalent interactions, excited states, and transition elements: two new functionals and systematic testing of four m06-class functionals and 12 other functionals. Theor. Chem. Acc. 120, 215–241. 10.1007/s00214-007-0310-x

[B89] Zhao Y. Truhlar D. G. (2008b). Density functionals with broad applicability in chemistry. Acc. Chem. Res. 41, 157–167. 10.1021/ar700111a 18186612

[B90] Zhou C. Fang F. Cheng Y. Li Y. Liu H. Zhou Y. (2018). Rhodium(III)-catalyzed C-H activation of benzoylacetonitriles and cyclization with sulfoxonium ylides to naphthols. Adv. Synth. Catal. 360, 2546–2551. 10.1002/adsc.201800362

[B91] Zhou M.-D. Peng Z. Wang H. Wang Z.-H. Hao D.-J. Li L. (2019). Ruthenium(II)-catalyzed homocoupling of weakly coordinating sulfoxonium ylides via C–H activation/annulations: synthesis of functionalized isocoumarins. Adv. Syn. Catal. 361, 5191–5197. 10.1002/adsc.201900764

[B92] Zhou P. Yang W. T. Rahman A. U. Li G. Jiang B. (2020). Rh(III)-Catalyzed [3 + 3] annulation reaction of cyclopropenones and sulfoxonium ylides toward trisubstituted 2-pyrones. J. Org. Chem. 85, 360–366. 10.1021/acs.joc.9b02253 31645097

[B93] Zhou T. Guo W. Xia Y. (2015). Rh^V^-nitrenoid as a key intermediate in Rh^III^-catalyzed heterocyclization by C-H activation: a computational perspective on the cycloaddition of benzamide and diazo compounds. Chem. Eur. J. 21, 9209–9218. 10.1002/chem.201500558 25982708

[B94] Zhu S. Shi K. Zhu H. Jia Z.-K. Xia X.-F. Wang D.. (2020). Copper-catalyzed annulation or homocoupling of sulfoxonium ylides: synthesis of 2,3-diaroylquinolines or α,α,β-tricarbonyl sulfoxonium ylides. Org. Lett. 22, 1504–1509. 10.1021/acs.orglett.0c00085 32043889

