# Supplementary material for: Ruthenium(II)-Catalyzed Homocoupling of α-Carbonyl Sulfoxonium Ylides Under Mild Conditions: Methodology Development and Mechanistic DFT Study
Source: Front Chem. 2020 Sep 16;8:648. doi: 10.3389/fchem.2020.00648 (PMC7525066; doi:10.3389/fchem.2020.00648)
Supplement: Supplementary file 1 [file Data_Sheet_1.PDF]

Supporting Information

Ruthenium(II)-Catalyzed Homocoupling of α-Carbonyl Sulfoxonium Ylides under Mild Conditions: Methodology Development and Mechanistic DFT Study

Maosheng Zhang,[a] Jinrong Zhang,[a] Zhenfang Teng,[b] Jianhui Chen,[a] and Yuanzhi Xia[a]*

[a]College of Chemistry and Materials Engineering, Wenzhou University, Wenzhou 325035, China

[b]Information Technology Center, Wenzhou University, Wenzhou 325035, China

xyz@wzu.edu.cn

Table of Content:

I. Gram-scale Reaction(P2)

II. Control Experiments (P3)

III. DFT calculation (P8)

IV. NMR Spectra (P50)

I. Gram-scale Reaction

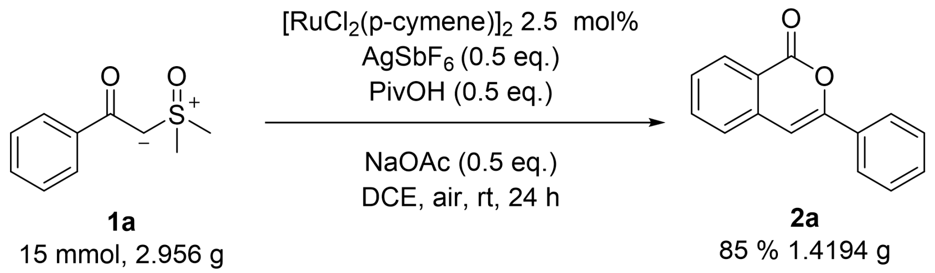

1a 15 mmol, 2.956 g

2a 85 % 1.4194 g

To a 250 mL flame-dried round-bottom flask, sulfur ylide, 2.9591 g (15.0 mmol) of **1a**, 0.2296 g (0.375 mmol) of $[RuCl_2(p\text{-cymene})]_2$, 0.6305 g (7.5 mmol) of NaOAc, 0.7667 g (7.5 mmol) of PivOH, 2.5772 g (7.5 mmol) of $AgSbF_6$ and DCE (60 mL) were added in sequence. The mixture was stirred at room temperature for 24 hours. The mixture is pumped through a suction funnel and silica gel and washed with mixed liquor of EA and PE. The filtrate was concentrated under reduced pressure and purified by flash chromatography on silica gel to give 1.6440 g (12.8 mmol, 85% yield) of **2a.**

II. Control Experiments

Deuterium Labeling Reactions

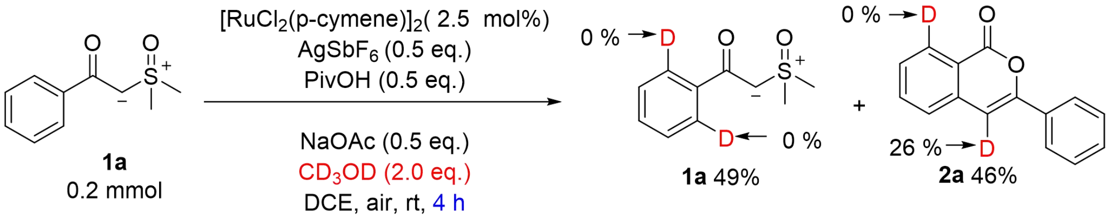

To a 10 mL flame-dried reaction tube, sulfur ylide **1a** (0.0395 g, 0.20 mmol), [RuCl$_2$(p-cymene)]$_2$ (5 mol%. 0.0061 g, 0.10 mmol), NaOAc (0.5 equiv 0.0084 g, 0.10 mmol), PivOH (0.5 equiv 0.0102 g, 0.10 mmol), AgSbF$_6$ (0.5 equiv 0.0346 g, 0.10 mmol), DCE (1 mL) and CD$_3$OD (0.4 mmol) were added in sequence. The mixture was stirred at room temperature for 4 hours. The yield (46%) of **2a** and the yield (49%) of **1a** was determined by ^{1}H NMR analysis using mesitylene as an internal standard. The mixture is pumped through a suction funnel and silica gel and washed with mixed liquor of EA and PE. The filtrate was concentrated under reduced pressure and purified by flash chromatography on silica gel to give pure homo-coupling product.

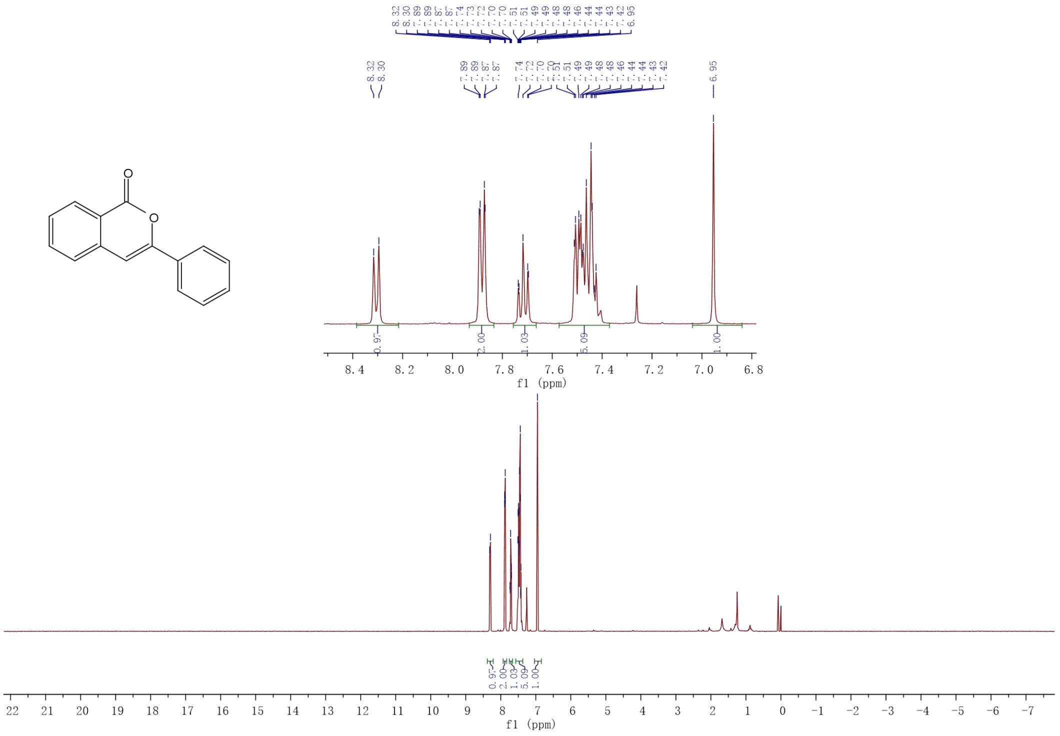

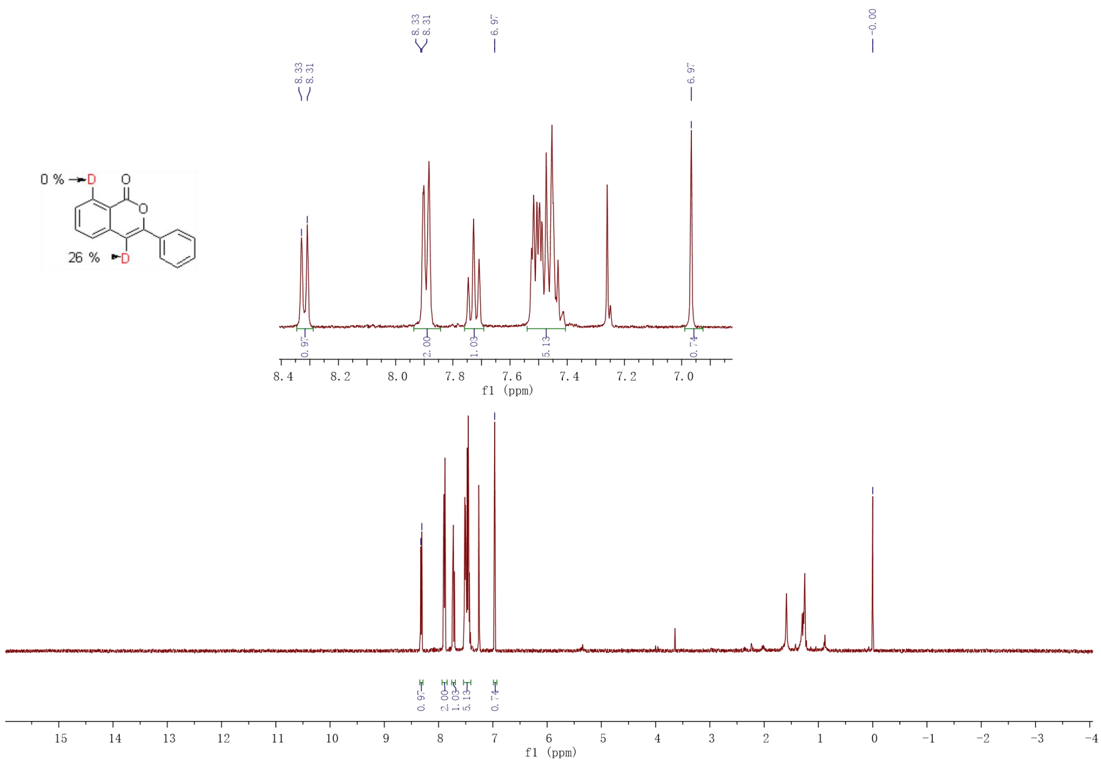

Cross coupling experiments

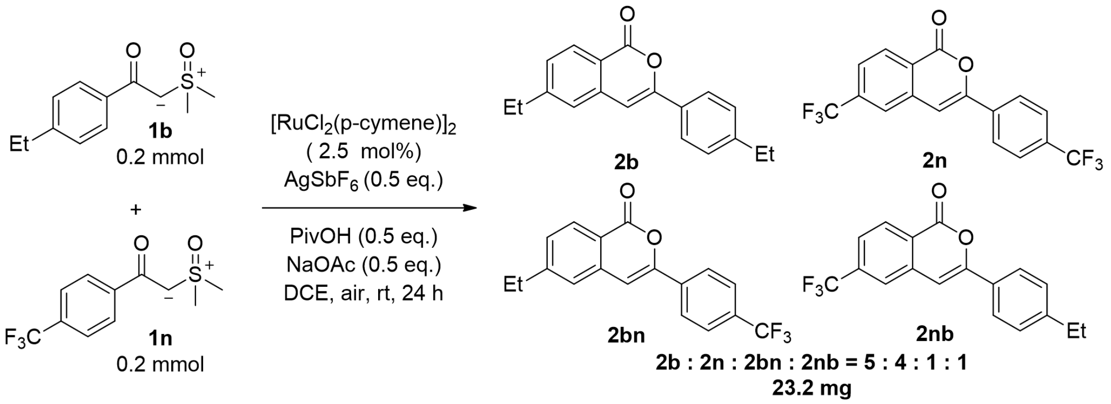

To a 10 mL flame-dried reaction tube, sulfur ylide **1b** (0.0395 g, 0.20 mmol), **1n** (0.0528 g, 0.20 mmol), $[RuCl_2(p\text{-cymene})]_2$ (5 mol%. 0.0061 g, 0.10 mmol), NaOAc (0.5 equiv 0.0084 g, 0.10 mmol), PivOH (0.5 equiv 0.0102 g, 0.10 mmol), $AgSbF_6$ (0.5 equiv 0.0346 g, 0.10 mmol) and DCE (1 mL) were added in sequence. The mixture was stirred at room temperature for 24 hours. The mixture is pumped through a suction funnel and silica gel and washed with mixed liquor of EA and PE. The filtrate was concentrated under reduced pressure and purified by flash chromatography on silica gel to give mixture homo-coupling product **2b**, **2n** and cross-coupling product **2bn**, **2nb**. The ratio of **2b, 2n, 2bn, 2nb** was determined by ^{1}H NMR analysis.

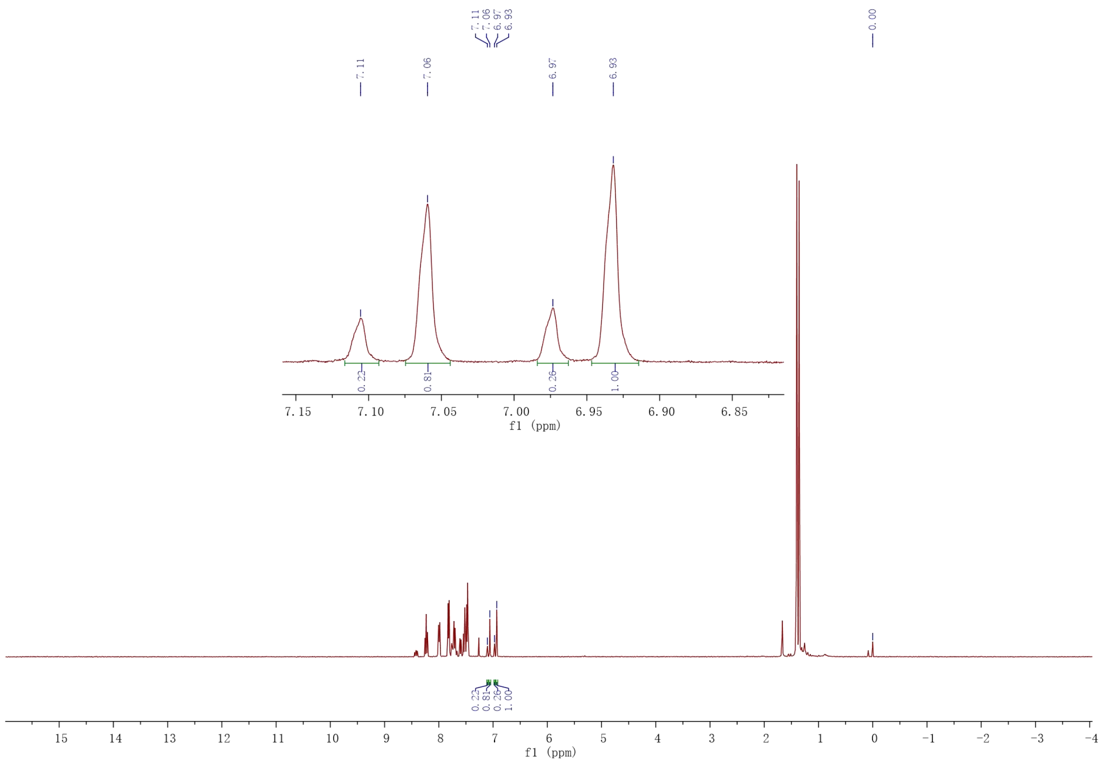

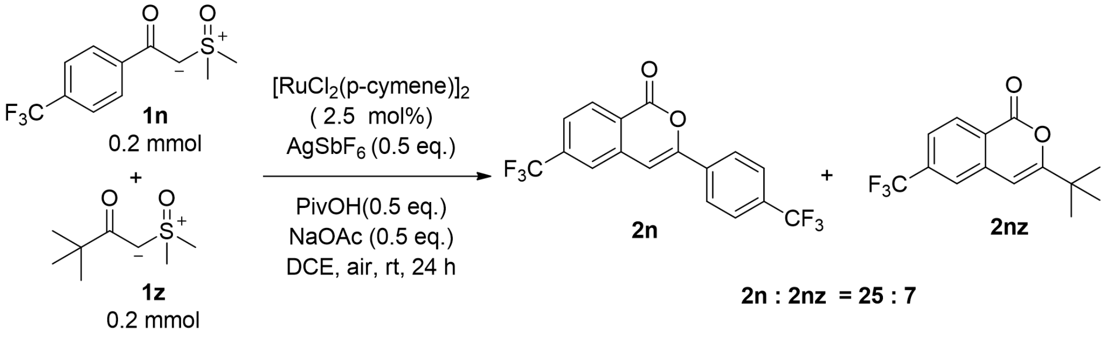

To a 10 mL flame-dried reaction tube, sulfur ylide **1n** (0.0528 g, 0.20 mmol), **1z** (0.0353 g, 0.20 mmol), $[RuCl_2(p\text{-cymene})]_2$ (5 mol%. 0.0061 g, 0.10 mmol), NaOAc (0.5 equiv 0.0084 g, 0.10 mmol), PivOH (0.5 equiv 0.0102 g, 0.10 mmol), $AgSbF_6$ (0.5 equiv 0.0346 g, 0.10 mmol) and DCE (1 mL) were added in sequence. The mixture was stirred at room temperature for 24 hours. The mixture is pumped through a suction funnel and silica gel and washed with mixed liquor of EA and PE. The filtrate was concentrated under reduced pressure to give mixture homo-coupling product **2n** and cross-coupling product **2nz**. The ratio of **2n**, **2nz** was determined by ^{1}H NMR analysis.

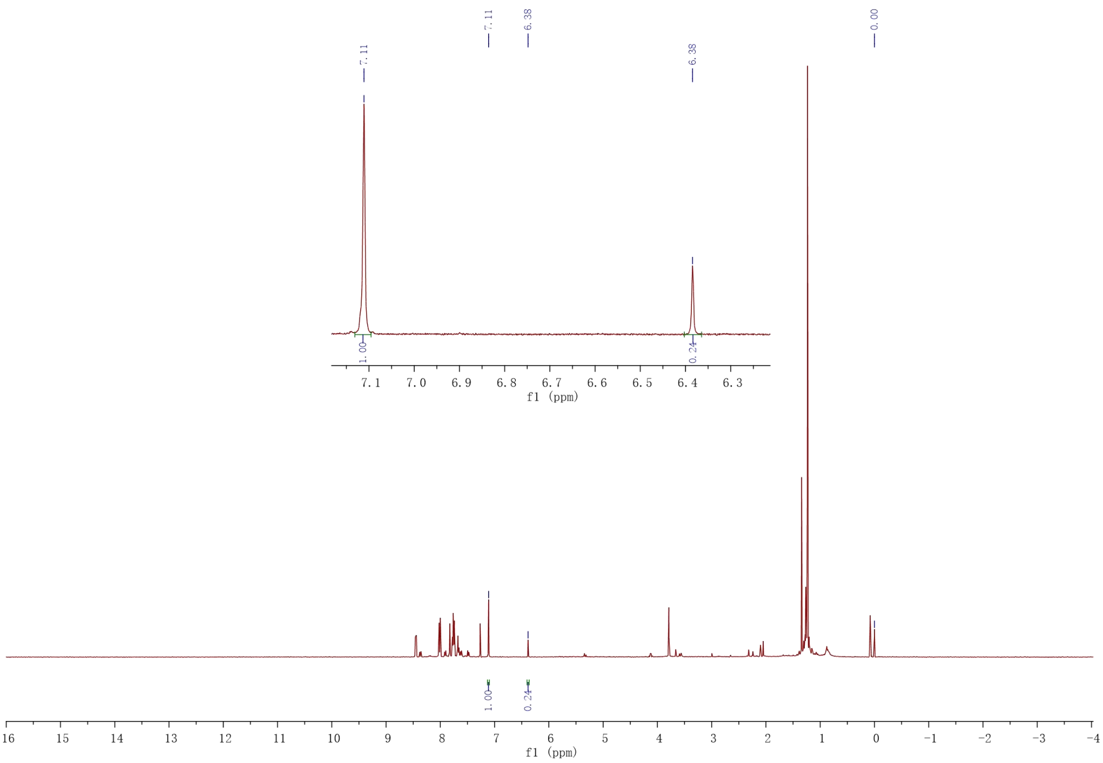

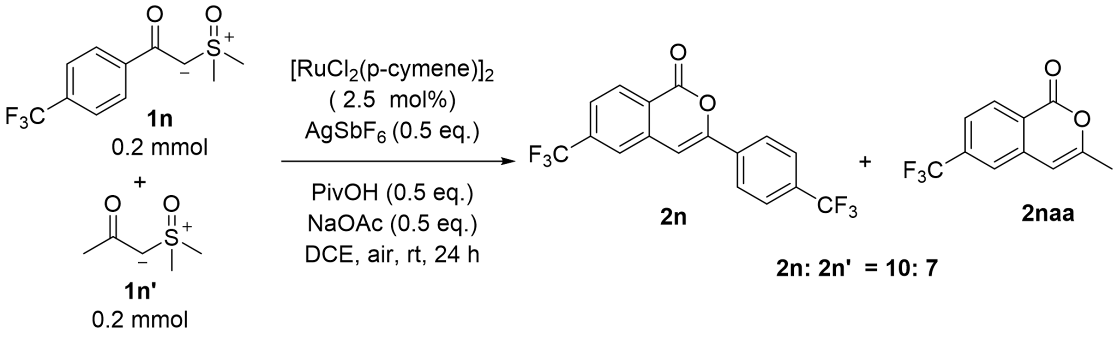

To a 10 mL flame-dried reaction tube, sulfur ylide **1n** (0.0528 g, 0.20 mmol), **1n'** (0.0267 g, 0.20 mmol), $[RuCl_2(p\text{-cymene})]_2$ (5 mol%. 0.0061 g, 0.10 mmol), NaOAc (0.5 equiv 0.0084 g, 0.10 mmol), PivOH (0.5 equiv 0.0102 g, 0.10 mmol), $AgSbF_6$ (0.5 equiv 0.0346 g, 0.10 mmol) and DCE (1 mL) were added in sequence. The mixture was stirred at room temperature for 24 hours. The mixture is pumped through a suction funnel and silica gel and washed with mixed liquor of EA and PE. The filtrate was concentrated under reduced pressure to give mixture homo-coupling product **2n** and cross-coupling product **2n'**. The ratio of **2n** and **2n'** was determined by ^{1}H NMR analysis.

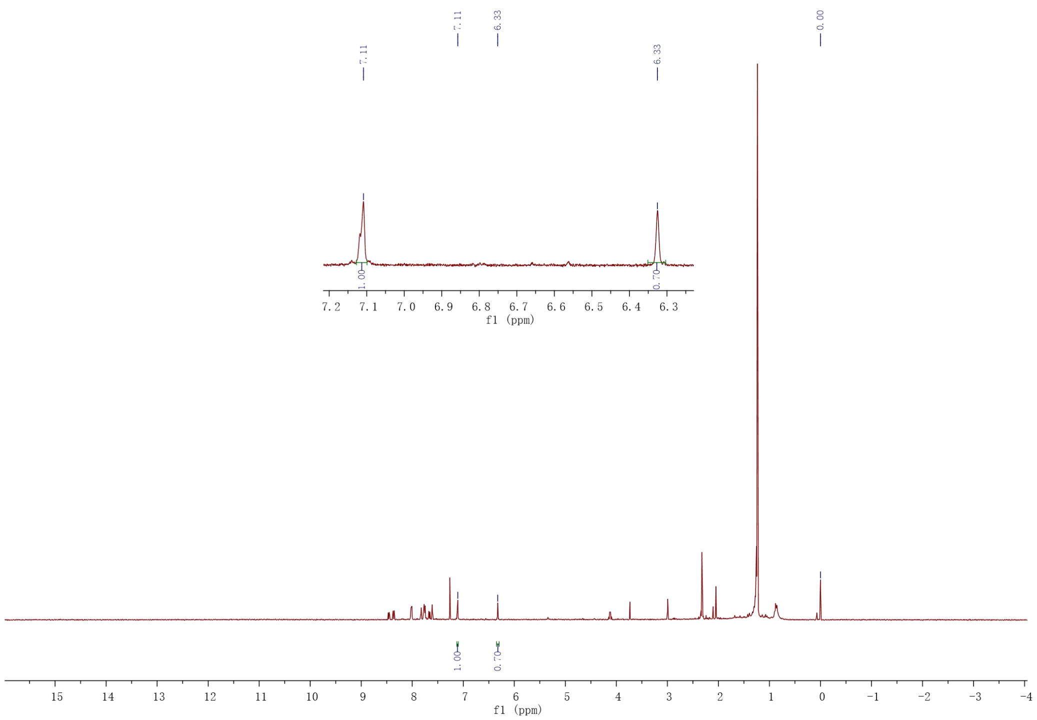
7.11
6.33
7.11
6.33
0.00
1.00
0.70
7.2 7.1 7.0 6.9 6.8 6.7 6.6 6.5 6.4 6.3
f1 (ppm)
15 14 13 12 11 10 9 8 7 6 5 4 3 2 1 0 -1 -2 -3 -4
f1 (ppm)

III. DFT calculation

1. Computational Details

All DFT calculations were carried out with the Gaussian 09 suite of computational programs.[13] The geometries of all stationary points were optimized using the B3LYP hybrid functional at the basis set level of 6-31G(d) for all atom except for Ru, which was described by the relativistic effective core potential basis set of Lanl2dz. Frequencies were analytically computed at the same level of theory to obtain the free energies and to confirm whether the structures are minima (no imaginary frequency) or transition states (only one imaginary frequency). The solvent effect of toluene was evaluated by using the SMD polarizable continuum model by carrying out single point calculations at the M06/6-311+G(d,P) (SDD fur Ru) level. All transition state structures were confirmed to connect the proposed [1]reactants and products by intrinsic reaction coordinate (IRC) calculations. All the energies given in the text are relative free energies corrected with solvation effects.

[1]Gaussian 09, Revision A.01, M. J. Frisch, G. W. Trucks, H. B. Schlegel, G. E. Scuseria, M. A. Robb, J. R. Cheeseman, G. Scalmani, V. Barone, B. Mennucci, G. A. Petersson, H. Nakatsuji, M. Caricato, X. Li, H. P. Hratchian, A. F. Izmaylov, J. Bloino, G. Zheng, J. L. Sonnenberg, M. Hada, M. Ehara, K. Toyota, R. Fukuda, J. Hasegawa, M. Ishida, T. Nakajima, Y. Honda, O. Kitao, H. Nakai, T. Vreven, J. A. Montgomery, Jr., J. E. Peralta, F. Ogliaro, M. Bearpark, J. J. Heyd, E. Brothers, K. N. Kudin, V. N. Staroverov, R. Kobayashi, J. Normand, K. Raghavachari, A. Rendell, J. C. Burant, S. S. Iyengar, J. Tomasi, M. Cossi, N. Rega, J. M. Millam, M. Klene, J. E. Knox, J. B. Cross, V. Bakken, C. Adamo, J. Jaramillo, R. Gomperts, R. E. Stratmann, O. Yazyev, A. J. Austin, R. Cammi, C. Pomelli, J. W. Ochterski, R. L. Martin, K. Morokuma, V. G. Zakrzewski, G. A. Voth, P. Salvador, J. J. Dannenberg, S. Dapprich, A. D. Daniels, Ö. Farkas, J. B. Foresman, J. V. Ortiz, J. Cioslowski, D. J. Fox, Gaussian, Inc., Wallingford CT, 2009.

2. Energies for Possible Reactions from IM8, IM10, and IM13

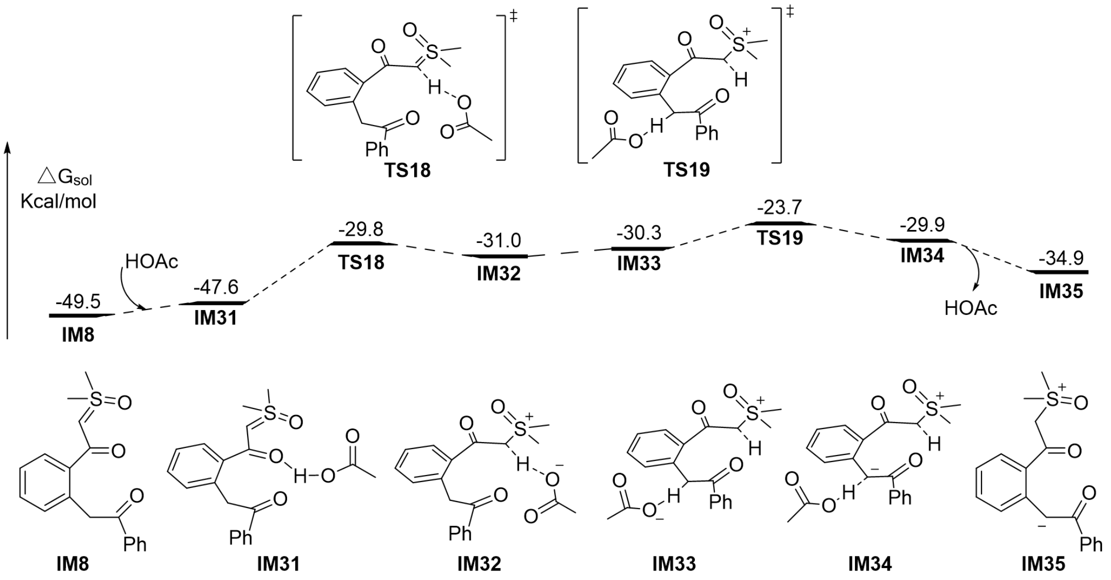

Figure S1.

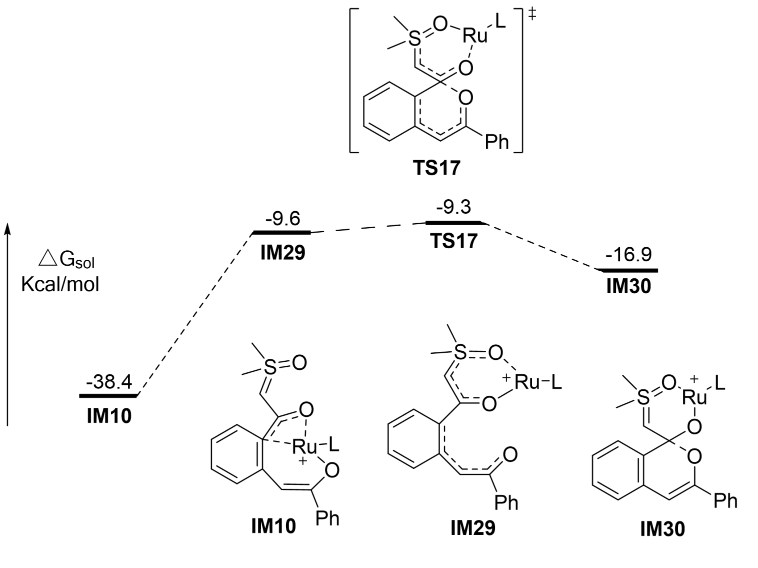

Figure S2.

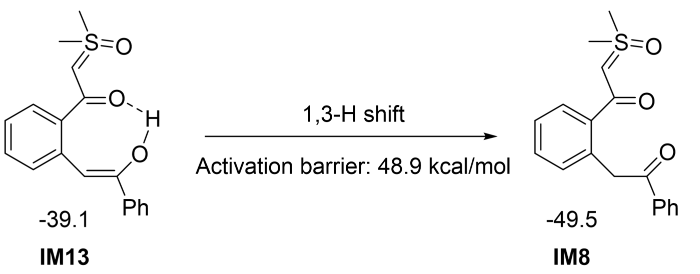

Figure S3.

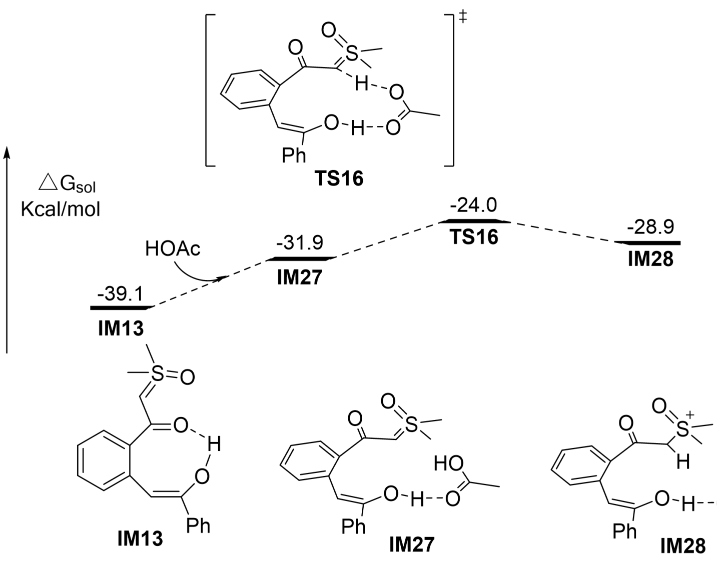

Figure S4.

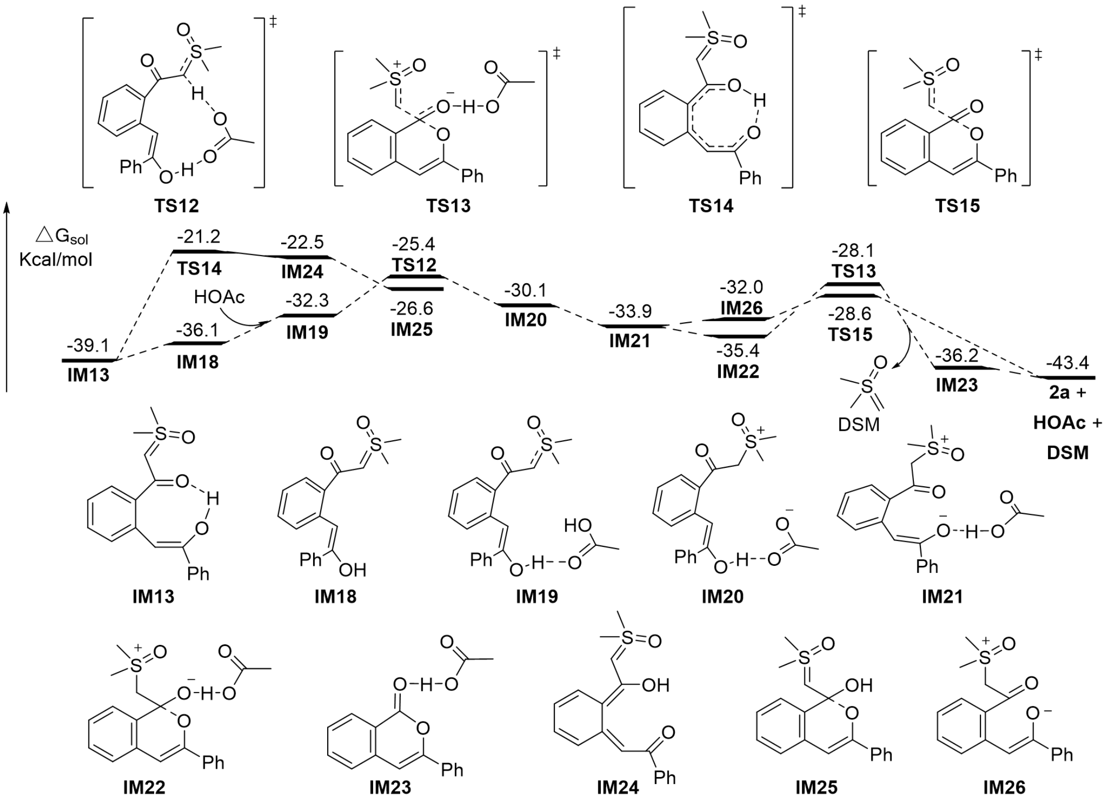

Figure S5.

3. Calculated Energy Values

Table S1. Energies (in Hartree) calculated by (SMD)M06/6-311+G(d,p)/SDD //B3LYP/6-31G(d)/Lanl2dz method.

Species	G_{298}[a]	E[b]	G_{Sol}[c]	# of imaginary frequencies
**1a**	-936.667965	-936.8270419	-936.606424477	0
**AcOH**	-229.046884	-229.081788	-229.039289868	0
**2a**	-727.917027	-728.0860438	-727.767809347	0
**DMSO**	-553.135457	-553.186941	-553.134600481	0
**Ylide2**	-592.334563	-592.4105397	-592.338269455	0
**Cat1.**	-711.447636	-711.6680688	-712.48394573	0
**IM1**	-1648.141251	-1648.5460337	-1649.13528101	0
**TS1**	-1648.108012	-1648.5091541	-1649.09913677	1
**IM2**	-1648.129271	-1648.5338761	-1649.12261883	0
**IM3**	-1419.072473	-1419.4206913	-1420.06127912	0
**IM4**	-2355.729662	-2356.2643074	-2356.70825032	0
**TS2**	-2355.725012	-2356.2563522	-2356.6880946	1
**IM5**	-1802.625743	-1803.0793209	-1803.55299295	0
**TS3**	-1802.593735	-1803.0516495	-1803.52815142	1
**IM6**	-1802.66026	-1803.1184063	-1803.60877415	0
**TS4**	-2031.668988	-2032.179583	-2032.61824686	1
**IM7**	-2149.565117	-2150.1598381	-2150.53910493	0
**IM8**	-1320.253908	-1320.52031	-1320.13736745	0
**IM9**	-1802.674498	-1803.133771	-1803.60776936	0
**IM10**	-1802.65105	-1803.1110547	-1803.59185218	0
**IM11**	-2031.700772	-2032.2170472	-2032.65451894	0
**TS5**	-2031.699778	-2032.2131385	-2032.649909	1
**IM12**	-2031.698378	-2032.2132087	-2032.65000343	0
**IM13**	-1320.236754	-1320.504779	-1320.1222598	0
**TS6**	-1549.255465	-1549.5737957	-1549.14414063	1
**TS7**	-2031.671485	-2032.1826117	-2032.62134496	1
**TS8**	-2031.665247	-2032.18228	-2032.635418	1
**IM14**	-2031.680857	-2032.20067	-2032.643189	0
**TS9**	-2031.678196	-2032.196625	-2032.641428	1
**IM15**	-2031.693476	-2032.211924	-2032.658892	0
**TS10**	-2031.685058	-2032.205899	-2032.650352	1
**IM16**	-2031.707516	-2032.225549	-2032.660103	0
**TS11**	-2031.693086	-2032.204897	-2032.632252	1

**IM17**	-1439.379125	-1439.792308	-1440.279326	0
**IM18**	-1320.231577	-1320.499929	-1320.117753	0
**IM19**	-1549.276023	-1549.597504	-1549.169312	0
**TS12**	-1549.265375	-1549.584146	-1549.155653	1
**IM20**	-1549.267651	-1549.589639	-1549.166284	0
**IM21**	-1549.277717	-1549.601616	-1549.174289	0
**IM22**	-1549.282901	-1549.60863	-1549.178513	0
**TS13**	-1549.275903	-1549.598635	-1549.16381	1
**IM23**	-1549.29266	-1549.610097	-1549.171401	0
**TS14**	-1320.199364	-1320.4685041	-1320.09489834	1
**IM24**	-1320.201647	-1320.4708515	-1320.09704047	0
**IM25**	-1320.209835	-1320.4797959	-1320.1042364	0
**IM26**	-1320.217544	-1320.486981	-1320.11238789	0
**TS15**	-1320.219894	-1320.4897909	-1320.1074637	1
**IM27**	-1549.272182	-1549.5930001	-1549.16805079	0
**TS16**	-1549.261981	-1549.581057	-1549.15367936	1
**IM28**	-1549.262448	-1549.5873347	-1549.16731767	0
**IM29**	-1802.598658	-1803.0532473	-1803.54062259	0
**TS17**	-1802.594004	-1803.0492817	-1803.54082853	1
**IM30**	-1802.613994	-1803.0709791	-1803.55457865	0
**IM31**	-1549.304784	-1549.625353	-1549.192811	0
**TS18**	-1549.277351	-1549.596534	-1549.16295	1
**IM32**	-1549.269709	-1549.591514	-1549.167587	0
**IM33**	-1549.27121	-1549.595279	-1549.168655	0
**TS19**	-1549.26668	-1549.587933	-1549.155307	1
**IM34**	-1549.273685	-1549.598003	-1549.168247	0
**IM35**	-1320.225789	-1320.49591	-1320.117692	0

[a] Sum of electronic and thermal free energies

[b] Electronic energies

[c] Single point energies in solution

4. Cartesian Coordinates for All Species

1a

C	-0.61150900	-0.95833800	-0.26477100
C	0.59206800	-0.27661200	-0.08831000
C	0.59827700	1.09954300	0.18169800
C	-0.62535100	1.77908900	0.26310600
C	-1.82835800	1.09821800	0.09115300
C	-1.82516000	-0.27369600	-0.17215400
H	-0.60151900	-2.02392800	-0.47858100
H	1.52645800	-0.82179800	-0.18111800
H	-0.60270900	2.84536700	0.46158100
H	-2.76997000	1.63682500	0.16107900
H	-2.76283300	-0.80631400	-0.30849200
C	1.85490900	1.90501600	0.38297600
O	1.77451800	3.15883500	0.42793500
C	3.09122000	1.22341100	0.52250100
H	3.27487700	0.16054600	0.58067000
S	4.48987600	2.14006700	0.75539000
O	5.72392200	1.33153400	0.89527400
C	4.66200700	3.33630500	-0.59438500
H	3.70151500	3.84582700	-0.69386400
H	5.47677400	4.02394000	-0.35559000
H	4.89791300	2.75019600	-1.48475900
C	4.29380600	3.23496000	2.18824100
H	5.13830300	3.92723700	2.22592100
H	3.33782300	3.74832400	2.06659400
H	4.28857900	2.58278500	3.06391700

AcOH

C	-4.50930800	0.75088700	0.32507300
H	-4.72539100	-0.29939400	0.54700000
H	-3.67553500	0.76926400	-0.38444800
H	-5.38534700	1.22597700	-0.11668800
C	-4.12634100	1.47672300	1.59037900
O	-4.69199300	2.43652200	2.06280000
O	-3.03292700	0.91618400	2.17031200
H	-2.85773500	1.44174800	2.97352300

PivOH

C	-4.50842500	0.73822000	0.31188700
C	-4.09171200	1.45445400	1.59737700
O	-4.63804800	2.41779400	2.08935200
O	-2.99688200	0.88671200	2.17225700
H	-2.81834300	1.41246200	2.97452300
C	-5.74511300	1.44017100	-0.26925200
H	-5.53501500	2.48970900	-0.49635600
H	-6.05475500	0.93999500	-1.19377100
H	-6.58209400	1.41618400	0.43509900
C	-4.83602300	-0.73493800	0.64571000
H	-5.14945800	-1.25767200	-0.26535800
H	-3.96635100	-1.25362300	1.05899300
H	-5.65497100	-0.80419400	1.37103200
C	-3.33699700	0.79641000	-0.69460600
H	-3.62779900	0.29759500	-1.62627800
H	-3.07316700	1.83241200	-0.93686300
H	-2.44647100	0.29866200	-0.30037600

DMSO

S	-3.27939900	2.98494000	-7.44762100
O	-3.55745100	1.56474500	-7.88359300
C	-1.55230300	3.38463900	-7.93165800
H	-1.27240900	4.36726000	-7.53938700
H	-0.87854300	2.60772600	-7.55820300
H	-1.52244100	3.39995700	-9.02380900
C	-2.96301600	2.95597400	-5.63757000
H	-2.20016700	2.20492500	-5.41156500
H	-2.64909200	3.94762000	-5.29720900
H	-3.90264900	2.67878700	-5.15368000

Cat1.

Ru	-1.16571500	1.31918900	-0.71028100
C	-1.44129000	0.63188600	-2.74434700
C	-2.68939200	1.09660100	-2.22097500
C	-3.27758000	0.50299500	-1.05541200
C	-2.50480200	-0.47566500	-0.39447700
C	-1.24949200	-0.93165000	-0.91782300
C	-0.71608600	-0.44045200	-2.13017100
H	-1.00937300	1.14369000	-3.59912200
H	-3.17676900	1.94502700	-2.69139300
H	-2.83496600	-0.85112400	0.56913900
H	-0.67184900	-1.63203000	-0.32483400
C	0.58992300	-0.92172800	-2.73385100
H	1.04131500	-0.05707500	-3.23747400
C	0.26853000	-1.98293200	-3.81290100
H	-0.18684600	-2.87401500	-3.36629300
H	-0.41423200	-1.59668100	-4.57742500
H	1.19324200	-2.28939800	-4.31151400
C	1.59890200	-1.45331000	-1.70649000
H	2.54107400	-1.68573400	-2.21122200
H	1.81369400	-0.71551000	-0.92531600
H	1.25384900	-2.37898200	-1.23127100

C	-4.58423000	0.99990400	-0.50530900
H	-4.69817000	2.07714400	-0.65650600
H	-5.41089800	0.49911800	-1.02511500
H	-4.67778300	0.78472300	0.56214900
C	0.39851400	4.82482900	0.25791100
H	0.22303000	5.57407200	-0.52497500
H	1.47691000	4.71109300	0.38738700
H	-0.07468200	5.17857900	1.17676700
C	-0.21059900	3.53780700	-0.17557000
O	-1.44191800	3.24424100	0.05486600
O	0.44810700	2.64667100	-0.82734600

IM1

Ru	-2.02070000	3.28178000	-6.33655700
C	-1.20832300	1.73820300	-7.68910900
C	-2.13898800	2.51597000	-8.41059700
C	-3.49354600	2.65683900	-7.95092400
C	-3.84588500	2.01043300	-6.74535200
C	-2.89666000	1.21600200	-6.02316500
C	-1.57354300	1.04331300	-6.48175600
H	-0.17293900	1.71734100	-8.01470500
H	-1.81055600	3.07262800	-9.28208000
H	-4.83310400	2.16644700	-6.32705600
H	-3.19384800	0.79954800	-5.06810800
C	-0.53344600	0.20061200	-5.76472800
H	0.42932200	0.71219700	-5.89603600
C	-0.43390500	-1.17702900	-6.45782700
H	-1.37242300	-1.73436000	-6.35830000
H	-0.20832800	-1.08015900	-7.52521600
H	0.36328000	-1.77014100	-5.99794100
C	-0.78340500	0.04551300	-4.25880700
H	0.06518000	-0.46770700	-3.79591000
H	-0.90132800	1.01913100	-3.77002200
H	-1.67559500	-0.55706300	-4.05199900
C	-4.46776000	3.51399000	-8.70956900
H	-3.98863200	4.42612700	-9.07821400
H	-4.84540400	2.95920500	-9.57787900
H	-5.32118800	3.79196500	-8.08702700
C	0.18529300	6.60645800	-6.04305100
H	1.25895200	6.42142000	-6.12970100
H	-0.02357300	6.96140800	-5.02609500
H	-0.12959100	7.37252600	-6.75468800
C	-0.58045300	5.34099400	-6.26481000
O	-1.73617900	5.33819300	-6.80954900
O	-0.13588500	4.20387000	-5.87790700
C	-6.92913000	3.18868400	-3.47989200
C	-5.58580500	3.33969800	-3.13050700
C	-4.73273800	4.11016600	-3.93313000
C	-5.24041900	4.74766700	-5.07681600
C	-6.58968500	4.61583000	-5.40553100
C	-7.43324900	3.82926300	-4.61405600
H	-7.58237600	2.57913900	-2.86245300
H	-5.19476500	2.84948600	-2.24309600
H	-4.58201300	5.36428500	-5.68214000
H	-6.98781000	5.13737800	-6.27160600
H	-8.48235300	3.72537400	-4.87520100
C	-3.30017100	4.31192700	-3.57169500
O	-2.35651900	4.12507600	-4.41468800
C	-3.00224100	4.75886000	-2.28049800
H	-3.75696300	5.09343800	-1.58048200
S	-1.41280400	5.28441900	-1.92384600
O	-0.90201300	6.53680000	-2.53124200
C	-1.41615000	5.43059300	-0.12343000
H	-1.65500200	4.46758900	0.33227400
H	-0.42404000	5.77950300	0.17113000
H	-2.16396400	6.18264800	0.13915500
C	-0.22617100	3.96051500	-2.25806700
H	0.72187900	4.26618700	-1.80835200
H	-0.59039900	3.02420700	-1.83109800
H	-0.13664600	3.90030000	-3.34437800

TS1

Ru	-4.16122600	2.50908100	-4.77073900
C	-4.96373500	0.68251700	-5.82196200
C	-6.02331500	1.32201800	-5.14342000
C	-5.99017200	1.54093600	-3.72364500
C	-4.80164100	1.17563400	-3.05954500
C	-3.71829600	0.52455900	-3.73886500
C	-3.79174300	0.23981500	-5.11850700
H	-5.01726100	0.55739700	-6.89821900
H	-6.87671700	1.67740500	-5.71143900
H	-4.69191400	1.41290800	-2.00566400
H	-2.81891800	0.28837700	-3.18358400
C	-2.68082700	-0.44314500	-5.89403800
H	-2.62729600	0.06604500	-6.86472500
C	-3.07708300	-1.91623400	-6.14618500
H	-3.14486100	-2.47206700	-5.20405100
H	-4.04127000	-1.99941700	-6.65924400
H	-2.32054800	-2.40155500	-6.77121600
C	-1.29745100	-0.34269500	-5.23914200

H	-0.54709600	-0.78197800	-5.90386300
H	-1.01907500	0.70017300	-5.05759500
H	-1.25003900	-0.89564700	-4.29334500
C	-7.15628700	2.15394900	-2.99835200
H	-7.63720000	2.93033000	-3.59937200
H	-7.90670500	1.38278800	-2.78304800
H	-6.84689600	2.59467100	-2.04660800
C	-1.47980200	3.87046600	-7.98454900
H	-1.10940700	4.89402000	-8.08246600
H	-1.88706900	3.57610700	-8.95857900
H	-0.66864200	3.19077300	-7.71915600
C	-2.59041400	3.82033000	-6.95945800
O	-2.59555700	2.83249800	-6.14529700
O	-3.46444000	4.72739900	-6.96300400
C	-6.99646600	6.40578500	-3.97062600
C	-5.74559700	6.22559800	-3.37552600
C	-4.86214200	5.27403000	-3.89087900
C	-5.21874600	4.46929900	-5.00899000
C	-6.48028900	4.69032900	-5.59643600
C	-7.36133100	5.64511500	-5.08586700
H	-7.68251900	7.14527600	-3.56837400
H	-5.47761200	6.81113000	-2.50068800
H	-4.33442900	4.41502100	-5.94388300
H	-6.75323000	4.14044200	-6.49394900
H	-8.32538800	5.80616000	-5.56001100
C	-3.52516900	4.99581000	-3.31886500
O	-3.01970400	3.82367800	-3.56003100
C	-2.83396800	5.94820700	-2.58665400
H	-3.16038500	6.96360200	-2.40721100
S	-1.24813500	5.66159000	-1.99641600
O	-0.69589700	6.81976000	-1.27139500
C	-1.26669300	4.18199800	-0.95828400
H	-1.74170400	3.37736000	-1.52239800
H	-0.23551800	3.94221000	-0.68731700
H	-1.84521900	4.44253000	-0.06925800
C	-0.18589900	5.17961300	-3.37779400
H	0.78035800	4.86609300	-2.97480400
H	-0.68355200	4.37984200	-3.93090900
H	-0.07137500	6.07530500	-3.99264600

IM2

Ru	-4.25244800	2.54430200	-4.61351400
C	-4.84163600	0.68353200	-5.74738000
C	-5.96759500	1.16948100	-5.05111800
C	-5.90942500	1.37581200	-3.63317800
C	-4.67210200	1.08139600	-3.00388500
C	-3.59202000	0.42239000	-3.69119500
C	-3.65612300	0.20870700	-5.06626300
H	-4.88804400	0.60443800	-6.82941600
H	-6.86388900	1.43907700	-5.59927800
H	-4.56003600	1.30443200	-1.94736600
H	-2.69897900	0.16502000	-3.13397600
C	-2.55209800	-0.43719400	-5.88429900
H	-2.48099300	0.13910200	-6.81757200
C	-2.96428700	-1.87993100	-6.25516900
H	-3.04967700	-2.50368500	-5.35804400
H	-3.92596900	-1.90826400	-6.77882100
H	-2.21093700	-2.32939500	-6.91040700
C	-1.17340800	-0.41292400	-5.21249400
H	-0.42044500	-0.81173600	-5.89936200
H	-0.87548600	0.60538400	-4.93883100
H	-1.14847100	-1.03678200	-4.31114000
C	-7.09455000	1.88364000	-2.85905200
H	-7.66483500	2.61804700	-3.43432500
H	-7.76426700	1.04921600	-2.61570900
H	-6.78409200	2.35004300	-1.91985300
C	-1.95566900	4.15717300	-8.21233700
H	-1.64826800	5.20782600	-8.24826500
H	-2.34285600	3.90129300	-9.20389600
H	-1.10142400	3.52644500	-7.96711900
C	-3.03910200	3.98711500	-7.18724800
O	-2.89458100	3.27194300	-6.18652000
O	-4.13783400	4.66397400	-7.45630300
C	-6.60129300	6.82712900	-4.50439300
C	-5.39208500	6.61007300	-3.84711400
C	-4.76075700	5.36064600	-3.92395500
C	-5.33362100	4.28855200	-4.66141800
C	-6.55004200	4.54053700	-5.32192500
C	-7.17648800	5.79244600	-5.24438800
H	-7.08929800	7.79473600	-4.44213700
H	-4.94877300	7.41814100	-3.27188500
H	-4.78038200	4.56823700	-6.71140400
H	-7.02724200	3.75923400	-5.90930200
H	-8.11718200	5.95492000	-5.76394100
C	-3.48064700	5.03320700	-3.27164200
O	-3.08074400	3.80099400	-3.38785200
C	-2.72359600	5.96887600	-2.57739500
H	-2.97226800	7.01181500	-2.43885100
S	-1.23824500	5.56556900	-1.83259000

O	-0.60934000	6.71738300	-1.16032900
C	-1.50228800	4.20071100	-0.67601700
H	-2.02545900	3.40355500	-1.20697400
H	-0.52996300	3.87640800	-0.29722300
H	-2.11317100	4.60595500	0.13367400
C	-0.12872900	4.84142800	-3.06395700
H	0.75202100	4.45074100	-2.54829000
H	-0.67556100	4.06253400	-3.59903100
H	0.15138400	5.65993700	-3.73082000

IM3

Ru	-4.86858900	2.28806700	-4.37674100
C	-5.41479900	0.47459900	-5.54375200
C	-6.49274300	0.78205100	-4.68220300
C	-6.25456000	0.96497100	-3.29023900
C	-4.90792200	0.76065900	-2.83178100
C	-3.88674900	0.22028900	-3.67809200
C	-4.11038600	0.09006000	-5.05076900
H	-5.58833000	0.46410500	-6.61570600
H	-7.47197300	0.99102900	-5.09811400
H	-4.68042300	0.95774700	-1.78871200
H	-2.90461800	0.03358500	-3.26036400
C	-3.06698000	-0.39433900	-6.04186500
H	-3.21801500	0.18339300	-6.96429600
C	-3.33545600	-1.88039800	-6.37474300
H	-3.19432800	-2.50996900	-5.48883000
H	-4.35432700	-2.03807200	-6.74422400
H	-2.64004400	-2.22162200	-7.14846700
C	-1.61690900	-0.18555000	-5.58593100
H	-0.93216100	-0.46678900	-6.39187700
H	-1.42085300	0.86143500	-5.32688700
H	-1.36729500	-0.80925300	-4.71963000
C	-7.34830300	1.35394500	-2.33525200
H	-8.11186500	1.96491400	-2.82312200
H	-7.83375500	0.44969200	-1.94606500
H	-6.95062800	1.91416000	-1.48435100
C	-7.11257100	6.57805900	-4.66775900
C	-5.78200500	6.44776900	-4.27672900
C	-5.20807000	5.17356200	-4.16950200
C	-5.95957700	4.00307000	-4.46242000
C	-7.29715300	4.16680100	-4.85517200
C	-7.86763000	5.43773300	-4.96022100
H	-7.56035700	7.56406900	-4.74506500
H	-5.20375100	7.34059400	-4.05605600
H	-7.90740100	3.30199500	-5.10046000
H	-8.90351500	5.54118900	-5.27170900
C	-3.81118600	4.92069500	-3.77439200
O	-3.44095800	3.66134300	-3.73636500
C	-2.91819100	5.92689000	-3.46580000
H	-3.12706900	6.98805000	-3.48388100
S	-1.29071500	5.61150800	-3.00220000
O	-0.54489300	6.84796900	-2.71002100
C	-1.29811000	4.49197200	-1.58423000
H	-1.92252800	3.63204200	-1.83273200
H	-0.26517100	4.20764700	-1.36872300
H	-1.71924700	5.06203300	-0.75304000
C	-0.48228800	4.64542200	-4.29756500
H	0.51153300	4.36239800	-3.94160800
H	-1.10713300	3.77766000	-4.51593700
H	-0.40418300	5.30634600	-5.16381500

IM4

Ru	-4.23583500	2.99457700	-4.41562600
C	-3.68299100	0.82017400	-3.52699600
C	-3.93507000	0.72442800	-4.90875600
C	-5.18026000	1.13222800	-5.48087800
C	-6.11447200	1.73643400	-4.60464700
C	-5.87122200	1.85418700	-3.20912500
C	-4.65198900	1.39196300	-2.65420700
H	-2.72520500	0.49593400	-3.13776700
H	-3.15975300	0.34251000	-5.56371900
H	-7.04886300	2.11521800	-5.00588600
H	-6.62217600	2.31779700	-2.58230400
C	-4.37371300	1.43357000	-1.15912700
H	-3.33002900	1.75650700	-1.03913500
C	-4.48409900	0.00381700	-0.58110000
H	-5.50425400	-0.38162400	-0.68966200
H	-3.80413900	-0.69187400	-1.08284400
H	-4.23716200	0.00921200	0.48604400
C	-5.27360800	2.39940400	-0.37607100
H	-4.93632700	2.46011500	0.66437000
H	-5.26389900	3.40688300	-0.80504600
H	-6.31263900	2.05195300	-0.35113900
C	-5.51245200	0.85174700	-6.92184300
H	-4.61931000	0.88755600	-7.54974500
H	-5.94535500	-0.15341100	-7.00943400
H	-6.24784500	1.56212100	-7.31186800
C	-6.83058600	7.07353000	-5.08786300
C	-5.82674800	6.98123400	-4.13373500
C	-5.07326600	5.79705400	-4.00686000

C	-5.31508800	4.67827400	-4.84812900
C	-6.33091400	4.80980900	-5.81080600
C	-7.08121100	5.98012200	-5.92651600
H	-7.41501600	7.98391200	-5.18007100
H	-5.63770300	7.83364400	-3.48740800
H	-6.53781600	3.99311300	-6.49698900
H	-7.86336700	6.04480700	-6.67898100
C	-4.00877000	5.59797200	-3.01209100
O	-3.37959600	4.46143600	-3.06446500
C	-3.71977800	6.59220800	-2.06658200
H	-4.24267000	7.53872000	-2.03822900
S	-2.62569600	6.53495200	-0.77040700
O	-2.93028700	7.53336600	0.27285700
C	-2.55473300	4.85975500	-0.10589400
H	-2.49420200	4.15751300	-0.93864400
H	-1.69710600	4.79662100	0.56771100
H	-3.48560800	4.72051700	0.44726700
C	-0.91514600	6.81173600	-1.31629500
H	-0.26248900	6.75937100	-0.44084400
H	-0.63808200	6.06635000	-2.06358200
H	-0.89304300	7.81615500	-1.74496400
C	-4.58561500	6.31272800	-8.94441300
C	-3.98490300	5.51921600	-7.96812300
C	-3.72862200	4.15997000	-8.21693300
C	-4.08830600	3.61561200	-9.46456100
C	-4.69924300	4.40614900	-10.43180100
C	-4.94692000	5.75868500	-10.17399700
H	-4.77676600	7.36222500	-8.74153500
H	-3.72998400	5.96817800	-7.01485600
H	-3.86768200	2.57153300	-9.65765200
H	-4.97596200	3.97411800	-11.38901800
H	-5.41710600	6.37909200	-10.93176600
C	-3.01918400	3.26284300	-7.26206000
O	-2.62205100	2.14663100	-7.62567000
C	-2.73195600	3.69460200	-5.86103300
H	-2.63574700	4.77065700	-5.70429900
S	-1.09353900	3.04033400	-5.42929500
O	-0.98474600	1.69926200	-4.81997600
C	0.06154900	3.15796000	-6.82686000
H	0.00321700	4.15739600	-7.26506300
H	1.05947300	2.95157600	-6.43272600
H	-0.24219600	2.39937800	-7.54763700
C	-0.46451100	4.28297800	-4.28180600
H	0.46691600	3.89145700	-3.86633100
H	-0.29747200	5.22795300	-4.80498400
H	-1.24208700	4.38830600	-3.52070300

TS2

Ru	-4.22339800	3.10988600	-4.46954300
C	-3.68206600	0.94683900	-3.48576000
C	-3.87300400	0.78453000	-4.87073000
C	-5.10630800	1.13142600	-5.49677100
C	-6.08308700	1.73861100	-4.67210700
C	-5.92554600	1.84214700	-3.26002400
C	-4.72988100	1.43318700	-2.64694100
H	-2.72107000	0.68980800	-3.05607800
H	-3.05718100	0.40815900	-5.47743000
H	-7.01288000	2.07690200	-5.11786200
H	-6.73044400	2.26945500	-2.67441400
C	-4.52045600	1.46607700	-1.14212600
H	-3.47300500	1.75428600	-0.97782500
C	-4.70085100	0.04037800	-0.57180900
H	-5.72860100	-0.31039100	-0.71919200
H	-4.02577500	-0.67606800	-1.05101600
H	-4.49291000	0.03573600	0.50353100
C	-5.41842100	2.46677300	-0.40135700
H	-5.13054000	2.51410900	0.65458800
H	-5.34626800	3.47395900	-0.82652100
H	-6.47125600	2.16376000	-0.42641800
C	-5.38739200	0.80141300	-6.93787700
H	-4.47818100	0.84024700	-7.54174500
H	-5.79381400	-0.21609600	-7.00567100
H	-6.12822400	1.48216200	-7.36811100
C	-6.97214100	7.12715300	-4.76809700
C	-5.92272700	7.00473300	-3.86608100
C	-5.13444400	5.83867000	-3.85060200
C	-5.39241300	4.77155100	-4.74816000
C	-6.45056100	4.92778800	-5.65650300
C	-7.23373000	6.08393000	-5.66421000
H	-7.58061200	8.02628400	-4.77838800
H	-5.71956300	7.82280700	-3.18075400
H	-6.66607500	4.14796600	-6.38208300
H	-8.04992200	6.17556100	-6.37658700
C	-4.00623700	5.60577000	-2.93498200
O	-3.32281000	4.51744900	-3.13439400
C	-3.72358100	6.50863900	-1.90419200
H	-4.29951900	7.41029800	-1.74418000
S	-2.53631400	6.38245700	-0.69418500

O	-2.84259400	7.20856200	0.48978300
C	-2.28944200	4.64791900	-0.26489900
H	-2.20068700	4.06755100	-1.18441200
H	-1.40232100	4.58266900	0.36916400
H	-3.18045600	4.35154800	0.29230900
C	-0.90285200	6.87731300	-1.31222700
H	-0.17815700	6.76032700	-0.50213000
H	-0.63405200	6.27059600	-2.17847700
H	-0.99199000	7.92956100	-1.59153400
C	-4.72305400	6.14026400	-9.14596200
C	-4.15674200	5.41298500	-8.10007600
C	-3.68941000	4.10493400	-8.31930200
C	-3.80079000	3.54361400	-9.60565500
C	-4.36925300	4.27119500	-10.64535000
C	-4.83081600	5.57228500	-10.41732400
H	-5.08120300	7.14978500	-8.96732000
H	-4.09385000	5.86425100	-7.11670700
H	-3.42674400	2.53854100	-9.76809300
H	-4.45050800	3.82991800	-11.63439800
H	-5.27119200	6.14221100	-11.23059900
C	-3.03462700	3.28194400	-7.26226200
O	-2.54897700	2.17804000	-7.53416800
C	-2.98561700	3.79557400	-5.85570400
H	-2.70595400	4.85057100	-5.75835000
S	-0.90677300	3.03876800	-5.35663600
O	-0.85639800	1.84794500	-4.45296600
C	0.29682700	2.81261400	-6.70818800
H	0.31378800	3.71088000	-7.33143200
H	1.27705200	2.61918300	-6.26421400
H	-0.04916700	1.95899100	-7.29083300
C	-0.16383700	4.45164300	-4.48839100
H	0.79566100	4.14298300	-4.06528400
H	-0.03583400	5.28927400	-5.18001600
H	-0.87777200	4.71068000	-3.70497800

IM5

Ru	-4.67963400	3.30653900	-4.04525100
C	-5.81480500	1.48554200	-4.93051600
C	-6.74224700	2.26377400	-4.19385100
C	-6.69809900	2.30834100	-2.75970700
C	-5.57572700	1.74663200	-2.15322200
C	-4.57546000	1.06949600	-2.91494600
C	-4.73000600	0.81894200	-4.29229700
H	-5.95002700	1.37922400	-6.00332300
H	-7.57707200	2.72240400	-4.71334400
H	-5.44698800	1.83036700	-1.07827600
H	-3.71719100	0.66335700	-2.39058800
C	-3.81244900	-0.09708800	-5.08471800
H	-3.87746000	0.21821700	-6.13465400
C	-4.34543000	-1.54581100	-5.00209300
H	-4.30273600	-1.92119700	-3.97311200
H	-5.38271500	-1.61708100	-5.34646800
H	-3.73494600	-2.20450900	-5.62821800
C	-2.33722900	-0.02200200	-4.66256700
H	-1.73061500	-0.63397500	-5.33731300
H	-1.96005300	1.00565700	-4.70158100
H	-2.18132500	-0.41141600	-3.64971700
C	-7.79111800	2.98582200	-1.98132600
H	-7.97079400	3.99759300	-2.36120400
H	-8.72891500	2.42501200	-2.07728800
H	-7.54361700	3.05410700	-0.91887900
C	-6.23330700	7.83080200	-3.19364600
C	-5.02244700	7.35002700	-2.70628000
C	-4.60657900	6.04580500	-3.01739100
C	-5.41106500	5.20334400	-3.82339000
C	-6.61547000	5.71349700	-4.32057800
C	-7.02681500	7.01226100	-4.00356000
H	-6.55579900	8.83870600	-2.95148400
H	-4.40508500	7.99063900	-2.08273700
H	-7.24294400	5.10911200	-4.97029900
H	-7.96769700	7.38889500	-4.39634700
C	-3.34800500	5.43529300	-2.57323700
O	-3.16706200	4.19129700	-2.93159000
C	-2.39233400	6.10653500	-1.82863700
H	-2.46917500	7.12913600	-1.48583000
S	-0.87064900	5.42147900	-1.43598000
O	-0.04935400	6.32689600	-0.61010800
C	-1.12780100	3.83876200	-0.60123300
H	-1.78457000	3.22973000	-1.22417700
H	-0.15076000	3.37358100	-0.44923800
H	-1.59364100	4.07401500	0.35822400
C	-0.01209600	4.95718100	-2.95387300
H	0.86095600	4.35832400	-2.68228600
H	-0.70279500	4.42755700	-3.61424900
H	0.29814800	5.89836700	-3.41325500
C	-5.10937000	6.86749500	-8.41514200
C	-4.71384200	5.87961300	-7.51609700
C	-3.42259200	5.90394400	-6.96506400
C	-2.53191800	6.92986000	-7.32397400

C	-2.93049800	7.91699900	-8.21885500
C	-4.21841400	7.88572600	-8.76574100
H	-6.10723100	6.84610000	-8.84307500
H	-5.40815700	5.09065500	-7.23906500
H	-1.53532000	6.92934200	-6.89479500
H	-2.24144300	8.70924100	-8.49605800
H	-4.52685900	8.65545300	-9.46749700
C	-2.96032900	4.85366600	-6.02839800
O	-1.82204300	4.82896100	-5.56205200
C	-3.86257500	3.71066700	-5.67100600
H	-3.92794700	2.97012300	-6.48406300

TS3

Ru	-4.41300000	2.81095000	-3.60210000
C	-4.59156900	0.96112900	-4.77579300
C	-5.88357000	1.32593500	-4.30687400
C	-6.18179700	1.36607800	-2.89948300
C	-5.11326200	1.13910900	-2.02286000
C	-3.79632200	0.82057000	-2.50317400
C	-3.51708900	0.65274400	-3.87058600
H	-4.40970000	0.91575100	-5.84576100
H	-6.67209900	1.53434800	-5.02426300
H	-5.26599300	1.25360700	-0.95382900
H	-2.99897000	0.71168700	-1.77547400
C	-2.16043600	0.21915800	-4.40190100
H	-2.13862600	0.49593700	-5.46436100
C	-2.01538900	-1.31434400	-4.31520400
H	-2.01674400	-1.64805000	-3.27028800
H	-2.83654800	-1.82531200	-4.83087300
H	-1.07311500	-1.63934000	-4.77203700
C	-0.97994300	0.93297900	-3.72313300
H	-0.03923800	0.63979900	-4.20382700
H	-1.08779600	2.02001100	-3.79579700
H	-0.89861500	0.66253500	-2.66288200
C	-7.57586300	1.66724900	-2.41552800
H	-8.03541500	2.46281300	-3.01146000
H	-8.21714000	0.77905200	-2.49628800
H	-7.57240200	1.98491900	-1.36831000
C	-6.71790400	7.06489800	-4.36454500
C	-5.71612000	6.90856100	-3.41072400
C	-5.01146300	5.70264200	-3.30668200
C	-5.27640800	4.61001900	-4.19967400
C	-6.31392000	4.80543700	-5.15106600
C	-7.01991800	5.99830700	-5.22848500
H	-7.26699500	7.99950600	-4.43306600
H	-5.50310700	7.72367700	-2.72365700
H	-6.54913000	4.00130900	-5.84528100
H	-7.81456000	6.10836600	-5.96328800
C	-3.99676900	5.42108800	-2.28582600
O	-3.59118400	4.20092900	-2.19965700
C	-3.47763900	6.40757700	-1.43174100
H	-3.70461000	7.46358200	-1.45996100
S	-2.16525300	6.05297100	-0.42030700
O	-1.73827700	7.20232900	0.41163500
C	-2.58428500	4.64031900	0.63326800
H	-2.93903400	3.83957100	-0.01735900
H	-1.69389800	4.35733900	1.20011400
H	-3.37450300	4.98779200	1.30196400
C	-0.77844500	5.41815700	-1.39270700
H	-0.06183300	4.94363800	-0.71726600
H	-1.17027300	4.74315200	-2.15906700
H	-0.33835300	6.29625700	-1.87021700
C	-2.73147600	6.30756800	-8.71323100
C	-3.11131100	5.69104300	-7.52012900
C	-2.15150300	5.35005500	-6.55329200
C	-0.80456300	5.65657100	-6.80844600
C	-0.42283800	6.25828500	-8.00491300
C	-1.38622000	6.58537800	-8.96411100
H	-3.48861800	6.57636900	-9.44574300
H	-4.16349700	5.50478100	-7.32674400
H	-0.07209000	5.41321200	-6.04553600
H	0.62582700	6.47760300	-8.19083200
H	-1.09078400	7.05950500	-9.89668800
C	-2.49892300	4.70930500	-5.23099000
O	-1.69600800	4.85700400	-4.28462000
C	-3.71258000	3.91420200	-5.15017500
H	-4.14296800	3.69378600	-6.12771400

IM6

Ru	-4.50428900	3.04313800	-3.87605700
C	-4.35451400	1.34474300	-5.32270900
C	-5.72778800	1.55511000	-5.04420700
C	-6.20819600	1.53776600	-3.69935100
C	-5.29344800	1.21795300	-2.65126300
C	-3.92476000	1.05489700	-2.94135700
C	-3.40903900	1.11583700	-4.27907500
H	-4.00337200	1.40894900	-6.34753900
H	-6.41185000	1.79035300	-5.85267100
H	-5.63714800	1.19725500	-1.62283400
H	-3.22960300	0.93482100	-2.11781500

C	-1.94700300	0.86314000	-4.60913400
H	-1.74115600	1.39078200	-5.54936300
C	-1.72966000	-0.64665600	-4.85161400
H	-1.92666500	-1.22459900	-3.94088100
H	-2.38371900	-1.02933700	-5.64264200
H	-0.69289400	-0.83358300	-5.15077900
C	-0.96830100	1.40058300	-3.55398800
H	0.05836700	1.29114400	-3.91764200
H	-1.14803100	2.46038600	-3.34620600
H	-1.03496700	0.84269700	-2.61201000
C	-7.65925600	1.80148700	-3.39922000
H	-8.10110700	2.48308200	-4.13053000
H	-8.21984700	0.85841600	-3.43450200
H	-7.78834500	2.23194700	-2.40236800
C	-7.61684400	5.94269000	-2.88870100
C	-6.39342000	5.76919900	-2.26790600
C	-5.24694700	5.38514800	-3.00136400
C	-5.35009800	5.17361400	-4.42066600
C	-6.63842600	5.33549300	-5.01248000
C	-7.73791400	5.72429800	-4.27596200
H	-8.48190300	6.24850900	-2.30807100
H	-6.29867800	5.92579400	-1.19787500
H	-6.72781300	5.18550700	-6.08504900
H	-8.69548200	5.87181800	-4.76672400
C	-3.93313500	5.24235000	-2.29386300
O	-3.23531100	4.19867600	-2.60898400
C	-3.57205700	6.15758400	-1.32582800
H	-4.11189000	7.07170600	-1.11713400
S	-2.02983600	6.13250600	-0.56728100
O	-1.88289600	7.19162800	0.44759300
C	-1.77244500	4.49171200	0.14066000
H	-1.96971000	3.75198900	-0.63718900
H	-0.74642900	4.44000900	0.51301500
H	-2.48362800	4.40187900	0.96476500
C	-0.74512000	6.25153400	-1.83076300
H	0.22385600	6.10966100	-1.34578100
H	-0.94679400	5.50774000	-2.60298100
H	-0.83169300	7.25495000	-2.25212600
C	-1.11530200	3.67606200	-8.17551100
C	-2.09308500	3.96763100	-7.22452100
C	-1.89854700	4.99833600	-6.28941100
C	-0.70224700	5.73381700	-6.33772200
C	0.27365500	5.44515400	-7.28744600
C	0.07042100	4.41367000	-8.20874400
H	-1.28111600	2.87970700	-8.89562000
H	-3.00967700	3.38795200	-7.21820400
H	-0.56461000	6.54012200	-5.62544600
H	1.19014700	6.02745200	-7.31660300
H	0.82890800	4.19020500	-8.95352200
C	-2.90534700	5.37840000	-5.24229900
O	-2.63371600	6.25568800	-4.42177500
C	-4.22959800	4.69133900	-5.24207400
H	-4.54843600	4.41120200	-6.24489200

TS4

Ru	-5.14752800	2.87110300	-4.58759000
C	-5.65480200	1.83164200	-2.68819400
C	-4.25905400	1.65942900	-2.92553800
C	-3.81059500	1.10657400	-4.15208500
C	-4.80021700	0.77810200	-5.13611000
C	-6.19194900	0.90910700	-4.85070100
C	-6.65953700	1.40376900	-3.60488600
H	-5.96044900	2.35895800	-1.79070400
H	-3.53193800	2.07928500	-2.23721900
H	-4.48841100	0.45521100	-6.12375000
H	-6.89865400	0.72090300	-5.65091000
C	-8.13078900	1.52576600	-3.24826900
H	-8.21982600	2.40421200	-2.59587100
C	-8.56021500	0.28742600	-2.43038900
H	-8.47983300	-0.62639700	-3.03055700
H	-7.94517200	0.15925900	-1.53306300
H	-9.60332300	0.39137400	-2.11395300
C	-9.05227300	1.73658700	-4.45848200
H	-10.06959500	1.94629800	-4.11342400
H	-8.72023300	2.57439700	-5.08012400
H	-9.10803900	0.84113100	-5.08900300
C	-2.34604800	0.99767700	-4.45640200
H	-1.77895700	1.70979600	-3.85287500
H	-2.00187800	-0.01722800	-4.21810400
H	-2.14618700	1.18700400	-5.51404900
C	-6.20023000	4.92739600	-1.06397100
C	-4.79952800	4.86929300	-1.12533800
C	-4.11920500	5.05035000	-2.32760500
C	-4.85158600	5.27892100	-3.53736900
C	-6.26662300	5.28156400	-3.45095200
C	-6.93464400	5.11775000	-2.22796000
H	-6.70266800	4.83474200	-0.10492500
H	-4.22769400	4.71275200	-0.21467900
H	-6.84334700	5.53545800	-4.33295700

H	-8.01822000	5.18312300	-2.19443400
C	-2.60204400	4.97114700	-2.27815300
O	-2.07213100	3.83304800	-2.15273600
C	-1.90329400	6.18476400	-2.30974000
H	-2.33557900	7.17586100	-2.34940000
S	-0.20377000	6.20373600	-2.14533700
O	0.37085600	7.56318900	-2.20904100
C	0.25241700	5.37144700	-0.60469600
H	-0.26571500	4.40975700	-0.58681000
H	1.33845500	5.25467100	-0.57517200
H	-0.08611800	6.02184900	0.20462700
C	0.52887900	5.12047200	-3.39245000
H	1.60019100	5.04163500	-3.19251900
H	0.02250600	4.15563600	-3.32497200
H	0.34609700	5.59599000	-4.35841400
C	-0.63918600	7.20145500	-7.04446700
C	-1.63879600	6.74128700	-6.18839300
C	-2.24677800	5.49265200	-6.41163500
C	-1.82722900	4.71475100	-7.50512900
C	-0.83250700	5.17998600	-8.36150100
C	-0.23656100	6.42361500	-8.13368800
H	-0.17537300	8.16621000	-6.86122700
H	-1.92991100	7.34473100	-5.33503500
H	-2.29723700	3.75271800	-7.67796800
H	-0.52317200	4.57591200	-9.20950300
H	0.53915500	6.78586200	-8.80220900
C	-3.30956900	4.97516700	-5.51572500
O	-3.50985800	3.69806200	-5.54879800
C	-4.16919200	5.80871700	-4.77740700
H	-3.90284300	6.86246500	-4.71913000
C	-7.45289400	4.55449900	-8.10760900
C	-6.52867800	4.61426200	-6.91469400
O	-6.31710700	3.55144400	-6.26704500
O	-6.03238300	5.76857200	-6.65663300
H	-5.20240400	5.79072800	-5.82559400
H	-8.21250300	5.33754400	-8.02450400
H	-7.92429200	3.57472100	-8.18996400
H	-6.87452800	4.76120800	-9.01481000

IM7

Ru	-3.25673200	2.85953500	-3.51675700
C	-5.32447100	1.99298500	-3.29052300
C	-4.87750800	2.32397700	-2.00082600
C	-3.61286000	1.83088100	-1.52799900
C	-2.85816300	0.98588200	-2.37693500
C	-3.33908600	0.63810700	-3.68017800
C	-4.57197000	1.12611900	-4.16480500
H	-6.22660200	2.46174900	-3.67076400
H	-5.44170000	3.02566300	-1.39706400
H	-1.87441600	0.65046500	-2.06757900
H	-2.68706300	0.07378200	-4.33548700
C	-5.11127200	0.82001200	-5.55055800
H	-5.54359900	1.75777100	-5.92385400
C	-6.24777600	-0.22022400	-5.44212700
H	-5.86816600	-1.17779400	-5.06736500
H	-7.04617600	0.11373500	-4.77015400
H	-6.69022800	-0.39527200	-6.42832200
C	-4.03819400	0.36702500	-6.54993600
H	-4.47789900	0.28751100	-7.54933300
H	-3.20329500	1.07444800	-6.59779100
H	-3.63786500	-0.62231100	-6.29801300
C	-3.11177400	2.20828100	-0.16170100
H	-3.35968400	3.24477500	0.08396100
H	-3.58503500	1.56507400	0.59112300
H	-2.02959300	2.07427400	-0.08306800
C	-6.41131100	6.27342700	-1.02846700
C	-5.05107900	5.98557400	-0.92051300
C	-4.23984200	5.92924000	-2.06353500
C	-4.78215600	6.21109300	-3.33204800
C	-6.14872800	6.50935500	-3.41917100
C	-6.96200000	6.53001300	-2.28584700
H	-7.03320000	6.30705100	-0.13877500
H	-4.61023500	5.79619400	0.05474800
H	-6.57986700	6.74477100	-4.38948200
H	-8.01804600	6.76463800	-2.38244000
C	-2.79139100	5.56908000	-1.93582700
O	-2.32695600	4.53715800	-2.52403000
C	-1.98898700	6.41036100	-1.15609000
H	-2.34847500	7.35940400	-0.77853000
S	-0.28534800	6.25187800	-1.13056100
O	0.51905000	6.70612600	-2.28562000
C	0.19953300	7.16932600	0.35021700
H	-0.28967200	6.75311100	1.23282400
H	1.28745600	7.10607700	0.42436300
H	-0.09716500	8.20939800	0.19450500
C	0.14141100	4.53741400	-0.74934400
H	1.22867400	4.49715600	-0.65148400
H	-0.36049100	4.23044300	0.17016000
H	-0.19651400	3.94785700	-1.60208400

C	-5.50987000	6.88103200	-8.68026600
C	-5.11696300	6.67760500	-7.35919500
C	-4.59128400	5.43576800	-6.95584100
C	-4.45774000	4.40869800	-7.91218700
C	-4.83558000	4.62082300	-9.23219500
C	-5.36622000	5.85681400	-9.61838600
H	-5.92204100	7.84017500	-8.97835800
H	-5.23932200	7.48681200	-6.64770400
H	-4.03325200	3.46014500	-7.60402700
H	-4.71318800	3.82895600	-9.96514600
H	-5.66194900	6.02190800	-10.65042900
C	-4.17921600	5.16253200	-5.56348500
O	-4.03694700	3.97546200	-5.21847700
C	-3.91510600	6.28233100	-4.57971700
H	-4.04208700	7.25658800	-5.05499400
C	0.57781200	3.37195700	-5.61458300
C	-0.84283300	3.75110100	-5.11757200
O	-1.41585800	2.77976200	-4.43851600
O	-1.32832800	4.85568100	-5.34362500
H	-2.84930300	6.17143300	-4.35550600
C	0.53700100	2.01949500	-6.35389700
H	1.53921600	1.75478000	-6.71139800
H	0.18240100	1.21875500	-5.69864500
H	-0.12450400	2.06592200	-7.22774000
C	1.49874800	3.26135600	-4.37886600
H	2.52020200	3.01034500	-4.68873000
H	1.53846800	4.21430900	-3.83707600
H	1.14807000	2.47939400	-3.69621800
C	1.10009100	4.47133200	-6.55263900
H	2.11186300	4.22163400	-6.89379800
H	0.45826900	4.57818400	-7.43307700
H	1.13051300	5.44039700	-6.04749100

IM8

C	-7.56190700	6.21730400	-2.63201400
C	-6.28383800	5.95783700	-2.14013200
C	-5.26804400	5.46088800	-2.96827500
C	-5.54741500	5.21170600	-4.33142100
C	-6.83122600	5.49244000	-4.81051100
C	-7.83559200	5.98824900	-3.97851800
H	-8.33499500	6.59301700	-1.96712400
H	-6.07045000	6.12106600	-1.08783300
H	-7.04873200	5.31486300	-5.86150200
H	-8.82464400	6.18816500	-4.38221600
C	-3.90406400	5.18831600	-2.38927200
O	-3.22138300	4.21531600	-2.80185800
C	-3.44735700	6.07139300	-1.37835200
H	-3.89012300	7.01655500	-1.09627200
S	-1.86076100	5.90070700	-0.81962600
O	-1.46123700	6.90945000	0.18966200
C	-1.63889300	4.22359000	-0.17733100
H	-2.01242300	3.53909100	-0.94145100
H	-0.58001100	4.06861300	0.04312300
H	-2.23675100	4.16914300	0.73454600
C	-0.69550400	5.91989700	-2.21072000
H	0.30572100	5.68926200	-1.83912400
H	-1.05037500	5.18870700	-2.93940000
H	-0.73979300	6.92987100	-2.62267900
C	-1.27637400	3.33636900	-7.93009000
C	-2.28669200	3.74971800	-7.06148600
C	-2.25464600	5.03227300	-6.49340900
C	-1.19242500	5.89210300	-6.81580100
C	-0.18605300	5.48117700	-7.68444500
C	-0.22577800	4.20033400	-8.24367700
H	-1.30956400	2.33949900	-8.36124100
H	-3.09405600	3.06486700	-6.82386700
H	-1.18356000	6.88223100	-6.37166900
H	0.62927200	6.15690400	-7.92899800
H	0.55951200	3.87749200	-8.92222000
C	-3.30921700	5.53997100	-5.54903400
O	-3.18746700	6.63615000	-5.02538500
C	-4.52409800	4.64524800	-5.28935900
H	-5.00221000	4.44171600	-6.25715400
H	-4.14635400	3.69157500	-4.90858300

IM9

Ru	-3.67267000	2.06026700	-3.59365500
C	-4.64366800	0.20299100	-4.33810500
C	-5.58681500	0.91598800	-3.53536400
C	-5.26848500	1.26370800	-2.19812800
C	-4.02429400	0.80153500	-1.64509000
C	-3.10312800	0.11339400	-2.44185600
C	-3.38084000	-0.18723500	-3.82219400
H	-4.88087900	-0.01029700	-5.37601200
H	-6.52369100	1.24928500	-3.96891600
H	-3.76383500	1.07427100	-0.62782000
H	-2.13464800	-0.14354200	-2.02724300
C	-2.40882500	-0.97585100	-4.68616700
H	-2.67172200	-0.76148900	-5.73075000
C	-2.62383200	-2.48674800	-4.44946400

H	-2.38662700	-2.76160600	-3.41475400
H	-3.65894700	-2.78424800	-4.64919600
H	-1.97010900	-3.06808000	-5.10842300
C	-0.93573100	-0.58634500	-4.48559200
H	-0.31338600	-1.11714000	-5.21352800
H	-0.78317600	0.48895200	-4.61886400
H	-0.57109900	-0.86583800	-3.48993700
C	-6.21102800	2.07534500	-1.34959400
H	-6.96080200	2.58402400	-1.96186700
H	-6.73869800	1.41707400	-0.64768400
H	-5.67056700	2.82632500	-0.76500500
C	-7.07756600	6.04830200	-5.51115900
C	-6.24293200	6.02234700	-4.39825000
C	-5.15605700	5.13406000	-4.31970500
C	-4.89866500	4.24909500	-5.39933800
C	-5.76304600	4.28405200	-6.50702700
C	-6.83616900	5.16979900	-6.57008900
H	-7.91854800	6.73388200	-5.54429200
H	-6.46689700	6.66875600	-3.55605200
H	-5.57867300	3.60540300	-7.33636500
H	-7.48473500	5.17189500	-7.44146300
C	-4.34136800	5.07351300	-3.07731100
O	-3.73924800	3.99627400	-2.71562800
C	-4.23355800	6.20245300	-2.25266400
H	-4.65844000	7.17401400	-2.45759000
S	-3.52279600	6.01283800	-0.71591000
O	-4.15246400	5.10917100	0.27541200
C	-1.77865500	5.55395800	-0.89259500
H	-1.25929900	6.32659500	-1.46354100
H	-1.36276800	5.44291000	0.11164300
H	-1.77278500	4.60436500	-1.42834600
C	-3.45383800	7.70305400	-0.08000800
H	-2.97468900	7.65659000	0.90023900
H	-2.89528100	8.34383300	-0.76539400
H	-4.48424000	8.04905000	0.02881900
C	-1.44657500	6.67202800	-7.21510100
C	-2.18761700	5.62049600	-6.68189500
C	-1.62718900	4.79628400	-5.68833000
C	-0.31233800	5.04167200	-5.24229700
C	0.42226100	6.09196000	-5.77946400
C	-0.14475600	6.90908000	-6.76504400
H	-1.87999200	7.30319400	-7.98492200
H	-3.19464500	5.43343200	-7.03753300
H	0.11481200	4.39338300	-4.48424100
H	1.43701900	6.27550100	-5.43884700
H	0.43166600	7.72840800	-7.18525100
C	-2.36609000	3.65556400	-5.13959800
O	-1.80586800	2.83779200	-4.35550000
C	-3.79267200	3.26130300	-5.35994100
H	-3.82995300	2.56229300	-6.20170300

IM10

Ru	-4.26704500	2.93525500	-2.71293700
C	-6.10698000	1.82036800	-3.33008700
C	-6.18137300	2.08217900	-1.94214900
C	-5.17368800	1.61009600	-1.03819400
C	-4.04903600	0.97597800	-1.61297200
C	-3.96921700	0.69675200	-3.01507200
C	-5.01034300	1.08336800	-3.88933700
H	-6.87172600	2.21788500	-3.98875600
H	-7.00161800	2.67961800	-1.55657300
H	-3.21273300	0.70524900	-0.97565200
H	-3.07822800	0.22055500	-3.40508500
C	-4.98801500	0.81357500	-5.38183800
H	-5.38539100	1.71599700	-5.86473300
C	-5.95000600	-0.35512200	-5.69693500
H	-5.60379900	-1.28492600	-5.23135400
H	-6.96646800	-0.15557800	-5.34139300
H	-5.99716700	-0.51612400	-6.77893300
C	-3.59018900	0.54790300	-5.95574000
H	-3.65275100	0.45994600	-7.04516200
H	-2.89770300	1.36225500	-5.71919600
H	-3.16683800	-0.39281600	-5.58422300
C	-5.31064700	1.78844700	0.44893100
H	-5.81962800	2.72360300	0.69635100
H	-5.90683700	0.96499100	0.86243300
H	-4.33693400	1.77484600	0.94758800
C	-5.62682700	5.55933500	0.06874500
C	-4.51421100	4.98514300	-0.48946900
C	-4.23458600	5.09151200	-1.90237200
C	-5.14407300	5.86685800	-2.73657500
C	-6.26102700	6.48440200	-2.09717900
C	-6.49926600	6.32751300	-0.75202300
H	-5.82140300	5.47103400	1.13315500
H	-3.79137400	4.46399600	0.13206500
H	-6.94063200	7.07421100	-2.70598600
H	-7.36708500	6.80382500	-0.30375100
C	-2.75737500	4.99115200	-2.22084100
O	-2.33983200	3.76636800	-2.29145600

C	-1.95979600	6.10078000	-2.39847600
H	-2.28779100	7.12823600	-2.31412000
S	-0.33514500	5.95285700	-2.94597200
O	0.32626600	7.25782800	-3.12764800
C	0.57832000	4.90431300	-1.79342300
H	0.01912100	3.97551200	-1.66218400
H	1.57539900	4.73151200	-2.20614400
H	0.64269000	5.46347000	-0.85727100
C	-0.34010800	4.98580300	-4.47290500
H	0.69479200	4.75787300	-4.73958800
H	-0.93420500	4.08463500	-4.30644700
H	-0.80582900	5.62082200	-5.22983100
C	-3.18919600	4.77713400	-8.54515600
C	-3.57308400	4.45432800	-7.24595100
C	-3.82409100	5.46599700	-6.30217200
C	-3.65749600	6.80889000	-6.68849800
C	-3.26929300	7.12930900	-7.98812200
C	-3.03812300	6.11509300	-8.92092300
H	-3.01028300	3.98644300	-9.26834000
H	-3.69201500	3.41832500	-6.95002500
H	-3.81350100	7.60262600	-5.96444000
H	-3.14183800	8.17006300	-8.27116800
H	-2.73824100	6.36591600	-9.93431400
C	-4.22687600	5.10968300	-4.91602300
O	-3.93316300	3.90240500	-4.53778900
C	-4.94366600	6.01766600	-4.12501200
H	-5.45451800	6.83269000	-4.62579900

IM11

Ru	-3.37125200	2.95357100	-3.44488300
C	-5.52338800	2.54615900	-3.77596700
C	-5.28882900	2.53360700	-2.38537500
C	-4.29936200	1.65794100	-1.81213500
C	-3.57092400	0.83094200	-2.69120800
C	-3.81331600	0.85893500	-4.10375000
C	-4.80385100	1.68701700	-4.67951400
H	-6.20483100	3.28509100	-4.18446500
H	-5.80915500	3.24113700	-1.74994300
H	-2.75622800	0.22612000	-2.30729800
H	-3.16538400	0.27549100	-4.74760700
C	-5.12874600	1.70276900	-6.16302300
H	-5.44824800	2.72354000	-6.40722300
C	-6.32550000	0.75959600	-6.42078500
H	-6.06094600	-0.28085800	-6.19873700
H	-7.19428300	1.02635900	-5.80874800
H	-6.62410600	0.81518200	-7.47274000
C	-3.94325300	1.35698900	-7.07431200
H	-4.23929700	1.48363100	-8.12017700
H	-3.08594900	2.01225600	-6.88964600
H	-3.62244200	0.31504000	-6.95553700
C	-4.02841900	1.67902100	-0.33407900
H	-3.04153800	1.26905000	-0.10333700
H	-4.08696800	2.69738800	0.05925400
H	-4.77972200	1.07275800	0.18794400
C	-6.14151500	5.96801900	-0.13902500
C	-4.81991200	5.75596600	-0.52531100
C	-4.43493700	5.78656200	-1.87575300
C	-5.39539500	6.02702400	-2.88929000
C	-6.72389700	6.26235300	-2.46172800
C	-7.09817500	6.23087800	-1.12333300
H	-6.41503500	5.94905800	0.91159800
H	-4.05961100	5.58286100	0.23260800
H	-7.47587300	6.46946900	-3.21895900
H	-8.13245200	6.41635700	-0.84778200
C	-2.96681300	5.56448500	-2.10912200
O	-2.49035600	4.36789300	-2.11022800
C	-2.13629200	6.67548600	-2.15188300
H	-2.49081900	7.69776900	-2.14015600
S	-0.46215800	6.47170600	-2.45445900
O	0.00394400	5.96394500	-3.76717000
C	0.21697600	8.11672900	-2.14469500
H	-0.03161400	8.44438900	-1.13320500
H	1.29746100	8.05116000	-2.28930300
H	-0.21672100	8.78755100	-2.89020500
C	0.25173000	5.44298000	-1.14777700
H	1.32506800	5.36788200	-1.33717300
H	0.04258400	5.89431600	-0.17573100
H	-0.23690300	4.47206300	-1.24181700
C	-5.46623900	5.88566700	-8.70292300
C	-5.45489500	5.65504400	-7.32695400
C	-4.25340000	5.70105200	-6.59954200
C	-3.06289100	5.98878500	-7.28851700
C	-3.07681100	6.22426900	-8.66300600
C	-4.27665300	6.17062600	-9.37592400
H	-6.40453800	5.84018400	-9.24890000
H	-6.38423100	5.42949500	-6.81081800
H	-2.12650400	6.05758200	-6.74358600
H	-2.14831300	6.45726300	-9.17679400
H	-4.28469900	6.35177300	-10.44689300

C	-4.23422700	5.46134800	-5.12313100
O	-3.24650700	4.65985500	-4.68690000
C	-5.15666900	6.08077300	-4.33414900
H	-5.87767600	6.68964700	-4.87171200
C	0.81204000	2.73318300	-5.10799400
C	-0.63358100	3.08965400	-4.91609500
O	-1.26459300	2.68616900	-3.91583200
O	-1.15149700	3.83380900	-5.84968900
H	-2.06143300	4.19827000	-5.53193600
H	1.10326500	1.92286100	-4.43881300
H	1.41238500	3.62397300	-4.89112700
H	0.99531900	2.45715400	-6.15009000

TS5

Ru	-3.33805700	2.91354300	-3.34705900
C	-5.51890900	2.46211500	-3.59055800
C	-5.22044500	2.40930000	-2.21781600
C	-4.15781200	1.56921100	-1.72973900
C	-3.44625100	0.79704100	-2.67624100
C	-3.77575500	0.83526100	-4.07162000
C	-4.81342600	1.65788400	-4.55850600
H	-6.25174900	3.18180800	-3.94049900
H	-5.73125800	3.07687500	-1.53314300
H	-2.58372700	0.22261600	-2.35482900
H	-3.15255300	0.28605400	-4.76715300
C	-5.21272800	1.72950600	-6.02208400
H	-5.50758000	2.76858100	-6.21822400
C	-6.45814900	0.83907600	-6.23758000
H	-6.22119900	-0.21584400	-6.05753200
H	-7.28381900	1.11946600	-5.57404900
H	-6.80804800	0.93507200	-7.27052400
C	-4.09286600	1.36456900	-7.00592400
H	-4.43991700	1.53690300	-8.02938200
H	-3.19711200	1.97373700	-6.84939000
H	-3.81740100	0.30534000	-6.93534300
C	-3.80102800	1.55706900	-0.26968800
H	-2.78094700	1.19726100	-0.11108000
H	-3.89105900	2.55631200	0.16477400
H	-4.48459600	0.89055100	0.27161200
C	-6.28811500	5.90974700	-0.24836000
C	-4.94798500	5.71470400	-0.57584700
C	-4.50256700	5.77335000	-1.90598500
C	-5.42018600	6.01877600	-2.95636600
C	-6.76928300	6.23196600	-2.59033500
C	-7.20297400	6.17657600	-1.27031600
H	-6.60899300	5.87365200	0.78837100
H	-4.22093300	5.53480600	0.21220000
H	-7.48834000	6.44235700	-3.37799400
H	-8.25031400	6.34753700	-1.03868000
C	-3.02429600	5.57536200	-2.09141200
O	-2.53044700	4.38657200	-2.03770100
C	-2.21854500	6.70048000	-2.17836400
H	-2.60483300	7.71144800	-2.20403300
S	-0.55570700	6.55021000	-2.57695000
O	-0.16460700	6.20925800	-3.96562900
C	0.12725600	8.15866100	-2.11809000
H	-0.04822600	8.35292300	-1.05804800
H	1.19410700	8.13395800	-2.35068600
H	-0.36892100	8.90847900	-2.73901700
C	0.23909100	5.38656100	-1.44471600
H	1.31440700	5.44530400	-1.62971400
H	-0.01046200	5.64848500	-0.41472000
H	-0.15880600	4.40560700	-1.70739400
C	-5.21934300	6.02990200	-8.77711100
C	-5.28097500	5.76692600	-7.40928600
C	-4.11162500	5.75081300	-6.62935000
C	-2.87745600	6.00303300	-7.25321600
C	-2.82108800	6.26728100	-8.62103700
C	-3.98853200	6.28107300	-9.38717800
H	-6.13188600	6.03294300	-9.36655100
H	-6.23962700	5.56050600	-6.94139900
H	-1.96364900	6.00698300	-6.66814100
H	-1.86099000	6.46788200	-9.08772900
H	-3.93970000	6.48532300	-10.45286600
C	-4.18254400	5.49770700	-5.16444800
O	-3.22947600	4.65649900	-4.65950800
C	-5.11690400	6.10320600	-4.39166300
H	-5.80284500	6.73862700	-4.94396200
C	0.71225700	2.75936800	-5.11585100
C	-0.75175400	3.06799400	-4.91018800
O	-1.27179500	2.72575500	-3.79248000
O	-1.37652600	3.65827800	-5.83889700
H	-2.44359100	4.25709300	-5.34391900
H	1.08384100	2.05888500	-4.36616300
H	1.27243100	3.69892700	-5.04940100
H	0.86494900	2.35797700	-6.12150800

IM12

Ru	-3.33456700	2.91183500	-3.32333900
C	-5.51393600	2.43319300	-3.55749400

C	-5.20643300	2.37851700	-2.18740900
C	-4.12506200	1.55570400	-1.71141200
C	-3.40799100	0.79777900	-2.66697100
C	-3.74977400	0.83280900	-4.05943000
C	-4.80258200	1.64323200	-4.53350800
H	-6.26059400	3.14246700	-3.89965300
H	-5.72180700	3.03607500	-1.49637700
H	-2.53323200	0.23747800	-2.35379900
H	-3.12518800	0.29481100	-4.76228600
C	-5.21079700	1.72108400	-5.99441600
H	-5.48750500	2.76571200	-6.18892800
C	-6.47475300	0.85466800	-6.20027500
H	-6.25730400	-0.20409300	-6.01845000
H	-7.29175400	1.15279200	-5.53375600
H	-6.82772200	0.95449900	-7.23178400
C	-4.10486200	1.33299700	-6.98493700
H	-4.45323600	1.51617200	-8.00607600
H	-3.19388900	1.91959800	-6.83044300
H	-3.85603800	0.26688600	-6.91805500
C	-3.75473400	1.54487600	-0.25477100
H	-2.72771600	1.20062200	-0.10683600
H	-3.85595400	2.54131900	0.18362700
H	-4.42276900	0.86595300	0.29047700
C	-6.32969100	5.89710800	-0.26124300
C	-4.98669800	5.70666300	-0.57932900
C	-4.53091100	5.77899900	-1.90518000
C	-5.44142700	6.02995000	-2.95995100
C	-6.79391600	6.23680100	-2.60422900
C	-7.23742400	6.17011600	-1.28795800
H	-6.65874100	5.85227700	0.77259000
H	-4.26546800	5.52058100	0.21253200
H	-7.50731000	6.45116800	-3.39593300
H	-8.28682100	6.33694500	-1.06285200
C	-3.05185200	5.58446400	-2.08561500
O	-2.55721500	4.39701600	-2.00936700
C	-2.24791400	6.70776100	-2.19990700
H	-2.63601400	7.71767000	-2.24269600
S	-0.59095700	6.55622300	-2.62630500
O	-0.22656300	6.26114700	-4.03298200
C	0.11308800	8.14413100	-2.12900500
H	-0.03609900	8.30209200	-1.05892000
H	1.17393800	8.12185300	-2.38801600
H	-0.39290000	8.91825900	-2.71094900
C	0.21536700	5.35148400	-1.54679000
H	1.29101300	5.44902000	-1.71341200
H	-0.05436700	5.55075200	-0.50809100
H	-0.15253700	4.37586100	-1.86730400
C	-5.17069900	6.10684100	-8.78370700
C	-5.25709900	5.83696700	-7.41884500
C	-4.09774300	5.78257900	-6.62568900
C	-2.84849700	6.00129600	-7.23240000
C	-2.76797900	6.27123100	-8.59778200
C	-3.92535100	6.32476600	-9.37721700
H	-6.07526200	6.13977200	-9.38432600
H	-6.22663100	5.65309100	-6.96432400
H	-1.94207800	5.97554200	-6.63634700
H	-1.79677500	6.44504300	-9.05190600
H	-3.85762700	6.53344300	-10.44099200
C	-4.19257300	5.52341400	-5.16544400
O	-3.24713000	4.66850100	-4.65227000
C	-5.12564900	6.12665300	-4.39246200
H	-5.80082000	6.77406000	-4.94407200
C	0.70735200	2.78070500	-5.08820500
C	-0.76982400	3.05145500	-4.90656500
O	-1.26782400	2.76812700	-3.75457400
O	-1.41909400	3.54946300	-5.86248300
H	-2.53906700	4.26798600	-5.33576800
H	1.09728800	2.12496600	-4.30763300
H	1.23708600	3.73975300	-5.05669200
H	0.88081500	2.34308500	-6.07500000

IM13

C	-3.46888900	7.56152200	-2.54341400
C	-3.52765700	6.24636500	-2.09623600
C	-3.32268500	5.15962100	-2.96418300
C	-3.04417600	5.39094400	-4.33657100
C	-3.03120100	6.73616300	-4.76460600
C	-3.22816900	7.80385600	-3.89662900
H	-3.61809400	8.38327500	-1.84864900
H	-3.72917300	6.04158500	-1.04862800
H	-2.84459300	6.93271600	-5.81721200
H	-3.19040500	8.82187800	-4.27566900
C	-3.45545300	3.78627500	-2.37539800
O	-2.55898900	2.90658000	-2.54741600
C	-4.60321700	3.52587600	-1.60429500
S	-4.80901400	1.98410800	-0.92016800
O	-6.06485700	1.83557100	-0.15036900
C	-4.70511800	0.72059000	-2.21414200
H	-3.80419500	0.92357000	-2.79691000

H	-4.68323400	-0.26498300	-1.74315200
H	-5.60517700	0.83961800	-2.82066900
C	-3.37390800	1.58676400	0.11024800
H	-3.44949100	0.54372300	0.42645000
H	-2.47994500	1.77556800	-0.48709100
H	-3.42778900	2.25636400	0.97088900
C	-2.48033000	1.92969300	-9.02260800
C	-2.70709300	2.62432600	-7.83689500
C	-1.72664800	2.67616300	-6.83060000
C	-0.52004600	1.98855700	-7.04567600
C	-0.29204100	1.29863700	-8.23562400
C	-1.26868400	1.26648600	-9.23156200
H	-3.25700900	1.89910900	-9.78261500
H	-3.66683200	3.10849900	-7.68537800
H	0.23541800	2.00622700	-6.26889200
H	0.65298400	0.78168700	-8.38274200
H	-1.09285300	0.72474100	-10.15723200
C	-1.91962500	3.41655900	-5.55356100
O	-1.02286400	3.06031200	-4.60875700
C	-2.85678800	4.39049200	-5.41338600
H	-3.42406200	4.62485700	-6.30877500
H	-1.44849400	3.17546600	-3.71474500
H	-5.45659500	4.17466200	-1.46520600

TS6

C	-7.42580100	5.60177600	-2.54452800
C	-6.10143600	5.58960600	-2.11484300
C	-5.04194800	5.34931000	-3.00342700
C	-5.32104900	5.14037200	-4.37417600
C	-6.66236600	5.16250800	-4.78873500
C	-7.70611800	5.37956500	-3.89211800
H	-8.22850100	5.77567800	-1.83307300
H	-5.87434800	5.73387000	-1.06232900
H	-6.88262800	5.01883100	-5.84271600
H	-8.73296400	5.38179900	-4.24847700
C	-3.64758700	5.24615700	-2.43914700
O	-2.88173100	4.32066400	-2.81453500
C	-3.27499300	6.21685400	-1.48013500
H	-3.82736700	7.10338600	-1.20067800
S	-1.69571700	6.19480300	-0.86914200
O	-1.40952800	7.27373300	0.10504000
C	-1.36657600	4.56828500	-0.14560400
H	-1.66920400	3.82419300	-0.88532700
H	-0.30489200	4.50186700	0.10391000
H	-1.98070100	4.50900900	0.75511700
C	-0.49371800	6.24171800	-2.22540300
H	0.50861600	6.12171400	-1.80733200
H	-0.75615700	5.44349400	-2.92155800
H	-0.60979600	7.21824600	-2.69865900
C	-0.68376500	2.38140900	-6.12858000
C	-1.77374900	3.11766300	-5.67080000
C	-1.79328300	4.51434800	-5.81745000
C	-0.69552800	5.16123500	-6.41057100
C	0.38816100	4.42025000	-6.87477300
C	0.39442900	3.02947000	-6.73697100
H	-0.67023400	1.30231300	-6.00334100
H	-2.59148200	2.62743000	-5.15422500
H	-0.71513300	6.24084200	-6.51536300
H	1.22636800	4.92490600	-7.34731700
H	1.24146900	2.45200000	-7.09799400
C	-2.94334700	5.30892100	-5.34590600
O	-2.66525700	6.54092700	-5.01502500
C	-4.27890100	4.84360600	-5.42392400
H	-4.66827900	5.67462000	-6.43850500
H	-4.35777200	3.82919200	-5.81631500
C	-6.02359300	8.75680500	-7.41113600
H	-5.75783600	9.76536300	-7.09016600
H	-7.07059100	8.56345800	-7.14984600
H	-5.93312900	8.66196800	-8.49665000
C	-5.14942200	7.72953800	-6.71978500
O	-5.16414800	6.55078800	-7.23321000
O	-4.49915700	8.07603900	-5.70191200
H	-3.50365200	7.17847500	-5.17139200

TS7

Ru	-3.45749900	2.46186300	-3.73840700
C	-3.66686300	0.23443800	-3.77702900
C	-2.68452900	0.55317900	-2.82222700
C	-1.54343000	1.34818400	-3.18723400
C	-1.45868600	1.81929100	-4.51663800
C	-2.46785900	1.48630800	-5.47669200
C	-3.57412800	0.67015000	-5.14508700
H	-4.56427700	-0.28307500	-3.45373000
H	-2.82225800	0.25437000	-1.78868400
H	-0.66415200	2.50344500	-4.79360500
H	-2.41409200	1.93611100	-6.46082400
C	-4.63380900	0.24035200	-6.14464600
H	-5.58525200	0.20288800	-5.59742200
C	-4.31395900	-1.19488900	-6.62098200
H	-3.36698800	-1.22230800	-7.17229400

H	-4.23911500	-1.89674400	-5.78304600
H	-5.10465900	-1.55060100	-7.28953900
C	-4.80928800	1.19419000	-7.33379700
H	-5.64442100	0.85304900	-7.95369500
H	-5.03153000	2.21380700	-7.00532600
H	-3.92140300	1.21200300	-7.97705100
C	-0.49817700	1.70483900	-2.17179200
H	-0.90950500	1.67783300	-1.15889600
H	0.31730200	0.97143300	-2.21950500
H	-0.08346900	2.69694600	-2.36684000
C	-7.03055500	5.38555400	-0.53281200
C	-5.67358100	5.11474600	-0.67898100
C	-5.04112000	5.19272400	-1.93071900
C	-5.77624200	5.54384600	-3.08024300
C	-7.14831800	5.82453900	-2.90176600
C	-7.76894600	5.75334100	-1.65927000
H	-7.49913800	5.32315600	0.44459500
H	-5.08097400	4.84748200	0.19229700
H	-7.73396200	6.10354800	-3.77410700
H	-8.82623800	5.98712400	-1.57079800
C	-3.56573000	4.91648300	-1.90219500
O	-3.09916400	3.72739400	-2.02563100
C	-2.74351700	5.99340600	-1.58054100
H	-3.10030100	7.00629800	-1.44839200
S	-1.05706900	5.77029200	-1.40360000
O	-0.25445400	5.19453500	-2.50694400
C	-0.45806900	7.41889200	-0.96790800
H	-0.99009200	7.79603100	-0.09203300
H	0.61302100	7.32994400	-0.77395700
H	-0.62822900	8.05959100	-1.83621700
C	-0.76025400	4.79788500	0.09805800
H	0.31617800	4.63735500	0.19435200
H	-1.16772900	5.33206700	0.95889100
H	-1.28521100	3.85330400	-0.05081600
C	-3.27898500	8.24967100	-7.36343400
C	-3.79155100	7.50536900	-6.30195500
C	-3.48751000	6.13922700	-6.17372800
C	-2.64247400	5.54423700	-7.12618400
C	-2.13770500	6.28534800	-8.19074800
C	-2.45615800	7.64097900	-8.31333300
H	-3.51929500	9.30564700	-7.44682600
H	-4.41131100	7.99524500	-5.55778800
H	-2.39526300	4.49379200	-7.02254200
H	-1.49743100	5.80868600	-8.92765500
H	-2.06154400	8.22027900	-9.14323500
C	-4.00941300	5.32238300	-5.03457600
O	-3.29660400	4.29400400	-4.75385400
C	-5.27613200	5.64961600	-4.47926700
H	-5.76436900	6.46701500	-5.00513500
C	-7.88556400	2.00248300	-3.85900000
C	-6.58111100	2.69815900	-4.17337500
O	-5.58939600	2.47160000	-3.42632800
O	-6.59101500	3.50003800	-5.17658100
H	-5.90798300	4.46619300	-4.99581800
H	-7.73112400	1.15955800	-3.18373700
H	-8.55496800	2.72483500	-3.37818400
H	-8.36838600	1.67461400	-4.78392600

TS8

Ru	-4.42579700	2.91941800	-4.00859500
C	-4.71173800	1.19420400	-5.40537900
C	-5.93074000	1.43031900	-4.72852100
C	-6.01371700	1.40921900	-3.29213900
C	-4.82522100	1.17007400	-2.57114100
C	-3.59076700	0.99339800	-3.26174800
C	-3.49662300	0.95726600	-4.68391000
H	-4.68822000	1.23945800	-6.48999400
H	-6.81883000	1.67495700	-5.30249500
H	-4.82586600	1.28823800	-1.49399200
H	-2.68137200	0.92441200	-2.67518800
C	-2.21127900	0.62445300	-5.42016300
H	-2.27465800	1.10588800	-6.40460200
C	-2.14582600	-0.90296100	-5.64788400
H	-2.08362700	-1.43983300	-4.69420200
H	-3.02512400	-1.27141900	-6.18717400
H	-1.25758700	-1.15609900	-6.23610500
C	-0.93790200	1.13707800	-4.73255800
H	-0.07218900	0.94318300	-5.37340800
H	-0.98653600	2.21554700	-4.54979700
H	-0.75207600	0.62651800	-3.78021900
C	-7.30470200	1.70572900	-2.58143000
H	-7.96133900	2.33238600	-3.19194400
H	-7.83705600	0.76974800	-2.36839200
H	-7.10597300	2.21937800	-1.63738700
C	-7.69115100	5.79398700	-3.39094900
C	-6.53400700	5.65655600	-2.64645500
C	-5.33085600	5.23739900	-3.25879000
C	-5.29105800	4.99196200	-4.67858700
C	-6.51988700	5.11189000	-5.39185700

C	-7.68575600	5.51027800	-4.77186900
H	-8.60576400	6.12440400	-2.90810000
H	-6.54074300	5.86268900	-1.58141700
H	-6.50628800	4.92933100	-6.46285200
H	-8.59621600	5.62471700	-5.35280500
C	-4.08442800	5.11556300	-2.43960800
O	-3.29455200	4.17937100	-2.68425900
C	-3.74294600	6.27078200	-1.53866900
H	-3.69800000	7.20037400	-2.11524500
S	-2.16113900	6.19829500	-0.67436600
O	-1.83643700	7.50759300	-0.08439000
C	-2.34449100	4.89901300	0.55099000
H	-2.75152800	4.01201200	0.05882300
H	-1.36706200	4.73312000	1.00953200
H	-3.07102200	5.28354600	1.27229000
C	-0.86966500	5.65473500	-1.80883900
H	0.07490700	5.86879300	-1.30202800
H	-1.00239000	4.59469900	-2.02266300
H	-0.98123700	6.23632700	-2.72733800
C	-0.68650300	3.93784300	-8.10211100
C	-1.72804800	4.07930300	-7.18657500
C	-1.73147900	5.14188900	-6.26628000
C	-0.66967600	6.06299000	-6.29339500
C	0.36726900	5.92512300	-7.21157400
C	0.36221300	4.86070100	-8.11780000
H	-0.69472800	3.11209700	-8.80783000
H	-2.53269100	3.35166400	-7.18762400
H	-0.68610000	6.89391600	-5.59637700
H	1.17585200	6.65004900	-7.22805100
H	1.16902300	4.75424200	-8.83722000
C	-2.82204700	5.36073000	-5.26560100
O	-2.68199900	6.21655500	-4.38144100
C	-4.07411800	4.56397900	-5.38780100
H	-4.27663900	4.26601500	-6.41421000
C	-6.40060700	3.54912700	1.34948900
H	-7.40845100	3.37592800	0.95122300
H	-6.49941600	4.04663400	2.31672500
H	-5.92314700	2.57198900	1.47258500
C	-5.61658200	4.42124900	0.37279000
O	-5.11013800	3.84854000	-0.65413600
O	-5.52204900	5.65553000	0.61479300
H	-4.49389200	6.31867100	-0.70269000

IM14

Ru	-4.75405000	2.64366900	-3.86190100
C	-3.94507600	1.51871900	-5.58336300
C	-5.36159900	1.35439600	-5.60001700
C	-6.02818700	0.85555000	-4.45991200
C	-5.23695700	0.37716200	-3.35443200
C	-3.84942300	0.50981300	-3.34958500
C	-3.16841700	1.13691700	-4.45278900
H	-3.45101600	1.96676200	-6.43766100
H	-5.93771100	1.69615800	-6.45304900
H	-5.74540300	-0.01559000	-2.47930100
H	-3.28810600	0.19163400	-2.47854900
C	-1.65184500	1.23536600	-4.52495000
H	-1.41775900	2.16522100	-5.05833900
C	-1.10186100	0.06252700	-5.36748300
H	-1.33207200	-0.90139000	-4.89841400
H	-1.52532700	0.05788600	-6.37691700
H	-0.01313500	0.14147600	-5.45889300
C	-0.96241100	1.27804900	-3.15400800
H	0.10268100	1.49942700	-3.28032000
H	-1.40144900	2.04545500	-2.50859800
H	-1.02813900	0.31467100	-2.63464500
C	-7.52987300	0.77779400	-4.39290300
H	-7.99509700	1.33068700	-5.21320600
H	-7.85660900	-0.26771700	-4.45660400
H	-7.89552100	1.19222400	-3.44870700
C	-7.11414300	7.45815900	-2.82612600
C	-6.15811300	6.70250700	-2.15161500
C	-5.39103200	5.73464300	-2.81953500
C	-5.58462400	5.51909900	-4.19378800
C	-6.56684100	6.27746100	-4.85343100
C	-7.32337300	7.23818400	-4.18792700
H	-7.69592000	8.20170600	-2.28975100
H	-6.03628500	6.85135900	-1.08233500
H	-6.72701000	6.11130500	-5.91587800
H	-8.07068700	7.81157100	-4.72874600
C	-4.39092700	4.90076300	-1.99972400
O	-3.70941000	3.88844800	-2.53022200
C	-3.43434000	5.91048500	-1.31715300
H	-3.05074200	6.58437700	-2.08824500
S	-1.92927500	5.21740400	-0.56223400
O	-1.34973500	6.13477600	0.43705900
C	-2.32041200	3.61490100	0.15883900
H	-2.72998100	2.98339000	-0.62980100
H	-1.39131800	3.23103400	0.58774300
H	-3.05900500	3.79760100	0.94187200

C	-0.81812500	4.90722000	-1.94041000
H	-0.00383800	4.27124600	-1.58667900
H	-1.40748800	4.46392500	-2.74523400
H	-0.43892300	5.88860500	-2.23732500
C	-2.73813000	4.02983200	-8.91071700
C	-3.41893500	4.28303200	-7.71831100
C	-2.71774000	4.63017800	-6.55074100
C	-1.31703900	4.73350400	-6.61961800
C	-0.63535300	4.46792000	-7.80442800
C	-1.34522900	4.11306700	-8.95539800
H	-3.29758600	3.77848200	-9.80733200
H	-4.50316900	4.23578200	-7.71758700
H	-0.78061400	5.04384700	-5.72922000
H	0.44761500	4.54844400	-7.83666300
H	-0.81702700	3.91524100	-9.88370100
C	-3.38182600	4.97194600	-5.23892300
O	-2.73666100	5.63271900	-4.41785400
C	-4.78940200	4.52217300	-4.97212300
H	-5.30582200	4.28905100	-5.90276100
C	-7.19094600	3.24168600	-0.05074500
H	-6.67666100	3.19800800	0.91249400
H	-7.71034400	2.30354200	-0.25280900
H	-7.93496300	4.04580400	0.00379600
C	-6.22872900	3.55612100	-1.16515800
O	-5.24497000	4.36833700	-0.80497100
O	-6.38705900	3.11676200	-2.30997000
H	-3.89330800	6.48054000	-0.50676600

TS9

Ru	-4.69439800	2.61712600	-3.83553600
C	-3.82779100	1.47776300	-5.56982200
C	-5.24121800	1.27506000	-5.54686200
C	-5.87017600	0.74676100	-4.39893400
C	-5.04324400	0.34321100	-3.29148400
C	-3.66207900	0.54315800	-3.31633400
C	-3.01859300	1.14366800	-4.45663100
H	-3.36943800	1.92050300	-6.44637300
H	-5.84434000	1.57833800	-6.39586800
H	-5.51663900	-0.05447300	-2.39931200
H	-3.07397500	0.27207200	-2.44658100
C	-1.50646600	1.27265900	-4.57257600
H	-1.30758400	2.17701400	-5.16125200
C	-0.95059800	0.06646900	-5.36250800
H	-1.14567600	-0.87409100	-4.83378900
H	-1.40137100	-0.00479700	-6.35745900
H	0.13316700	0.16385700	-5.48885700
C	-0.78090600	1.40879100	-3.22712400
H	0.27877700	1.62668300	-3.39681300
H	-1.20549900	2.21943400	-2.62650700
H	-0.82714900	0.48330900	-2.64109300
C	-7.36428300	0.59941700	-4.31235800
H	-7.85504100	0.96906000	-5.21651700
H	-7.63419600	-0.45540500	-4.18202700
H	-7.74830900	1.16431400	-3.45729900
C	-6.94375800	7.55587300	-2.78932900
C	-5.95726100	6.81337800	-2.15066100
C	-5.26927700	5.78699500	-2.82376100
C	-5.57374000	5.50566300	-4.17211200
C	-6.57888000	6.26064200	-4.79373200
C	-7.26074900	7.27080600	-4.11919800
H	-7.46849700	8.33886900	-2.25098900
H	-5.75853200	7.01420900	-1.10305300
H	-6.81873300	6.05472700	-5.83356400
H	-8.03490600	7.83599000	-4.63020000
C	-4.24964400	4.98182700	-2.09542700
O	-3.65263300	3.95365200	-2.52788200
C	-3.50661800	5.77973300	-1.01488900
H	-3.12845300	6.72573500	-1.41741600
S	-2.04024800	4.97223000	-0.32717900
O	-1.52776100	5.71086200	0.84039600
C	-2.49139900	3.28386100	0.10620600
H	-2.67977700	2.72215600	-0.80702900
H	-1.65891100	2.89250500	0.69650300
H	-3.40545000	3.35226600	0.70112300
C	-0.84857000	4.92613500	-1.67847700
H	-0.07964300	4.19098800	-1.43166000
H	-1.37429000	4.70020500	-2.60993600
H	-0.41540700	5.93014400	-1.70432200
C	-3.01670100	4.00392300	-9.01634100
C	-3.62370100	4.25361100	-7.78407900
C	-2.85049000	4.58076000	-6.65704100
C	-1.45479200	4.66863100	-6.80589000
C	-0.84723200	4.40696400	-8.03113500
C	-1.62777200	4.07149100	-9.14150400
H	-3.63044100	3.76686800	-9.88072400
H	-4.70633900	4.21687600	-7.71995600
H	-0.86401700	4.96190800	-5.94451300
H	0.23286700	4.47494600	-8.12628900
H	-1.15725400	3.87584500	-10.10078500

C	-3.43006600	4.91074000	-5.30361600
O	-2.72704000	5.53963300	-4.50559700
C	-4.83288900	4.47898100	-4.96399900
H	-5.39791200	4.23404000	-5.86188600
C	-7.76755400	3.24535000	-0.62996300
H	-7.59834200	3.23132800	0.44907700
H	-8.23334500	2.31285400	-0.95662600
H	-8.45905700	4.06659600	-0.85483500
C	-6.46744100	3.49809200	-1.37149500
O	-5.55907700	4.13710700	-0.74951200
O	-6.40815900	3.09917300	-2.57690800
H	-4.13318700	5.97806300	-0.14343200

IM15

Ru	-4.43036900	2.50982600	-3.98405000
C	-3.48766800	1.47784500	-5.78615200
C	-4.87598000	1.13903900	-5.70872600
C	-5.40575300	0.52801100	-4.55140900
C	-4.50657500	0.23335000	-3.46610900
C	-3.15635800	0.59218000	-3.53855400
C	-2.61134400	1.23345200	-4.70732300
H	-3.10904900	1.95991000	-6.67975500
H	-5.53372800	1.37816500	-6.53752400
H	-4.90108700	-0.20715500	-2.55634000
H	-2.51880800	0.41404400	-2.67968700
C	-1.12892300	1.54195200	-4.85452000
H	-1.04646500	2.32313000	-5.62059400
C	-0.39795100	0.28591200	-5.37765800
H	-0.45723300	-0.53608600	-4.65415800
H	-0.82661500	-0.06447100	-6.32244900
H	0.66109600	0.50822300	-5.54733800
C	-0.46057400	2.07378900	-3.57742800
H	0.57815600	2.34614900	-3.79111700
H	-0.98131100	2.96124800	-3.20510000
H	-0.43593400	1.31673000	-2.78332700
C	-6.86776000	0.19826200	-4.42988400
H	-7.43534700	0.58536300	-5.28007200
H	-7.00659600	-0.88904200	-4.39081200
H	-7.28321300	0.63231800	-3.51581900
C	-6.39241400	7.48279000	-2.36213400
C	-5.19728100	6.84682200	-2.05860700
C	-4.72115000	5.77924700	-2.85014200
C	-5.42782400	5.39299300	-4.02223900
C	-6.63324400	6.04974400	-4.30208800
C	-7.11947800	7.06728100	-3.48397700
H	-6.75490800	8.29347700	-1.73840900
H	-4.64787100	7.17395800	-1.18160200
H	-7.18707100	5.76807800	-5.19310200
H	-8.05678300	7.55636600	-3.73342800
C	-3.56045200	5.00583500	-2.43693900
O	-3.33261200	3.81440800	-2.71609300
C	-2.51897200	5.61304900	-1.49681800
H	-1.52268300	5.42714100	-1.91063200
S	-2.42138900	4.86605700	0.17802900
O	-1.27579000	5.43854500	0.90641700
C	-3.99755600	5.14917800	0.99343300
H	-3.87045500	4.77899500	2.01505300
H	-4.17911200	6.22582900	1.01006900
H	-4.76884200	4.57798700	0.45352600
C	-2.33927500	3.08154800	-0.01293700
H	-2.17642700	2.69122100	0.99616300
H	-3.29259600	2.75007300	-0.44391200
H	-1.48730800	2.85552600	-0.65634700
C	-3.82974500	4.02084700	-9.30155200
C	-4.19757600	4.22692400	-7.97107400
C	-3.24051700	4.60982800	-7.01591200
C	-1.91043600	4.79761600	-7.43308600
C	-1.54216900	4.58380700	-8.75878400
C	-2.50152700	4.19202000	-9.69735000
H	-4.58283100	3.73557500	-10.03056500
H	-5.23958900	4.10556300	-7.69425200
H	-1.18190100	5.12286500	-6.69811000
H	-0.51001200	4.72984000	-9.06436800
H	-2.21742700	4.03065300	-10.73330200
C	-3.55591000	4.87598900	-5.56788000
O	-2.71786100	5.47207700	-4.88233000
C	-4.86465900	4.41364200	-4.98699800
H	-5.60314900	4.18834400	-5.75318900
C	-7.68903800	3.37867300	-1.11415500
H	-7.79937400	3.02420600	-0.08634900
H	-8.44042900	2.91666600	-1.75786300
H	-7.86136700	4.46207100	-1.12313800
C	-6.27760200	3.10397900	-1.61818400
O	-5.32254800	3.10900700	-0.81098700
O	-6.19836100	2.92086800	-2.89064400
H	-2.61918600	6.68377600	-1.31086700

TS10

Ru	-4.77553100	2.47383800	-3.51315000
C	-4.10438100	1.18351500	-5.26373500

C	-5.52434600	1.06779700	-5.12089000
C	-6.09546400	0.64427900	-3.90319300
C	-5.20835200	0.35648800	-2.80822400
C	-3.81437800	0.48154100	-2.95664500
C	-3.22118000	0.87148100	-4.20588200
H	-3.69799500	1.53963600	-6.20472900
H	-6.17114300	1.34072500	-5.94799500
H	-5.62443200	0.10213300	-1.83939000
H	-3.18122400	0.30726000	-2.09396600
C	-1.71786400	0.88452000	-4.42841700
H	-1.53124100	1.53587200	-5.29095100
C	-1.24530500	-0.53757800	-4.80145800
H	-1.41822100	-1.24050800	-3.97781900
H	-1.77007800	-0.91668900	-5.68468900
H	-0.17191700	-0.53337100	-5.01969500
C	-0.91382400	1.44108200	-3.24321900
H	0.14376500	1.51865100	-3.51640800
H	-1.27696300	2.43354100	-2.95911800
H	-0.97007400	0.78215000	-2.36775900
C	-7.58370100	0.56078600	-3.71256000
H	-8.11953800	0.90909600	-4.59935200
H	-7.88129900	-0.47603300	-3.51536000
H	-7.89082800	1.17667000	-2.86215800
C	-6.25072100	7.55166800	-2.12138200
C	-5.05771600	6.87534500	-1.86098100
C	-4.66658800	5.80142000	-2.66590800
C	-5.44027000	5.44323500	-3.78656400
C	-6.63634600	6.12176200	-4.03728800
C	-7.04583500	7.16093700	-3.20204500
H	-6.55270000	8.38100000	-1.48930400
H	-4.44561300	7.20280600	-1.02644900
H	-7.23657200	5.84858100	-4.90097300
H	-7.97466200	7.68461100	-3.40865100
C	-3.45770400	4.95123800	-2.43212400
O	-3.51998700	3.67188000	-2.32619500
C	-2.32146000	5.52314600	-1.57244400
H	-1.35794100	5.20040200	-1.97623000
S	-2.24846000	4.91569700	0.16089300
O	-1.03769800	5.46727300	0.79747400
C	-3.75769800	5.36754700	1.02627700
H	-3.59269600	5.05223200	2.06112800
H	-3.86856800	6.45197000	0.98516900
H	-4.59821800	4.81548200	0.58059000
C	-2.30472200	3.11826200	0.17742000
H	-2.06057000	2.84583700	1.20891900
H	-3.31612400	2.81570700	-0.11674100
H	-1.54523200	2.74732200	-0.51131400
C	-2.93839600	3.86084200	-8.65026600
C	-3.57594300	4.12607000	-7.43971800
C	-2.82566000	4.30985800	-6.26309300
C	-1.42100700	4.24107800	-6.33740300
C	-0.78761300	3.97371700	-7.54787300
C	-1.54452600	3.78006100	-8.70727700
H	-3.52886400	3.73176000	-9.55243800
H	-4.65658000	4.21794200	-7.42313900
H	-0.83938400	4.40429300	-5.43720700
H	0.29636100	3.92251200	-7.59056500
H	-1.05012500	3.57782900	-9.65291300
C	-3.46422300	4.63181600	-4.96441800
O	-2.69022600	5.14044300	-4.07011500
C	-4.87332000	4.42965800	-4.69538800
H	-5.48154700	4.20549500	-5.56548600
C	-7.72650900	3.92964100	-0.58326300
H	-7.81455000	3.73491300	0.48780900
H	-8.57519600	3.49433500	-1.11704500
H	-7.75685400	5.01275900	-0.75090300
C	-6.40394200	3.38422700	-1.11211600
O	-5.45942800	3.17037800	-0.32958900
O	-6.39668500	3.21764900	-2.39672600
H	-2.31179300	6.61171000	-1.47636100

IM16

Ru	-0.14424100	-0.31153400	3.33857400
C	-1.17435200	-1.92810400	2.20217000
C	-1.91980400	-1.63486300	3.36303700
C	-1.33110000	-1.72121900	4.67452200
C	0.03642900	-2.05694500	4.74657400
C	0.80395700	-2.31551400	3.56032500
C	0.21547400	-2.28735500	2.27508900
H	-1.63583400	-1.78338600	1.23192500
H	-2.93783000	-1.27419100	3.25848400
H	0.54215300	-2.03854800	5.70658500
H	1.87063800	-2.47747900	3.66179400
C	0.98254900	-2.54481800	0.99185200
H	0.58941900	-1.83419800	0.25403100
C	0.67673800	-3.97472300	0.49439600
H	1.04891400	-4.72571000	1.20103100
H	-0.39830900	-4.13921000	0.36178600
H	1.16516100	-4.14778800	-0.47042600

C	2.49393600	-2.30774700	1.10625600
H	2.95436900	-2.40091100	0.11752100
H	2.71213000	-1.30476600	1.48810900
H	2.97906300	-3.04420100	1.75804000
C	-2.13862500	-1.38848500	5.89884200
H	-2.74907600	-0.49502900	5.73590600
H	-2.81436100	-2.21908700	6.13794000
H	-1.49609600	-1.21574100	6.76632900
C	-4.04321000	1.02404400	1.25680000
C	-2.75052700	1.50519900	1.47940700
C	-1.71498700	1.20504000	0.58877000
C	-1.98119300	0.41803300	-0.55417900
C	-3.28315300	-0.07826300	-0.75334200
C	-4.30586800	0.22224000	0.14167000
H	-4.83872200	1.27264400	1.95329400
H	-2.55776600	2.08924400	2.37380100
H	-3.48786500	-0.68470000	-1.63209000
H	-5.30829400	-0.15730100	-0.03448700
C	-0.26400500	1.61024500	0.85744000
O	0.44519800	0.78088300	1.64755500
C	-0.23060000	3.07943500	1.39351900
H	-0.79210100	3.78747500	0.77610800
S	1.42003200	3.83818200	1.54239300
O	1.41244700	4.91716300	2.55377700
C	2.67301300	2.60097100	1.91195200
H	2.64528400	1.83033200	1.14369800
H	3.61814500	3.15092500	1.94283300
H	2.42397900	2.16180600	2.88135900
C	1.84632500	4.47221100	-0.08977600
H	2.84677100	4.90482300	-0.01231800
H	1.79892000	3.65224800	-0.80808300
H	1.11816700	5.25237900	-0.32508200
C	2.12481900	0.43404100	-4.68817800
C	1.08438000	0.53161700	-3.76710100
C	1.35152900	0.76625300	-2.40624300
C	2.68928300	0.91701600	-2.00090300
C	3.72911600	0.82158700	-2.92487600
C	3.45082800	0.57926000	-4.27162600
H	1.89958900	0.25775400	-5.73597400
H	0.05561100	0.45192800	-4.10477000
H	2.91357300	1.07340200	-0.95074400
H	4.75823800	0.92748100	-2.59281000
H	4.26024800	0.50755900	-4.99212100
C	0.24852800	0.84623800	-1.43159500
O	0.47009700	1.74792600	-0.41859900
C	-0.91634300	0.16538600	-1.50442100
H	-1.07511800	-0.54060500	-2.31143900
C	0.46742800	3.04972700	5.53648400
H	-0.45571600	3.32473900	6.05165400
H	0.76093800	3.88518500	4.88853600
H	1.27036300	2.88314900	6.25860500
C	0.25278500	1.81364300	4.70882700
O	-0.89951100	1.52455500	4.23549100
O	1.21449600	1.02446500	4.42747400
H	-0.60267500	3.10541300	2.41984000

TS11

Ru	0.16101000	-0.76723700	2.98530600
C	0.69116800	-2.12321400	1.26231400
C	-0.72564800	-2.16783200	1.46932100
C	-1.27529600	-2.49552700	2.72860100
C	-0.35396000	-2.75139700	3.80308000
C	1.04348000	-2.70437500	3.59784000
C	1.60149300	-2.40255200	2.30637000
H	1.06513800	-1.79516600	0.29863200
H	-1.38676600	-1.87741200	0.66023500
H	-0.73981100	-2.91133700	4.80468800
H	1.70097200	-2.82496300	4.45074400
C	3.09968700	-2.36308800	2.06045100
H	3.25811900	-1.69166200	1.20624200
C	3.57718600	-3.77333900	1.64746400
H	3.42487000	-4.49401000	2.45934000
H	3.04510300	-4.13952900	0.76304400
H	4.64729200	-3.75046700	1.41661800
C	3.91662300	-1.82441000	3.24625700
H	4.96081500	-1.70030200	2.94181700
H	3.54099400	-0.85806900	3.59646800
H	3.91358900	-2.52159400	4.09236000
C	-2.75919100	-2.51158200	2.96960100
H	-3.28981800	-1.91071700	2.22641600
H	-3.13221900	-3.54166200	2.90452300
H	-3.00350600	-2.12880600	3.96475700
C	-4.07864100	2.47207700	1.97345200
C	-2.75074700	2.06635000	2.02761100
C	-2.08594000	1.70975300	0.84371000
C	-2.75846200	1.74723400	-0.40198300
C	-4.09891400	2.17558400	-0.43505900
C	-4.74772300	2.53496300	0.73929200
H	-4.60023400	2.74406000	2.88608000

H	-2.22035200	2.00757500	2.97209000
H	-4.62261300	2.21512800	-1.38613600
H	-5.78259900	2.86252100	0.70328700
C	-0.68079100	1.30978100	0.86511500
O	-0.03005200	1.09673700	1.93390900
C	0.05881100	3.51301900	0.21493800
H	0.05076400	3.50125300	-0.87663100
S	1.60580000	3.97915000	0.76246300
O	2.28034300	5.23757500	0.34974600
C	1.48934600	3.91197100	2.56103500
H	1.18281900	2.90854600	2.86191400
H	2.46995200	4.18128200	2.95960600
H	0.75232900	4.66216400	2.85848100
C	2.68028600	2.58277200	0.36708800
H	3.68163400	2.82532500	0.72966300
H	2.28011100	1.68136000	0.83489600
H	2.69416000	2.49060900	-0.72150400
C	0.17541500	-0.68021600	-4.83122300
C	-0.59316100	-0.14700600	-3.80072300
C	-0.00508100	0.18668900	-2.56617600
C	1.37134700	-0.04989400	-2.39238700
C	2.13615600	-0.58897500	-3.42588900
C	1.54330900	-0.90307500	-4.64983300
H	-0.29680400	-0.93221600	-5.77607500
H	-1.65887400	-0.00867600	-3.95226900
H	1.84125300	0.20178600	-1.44817700
H	3.19894300	-0.75769300	-3.27698900
H	2.13959500	-1.32150600	-5.45510100
C	-0.81187200	0.76543900	-1.48161500
O	-0.19905600	0.66235400	-0.24566800
C	-2.04420400	1.32220800	-1.57819300
H	-2.51456400	1.41854200	-2.54930100
C	0.38473200	1.92970300	5.96297600
H	-0.24271000	1.63333500	6.80821000
H	-0.00797200	2.87853400	5.57928100
H	1.41560200	2.07525500	6.29111300
C	0.31590500	0.88766800	4.88608600
O	-0.79387100	0.35563900	4.54587500
O	1.35631700	0.50127300	4.25452900
H	-0.68567000	4.19796400	0.62389600

IM17

Ru	-0.25263000	-0.86865000	3.49787100
C	-0.72911000	-2.75572000	2.36074000
C	-1.56286400	-2.68253900	3.49154700
C	-1.00640400	-2.49958900	4.80542900
C	0.40739100	-2.41423000	4.90977100
C	1.25106000	-2.51648900	3.75609800
C	0.70602800	-2.68455900	2.46544000
H	-1.18169800	-2.80062700	1.37506900
H	-2.64055400	-2.67201000	3.36376700
H	0.85637100	-2.19695800	5.87336000
H	2.31724400	-2.36632400	3.87784000
C	1.55076700	-2.77229400	1.20697800
H	0.98355000	-2.27319300	0.41101200
C	1.71026400	-4.25864400	0.81405600
H	2.27155000	-4.80846100	1.57808200
H	0.74189200	-4.75387500	0.68256400
H	2.25931100	-4.33555900	-0.12997100
C	2.91318200	-2.07408600	1.31358800
H	3.40067400	-2.08221900	0.33389100
H	2.80917900	-1.03122600	1.63115500
H	3.58488000	-2.58757100	2.01142200
C	-1.89992400	-2.36481700	6.00650500
H	-2.78408900	-1.76359000	5.77605300
H	-2.23845100	-3.35832000	6.32660900
H	-1.37419000	-1.90078500	6.84489800
C	-4.07330200	1.99759300	0.90768400
C	-2.83602600	1.49652000	1.27298500
C	-1.89420000	1.17421000	0.27251400
C	-2.19869200	1.36745000	-1.10318100
C	-3.46637700	1.88299800	-1.44458100
C	-4.38504200	2.18919000	-0.45370700
H	-4.80420100	2.25035100	1.66937800
H	-2.57352000	1.35358200	2.31637800
H	-3.71243400	2.03977800	-2.49069000
H	-5.35751700	2.58670000	-0.72952700
C	-0.59542800	0.65627000	0.62940600
O	-0.16788100	0.46903700	1.78012300
C	1.98999700	-0.46347400	-4.75144200
C	0.91605600	-0.14622100	-3.92456700
C	1.13198400	0.18955600	-2.57557300
C	2.44591900	0.18742300	-2.07260400
C	3.51604000	-0.13053000	-2.90610800
C	3.29302300	-0.45527700	-4.24619800
H	1.80914300	-0.72556700	-5.78956000
H	-0.09381600	-0.18258800	-4.32183900
H	2.62546800	0.45532800	-1.03709700
H	4.52752400	-0.11512200	-2.51070900

H	4.12915700	-0.70220200	-4.89368300
C	0.00372100	0.55411000	-1.71183300
O	0.27204800	0.35713900	-0.37003100
C	-1.20332000	1.04994300	-2.08419800
H	-1.40375300	1.23630300	-3.13228300
C	-0.57569200	2.74288400	5.18914800
H	-1.49889200	2.85420800	5.76286500
H	-0.60413100	3.45121600	4.35233500
H	0.29253900	2.97797900	5.80840700
C	-0.46438600	1.35263400	4.64676000
O	-1.49778900	0.67256300	4.30860100
O	0.66397500	0.78749900	4.46536500

IM18

C	-2.74796700	7.34830200	-2.18487300
C	-3.23592200	6.11182500	-1.77343600
C	-3.16622300	4.97769800	-2.59573800
C	-2.62437400	5.09751900	-3.90410300
C	-2.16088000	6.36983600	-4.30897200
C	-2.20453100	7.47515700	-3.46342200
H	-2.80187500	8.20573400	-1.51967100
H	-3.68546900	5.99028000	-0.79376800
H	-1.81500200	6.49950000	-5.33112400
H	-1.83964400	8.43678900	-3.81522500
C	-3.69633900	3.70958800	-1.96489800
O	-4.57099000	3.81265000	-1.06574800
C	-3.15917100	2.45402100	-2.34198800
S	-3.70492500	1.07621100	-1.52430100
O	-3.06753600	-0.18310900	-1.97454900
C	-3.49102200	1.27123700	0.26757100
H	-3.91133000	2.24227700	0.53632200
H	-3.99169000	0.44400400	0.77628600
H	-2.41404700	1.23783400	0.44409700
C	-5.50750800	0.96663200	-1.66619400
H	-5.86380400	0.16384900	-1.01637200
H	-5.90948300	1.94180300	-1.38481700
H	-5.71133700	0.73567300	-2.71358600
C	-2.89843700	1.09668100	-8.16285600
C	-2.87094900	2.13088500	-7.23102300
C	-1.64884400	2.62545500	-6.74341800
C	-0.45546200	2.07272800	-7.23860100
C	-0.48645600	1.04016400	-8.17406800
C	-1.70619700	0.54457600	-8.63761800
H	-3.85337100	0.73022600	-8.53025700
H	-3.80262600	2.57702100	-6.89693000
H	0.49254700	2.45595400	-6.87827100
H	0.44659900	0.62069900	-8.54110300
H	-1.72881800	-0.25896300	-9.36885600
C	-1.60220000	3.70793600	-5.73915600
O	-0.40660600	4.37720900	-5.75525500
C	-2.60420400	3.98477000	-4.86787200
H	-3.44504200	3.30228400	-4.85127700
H	-0.37262100	4.95794600	-4.97515700
H	-2.35007500	2.24971000	-3.02798100

IM19

C	-2.53129500	6.99081700	-2.25349800
C	-3.04560800	5.72259500	-2.01011000
C	-3.07052900	4.73712500	-3.01059100
C	-2.61293600	5.05227700	-4.31549100
C	-2.08845300	6.34155300	-4.53512600
C	-2.03461300	7.29449100	-3.52314600
H	-2.51276400	7.73405400	-1.46095400
H	-3.42764400	5.45390600	-1.03106600
H	-1.74385700	6.59440700	-5.53313800
H	-1.62538900	8.27963000	-3.73233000
C	-3.51981100	3.37664300	-2.54394500
O	-4.23970100	3.28487800	-1.52280100
C	-2.98739500	2.20868700	-3.18661500
S	-3.49969900	0.68757500	-2.60995800
O	-2.96063900	-0.45078100	-3.38856200
C	-3.03221100	0.55736700	-0.86855900
H	-3.44102700	1.43027500	-0.35661300
H	-3.42717700	-0.38252500	-0.47593200
H	-1.94022400	0.56246800	-0.84734500
C	-5.31019500	0.58068700	-2.52715500
H	-5.58452000	-0.34086800	-2.00824000
H	-5.66256300	1.47336400	-2.00694800
H	-5.65717700	0.55633000	-3.56234000
C	-3.58436100	2.38748800	-9.41044900
C	-3.35517100	3.11052500	-8.24232000
C	-2.07572100	3.15047000	-7.66177200
C	-1.02637900	2.47120500	-8.30314200
C	-1.25695800	1.75260000	-9.47473900
C	-2.53642900	1.70307400	-10.03116200
H	-4.57990400	2.37143800	-9.84646000
H	-4.16719000	3.67164900	-7.78948200
H	-0.03414400	2.51229300	-7.86811200
H	-0.43432200	1.22829600	-9.95419000
H	-2.71456000	1.14447600	-10.94627100

C	-1.81425200	3.89200800	-6.40755100
O	-0.51113000	4.26237700	-6.27539100
C	-2.76678500	4.15214500	-5.47648800
H	-3.73663200	3.68345700	-5.61037000
H	-0.28258100	4.33408700	-5.32534600
H	-2.62118700	2.15553100	-4.20434300
C	2.02244500	3.13774200	-1.95181200
H	2.73899500	3.76902300	-2.47764900
H	1.88947100	3.49813800	-0.92632700
H	2.40067200	2.11201500	-1.89073100
C	0.69824000	3.15559000	-2.67681500
O	0.48251600	3.80233300	-3.68590200
O	-0.21050900	2.37730000	-2.07578800
H	-1.08053700	2.41665600	-2.56395300

TS12

C	-2.61338900	7.06637700	-2.29252400
C	-3.12342800	5.80031200	-2.04278000
C	-3.11446300	4.80248300	-3.03642100
C	-2.63586300	5.10359300	-4.33800300
C	-2.10352700	6.38742400	-4.55585400
C	-2.07924800	7.35080700	-3.55297600
H	-2.61525400	7.82047500	-1.51061400
H	-3.50998800	5.53958400	-1.06341900
H	-1.72806700	6.62564800	-5.54601700
H	-1.66046100	8.33209900	-3.76055100
C	-3.49715900	3.44359300	-2.56221500
O	-4.16047900	3.28815300	-1.52695400
C	-2.87056400	2.26605900	-3.23483700
S	-3.36368900	0.69454200	-2.58952600
O	-2.88708400	-0.42769700	-3.42443000
C	-2.70025300	0.62294400	-0.91661800
H	-3.19554400	1.39590100	-0.32679200
H	-2.88598700	-0.38315700	-0.53395200
H	-1.63096000	0.83557100	-1.01797900
C	-5.15804400	0.58802900	-2.37940000
H	-5.37309100	-0.35741600	-1.87550100
H	-5.48757000	1.45483900	-1.80640300
H	-5.58239200	0.58392300	-3.38597600
C	-3.51310300	2.56055100	-9.49750400
C	-3.28324200	3.25539300	-8.31209500
C	-2.05964500	3.13041800	-7.63273100
C	-1.06153900	2.31443300	-8.19003900
C	-1.29198300	1.62272300	-9.37784800
C	-2.51909100	1.73841800	-10.03393400
H	-4.46378900	2.67351000	-10.01233200
H	-4.04654600	3.92336300	-7.92365300
H	-0.11019300	2.23102300	-7.67657900
H	-0.51077800	0.99073000	-9.79221600
H	-2.69664000	1.20107000	-10.96186100
C	-1.79754200	3.84003700	-6.35622000
O	-0.48758900	4.05187500	-6.12571000
C	-2.79057400	4.20469800	-5.50120800
H	-3.79736700	3.85929400	-5.72074900
H	-0.28424700	4.07992800	-5.14793200
H	-2.92460800	2.20668600	-4.32132000
C	1.78963500	2.99273300	-1.90120900
H	2.47319900	3.78294800	-2.21668400
H	1.57070700	3.09383500	-0.83269600
H	2.26764700	2.01664000	-2.04198400
C	0.49648900	3.04805600	-2.70791700
O	0.37961100	3.88856200	-3.61622300
O	-0.39369200	2.18180500	-2.35310300
H	-1.62471600	2.29377000	-2.93529300

IM20

C	-2.52944700	6.88504800	-2.18607500
C	-3.25748500	5.71646300	-2.07791500
C	-3.20205400	4.73034800	-3.09291100
C	-2.46432300	4.97417300	-4.28854800
C	-1.68715800	6.15314000	-4.34027700
C	-1.71816300	7.08574100	-3.31717500
H	-2.56518300	7.62994600	-1.39637300
H	-3.85359800	5.50788600	-1.19601300
H	-1.08908900	6.33735100	-5.22579400
H	-1.12288500	7.99123000	-3.40219200
C	-3.80379100	3.43876400	-2.75145900
O	-4.53891400	3.27998400	-1.76778500
C	-3.38665900	2.18664700	-3.54796100
S	-3.39078000	0.67741900	-2.52279500
O	-2.90929800	-0.49106200	-3.28558400
C	-2.37955900	1.03567800	-1.08440900
H	-2.89987400	1.77243500	-0.46986100
H	-2.23486600	0.08666200	-0.56271800
H	-1.44197200	1.44427700	-1.50958900
C	-5.06778300	0.37952600	-1.92584200
H	-5.02035900	-0.48368400	-1.25759500
H	-5.41894400	1.28731000	-1.43562200
H	-5.67008400	0.13857300	-2.80526600
C	-3.49086200	2.63909200	-9.47840200

C	-3.20603800	3.28895800	-8.27911300
C	-1.95580300	3.13164100	-7.65743300
C	-0.98929700	2.32763000	-8.28394400
C	-1.27563000	1.67777000	-9.48262400
C	-2.52803200	1.82716600	-10.08266600
H	-4.46026600	2.77849700	-9.95010600
H	-3.94637600	3.94974200	-7.83762300
H	-0.01880800	2.22204400	-7.81198100
H	-0.51920500	1.05298500	-9.95043700
H	-2.74936500	1.32335100	-11.02001300
C	-1.62059000	3.79794500	-6.37030700
O	-0.32546800	3.98533600	-6.18780500
C	-2.60436500	4.13228400	-5.46835700
H	-3.60515100	3.77647600	-5.68959200
H	-0.00572900	3.99241300	-5.17306700
H	-4.04648700	1.93806400	-4.38389900
C	1.34816100	3.17161600	-1.77597900
H	2.18467100	3.85773500	-1.92208700
H	0.78773200	3.47290400	-0.88247400
H	1.72871800	2.15994000	-1.59562300
C	0.41708500	3.16498500	-2.99084700
O	0.69444200	3.98387700	-3.91456600
O	-0.55309300	2.35471500	-2.97400300
H	-2.33372500	2.24401300	-3.87282200

IM21

C	-3.15601400	7.93596000	-2.87219900
C	-3.04602400	6.60135600	-2.50610300
C	-3.15894600	5.56993500	-3.45594300
C	-3.31024400	5.87674100	-4.83960300
C	-3.45053900	7.24591700	-5.17225600
C	-3.38347600	8.25078200	-4.21844100
H	-3.08170300	8.71967800	-2.12393200
H	-2.89685700	6.32291300	-1.46722900
H	-3.57854300	7.50876800	-6.21961000
H	-3.49155300	9.28862200	-4.52432300
C	-3.22700200	4.20002900	-2.88453000
O	-2.64689800	3.85766200	-1.86414000
C	-4.44217400	3.37962600	-3.37956000
S	-4.46679900	1.58293200	-3.08113500
O	-5.87516300	1.12390400	-3.03362300
C	-3.53292200	0.70638400	-4.34153500
H	-2.47092100	0.91345400	-4.19050500
H	-3.78586700	-0.34666200	-4.18652500
H	-3.87424000	1.05116000	-5.31814900
C	-3.59643700	1.20066400	-1.55370700
H	-3.82699300	0.14816700	-1.36426800
H	-2.52829300	1.36836200	-1.70728400
H	-3.99490800	1.84198200	-0.76849100
C	-2.86078200	2.23914500	-9.36640500
C	-3.13275800	2.98737300	-8.22196600
C	-2.28478300	2.92883300	-7.10192000
C	-1.16588700	2.08085500	-7.16069400
C	-0.88766400	1.34187600	-8.30952700
C	-1.73375700	1.41580400	-9.41788000
H	-3.53501100	2.29424200	-10.21746400
H	-4.02386900	3.60730600	-8.18800100
H	-0.50541700	2.02057400	-6.30239000
H	-0.00713600	0.70481300	-8.33862600
H	-1.52094100	0.83363000	-10.31062600
C	-2.55548000	3.70971800	-5.84939100
O	-2.13169800	3.19726300	-4.72754600
C	-3.23188800	4.91430800	-5.92047600
H	-3.48752100	5.29469400	-6.90389500
H	-0.65384000	2.93005000	-4.19361600
H	-5.32555100	3.69304300	-2.80744800
C	1.59949200	1.45783400	-2.35846300
H	2.39987400	1.46122600	-3.10481600
H	1.80544500	2.27578200	-1.65914000
H	1.58812200	0.50937100	-1.82003700
C	0.26274700	1.69311200	-3.02517600
O	0.27297100	2.75500600	-3.81401400
O	-0.71229300	0.96926500	-2.84031900
H	-4.64732600	3.51219700	-4.44594100

IM22

C	-0.60015000	4.30739800	1.87906500
C	-0.44252100	2.92319600	1.97811900
C	-0.47909900	2.12963900	0.83469500
C	-0.64432900	2.71247700	-0.43908300
C	-0.80977100	4.10608000	-0.52485600
C	-0.79531600	4.89313300	0.62439100
H	-0.57276600	4.92506900	2.77266900
H	-0.28164200	2.43842500	2.93636500
H	-0.93053900	4.56871900	-1.50185200
H	-0.92416800	5.96932100	0.54092800
C	-0.41885000	0.60914200	0.96594300
O	0.19054500	0.12611900	2.02175100
C	-1.91529400	0.08661100	0.88505000
S	-2.09751500	-1.66284800	1.33562100

O	-3.44089300	-2.19026000	0.99701500
C	-0.78775800	-2.62399900	0.57866400
H	0.19472000	-2.28541600	0.93772200
H	-1.00040100	-3.66068700	0.85541500
H	-0.86963700	-2.49042700	-0.50109800
C	-1.76361400	-1.73361400	3.10124100
H	-1.66629900	-2.78497600	3.38087500
H	-0.84603000	-1.14918200	3.23790100
H	-2.62431600	-1.28179000	3.60027900
C	0.09831100	-0.92556000	-4.97005300
C	-0.24221500	-0.08161000	-3.91672100
C	0.27397600	-0.29714600	-2.62608300
C	1.13262700	-1.39333200	-2.42053600
C	1.47237100	-2.23356300	-3.48088000
C	0.95965000	-2.00558300	-4.75848100
H	-0.31654900	-0.74423300	-5.95813500
H	-0.93042100	0.73925200	-4.09451900
H	1.54010100	-1.57394500	-1.43127200
H	2.14403600	-3.06969800	-3.30439800
H	1.22345400	-2.66416200	-5.58163000
C	-0.06435200	0.59612600	-1.49699800
O	0.21628000	0.04799600	-0.28355100
C	-0.54730200	1.86060900	-1.60998200
H	-0.71638900	2.29132400	-2.58993400
H	1.70445600	-0.10616500	1.93279300
H	-2.54808400	0.60860900	1.60962700
C	4.52756800	-1.71443600	1.48898800
H	5.00209000	-1.02258900	0.78434600
H	4.95430000	-1.51158700	2.47651400
H	4.73840300	-2.74289000	1.19238000
C	3.03098700	-1.47296100	1.50821600
O	2.72364500	-0.27302200	1.95749700
O	2.22524300	-2.32344700	1.13567400
H	-2.36385500	0.16883800	-0.11028100

TS13

C	-3.37253300	7.90286700	-2.72787300
C	-2.88421600	6.60429700	-2.59514900
C	-2.81722900	5.76561500	-3.70880100
C	-3.22093000	6.22681400	-4.97989700
C	-3.71661700	7.53760800	-5.09708500
C	-3.79547600	8.36415200	-3.98077300
H	-3.42252300	8.55645600	-1.86146100
H	-2.54570500	6.22277800	-1.63710500
H	-4.02788500	7.90346900	-6.07263300
H	-4.17995100	9.37529400	-4.08521300
C	-2.34404200	4.35572800	-3.55354100
O	-1.69004800	3.99291700	-2.54680300
C	-4.25632100	3.53414200	-3.45688400
S	-4.37660800	1.87216600	-2.99129100
O	-5.69934700	1.19460200	-2.88788600
C	-3.31637300	0.97183500	-4.13407500
H	-2.27399500	1.21805500	-3.92165200
H	-3.51049200	-0.08877300	-3.96109100
H	-3.60433200	1.25503300	-5.14801400
C	-3.49848800	1.79833500	-1.42417200
H	-3.31067400	0.74910000	-1.19082100
H	-2.57160200	2.36935900	-1.53804200
H	-4.16055500	2.24584500	-0.67894400
C	-2.37870200	2.38629200	-9.32418000
C	-2.74135900	3.24524400	-8.29019200
C	-2.02147700	3.25709000	-7.08216400
C	-0.93808500	2.37250700	-6.93451200
C	-0.57854800	1.51571100	-7.97330800
C	-1.29429500	1.51849200	-9.17177500
H	-2.95078900	2.38739400	-10.24808400
H	-3.60234800	3.89564400	-8.41267200
H	-0.37587100	2.37585400	-6.00716400
H	0.26655100	0.84459300	-7.84534600
H	-1.01423200	0.84703200	-9.97876800
C	-2.37867200	4.17728100	-5.98613400
O	-1.88831000	3.77078200	-4.77505700
C	-3.05463000	5.34508900	-6.11483500
H	-3.38382000	5.67197100	-7.09438600
H	-0.27473400	3.14378500	-2.75837200
H	-4.75170800	4.12708500	-2.68548400
C	1.86702500	0.70725900	-2.57228000
H	2.48236800	0.98850500	-3.43301100
H	2.39851300	1.03955100	-1.67390400
H	1.73417400	-0.37484600	-2.54246300
C	0.51941400	1.39156100	-2.64945200
O	0.63478800	2.70896200	-2.77747600
O	-0.54536600	0.79056000	-2.59513200
H	-4.74343700	3.65523800	-4.42687800

IM23

C	-1.22395100	4.46429100	1.71748100
C	-0.40093700	3.35027500	1.79556700
C	-0.18431000	2.56094500	0.65620900
C	-0.78985500	2.89059200	-0.57917300

C	-1.62090100	4.02691300	-0.63705700
C	-1.83414700	4.79835000	0.49606600
H	-1.39629600	5.07810000	2.59649300
H	0.08508800	3.06831000	2.72357100
H	-2.09510400	4.29084800	-1.57862400
H	-2.47944200	5.67072000	0.43776400
C	0.69027200	1.39633600	0.73561800
O	1.29324600	1.02416700	1.73451300
C	-2.21620500	-0.19188100	0.90751000
S	-2.28747100	-1.59819500	1.80609900
O	-3.52897100	-2.26102800	2.32342400
C	-1.38757800	-2.82343800	0.82097800
H	-0.33316900	-2.55325900	0.76437100
H	-1.52278900	-3.79167600	1.30712700
H	-1.85089100	-2.83659500	-0.16848000
C	-1.19588500	-1.30257200	3.22045900
H	-1.29113300	-2.16075300	3.88875600
H	-0.16915800	-1.17287900	2.87742300
H	-1.56398000	-0.40221200	3.71822300
C	0.32459000	-0.90939400	-4.88606500
C	-0.05855800	-0.03432000	-3.87430500
C	0.67821900	0.04449700	-2.67900100
C	1.79921200	-0.78938700	-2.51854100
C	2.17701400	-1.66391500	-3.53545400
C	1.44589900	-1.72686900	-4.72256500
H	-0.25952600	-0.96069400	-5.80074100
H	-0.94809400	0.57397600	-4.00588700
H	2.37078700	-0.74410100	-1.59815500
H	3.04864200	-2.29805500	-3.39863300
H	1.74223400	-2.41138800	-5.51251700
C	0.29349000	0.98338400	-1.61421100
O	0.90719800	0.69601200	-0.41968100
C	-0.52757100	2.05589400	-1.71973500
H	-0.97457900	2.30304600	-2.67492000
H	2.45257900	-0.27223000	1.48349500
H	-2.59540300	0.65567700	1.47886600
C	3.96704100	-3.13237900	1.42201100
H	4.37123900	-3.06057100	0.40701400
H	4.78215100	-2.88935000	2.11215200
H	3.61127400	-4.14526200	1.61311900
C	2.83436000	-2.14721800	1.60404200
O	3.21237000	-0.89698200	1.32071400
O	1.71359100	-2.45687400	1.97159600
H	-2.70070800	-0.32045000	-0.06102100

TS14

C	-4.21260700	8.36885300	-2.41270600
C	-4.33822300	7.09556800	-1.87023300
C	-3.81130700	5.97567600	-2.53406200
C	-3.21552700	6.08762700	-3.82417100
C	-3.07425800	7.40684200	-4.32426300
C	-3.55095400	8.51526400	-3.64011200
H	-4.60867000	9.23264500	-1.88721300
H	-4.81473300	6.95784200	-0.90252400
H	-2.60543400	7.53645400	-5.29683700
H	-3.42744900	9.50502900	-4.07349400
C	-3.82926500	4.69150800	-1.80370700
O	-5.00921700	4.08154100	-1.58420300
C	-2.68003000	4.15688600	-1.31091400
S	-2.52191500	2.49672400	-0.83643500
O	-2.32402500	2.19356200	0.59926700
C	-3.85112200	1.51100600	-1.55525500
H	-4.04999200	1.88154500	-2.57452600
H	-3.49200400	0.47896100	-1.52375500
H	-4.72177100	1.63948100	-0.91394900
C	-1.07656500	1.97432300	-1.79192000
H	-0.88875000	0.92397100	-1.55785800
H	-1.27841000	2.12412800	-2.85577500
H	-0.23309900	2.58640300	-1.46463000
C	-1.08049300	1.70417100	-6.98662800
C	-1.53445800	2.69200900	-6.11193800
C	-2.85812600	2.68604100	-5.63919700
C	-3.70566500	1.64674800	-6.05700900
C	-3.25634200	0.66372300	-6.93671300
C	-1.94049400	0.68693500	-7.40636000
H	-0.05127000	1.72640400	-7.33718500
H	-0.85178700	3.46971400	-5.78099600
H	-4.72233100	1.63686500	-5.67791500
H	-3.93384700	-0.12391700	-7.25833200
H	-1.58748800	-0.08203000	-8.08886100
C	-3.39469000	3.70982000	-4.67020700
O	-4.39607300	3.32227700	-3.95009600
C	-2.79684700	4.96626100	-4.63613000
H	-2.10209300	5.20717800	-5.43485900
H	-1.73043500	4.67519200	-1.36421900
H	-5.34793000	3.98212300	-2.51706500

IM24

C	-1.23208100	4.56201700	0.81831500
C	-1.22397000	3.34805900	1.46967800

C	-0.67409200	2.17447600	0.88042200
C	-0.17146400	2.21129300	-0.47190800
C	-0.14628300	3.51157600	-1.07314600
C	-0.66019100	4.63637200	-0.46959200
H	-1.63225000	5.44747700	1.30250500
H	-1.57261300	3.30106400	2.49912100
H	0.26175300	3.58797500	-2.07778800
H	-0.63098200	5.58788500	-0.99513200
C	-0.56624700	1.02428100	1.75163900
O	-1.55356500	0.81563400	2.66649100
C	0.50684600	0.17582900	1.78611600
S	0.46485100	-1.37429300	2.51154400
O	0.89930200	-1.52613200	3.92201000
C	-1.16618700	-2.12346700	2.28302400
H	-1.42571500	-2.01639000	1.22749900
H	-1.06644700	-3.16242900	2.60586700
H	-1.85628100	-1.58459500	2.92981600
C	1.51732800	-2.35571200	1.41909800
H	1.58160900	-3.35809700	1.84806500
H	1.06799700	-2.35197900	0.42425600
H	2.50538100	-1.89013400	1.41285200
C	2.20962200	-1.70181300	-4.00584200
C	1.71740300	-0.83805100	-3.02674600
C	0.52017100	-1.12239100	-2.35045400
C	-0.15922500	-2.30773600	-2.67427900
C	0.32484600	-3.16751500	-3.65851300
C	1.51288300	-2.86782000	-4.32993600
H	3.14268900	-1.46653500	-4.51221100
H	2.28024100	0.05446100	-2.76894700
H	-1.07673400	-2.53135100	-2.13914800
H	-0.22490900	-4.07311700	-3.90436600
H	1.89489000	-3.53895600	-5.09503300
C	-0.05154100	-0.24722800	-1.25349900
O	-0.78366600	-0.82115500	-0.39321100
C	0.26605300	1.12815600	-1.29589700
H	0.74190500	1.45694000	-2.21505700
H	1.34744000	0.33268100	1.12335900
H	-2.33970900	1.30589900	2.37059600

IM25

C	-3.57124000	7.90214800	-2.37675200
C	-3.51127300	6.55184200	-2.02949400
C	-3.33124700	5.57433400	-3.00805000
C	-3.22581200	5.94357100	-4.36605600
C	-3.29989400	7.30719400	-4.70253200
C	-3.46708100	8.27691200	-3.71936300
H	-3.71398500	8.65515500	-1.60653500
H	-3.62588700	6.24821400	-0.99322200
H	-3.21897900	7.59748200	-5.74756400
H	-3.51979600	9.32619200	-3.99853700
C	-3.36602700	4.09587800	-2.66140400
O	-4.70256000	3.67971600	-2.73944100
C	-2.72001000	3.80623800	-1.33031600
S	-2.56822100	2.22128300	-0.89545600
O	-3.81230800	1.41258900	-1.09414800
C	-1.24354600	1.16619500	-1.63444400
H	-0.27052200	1.62249300	-1.43305200
H	-1.30570100	0.15840400	-1.21586900
H	-1.43755800	1.15858100	-2.70763700
C	-2.02077800	2.25316400	0.83104900
H	-1.84072300	1.22440000	1.15077300
H	-1.10801400	2.84823600	0.91410800
H	-2.82746800	2.70567300	1.40861200
C	-1.72481200	1.64336900	-8.04437000
C	-1.93087100	2.71831900	-7.18288600
C	-2.48381300	2.51812900	-5.90655800
C	-2.80942400	1.20870700	-5.51167700
C	-2.59973500	0.13491400	-6.37534600
C	-2.05749800	0.34655500	-7.64462600
H	-1.29175900	1.81651400	-9.02616200
H	-1.64181800	3.71975400	-7.48814200
H	-3.25381500	1.04877600	-4.53464500
H	-2.86833100	-0.86972900	-6.05881600
H	-1.89215000	-0.49175500	-8.31610800
C	-2.73234500	3.64785100	-4.99276400
O	-2.60444600	3.31816400	-3.67364000
C	-3.03582000	4.91150700	-5.36566000
H	-3.14087200	5.15826100	-6.41609900
H	-1.90409600	4.43569000	-0.99101800
H	-4.77519800	2.83524100	-2.25374000

IM26

C	-8.04871100	6.81102300	-3.14215000
C	-7.11325700	6.19735700	-2.32874600
C	-5.80940000	5.90329000	-2.78738400
C	-5.38802400	6.33119600	-4.08653000
C	-6.39232100	6.92133800	-4.90278000
C	-7.68020500	7.15101400	-4.45476100
H	-9.05337600	7.00627100	-2.77888200
H	-7.37311200	5.88650300	-1.32174500

H	-6.11180000	7.23803200	-5.90439800
H	-8.40462200	7.61388900	-5.12084700
C	-5.02309600	5.05973600	-1.87443200
O	-5.17332100	5.03401600	-0.65587500
C	-4.21849600	3.91648400	-2.51277200
S	-2.76239500	3.42513000	-1.51788700
O	-2.95334900	2.07443400	-0.93884600
C	-1.34030100	3.42557500	-2.63021000
H	-1.14172600	4.45295600	-2.93589700
H	-0.51449600	2.98399500	-2.06679000
H	-1.59301500	2.78980500	-3.48154400
C	-2.33053000	4.62139300	-0.23695300
H	-1.47483800	4.17226600	0.27426100
H	-2.10659700	5.56831400	-0.72816800
H	-3.20020100	4.70262500	0.41344700
C	-0.13953400	6.71554800	-6.56414800
C	-1.35472200	6.39682600	-5.95884600
C	-1.57329100	6.66369700	-4.59668200
C	-0.52906300	7.24583600	-3.85882500
C	0.68163400	7.57664400	-4.46584700
C	0.88283400	7.31214000	-5.82230700
H	0.01137500	6.49257000	-7.61752800
H	-2.13362200	5.91659300	-6.54406600
H	-0.69468700	7.44491100	-2.80486700
H	1.46979100	8.04331800	-3.87978300
H	1.82803500	7.56374800	-6.29622000
C	-2.85671100	6.32470700	-3.88168400
O	-2.76148000	6.07660100	-2.62324900
C	-4.04434800	6.31427800	-4.61352600
H	-3.97203600	6.61807500	-5.65303200
H	-3.89728900	4.11513300	-3.53724200
H	-4.82092600	3.00036900	-2.46238600

TS15

C	-7.40764800	4.83905800	-2.20518800
C	-6.04656200	4.65754700	-1.95715900
C	-5.11456500	4.85542500	-2.97439900
C	-5.53645100	5.21472700	-4.27235500
C	-6.91068300	5.40205700	-4.50669400
C	-7.83507700	5.22208900	-3.48143700
H	-8.13246600	4.68235300	-1.41082300
H	-5.68502200	4.35397700	-0.97946000
H	-7.24728200	5.67627100	-5.50399200
H	-8.89379600	5.37005700	-3.67861600
C	-3.64084800	4.73819400	-2.66509000
O	-3.23836800	4.08908500	-1.67256000
C	-3.19690100	6.58841000	-2.54255000
H	-3.09097800	7.01977900	-3.53999900
S	-1.70544000	6.84210900	-1.66872600
O	-1.05444100	8.17844800	-1.64870100
C	-2.11177900	6.25693300	-0.01895400
H	-2.58842500	5.27686000	-0.16678900
H	-1.18858200	6.20673100	0.56228900
H	-2.79687900	6.98608900	0.42002200
C	-0.56701900	5.58505500	-2.27297500
H	0.36003100	5.69753100	-1.70652300
H	-1.05683000	4.61847800	-2.11935800
H	-0.39835500	5.77034400	-3.33498500
C	-1.28584200	5.42121100	-8.29731000
C	-2.28403300	5.53052200	-7.33280200
C	-2.22158000	4.78005800	-6.14503700
C	-1.12041500	3.92743100	-5.94983800
C	-0.12438400	3.81848500	-6.91923800
C	-0.20120600	4.56325700	-8.09703700
H	-1.35042400	6.01509300	-9.20524900
H	-3.10679100	6.22064500	-7.49350900
H	-1.06650200	3.34083300	-5.03945100
H	0.71328100	3.14572500	-6.75408400
H	0.57739500	4.48072100	-8.85049900
C	-3.27529500	4.87065500	-5.11188700
O	-2.87389900	4.40740000	-3.89857700
C	-4.54261100	5.31117700	-5.32199100
H	-3.98089600	7.09913500	-1.97847800
H	-4.85059000	5.62451500	-6.31283700

IM27

C	-3.04349900	7.54726200	-2.24079500
C	-3.13111900	6.19649500	-1.91476200
C	-2.86256800	5.18898600	-2.85378200
C	-2.46554500	5.54757400	-4.17153600
C	-2.39556100	6.92188900	-4.47584600
C	-2.67633900	7.90888800	-3.53733500
H	-3.27166400	8.30493900	-1.49632700
H	-3.45973500	5.91580000	-0.91769600
H	-2.09644900	7.20890700	-5.48092200
H	-2.60686200	8.95690900	-3.81710600
C	-3.11611600	3.75751300	-2.45760800
O	-3.68899900	2.97281400	-3.23374500
C	-2.64281600	3.33964400	-1.15766500
S	-3.03281000	1.74433100	-0.67851500

O	-2.62083700	1.42729400	0.70908800
C	-4.79839000	1.36817200	-0.88774900
H	-5.07436700	1.66257600	-1.90180100
H	-4.95127700	0.30205500	-0.70408800
H	-5.32497100	1.96493700	-0.13983700
C	-2.25406100	0.63635800	-1.87024900
H	-2.62236200	-0.37696900	-1.69463800
H	-2.49302000	1.00460100	-2.86938300
H	-1.18112700	0.69962700	-1.67945600
C	-2.14719600	1.69126500	-8.52474800
C	-2.30317400	2.51814600	-7.41463100
C	-1.32199600	2.56447100	-6.40915100
C	-0.19676100	1.73176600	-6.53353500
C	-0.04452500	0.90175100	-7.64268500
C	-1.01574200	0.88065200	-8.64571400
H	-2.91880500	1.66764000	-9.29007200
H	-3.20419500	3.11407500	-7.30700400
H	0.55691200	1.75259900	-5.75403500
H	0.83716800	0.27110400	-7.72574200
H	-0.89759300	0.23147500	-9.50943000
C	-1.45739700	3.45278500	-5.23292800
O	-0.78816900	2.98129900	-4.14350600
C	-2.15886900	4.61101000	-5.26424300
H	-2.49271700	4.93843800	-6.24437200
H	-0.48420900	3.72093500	-3.56847500
H	-2.55338000	3.99447500	-0.29740500
C	1.00600100	4.38635400	-1.43018200
O	0.66195800	4.68255900	-2.56485600
O	0.26430500	3.66245500	-0.58964400
C	2.32043600	4.80328100	-0.81878800
H	2.13746400	5.43994000	0.05329800
H	2.86501600	3.92073400	-0.46802200
H	2.91503800	5.34382500	-1.55542100
H	-0.63349200	3.47885700	-0.98881600

TS16

C	-2.50552300	7.51833900	-2.23541700
C	-2.63882200	6.19140700	-1.85595100
C	-2.57147400	5.13352200	-2.78729800
C	-2.28032100	5.42300700	-4.15677300
C	-2.18544100	6.78985700	-4.51089400
C	-2.29563100	7.81648900	-3.58555500
H	-2.57026100	8.30870400	-1.49366400
H	-2.80699700	5.96979800	-0.80842300
H	-1.98702600	7.03158900	-5.55185700
H	-2.20094100	8.84909700	-3.91158900
C	-2.90653800	3.76542500	-2.31860800
O	-3.19164600	2.84889500	-3.09360000
C	-2.82742400	3.49638600	-0.83932100
S	-2.99512800	1.79026700	-0.44200200
O	-2.90087300	1.55349400	1.01556100
C	-4.54605400	1.08765000	-1.06288800
H	-4.61798700	1.32143700	-2.12523200
H	-4.52535300	0.01223500	-0.86901300
H	-5.34753900	1.55831400	-0.48879800
C	-1.71078100	0.91813700	-1.35719100
H	-1.64281300	-0.07629700	-0.91021500
H	-1.97831600	0.89502200	-2.41380400
H	-0.78970600	1.49651100	-1.23918200
C	-2.32425000	1.95941000	-8.80622300
C	-2.40216200	2.65719400	-7.60291300
C	-1.39001100	2.53818200	-6.63541900
C	-0.31255400	1.67502700	-6.89762800
C	-0.23711800	0.97615300	-8.10103500
C	-1.23957500	1.11762700	-9.06264500
H	-3.12007000	2.06210900	-9.53949400
H	-3.26831600	3.27867800	-7.39725300
H	0.46531400	1.56671100	-6.14998400
H	0.60910900	0.32028600	-8.28926800
H	-1.18127800	0.57042300	-9.99990900
C	-1.44409200	3.28291700	-5.35153000
O	-0.77263900	2.63628900	-4.38158800
C	-2.07896500	4.48573400	-5.26309100
H	-2.37554100	4.89793400	-6.22272800
H	-0.57022900	3.17488100	-3.58103400
H	-3.52734700	4.03315300	-0.19404000
C	0.37612600	4.15367500	-1.00621700
O	0.17002500	3.40804400	-1.98972200
O	-0.47694400	4.36750600	-0.06561100
C	1.69810200	4.89132300	-0.87169900
H	1.55784000	5.93101400	-1.19161600
H	2.02344800	4.90984500	0.17213200
H	2.45975500	4.43020400	-1.50382800
H	-1.61422300	3.85908000	-0.40809000

IM28

C	-2.41225800	7.47862200	-2.09774500
C	-2.57736300	6.14752800	-1.74907000
C	-2.51028500	5.11017200	-2.70467100
C	-2.17869200	5.41996000	-4.06189000

C	-2.05583800	6.79285000	-4.38351500
C	-2.16921900	7.80013400	-3.43757900
H	-2.47462700	8.25411000	-1.34064400
H	-2.76989700	5.90798200	-0.70974600
H	-1.82923000	7.05394100	-5.41395500
H	-2.04893100	8.83761100	-3.73874100
C	-2.90421000	3.75527500	-2.28153900
O	-3.24940800	2.86188300	-3.05120400
C	-2.94528200	3.47251100	-0.76668700
S	-3.02351900	1.70432500	-0.40823600
O	-2.93599000	1.44787300	1.04391200
C	-4.58256500	1.07385400	-1.06403500
H	-4.63099800	1.33247700	-2.12210700
H	-4.59021800	-0.00522400	-0.89186300
H	-5.38048700	1.55122000	-0.49013100
C	-1.72657300	0.87575600	-1.33483400
H	-1.62887500	-0.11300300	-0.88053400
H	-2.00841800	0.84385500	-2.38813000
H	-0.83375800	1.50863300	-1.23632700
C	-2.20495700	2.05093800	-8.76692900
C	-2.28920700	2.72932300	-7.55280400
C	-1.31240700	2.54988400	-6.55876600
C	-0.26464300	1.64706600	-6.80705000
C	-0.18191200	0.96845800	-8.02145900
C	-1.14885800	1.16945400	-9.00861100
H	-2.97367300	2.20065400	-9.52077500
H	-3.13437500	3.38318600	-7.36068200
H	0.48450700	1.49227700	-6.03853900
H	0.64201200	0.28172800	-8.19843100
H	-1.08524200	0.63801900	-9.95458400
C	-1.37239100	3.26915900	-5.25845100
O	-0.76220100	2.58148600	-4.28592600
C	-1.96275500	4.49879900	-5.17315900
H	-2.20173400	4.93896700	-6.13637600
H	-0.53637000	3.08983000	-3.44833600
H	-3.82775400	3.88627100	-0.26426100
C	0.22522600	4.21145900	-1.06847800
O	-0.20859800	3.30951700	-1.87692900
O	-0.32350400	4.49155100	0.01940000
C	1.46626500	4.98558100	-1.50306500
H	1.20052300	5.65341300	-2.33192900
H	1.86143000	5.58375800	-0.67909100
H	2.23401400	4.29851800	-1.87355300
H	-2.00701400	3.81557900	-0.25814300

IM29

Ru	1.17080400	-1.60700800	0.77486900
C	3.02818400	-2.84828300	1.15514500
C	2.40109200	-3.34537900	-0.01197300
C	1.02433500	-3.72233900	-0.00547300
C	0.31150300	-3.58870600	1.22657800
C	0.94379300	-3.08769800	2.39397100
C	2.32524800	-2.70798400	2.38860000
H	4.04926500	-2.48612600	1.08880800
H	2.95195900	-3.36123700	-0.94668200
H	-0.75514900	-3.78703300	1.24626600
H	0.34570000	-2.92942300	3.28393300
C	3.03911100	-2.19280200	3.62484900
H	3.87388700	-1.57362900	3.27137500
C	3.63642300	-3.39415500	4.39362400
H	2.84592400	-4.05794900	4.76221300
H	4.31034000	-3.98455200	3.76389800
H	4.20587900	-3.03598500	5.25750800
C	2.16271600	-1.32681300	4.54279500
H	2.77759800	-0.90430700	5.34359000
H	1.69843300	-0.49829900	3.99662000
H	1.36929900	-1.91101400	5.02273600
C	0.33988600	-4.21984700	-1.24778700
H	0.81221300	-3.82432000	-2.15119900
H	0.40367400	-5.31547800	-1.28784200
H	-0.71856400	-3.94564400	-1.25748500
C	-3.96463800	0.86179800	2.80290400
C	-2.79313400	0.40554800	2.23605900
C	-2.24246600	1.01474900	1.08088000
C	-2.99788300	2.03379800	0.38718500
C	-4.16603500	2.52734300	1.05941700
C	-4.63712300	1.96223100	2.21946400
H	-4.34914600	0.40513000	3.70963900
H	-2.23086400	-0.39243100	2.70865100
H	-4.72042300	3.33407200	0.58786000
H	-5.54269200	2.34748800	2.67951900
C	-0.88485900	0.64081400	0.73152800
O	-0.55387700	-0.59299600	0.95349200
C	0.05994500	1.66387400	0.41137800
H	-0.26644300	2.69762900	0.36160900
S	1.44269400	1.38907200	-0.47751800
O	2.15826400	0.08018400	-0.14088700
C	1.42253100	1.38648100	-2.31054100
H	1.09247800	2.37302000	-2.64520600

H	2.43321500	1.15484600	-2.65710400
H	0.67403100	0.64828100	-2.60211300
C	2.54588700	2.77134700	-0.11795900
H	3.45543600	2.63686800	-0.70747100
H	2.04366600	3.70379200	-0.38869300
H	2.75828000	2.74221000	0.95160100
C	-2.48589300	4.30206400	-4.90600900
C	-2.42408800	3.80141100	-3.60554900
C	-2.18768700	2.43538000	-3.37481500
C	-2.00956300	1.58661100	-4.48056300
C	-2.08676400	2.08376900	-5.77932900
C	-2.32395300	3.44438900	-5.99640100
H	-2.66173700	5.36188800	-5.06796700
H	-2.53934000	4.48414600	-2.76862000
H	-1.82772500	0.53223700	-4.29865300
H	-1.96622700	1.41161500	-6.62459100
H	-2.38276100	3.83354900	-7.00897800
C	-2.07027700	1.84652500	-1.99643500
O	-1.39727700	0.79283200	-1.85565000
C	-2.77902000	2.49806200	-0.93198300
H	-3.44136100	3.30087200	-1.23899600

TS17

Ru	-1.20788200	2.17521700	-2.55088200
C	0.54129400	0.86227500	-2.01999300
C	0.19974600	0.57280000	-3.36039300
C	-1.13789400	0.24651400	-3.72196700
C	-2.11203400	0.17222000	-2.67075400
C	-1.76048000	0.42358500	-1.32246500
C	-0.42750200	0.80271700	-0.96577600
H	1.54277000	1.21431800	-1.79397400
H	0.94027000	0.71558600	-4.14037700
H	-3.15297800	-0.00153000	-2.92357900
H	-2.54434800	0.44337000	-0.57463400
C	-0.01696800	1.10893300	0.46220300
H	0.84464900	1.78683700	0.40310800
C	0.45836400	-0.20062500	1.13405300
H	-0.36239700	-0.92263400	1.21256900
H	1.27513700	-0.67177800	0.57700600
H	0.81752800	0.01311700	2.14593700
C	-1.10787700	1.79915600	1.29494000
H	-0.69463100	2.09608600	2.26382100
H	-1.48942400	2.69681500	0.79651400
H	-1.95236000	1.13067400	1.49775000
C	-1.53106200	0.00495600	-5.15237100
H	-0.85465200	0.51163300	-5.84596000
H	-1.48775900	-1.07130400	-5.36630100
H	-2.55305900	0.34204000	-5.34744200
C	-5.80422300	5.13050200	0.26708100
C	-4.71813400	4.56759600	-0.38982300
C	-4.37154400	4.97932400	-1.68666600
C	-5.18705100	5.91192400	-2.39564000
C	-6.27283600	6.48711600	-1.68364400
C	-6.56874100	6.11413200	-0.38432800
H	-6.04804600	4.82757700	1.28070000
H	-4.09446700	3.83288300	0.10985500
H	-6.90164400	7.21031800	-2.19613100
H	-7.41069000	6.57149800	0.12790900
C	-3.10087600	4.49690900	-2.26172800
O	-2.82335700	3.23756400	-2.04883300
C	-2.06116800	5.50292600	-2.40451900
H	-2.29073200	6.54816900	-2.22896300
S	-0.79259800	5.27511700	-3.44610600
O	-0.16889400	3.87583100	-3.35974800
C	-0.94976200	5.51087800	-5.25887400
H	-1.36938900	6.50433600	-5.43612300
H	0.04041700	5.40240300	-5.70894400
H	-1.65280300	4.74898300	-5.59607500
C	0.44489900	6.51632900	-3.01456100
H	1.30848400	6.36888600	-3.66636400
H	0.01483500	7.51037300	-3.16284500
H	0.70735800	6.35929400	-1.96775200
C	-5.15411200	6.67014400	-8.15240100
C	-4.97960200	6.61104800	-6.77061400
C	-4.59026300	5.41205300	-6.14785200
C	-4.37281200	4.27906000	-6.95087500
C	-4.56008700	4.33652700	-8.33079600
C	-4.95011300	5.53285500	-8.93793400
H	-5.44877300	7.60702900	-8.61698900
H	-5.12662900	7.50890400	-6.17785500
H	-4.07505400	3.35194800	-6.47173900
H	-4.40504800	3.44635700	-8.93469400
H	-5.09285700	5.57961700	-10.01375500
C	-4.36804400	5.31444300	-4.67297300
O	-3.57274700	4.37574000	-4.26834400
C	-5.04833700	6.16577200	-3.79949100
H	-5.75392200	6.86819800	-4.22788200

IM30

Ru	1.02689000	-1.40076400	0.42111700

C	2.87022100	-2.66875200	0.89142000
C	2.43204900	-2.99612800	-0.40600400
C	1.06564700	-3.33115900	-0.65564500
C	0.17789500	-3.43176300	0.47164700
C	0.62010000	-3.10615100	1.77082800
C	1.96758500	-2.66329700	2.00007500
H	3.87973100	-2.29751900	1.03340900
H	3.10548000	-2.86553300	-1.24649100
H	-0.87251400	-3.64609100	0.30369200
H	-0.10190800	-3.07858200	2.57864500
C	2.45893400	-2.26536400	3.38002400
H	3.36592500	-1.66547500	3.23024500
C	2.85804800	-3.54238600	4.15402000
H	1.98686200	-4.18307300	4.33230000
H	3.60767400	-4.12886800	3.61231300
H	3.27951500	-3.26927100	5.12685100
C	1.45820400	-1.41537500	4.17901900
H	1.92399900	-1.08332500	5.11224000
H	1.14565800	-0.52799500	3.61771600
H	0.56132900	-1.98297200	4.45167300
C	0.57280800	-3.60826600	-2.04871700
H	1.16057900	-3.06643800	-2.79463200
H	0.66433800	-4.68165900	-2.26145600
H	-0.47936800	-3.33298400	-2.16155600
C	-3.17798400	1.55454800	3.42034900
C	-2.17217100	1.01281200	2.61622400
C	-2.09139700	1.35767700	1.26866400
C	-3.02727200	2.24287400	0.69813800
C	-4.03328300	2.78083500	1.51880400
C	-4.10409100	2.44407600	2.86833500
H	-3.23654200	1.28667600	4.47122500
H	-1.44858900	0.32055600	3.03452100
H	-4.76654100	3.45599100	1.08520800
H	-4.88656400	2.87011300	3.48979200
C	-0.94078500	0.87091300	0.39810000
O	-0.65109700	-0.45930800	0.71482700
C	0.24217800	1.80531900	0.58598600
H	0.21509600	2.66610400	1.24056800
S	1.52407800	1.68106200	-0.40671800
O	2.02017200	0.22439100	-0.59016200
C	1.49445400	2.20618900	-2.16724600
H	1.25695300	3.27223700	-2.19611700
H	2.46758200	1.99668600	-2.61763200
H	0.70542500	1.63123700	-2.65402100
C	2.82139500	2.75021100	0.26262900
H	3.73021600	2.55406500	-0.31027300
H	2.51767900	3.79431100	0.15620900
H	2.95902700	2.48564600	1.31153600
C	-2.52797700	3.03400000	-5.08490700
C	-2.54496100	2.99347200	-3.69312400
C	-2.11793600	1.84391400	-3.00277500
C	-1.66033600	0.74349200	-3.75143700
C	-1.64552100	0.78890900	-5.14567900
C	-2.07922100	1.93222200	-5.81884100
H	-2.85957600	3.93183700	-5.59873100
H	-2.87479600	3.86630800	-3.13796600
H	-1.34112600	-0.15407000	-3.23198900
H	-1.30222400	-0.07535500	-5.70791300
H	-2.06723600	1.96676800	-6.90429000
C	-2.15326300	1.78288900	-1.52973000
O	-1.31086300	0.82206200	-1.01542900
C	-2.95939800	2.51136200	-0.72574300
H	-3.65386200	3.21931200	-1.16173400

IM31

C	-7.30093600	6.05538100	-2.56761900
C	-6.02207100	5.81260700	-2.07158400
C	-4.99573100	5.33427200	-2.89891000
C	-5.26196300	5.08833800	-4.26631200
C	-6.54772900	5.35572600	-4.74793200
C	-7.56314000	5.83023200	-3.91719900
H	-8.08265700	6.41574000	-1.90468400
H	-5.81450700	5.97238400	-1.01770700
H	-6.75790800	5.18515800	-5.80124700
H	-8.55239200	6.01930500	-4.32535000
C	-3.63453500	5.09892000	-2.31656900
O	-2.91358400	4.13354100	-2.73065200
C	-3.17617800	5.97759900	-1.32904900
H	-3.62892100	6.91074700	-1.02433000
S	-1.61216700	5.76364500	-0.68037900
O	-1.25358300	6.81691900	0.29827900
C	-1.46939500	4.12609800	0.06723200
H	-1.74762300	3.35113400	-0.65396200
H	-0.43666900	4.01040400	0.40680500
H	-2.14991600	4.13298800	0.92146000
C	-0.38890400	5.70309900	-2.01340900
H	0.58965500	5.50880600	-1.56803700
H	-0.69334700	4.92189800	-2.71095800
H	-0.41596300	6.68364300	-2.49267900

C	-0.39821500	2.73294700	-6.57651800
C	-1.54087600	3.27128100	-5.98286900
C	-1.73155200	4.66149600	-5.95025500
C	-0.75433700	5.49700400	-6.51719600
C	0.37697500	4.95729400	-7.12145900
C	0.55719300	3.57125100	-7.15294000
H	-0.25388300	1.65595000	-6.58590600
H	-2.25673400	2.60283100	-5.51633200
H	-0.90938400	6.57044000	-6.47581400
H	1.11923200	5.61373400	-7.56792600
H	1.44131800	3.14729400	-7.62196700
C	-2.92793000	5.32310100	-5.32660600
O	-2.87539700	6.49704000	-4.99361000
C	-4.23255700	4.52878200	-5.22638900
H	-4.65744900	4.52593400	-6.24128900
H	-4.02336400	3.48324200	-4.99240800
C	-2.80502800	-0.64855600	-2.66214900
H	-2.44595600	-0.84352600	-3.67823100
H	-3.85697500	-0.95124200	-2.62719800
H	-2.22682700	-1.23203700	-1.94494100
C	-2.68349500	0.82495200	-2.34282300
O	-2.03774400	1.26506100	-1.40668400
O	-3.37094300	1.58171400	-3.20447000
H	-3.25742700	2.55297100	-2.96541400

TS18

C	-7.49169800	7.21932700	-2.53016000
C	-6.49328700	6.45322300	-1.94780600
C	-5.33538900	6.06936300	-2.65601800
C	-5.19421200	6.45893700	-4.01340500
C	-6.22366600	7.22905500	-4.57684000
C	-7.35212500	7.61504700	-3.86052100
H	-8.36860700	7.50284600	-1.95509400
H	-6.58078000	6.12705000	-0.91801300
H	-6.13101500	7.52796500	-5.61776100
H	-8.11992700	8.21452000	-4.34246700
C	-4.37310100	5.26976700	-1.82838500
O	-4.71888700	4.85085000	-0.71362000
C	-3.00514000	4.94886100	-2.31612500
S	-1.87215900	4.51231600	-1.01101100
O	-0.47268500	4.50130500	-1.48314900
C	-2.03656300	5.65046900	0.38613800
H	-3.07453800	5.65836400	0.71709400
H	-1.35343600	5.30323300	1.16494600
H	-1.72493500	6.63057100	0.01757900
C	-2.38013900	2.90701600	-0.37348300
H	-1.62418600	2.60020300	0.35313400
H	-3.37230100	3.01912100	0.06520800
H	-2.40803000	2.24895300	-1.24896800
C	-0.75385600	5.89594200	-7.82719900
C	-1.72595900	5.89140800	-6.82592400
C	-1.85219100	7.00104200	-5.97400400
C	-0.99346100	8.10102500	-6.13439400
C	-0.03866100	8.10607600	-7.14594900
C	0.08160100	7.00158200	-7.99542600
H	-0.64775000	5.02948700	-8.47412200
H	-2.34180100	5.00857600	-6.68194000
H	-1.09690900	8.94166500	-5.45558900
H	0.61443700	8.96548100	-7.27267700
H	0.83067500	7.00191500	-8.78325700
C	-2.86059500	7.05446300	-4.87099900
O	-2.72927100	7.83687400	-3.93592600
C	-4.05524700	6.10166700	-4.94614000
H	-4.43183500	6.13051900	-5.97555600
H	-3.69913600	5.06486300	-4.83726600
C	-2.54533800	0.87376200	-5.00525600
H	-2.56039700	0.76918700	-6.09194200
H	-3.28163000	0.19626700	-4.55876900
H	-1.56172400	0.58046800	-4.62126100
C	-2.85058800	2.31286800	-4.60022600
O	-3.04813700	3.18019800	-5.46218400
O	-2.88505700	2.51507100	-3.31923700
H	-3.00701100	3.84627600	-2.96021900
H	-2.49108000	5.72795000	-2.87626100

IM32

C	-6.79046900	7.13133700	-3.63292200
C	-6.04292200	6.30422700	-2.80643500
C	-4.84516200	5.70767800	-3.24737300
C	-4.40906600	5.91586600	-4.58238800
C	-5.17246300	6.77139100	-5.38910700
C	-6.34020200	7.37704400	-4.92960600
H	-7.70738500	7.58499200	-3.26786400
H	-6.36302600	6.10457000	-1.78985900
H	-4.84651400	6.96530900	-6.40528600
H	-6.89922400	8.03365200	-5.59092300
C	-4.12708900	4.95110800	-2.17977900
O	-4.70721000	4.58937200	-1.15511800
C	-2.62396400	4.74134100	-2.24726200
S	-1.86240100	4.55822700	-0.61688900

O	-0.39407700	4.69247300	-0.70146200
C	-2.53077900	5.79768400	0.51698600
H	-3.61335800	5.68949200	0.57035500
H	-2.04881400	5.62681400	1.48262300
H	-2.24116600	6.77414200	0.12164800
C	-2.37823500	2.96539500	0.03314000
H	-1.78513700	2.79114000	0.93407900
H	-3.45056700	3.01797900	0.22709900
H	-2.16853800	2.24662000	-0.77526700
C	-5.40482400	2.31555100	-7.68081900
C	-4.67255100	3.19716300	-6.88294800
C	-3.95468600	4.24460900	-7.48373700
C	-3.97977600	4.38794200	-8.88280000
C	-4.71330200	3.50809300	-9.66965500
C	-5.43034700	2.46826100	-9.06733200
H	-5.95371000	1.50383200	-7.21118100
H	-4.65243800	3.03896400	-5.80747600
H	-3.41249500	5.19982100	-9.32634700
H	-4.72852500	3.62748300	-10.74987700
H	-6.00413100	1.77785300	-9.68074100
C	-3.14930600	5.23519900	-6.70583100
O	-2.46853000	6.07890000	-7.27588400
C	-3.18564200	5.22430900	-5.17311600
H	-3.15011900	4.19807100	-4.79183600
H	-2.27051100	5.74949200	-4.87433000
C	-3.56411200	0.19462900	-3.56531000
H	-4.62972500	0.00798600	-3.72129500
H	-3.19405400	-0.39373800	-2.72013900
H	-3.02118700	-0.13939800	-4.45843900
C	-3.29835600	1.68615900	-3.35254700
O	-4.13161100	2.51715400	-3.79205800
O	-2.20944200	1.99638500	-2.75682100
H	-2.38648100	3.71674300	-2.69221100
H	-2.04702400	5.53326100	-2.72955500

IM33

C	-8.06170200	6.91630500	-2.99094100
C	-7.12986900	6.15957800	-2.29582900
C	-5.86340100	5.86150600	-2.83973700
C	-5.49920700	6.38644600	-4.10413800
C	-6.45882200	7.16491000	-4.77462300
C	-7.72132900	7.41703200	-4.24816100
H	-9.03806600	7.11470000	-2.55853000
H	-7.36519100	5.76842400	-1.31185500
H	-6.19773500	7.58418100	-5.74256900
H	-8.43165400	8.01312400	-4.81473200
C	-5.03258700	5.00005700	-1.94776800
O	-5.10665900	5.09339000	-0.72977800
C	-4.29400900	3.80024200	-2.54116000
S	-2.88428900	3.22287600	-1.51374900
O	-3.15605100	1.91992800	-0.86873200
C	-1.46546900	3.14741000	-2.61763000
H	-1.15127900	4.17091600	-2.83494200
H	-0.69859100	2.59254200	-2.07138700
H	-1.79291300	2.65959700	-3.54891600
C	-2.38620100	4.46197100	-0.29557500
H	-1.45146100	4.08028300	0.12378500
H	-2.25262500	5.41542700	-0.80790600
H	-3.17378100	4.52177000	0.45188200
C	-0.27353500	6.62164900	-6.74896300
C	-1.48026900	6.33622600	-6.10973800
C	-1.64530600	6.67468200	-4.75551800
C	-0.59351800	7.29503300	-4.06002300
C	0.59877100	7.59616400	-4.71151800
C	0.75998300	7.25830600	-6.05875400
H	-0.14109400	6.34194000	-7.79035500
H	-2.26308200	5.80023600	-6.64116100
H	-0.73553300	7.55037900	-3.01437800
H	1.40145200	8.09351700	-4.17364300
H	1.69226000	7.48890500	-6.56779000
C	-2.90600700	6.37332900	-4.01416700
O	-2.87684800	6.23611300	-2.78651800
C	-4.17438000	6.22751900	-4.81306100
H	-4.13646600	6.95687000	-5.62923100
H	-4.10199600	5.25621300	-5.37594500
C	-2.65603700	1.91560300	-7.11029400
H	-2.88029600	2.08911000	-8.16526200
H	-3.09533800	0.96365600	-6.79151500
H	-1.57058100	1.82605500	-6.98015200
C	-3.19019900	3.05550500	-6.23457200
O	-3.73156800	4.03841200	-6.79753000
O	-3.03682700	2.91935200	-4.97674700
H	-3.92221400	3.82112600	-3.58358800
H	-4.96647300	2.93546000	-2.46432900

TS19

C	-8.01383600	6.79745000	-3.17894700
C	-7.06887200	6.16262600	-2.38919000
C	-5.77856100	5.85660800	-2.87346800
C	-5.39112800	6.27146100	-4.17671600

C	-6.38127900	6.91408000	-4.95264900
C	-7.66317800	7.16160000	-4.48291200
H	-9.00749700	7.00145000	-2.79090700
H	-7.31211800	5.86355100	-1.37485900
H	-6.11385200	7.23854600	-5.95491400
H	-8.38587600	7.65393300	-5.12867800
C	-4.96613400	5.07239200	-1.90621600
O	-5.04313800	5.24292300	-0.69586000
C	-4.28448800	3.79893600	-2.41533400
S	-2.84752600	3.26488100	-1.40556200
O	-3.10272800	1.97458200	-0.72741400
C	-1.44751300	3.16264200	-2.53316600
H	-1.18487200	4.18030400	-2.82766400
H	-0.64853100	2.67159000	-1.97222400
H	-1.76638500	2.58686900	-3.40710900
C	-2.33477500	4.52839500	-0.22117900
H	-1.42211200	4.13004900	0.23035100
H	-2.16624900	5.45661400	-0.76733200
H	-3.13723000	4.63599900	0.50514700
C	-0.15940500	6.81670800	-6.70128400
C	-1.36351300	6.44649400	-6.10254900
C	-1.56389700	6.65930000	-4.72844700
C	-0.53459700	7.24337000	-3.97308500
C	0.65955700	7.62903400	-4.57784700
C	0.85067500	7.41529100	-5.94517200
H	-0.00955900	6.63481700	-7.76218600
H	-2.13037600	5.95750300	-6.69512500
H	-0.69572100	7.40224200	-2.91159000
H	1.44115800	8.09674900	-3.98468800
H	1.78336100	7.71107900	-6.41835700
C	-2.83212800	6.26775400	-4.02196700
O	-2.76418700	6.01870700	-2.79536800
C	-4.04856900	6.15793300	-4.80335500
H	-3.99758000	6.80369000	-5.68360400
H	-3.95873500	5.01791800	-5.53847300
C	-3.14343100	2.03990000	-7.27197900
H	-4.06499400	1.89245300	-7.84442400
H	-2.78801400	1.08246300	-6.88592800
H	-2.40029800	2.45691700	-7.96088600
C	-3.39394800	3.01871400	-6.13263400
O	-3.89741300	4.15058900	-6.49669600
O	-3.11766000	2.70508500	-4.95941300
H	-3.95370700	3.76259900	-3.45968300
H	-4.97395600	2.96192800	-2.24404900

IM34

C	-8.02031800	6.80708300	-3.16434400
C	-7.07310800	6.20670200	-2.35285500
C	-5.77408600	5.90516800	-2.81826400
C	-5.37137000	6.30707100	-4.12904700
C	-6.37950100	6.89867900	-4.93625900
C	-7.66470400	7.13516400	-4.48072300
H	-9.02065500	7.00582600	-2.79121500
H	-7.32098700	5.91934600	-1.33600000
H	-6.10932200	7.20492800	-5.94400200
H	-8.39279100	7.59394000	-5.14545500
C	-4.97551400	5.10736500	-1.86242000
O	-5.08542500	5.20781300	-0.64464600
C	-4.26000100	3.86922900	-2.41455900
S	-2.81082300	3.35115300	-1.41656000
O	-3.05239400	2.04800900	-0.75592400
C	-1.41368400	3.25881100	-2.55055900
H	-1.16839400	4.27785600	-2.85336000
H	-0.60628200	2.77592600	-1.99449500
H	-1.72746900	2.65959100	-3.40807100
C	-2.31275700	4.60162100	-0.21366000
H	-1.42068800	4.18502100	0.26182400
H	-2.12195900	5.52790400	-0.75575600
H	-3.13772700	4.71136500	0.48743100
C	-0.13125600	6.88347500	-6.62496200
C	-1.33839400	6.50331900	-6.03888900
C	-1.55410600	6.67953300	-4.66194800
C	-0.52044100	7.23678100	-3.89183600
C	0.68085000	7.62947700	-4.47957200
C	0.88135200	7.45342900	-5.85024900
H	0.02000500	6.72974600	-7.69049900
H	-2.10369400	6.04229500	-6.65559500
H	-0.68601400	7.36756000	-2.82745800
H	1.46170300	8.07450800	-3.86769100
H	1.81908800	7.75374600	-6.31045400
C	-2.82936300	6.28335200	-3.95855700
O	-2.73299500	5.99952200	-2.71872700
C	-4.03076300	6.26854100	-4.69104300
H	-3.97107500	6.71490400	-5.68150200
H	-3.92493800	4.62823600	-5.85382400
C	-3.28685900	1.86831300	-7.43657700
H	-4.25756300	1.75203100	-7.92927000
H	-2.92721500	0.90049100	-7.08609200
H	-2.59331900	2.27782700	-8.17864900

C	-3.41254600	2.82155400	-6.27265600
O	-3.89065500	4.01048900	-6.64419200
O	-3.11556800	2.54048100	-5.12115800
H	-3.93585000	3.91905600	-3.45296300
H	-4.92031600	3.00282100	-2.27926300

IM35

C	-8.04871100	6.81102300	-3.14215000
C	-7.11325700	6.19735700	-2.32874600
C	-5.80940000	5.90329000	-2.78738400
C	-5.38802400	6.33119600	-4.08653000
C	-6.39232100	6.92133800	-4.90278000
C	-7.68020500	7.15101400	-4.45476100
H	-9.05337600	7.00627100	-2.77888200
H	-7.37311200	5.88650300	-1.32174500
H	-6.11180000	7.23803200	-5.90439800
H	-8.40462200	7.61388900	-5.12084700
C	-5.02309600	5.05973600	-1.87443200
O	-5.17332100	5.03401600	-0.65587500
C	-4.21849600	3.91648400	-2.51277200
S	-2.76239500	3.42513000	-1.51788700
O	-2.95334900	2.07443400	-0.93884600
C	-1.34030100	3.42557500	-2.63021000
H	-1.14172600	4.45295600	-2.93589700
H	-0.51449600	2.98399500	-2.06679000
H	-1.59301500	2.78980500	-3.48154400
C	-2.33053000	4.62139300	-0.23695300
H	-1.47483800	4.17226600	0.27426100
H	-2.10659700	5.56831400	-0.72816800
H	-3.20020100	4.70262500	0.41344700
C	-0.13953400	6.71554800	-6.56414800
C	-1.35472200	6.39682600	-5.95884600
C	-1.57329100	6.66369700	-4.59668200
C	-0.52906300	7.24583600	-3.85882500
C	0.68163400	7.57664400	-4.46584700
C	0.88283400	7.31214000	-5.82230700
H	0.01137500	6.49257000	-7.61752800
H	-2.13362200	5.91659300	-6.54406600
H	-0.69468700	7.44491100	-2.80486700
H	1.46979100	8.04331800	-3.87978300
H	1.82803500	7.56374800	-6.29622000
C	-2.85671100	6.32470700	-3.88168400
O	-2.76148000	6.07660100	-2.62324900
C	-4.04434800	6.31427800	-4.61352600
H	-3.97203600	6.61807500	-5.65303200
H	-3.89728900	4.11513300	-3.53724200
H	-4.82092600	3.00036900	-2.46238600

IV. NMR Spectra

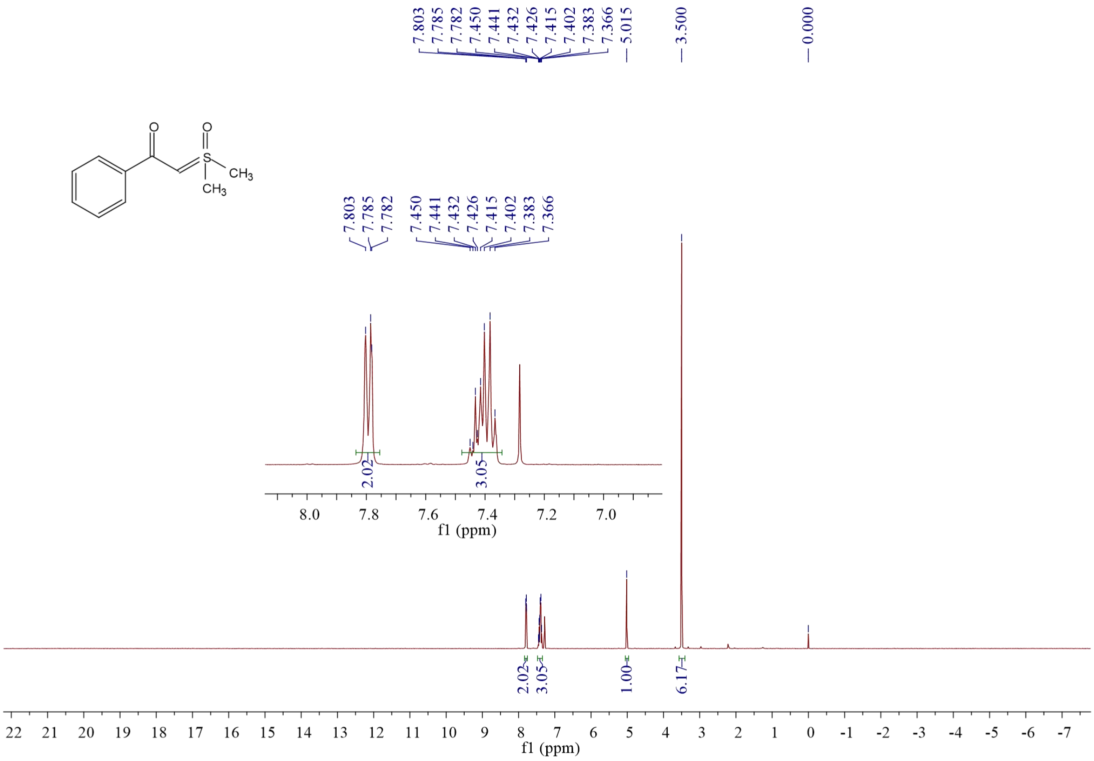

^{1}H NMR (400 Hz, $CDCl_3$): **1a**

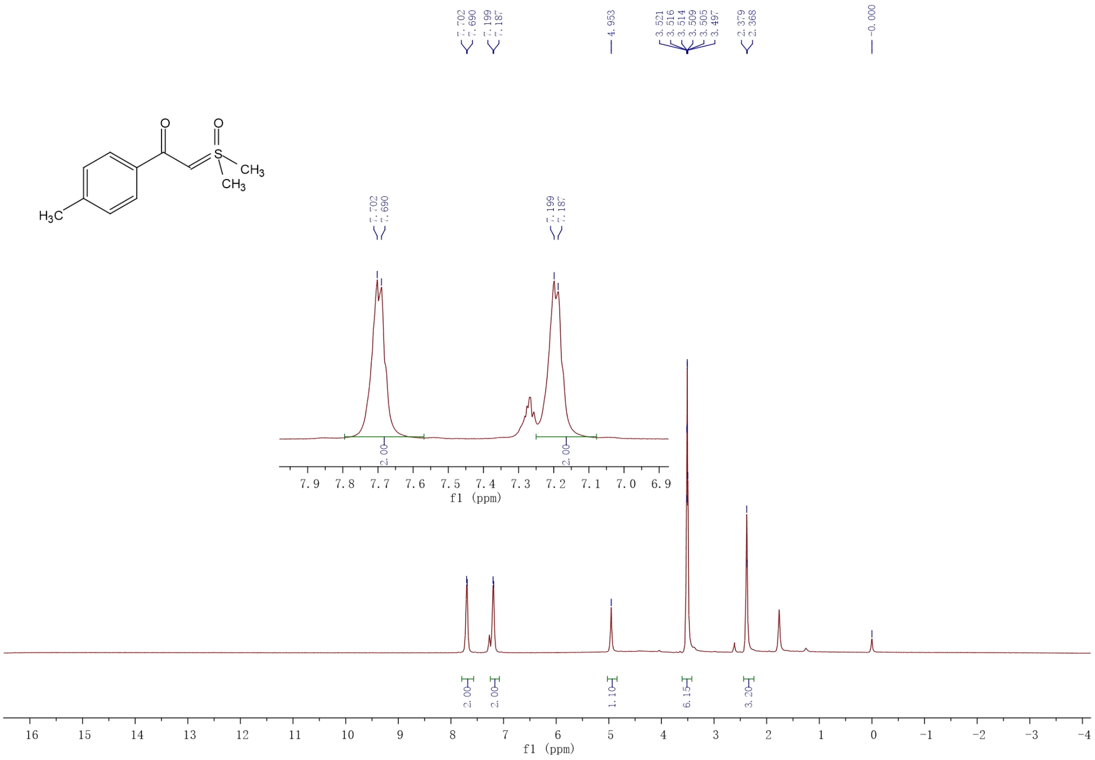

^{1}H NMR (500 Hz, $CDCl_3$): **1b**

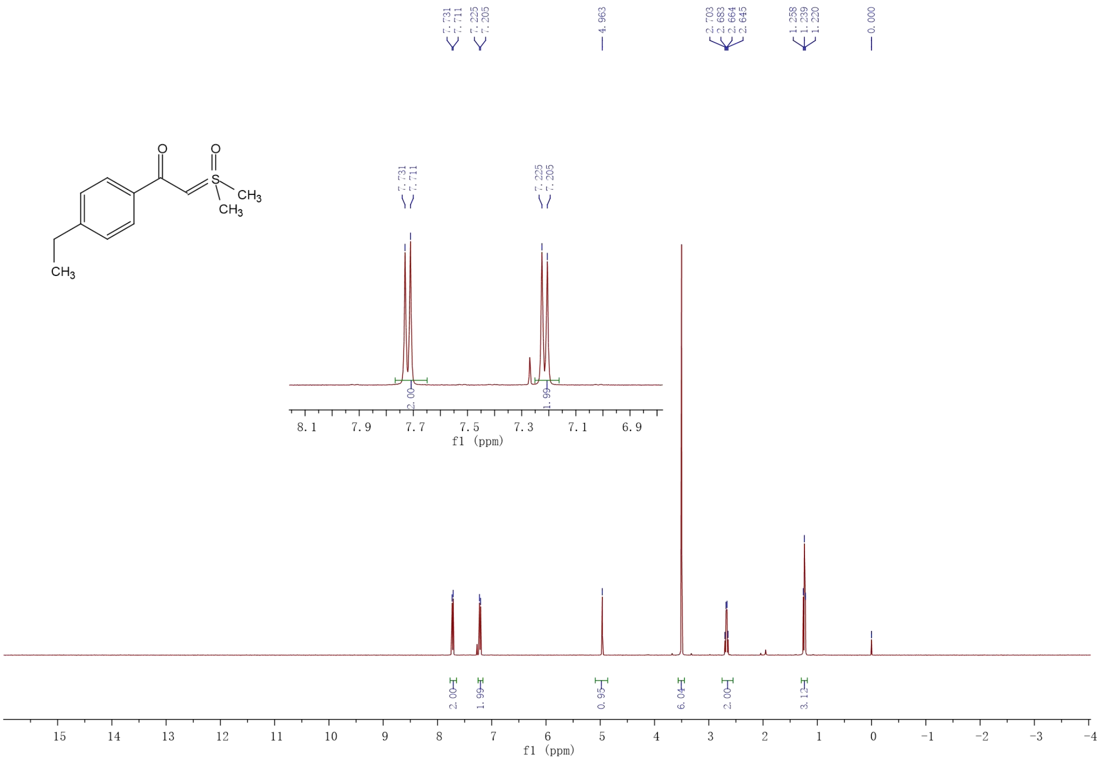

^{1}H NMR (400 Hz, $CDCl_3$): **1c**

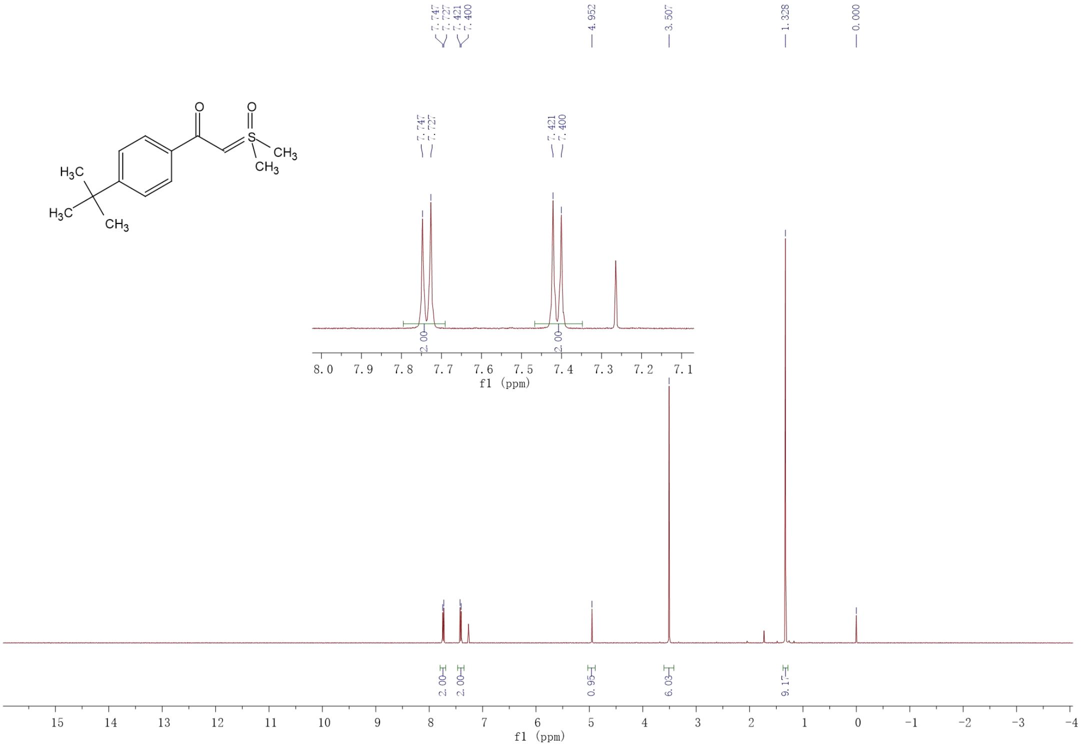

^{1}H NMR (400 Hz, $CDCl_3$): **1d**

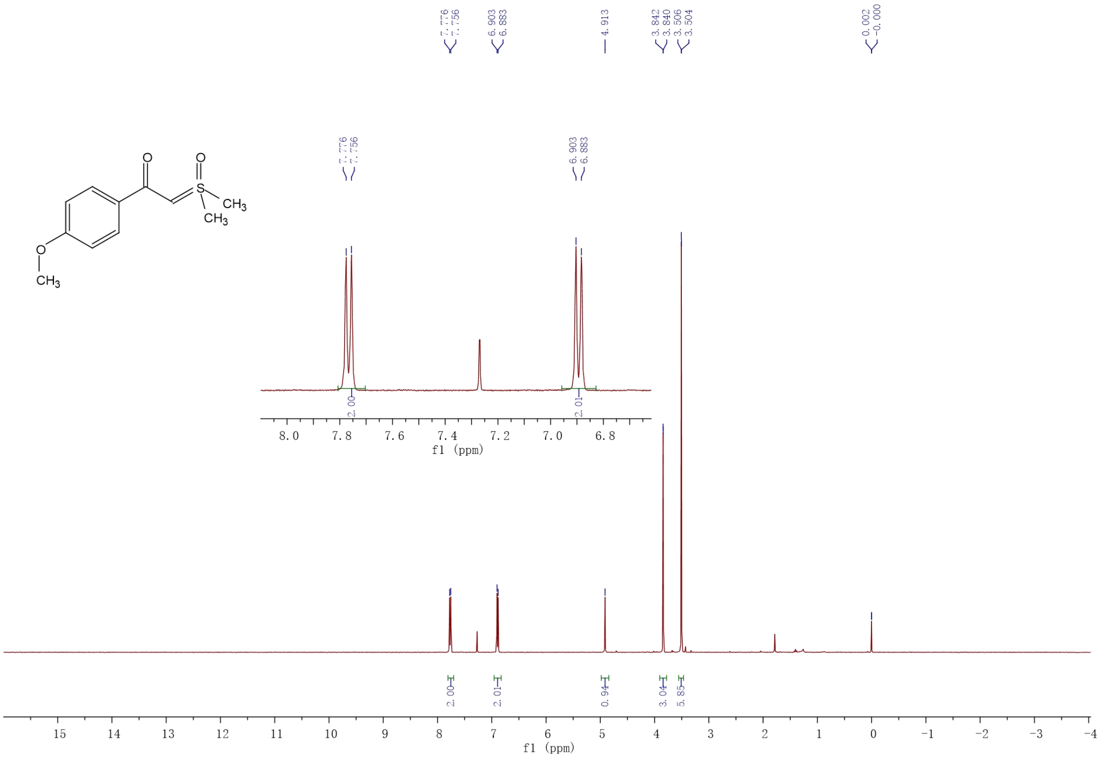

^{1}H NMR (400 Hz, $CDCl_3$): **1e**

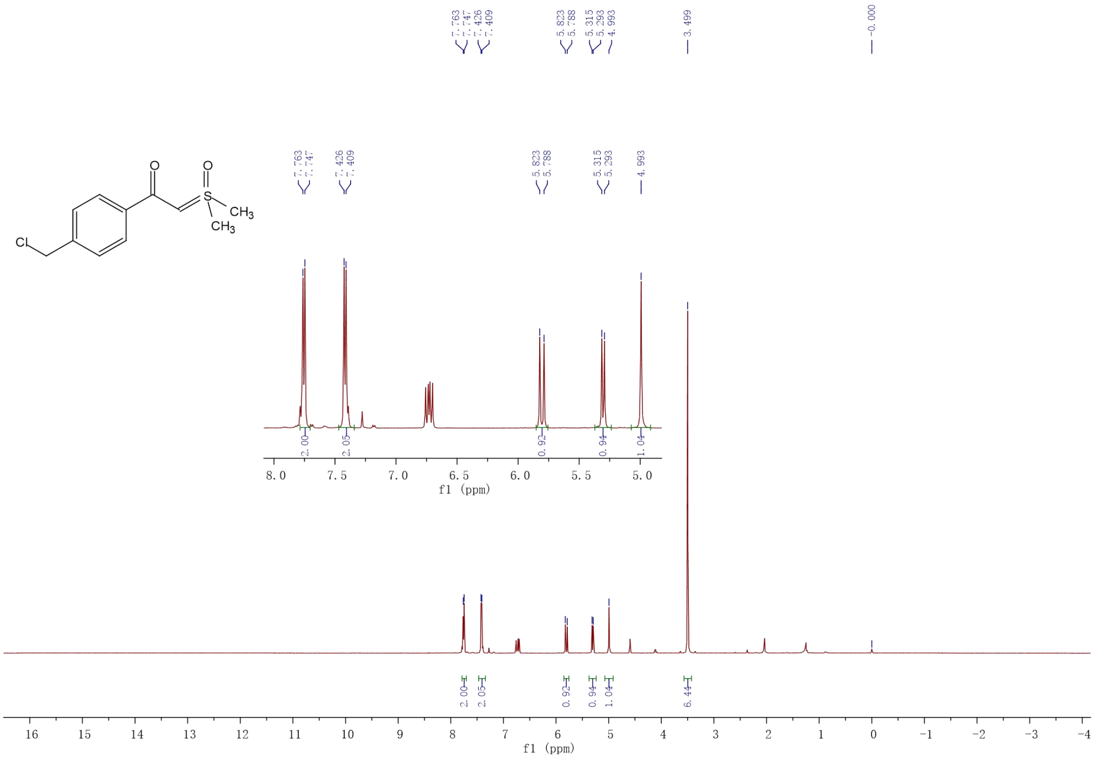

^{1}H NMR (500 Hz, $CDCl_3$):

1f

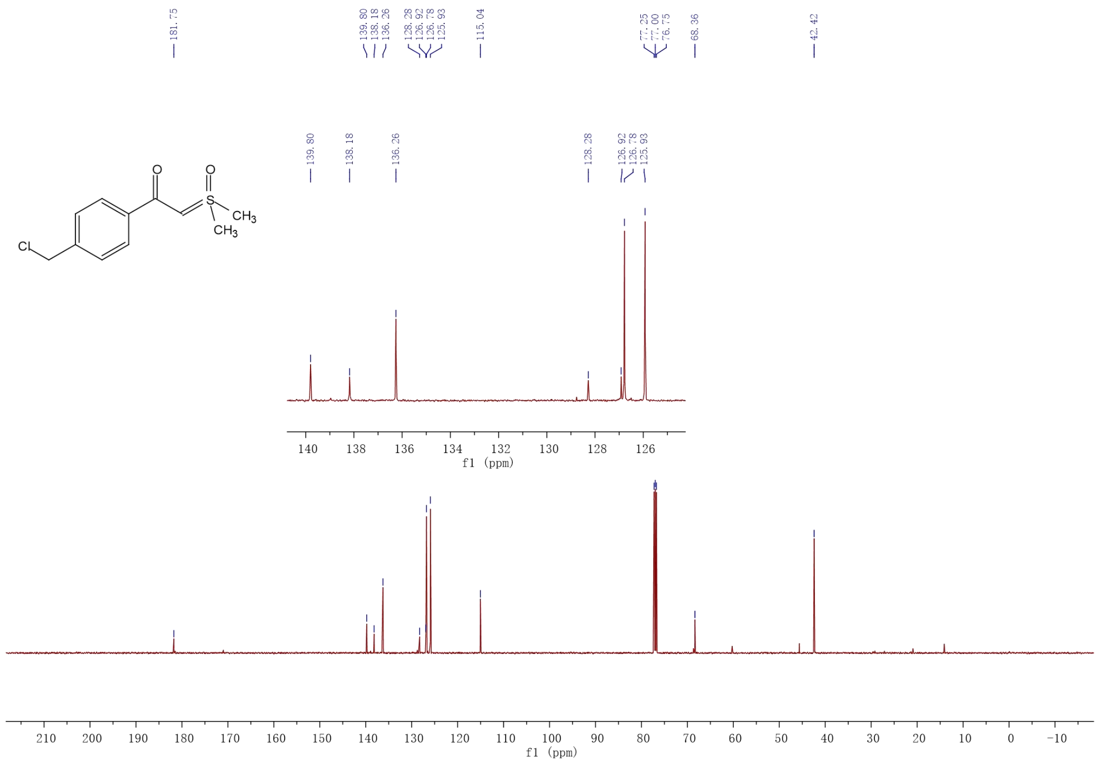

^{13}C NMR (500 Hz, $CDCl_3$): **1f**

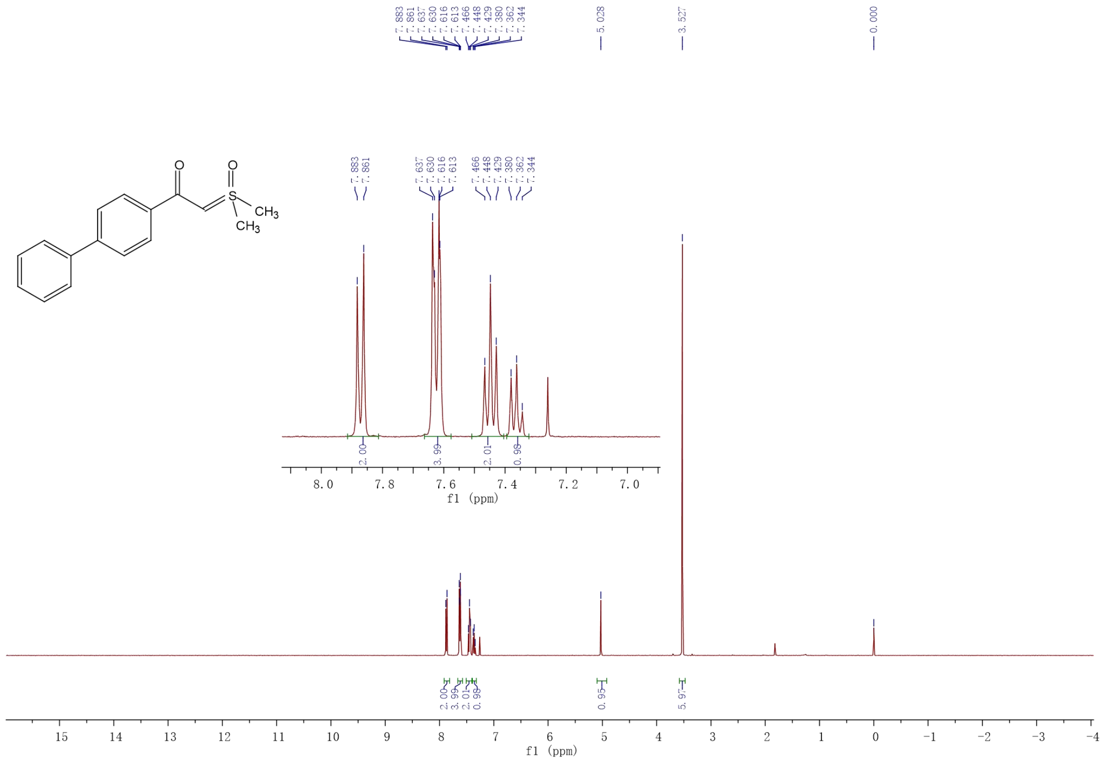

^{1}H NMR (400 Hz, $CDCl_3$): **1g**

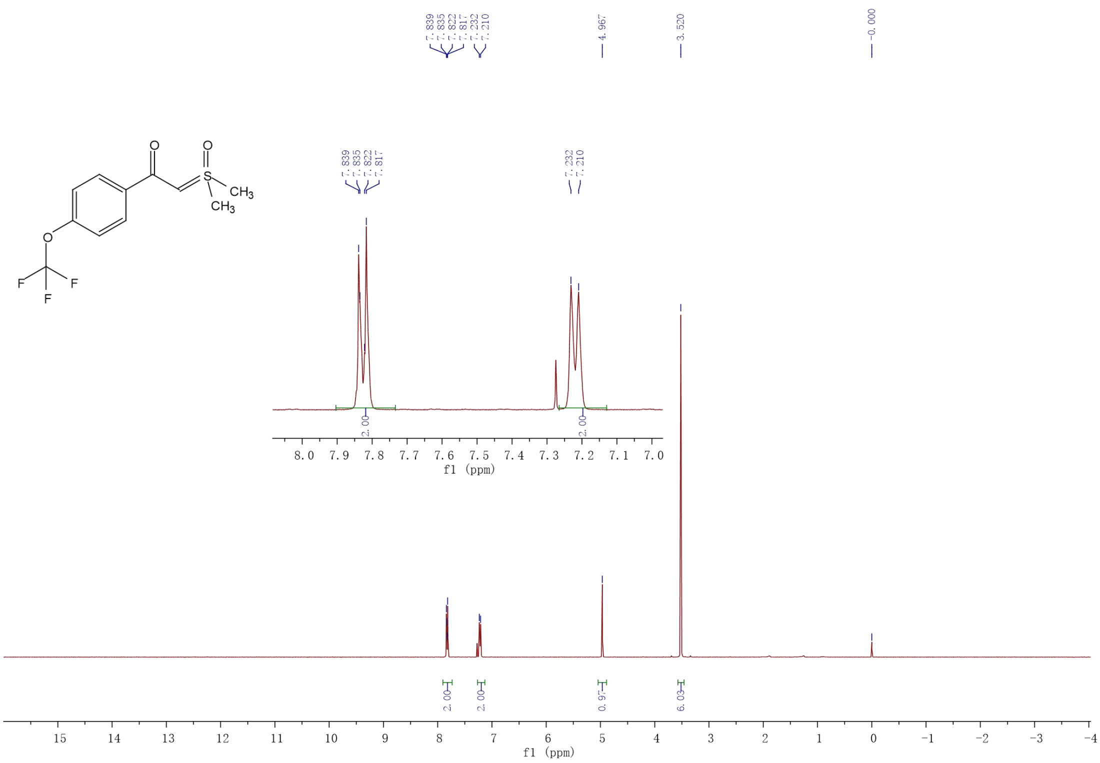

^{1}H NMR (400 Hz, $CDCl_3$): **1h**

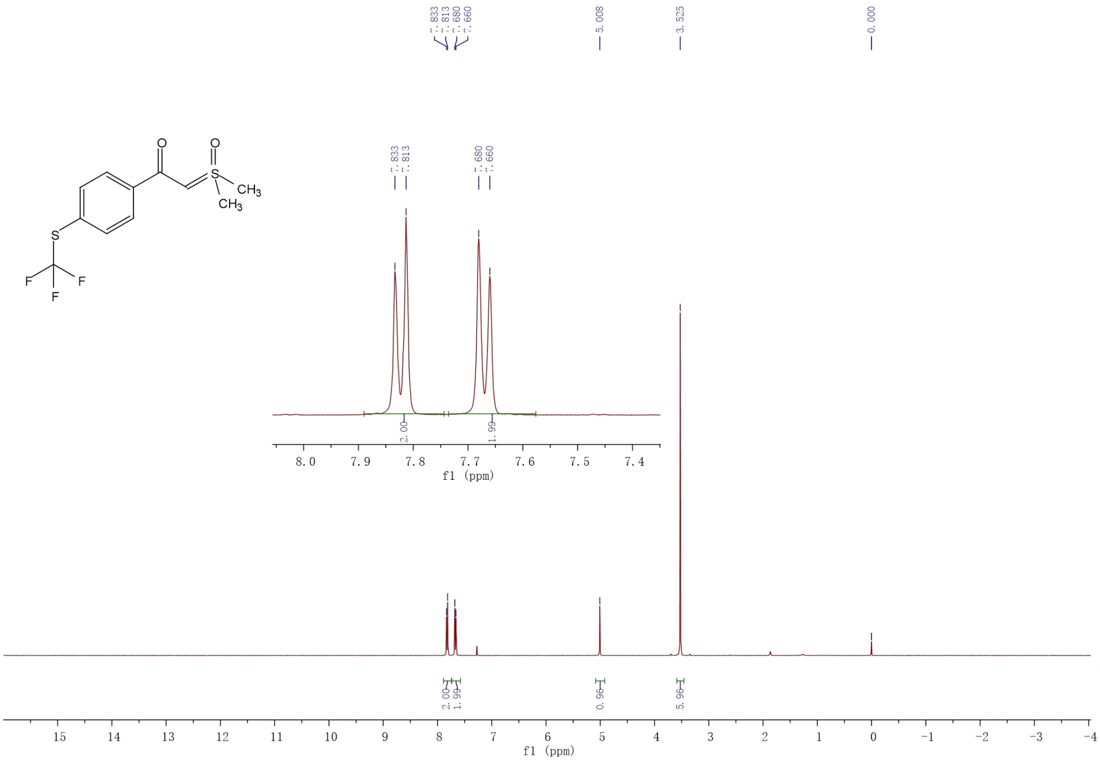

^{1}H NMR (400 Hz, $CDCl_3$): **1i**

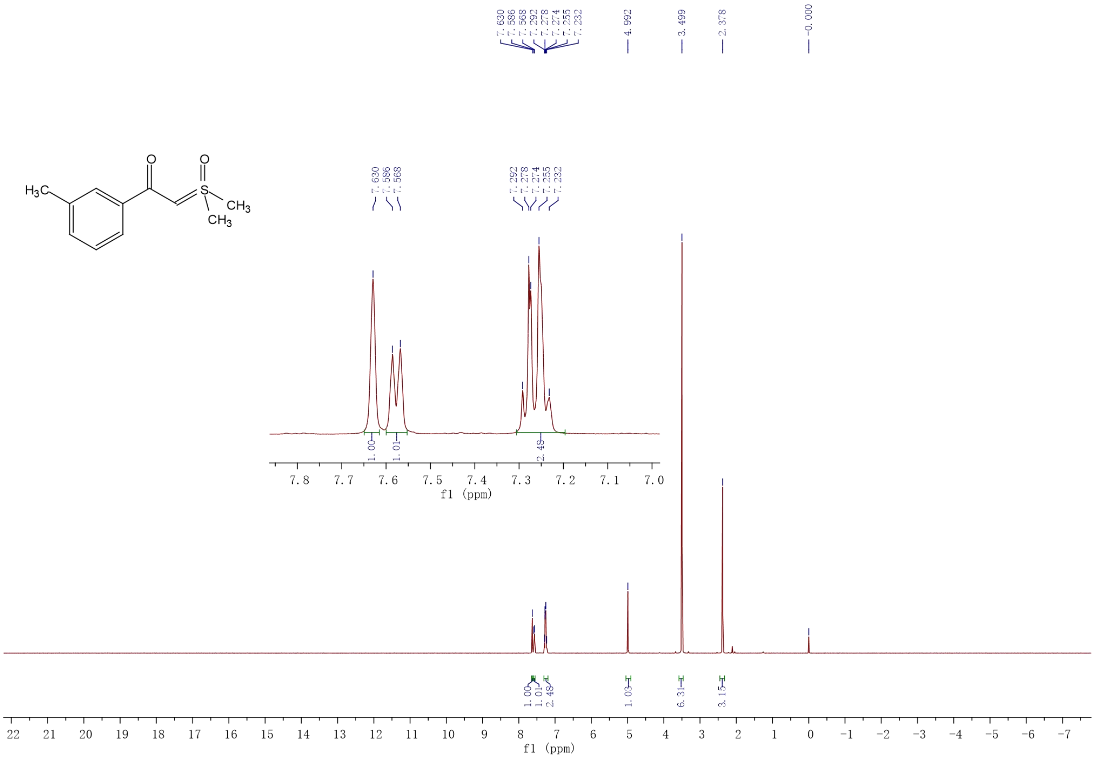

^{1}H NMR (400 Hz, $CDCl_3$): **1j**

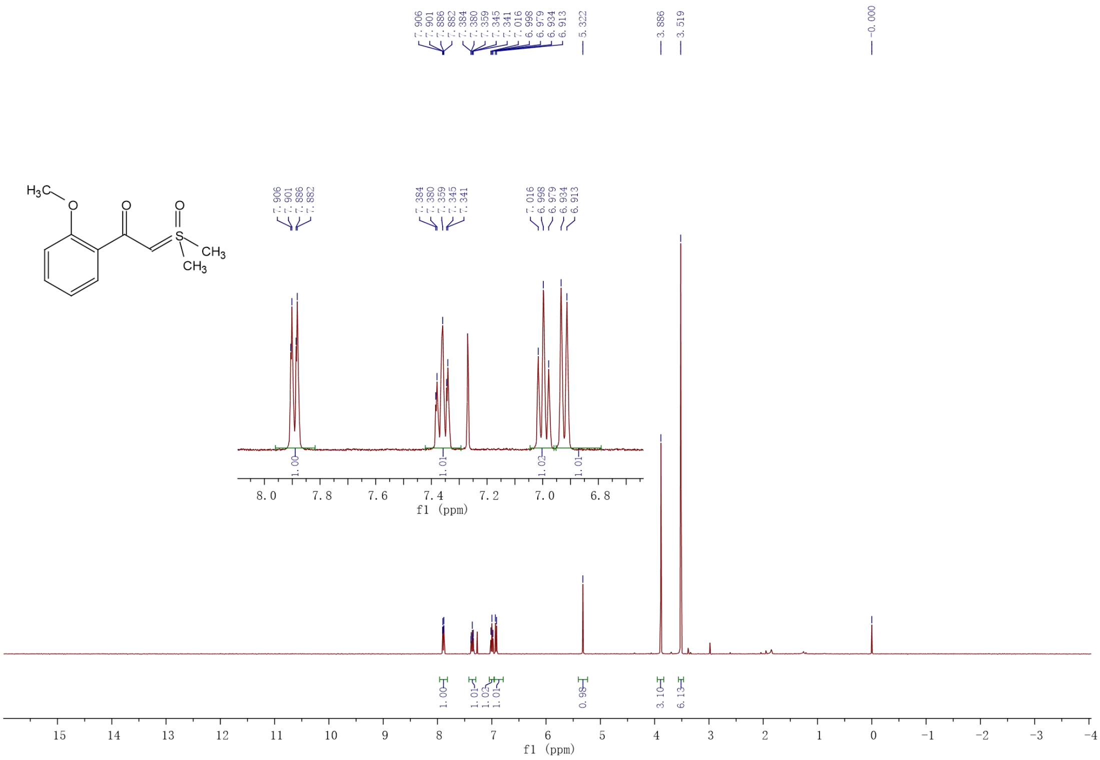

^{1}H NMR (400 Hz, $CDCl_3$): **1k**

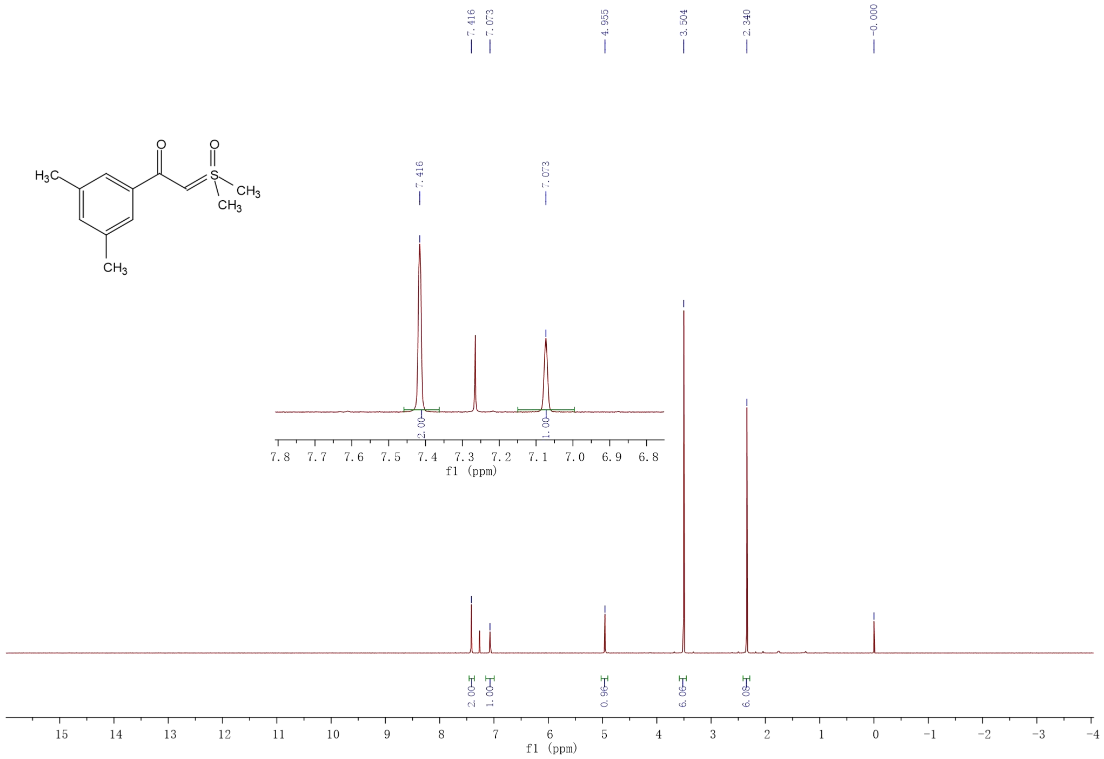

^{1}H NMR (400 Hz, $CDCl_3$): **1l**

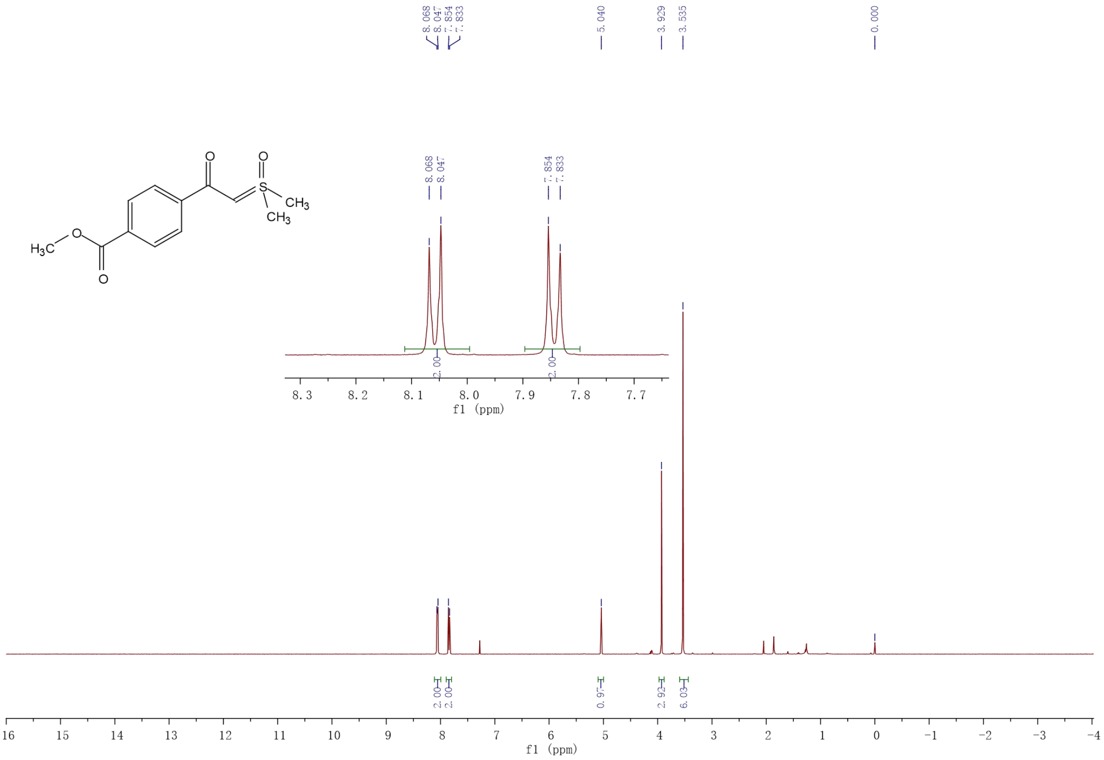

^{1}H NMR (400 Hz, $CDCl_3$): **1m**

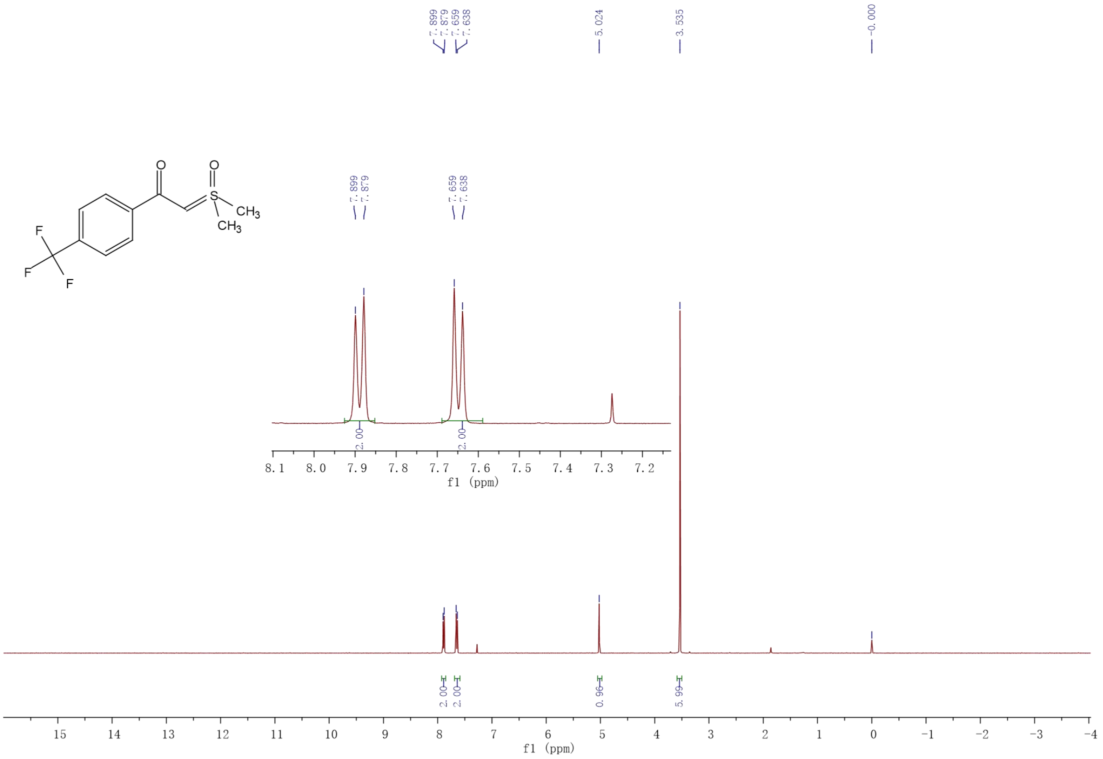

^{1}H NMR (400 Hz, $CDCl_3$): **1n**

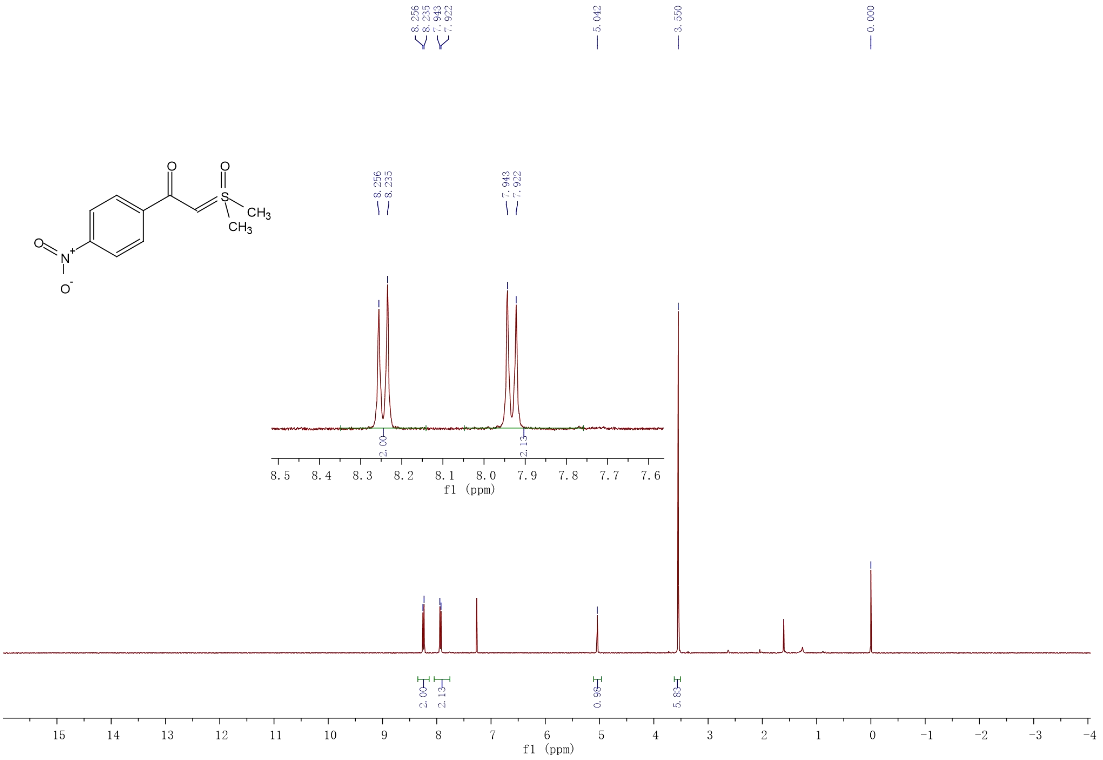

^{1}H NMR (400 Hz, $CDCl_3$):

1o

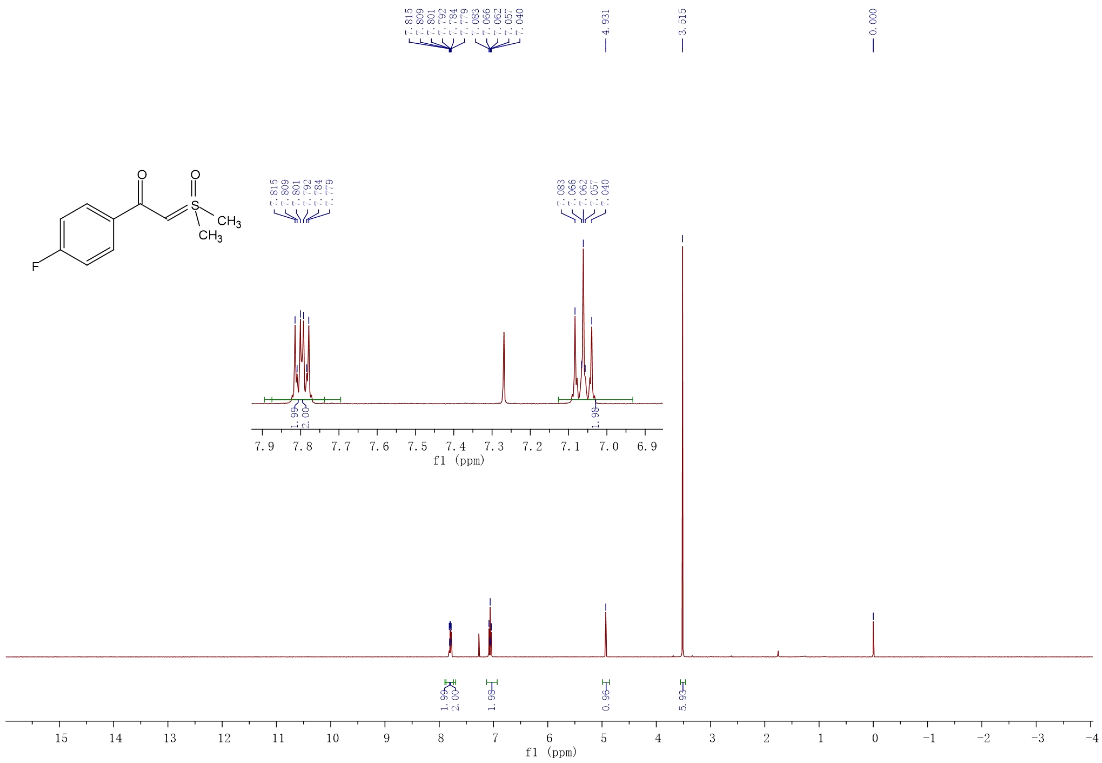

^{1}H NMR (400 Hz, $CDCl_3$): **1p**

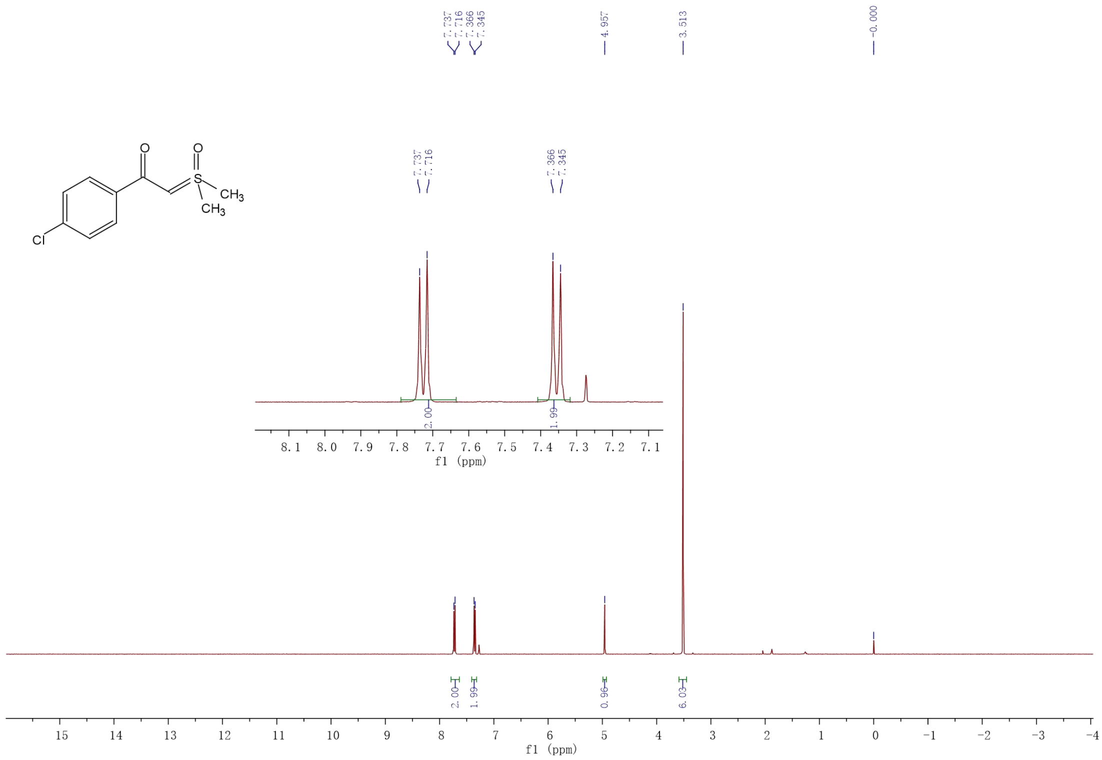

^{1}H NMR (400 Hz, $CDCl_3$): **1q**

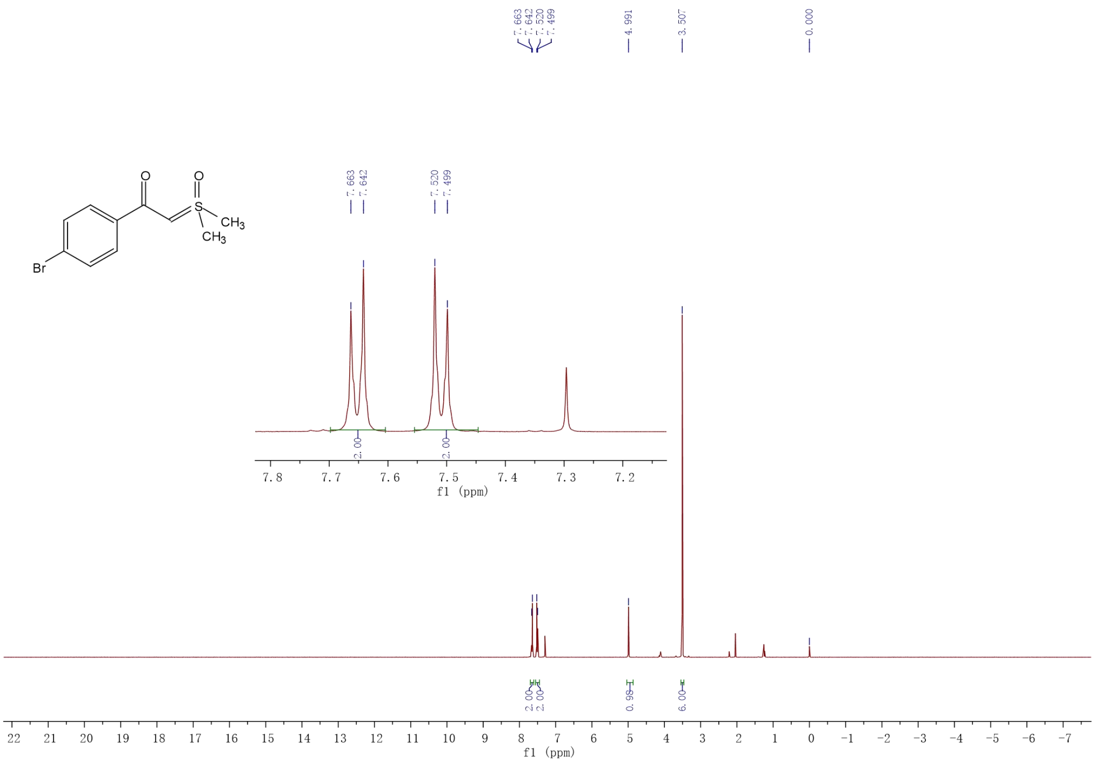

^{1}H NMR (400 Hz, $CDCl_3$): **1r**

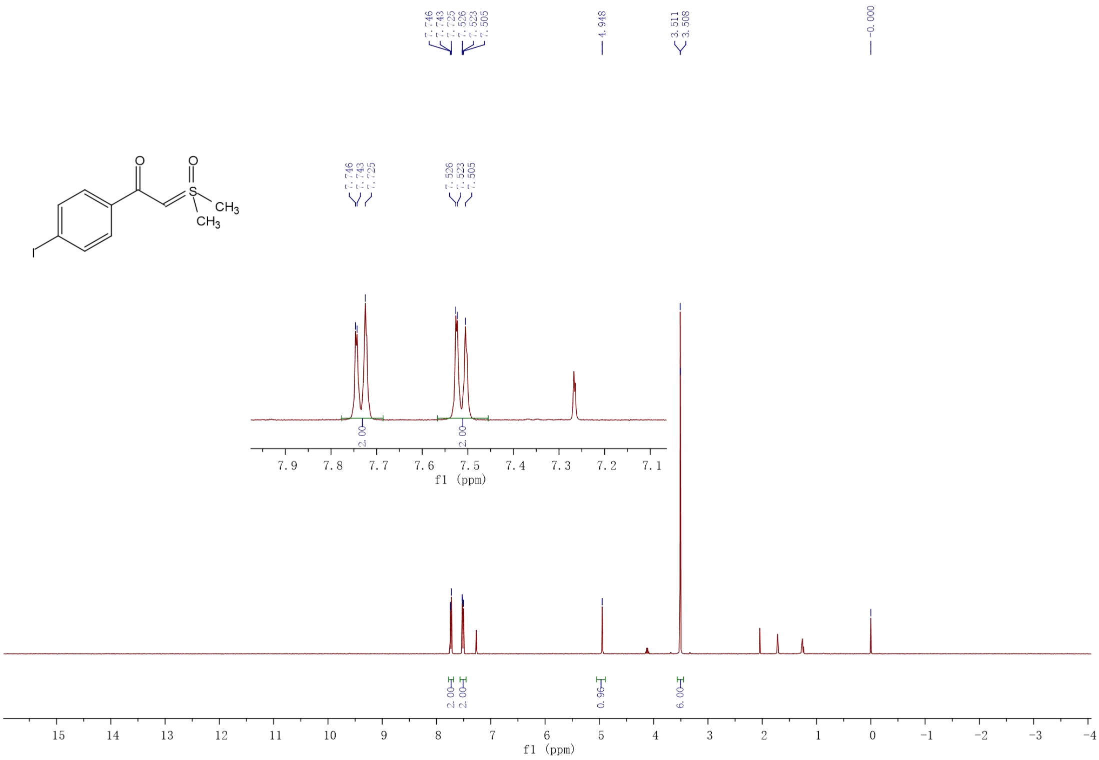

^{1}H NMR (400 Hz, $CDCl_3$): **1s**

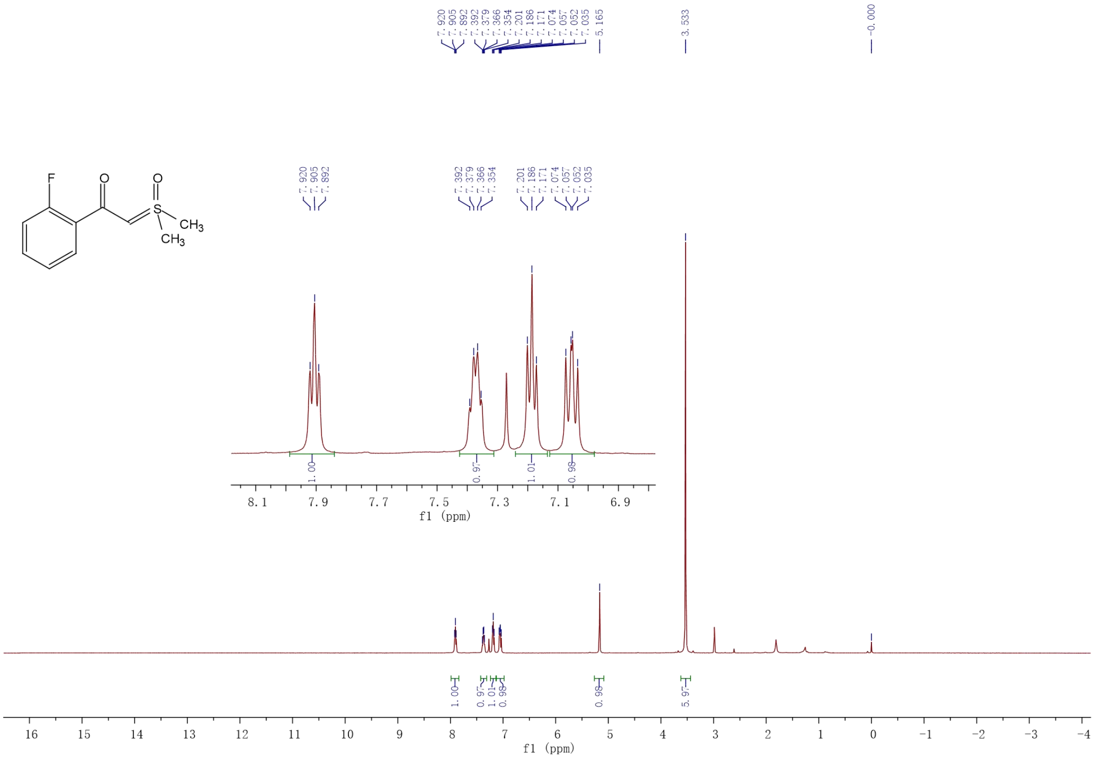

^{1}H NMR (500 Hz, $CDCl_3$): **1t**

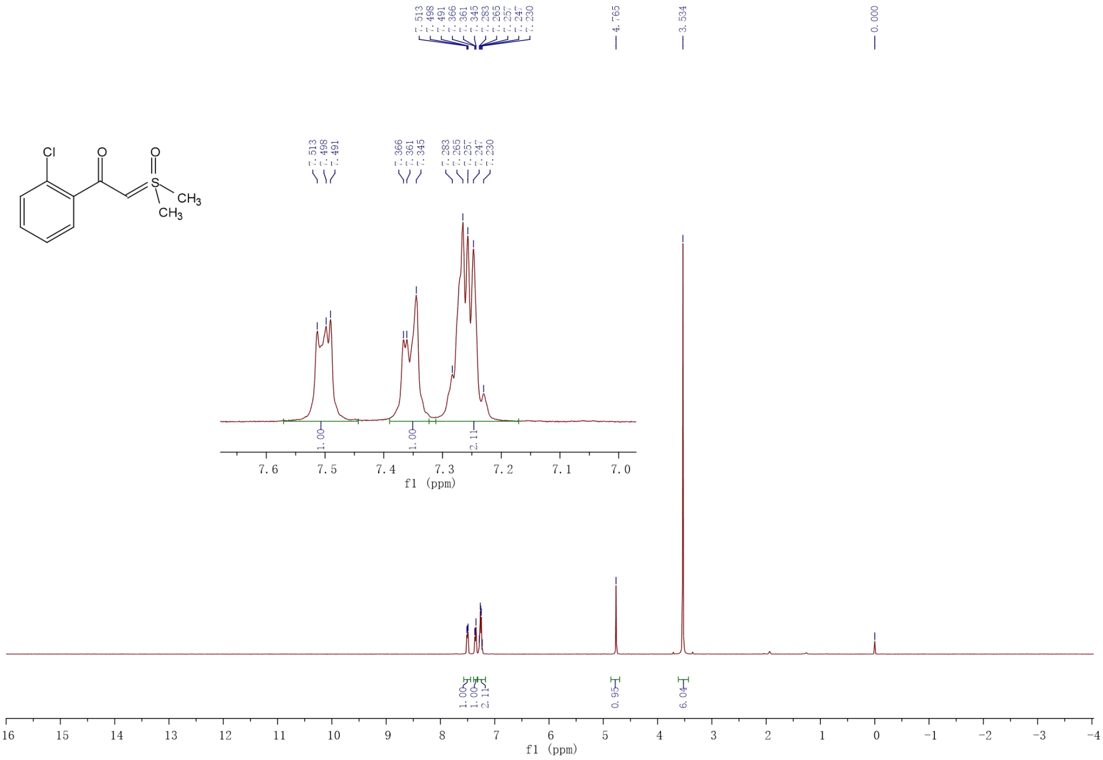

^{1}H NMR (400 Hz, $CDCl_3$): **1u**

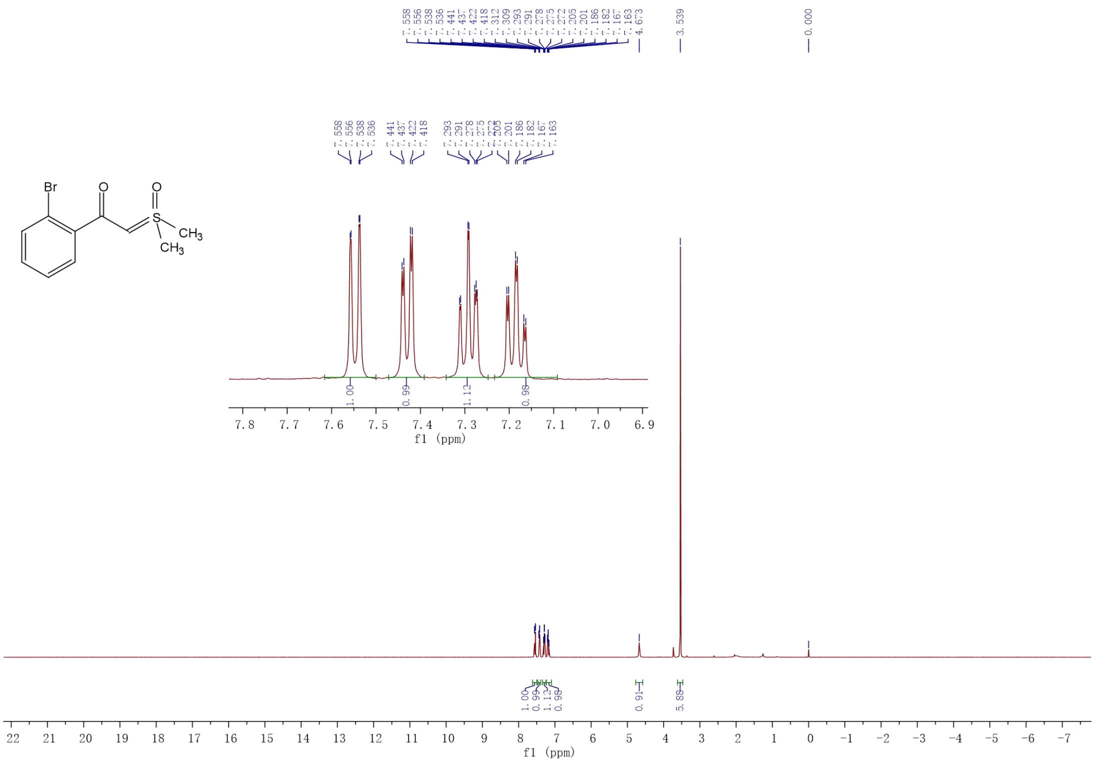

^{1}H NMR (400 Hz, $CDCl_3$): **1v**

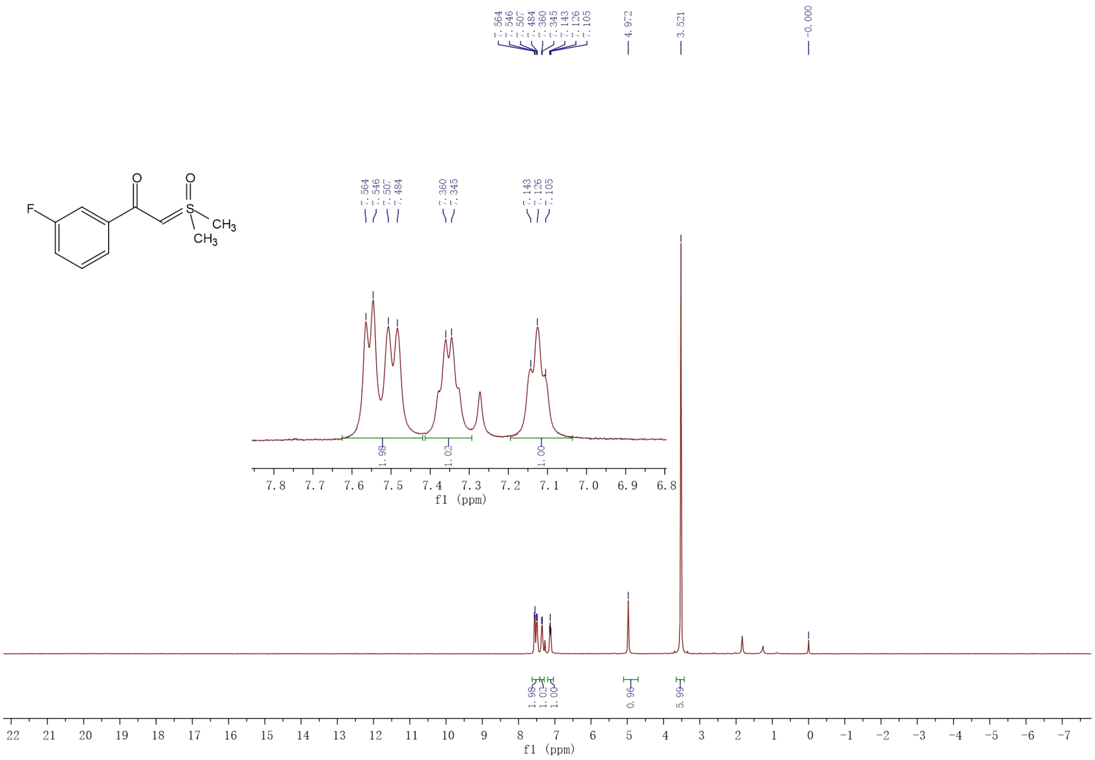

^{1}H NMR (400 Hz, $CDCl_3$): **1w**

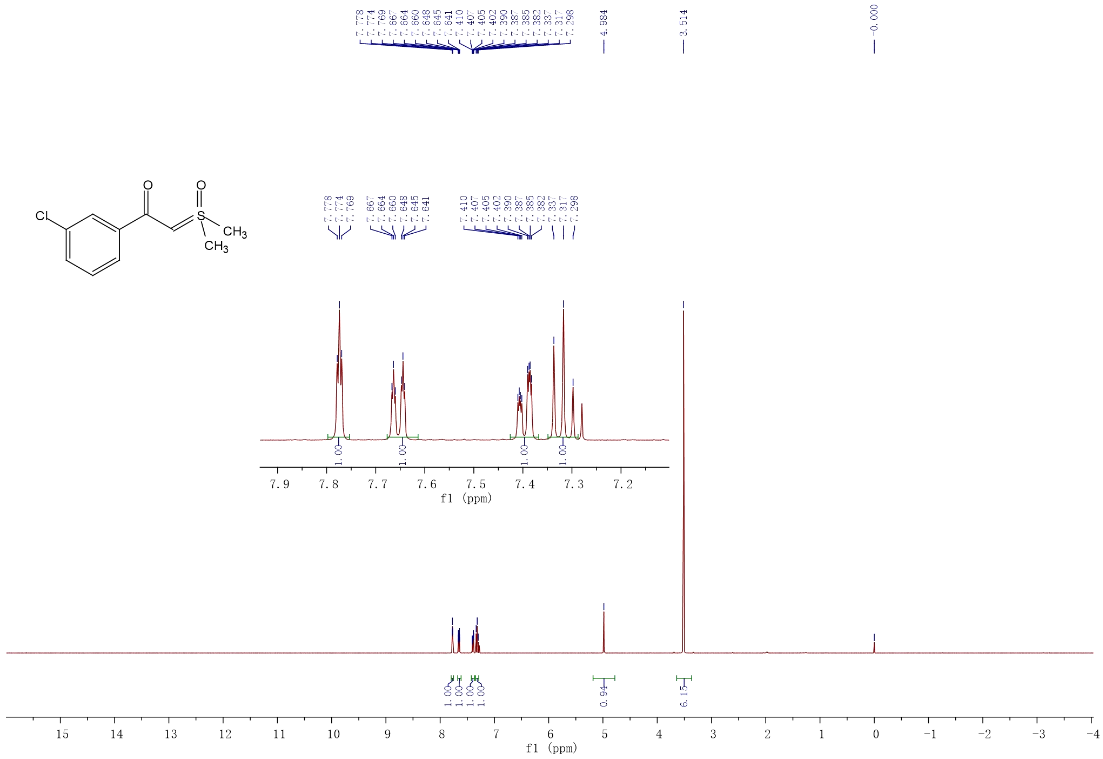

^{1}H NMR (400 Hz, $CDCl_3$): **1x**

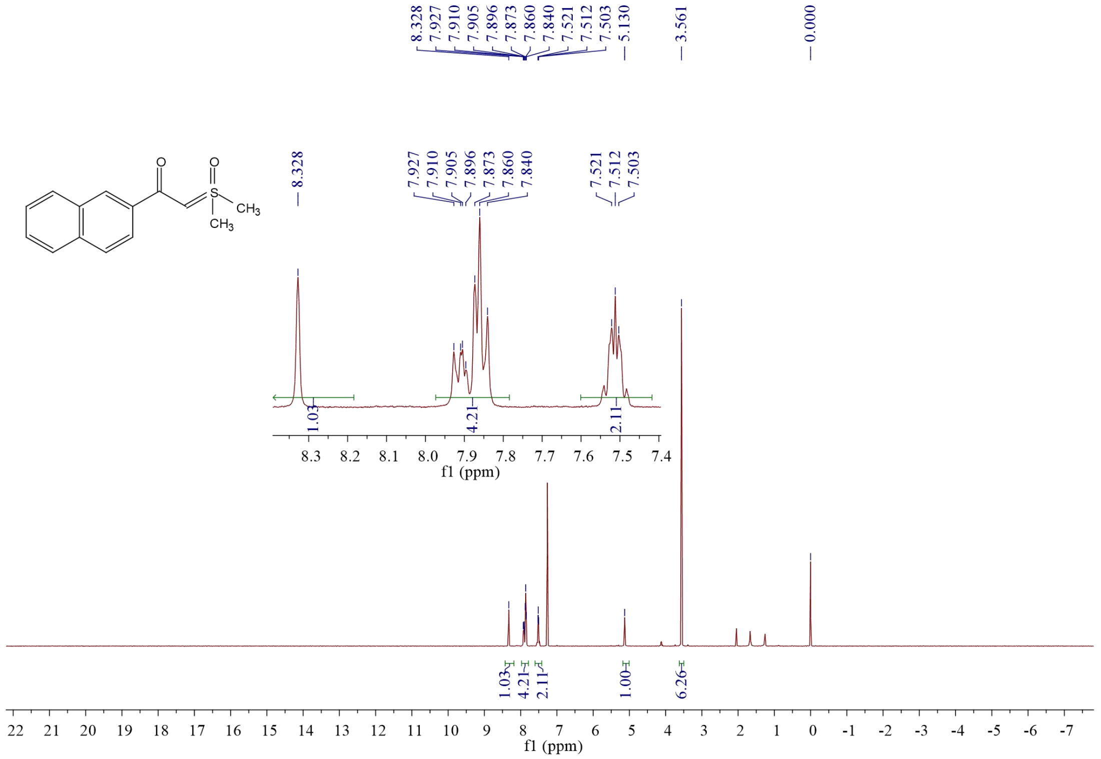

^{1}H NMR (400 Hz, $CDCl_3$): **1y**

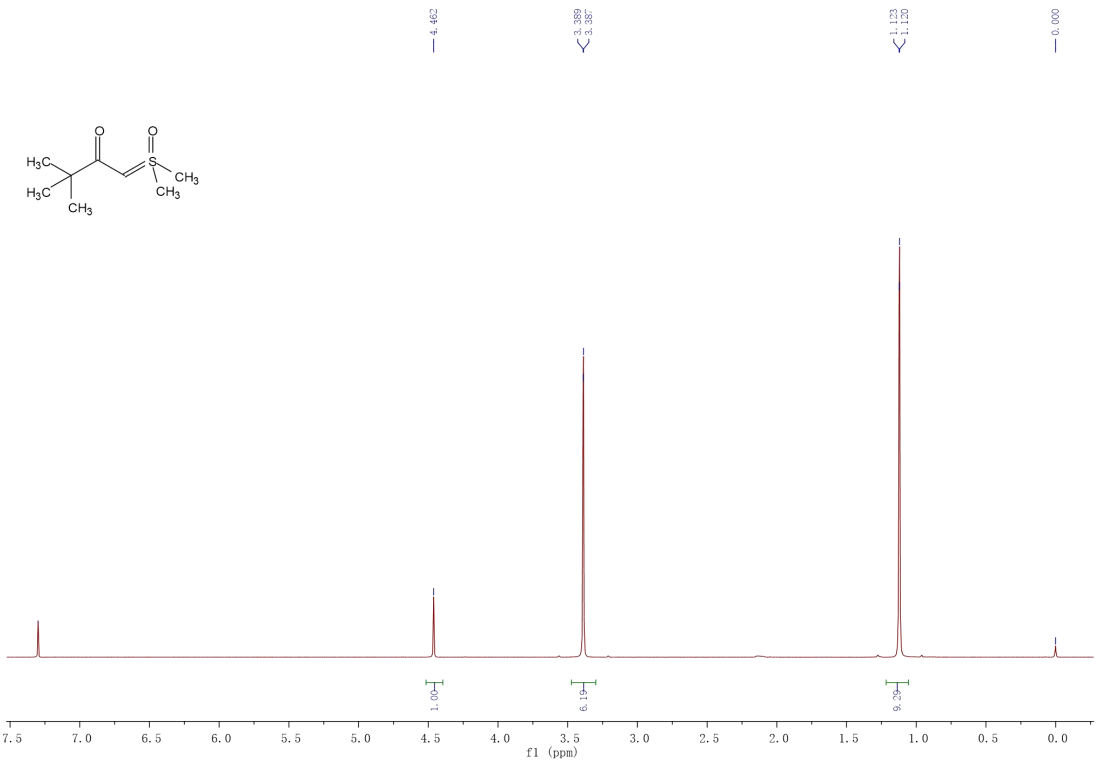

^{1}H NMR (400 Hz, $CDCl_3$): **1z**

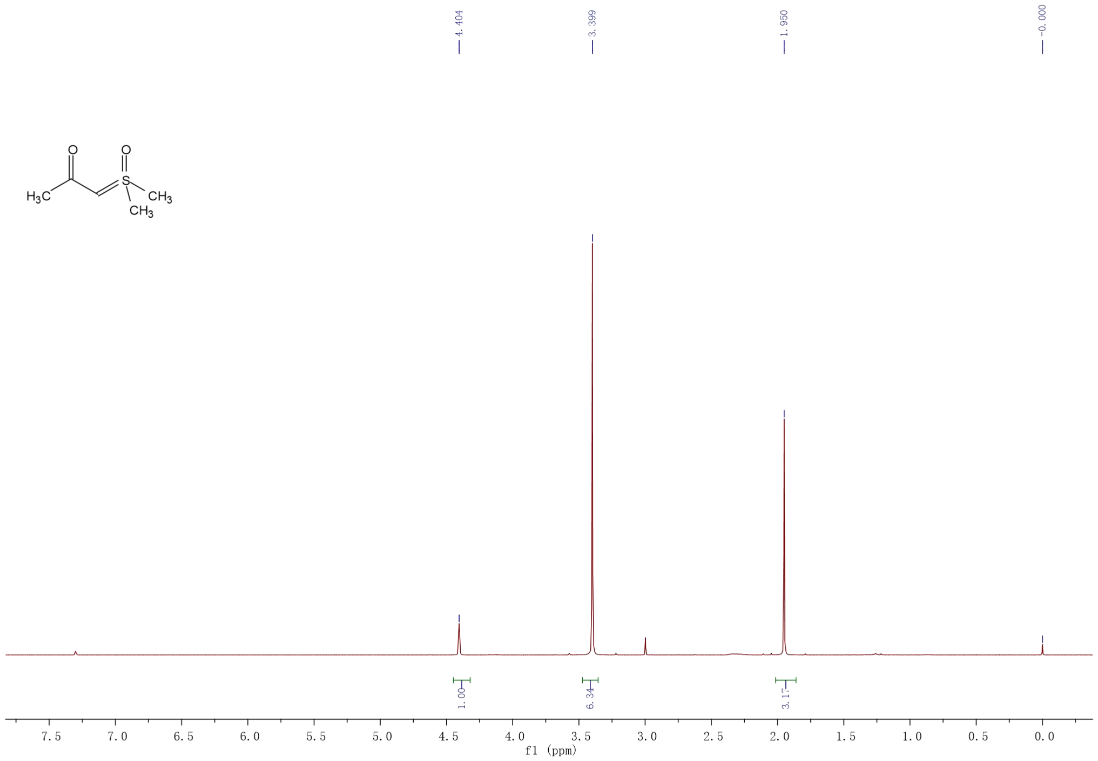

^{1}H NMR (400 Hz, $CDCl_3$): **1aa**

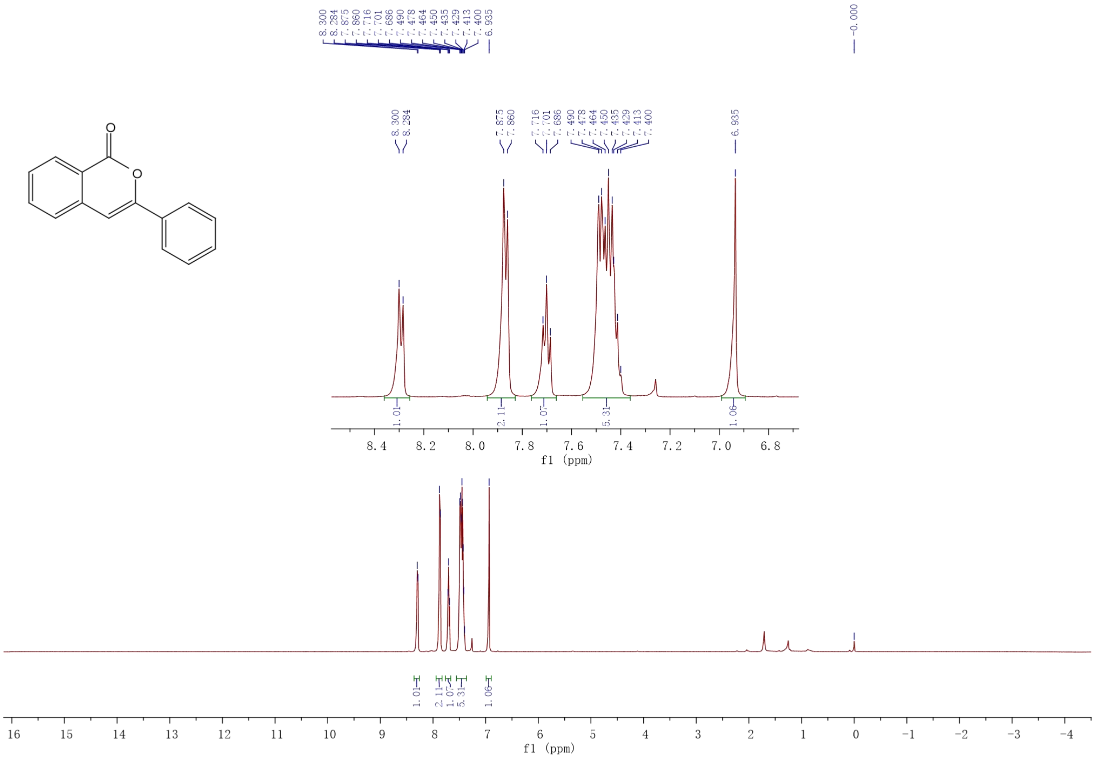

^{1}H NMR (500 Hz, CDCl$_3$): **2a**

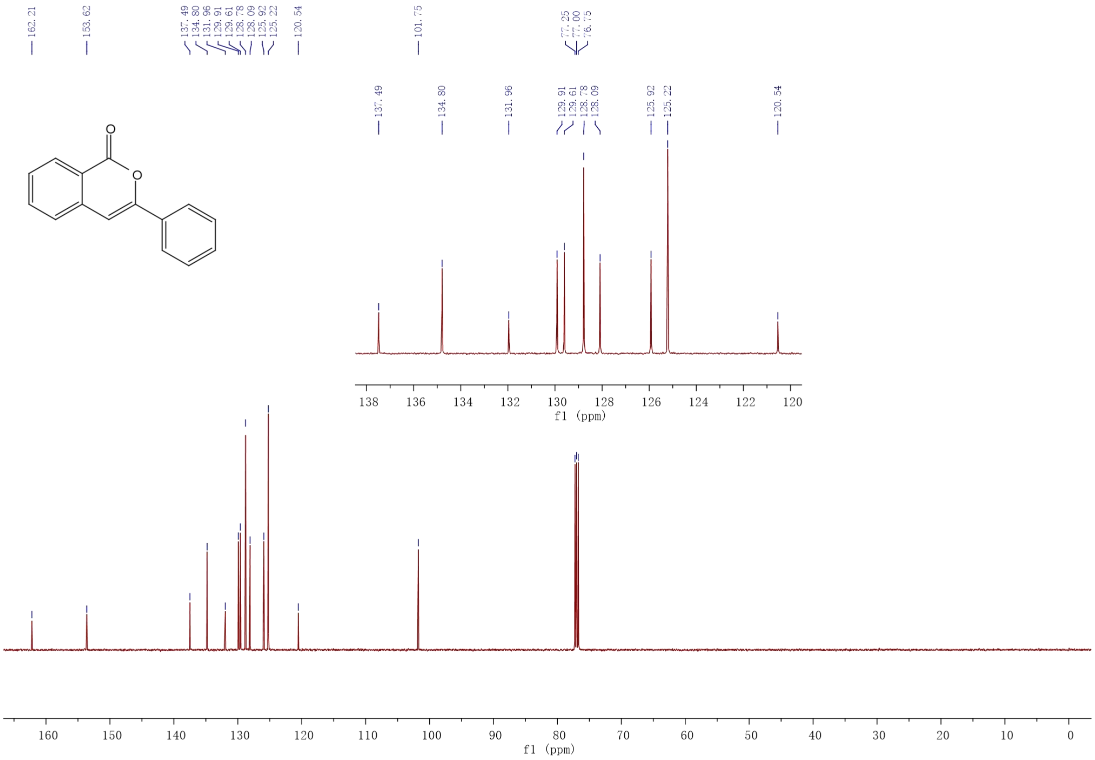

^{13}C NMR (500 Hz, CDCl$_3$): **2a**

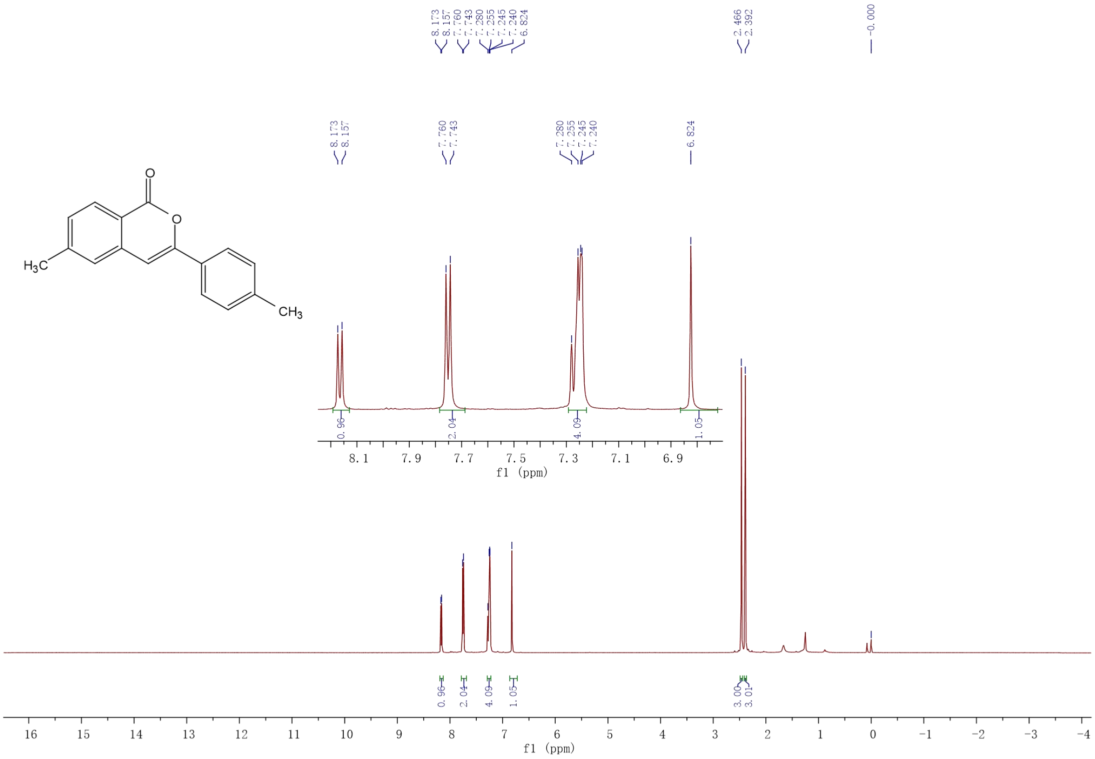

^{1}H NMR (500 Hz, $CDCl_3$): **2b**

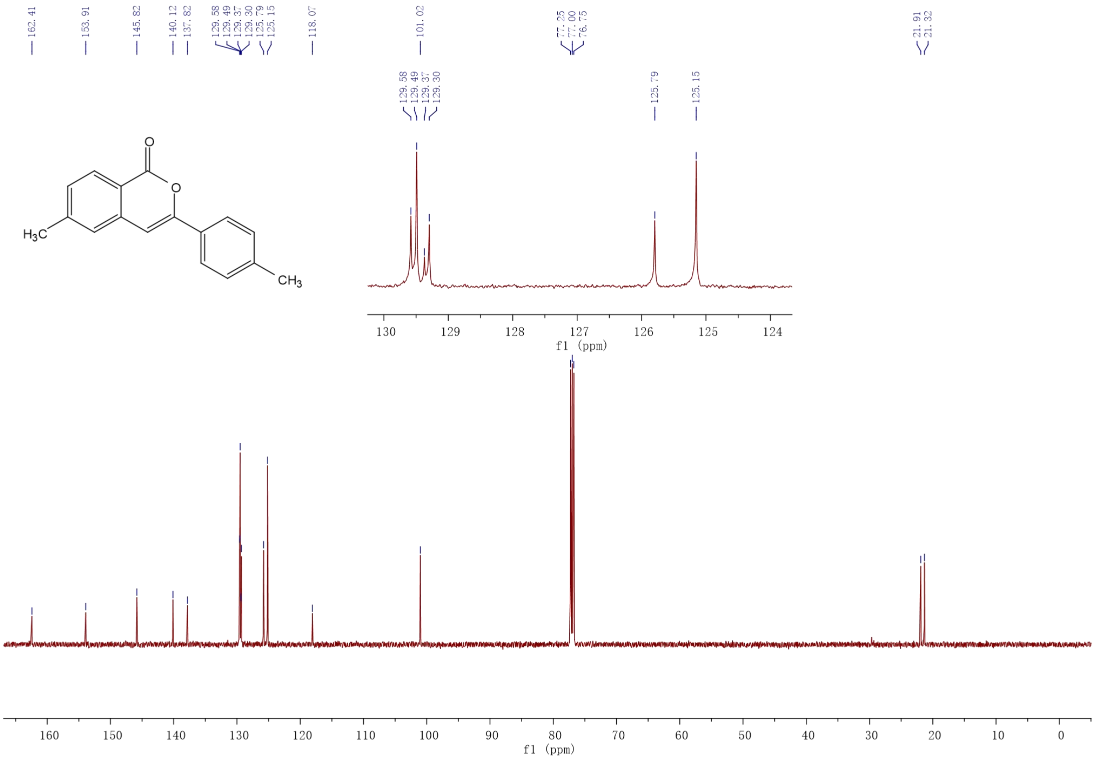

^{13}C NMR (500 Hz, $CDCl_3$): **2b**

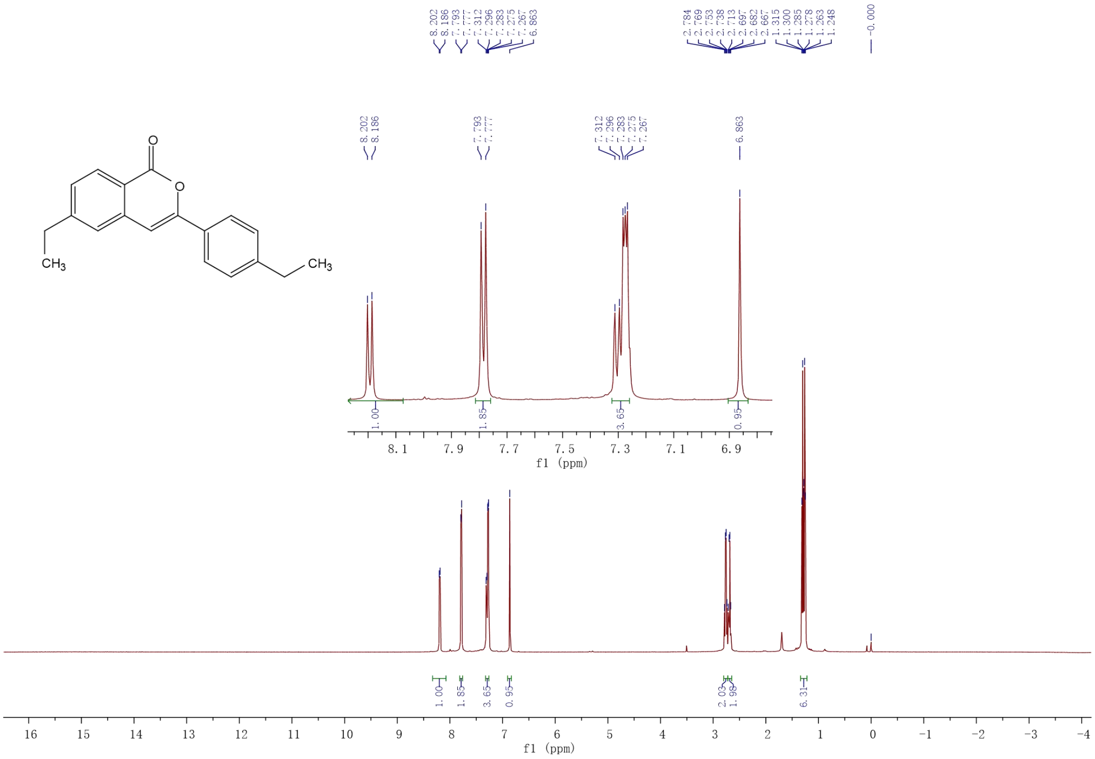

^{1}H NMR (500 Hz, $CDCl_3$): **2c**

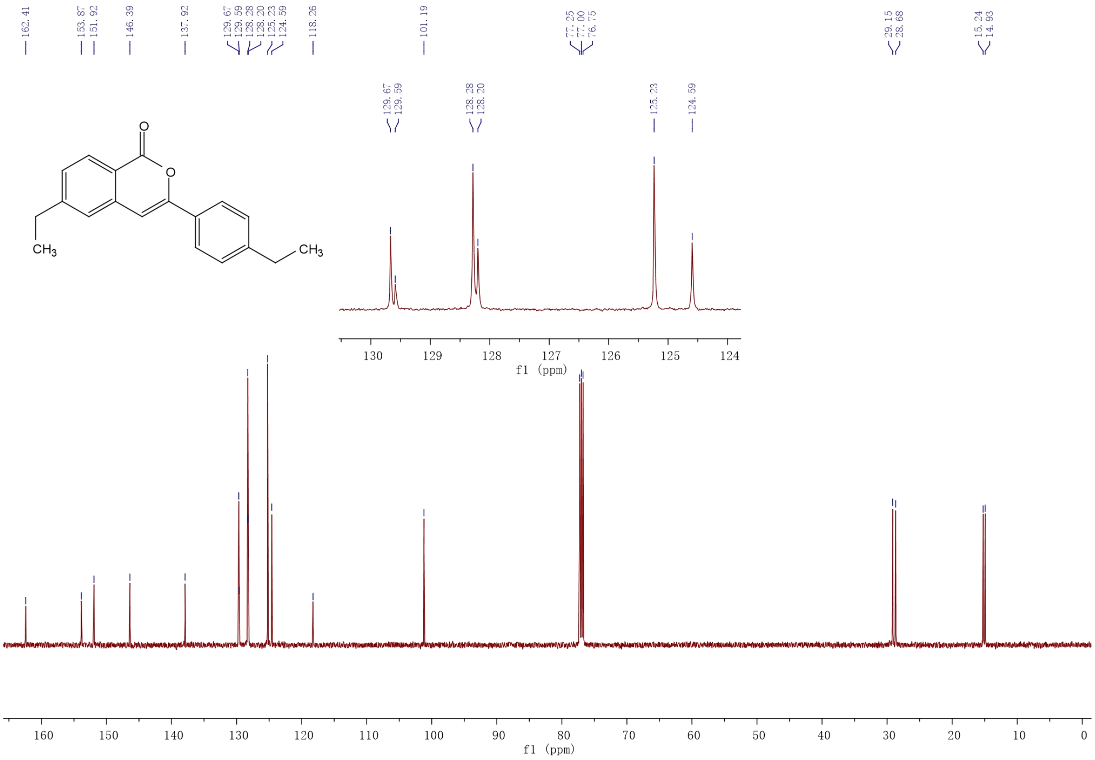

^{13}C NMR (500 Hz, $CDCl_3$): **2c**

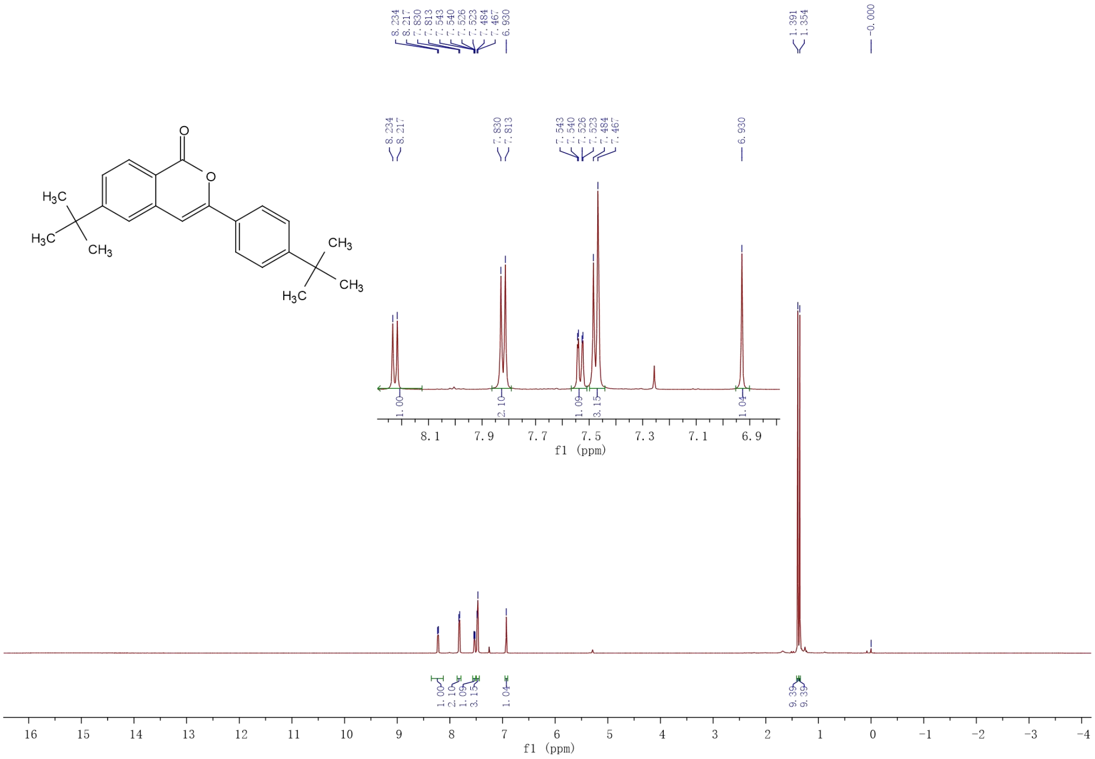

^{1}H NMR (500 Hz, $CDCl_3$): **2d**

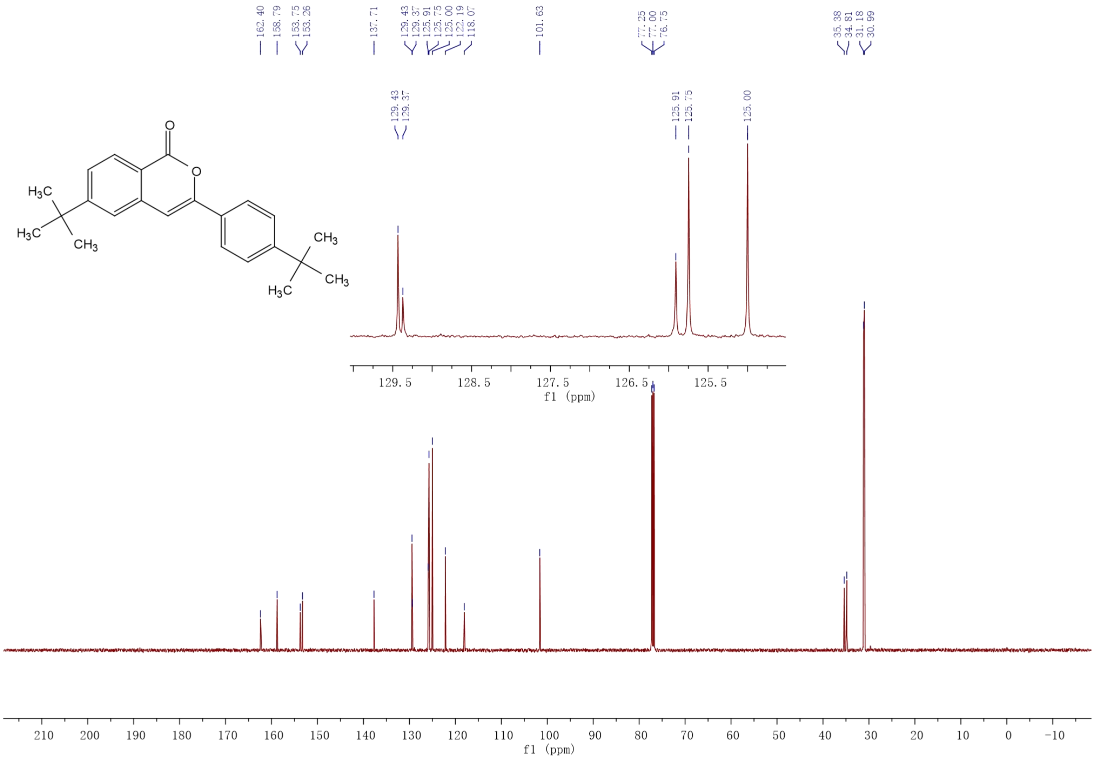

^{13}C NMR (500 Hz, $CDCl_3$): **2d**

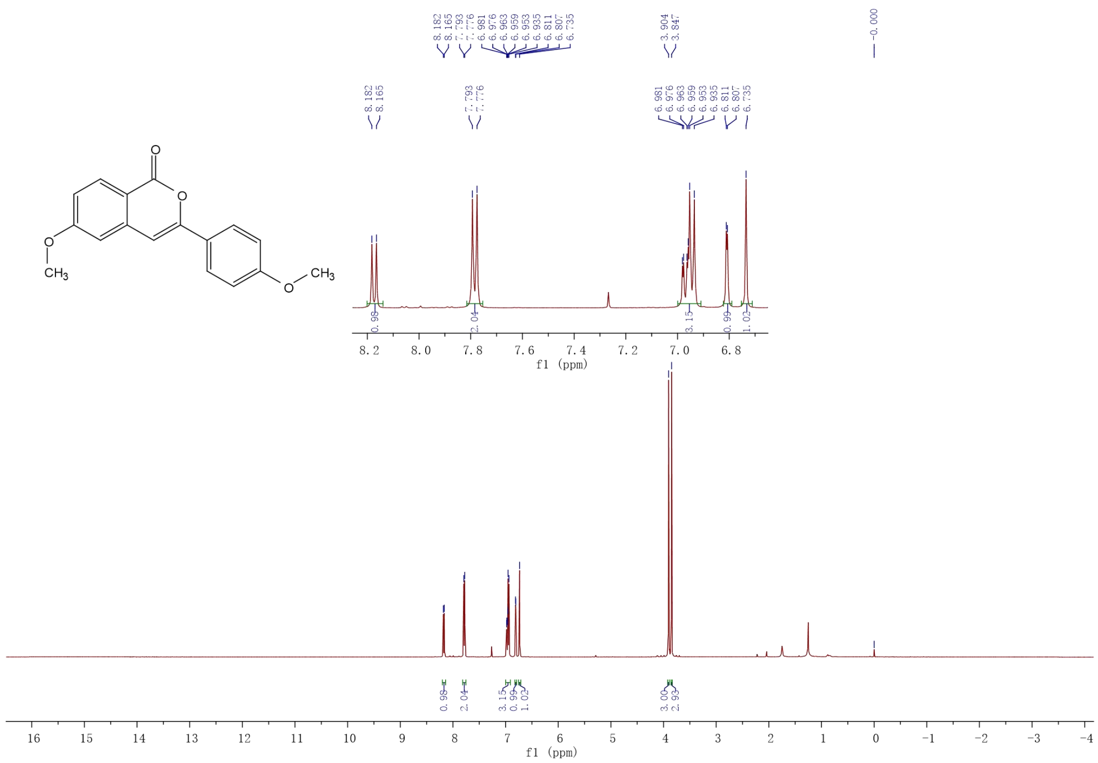

^{1}H NMR (500 Hz, $CDCl_3$): **2e**

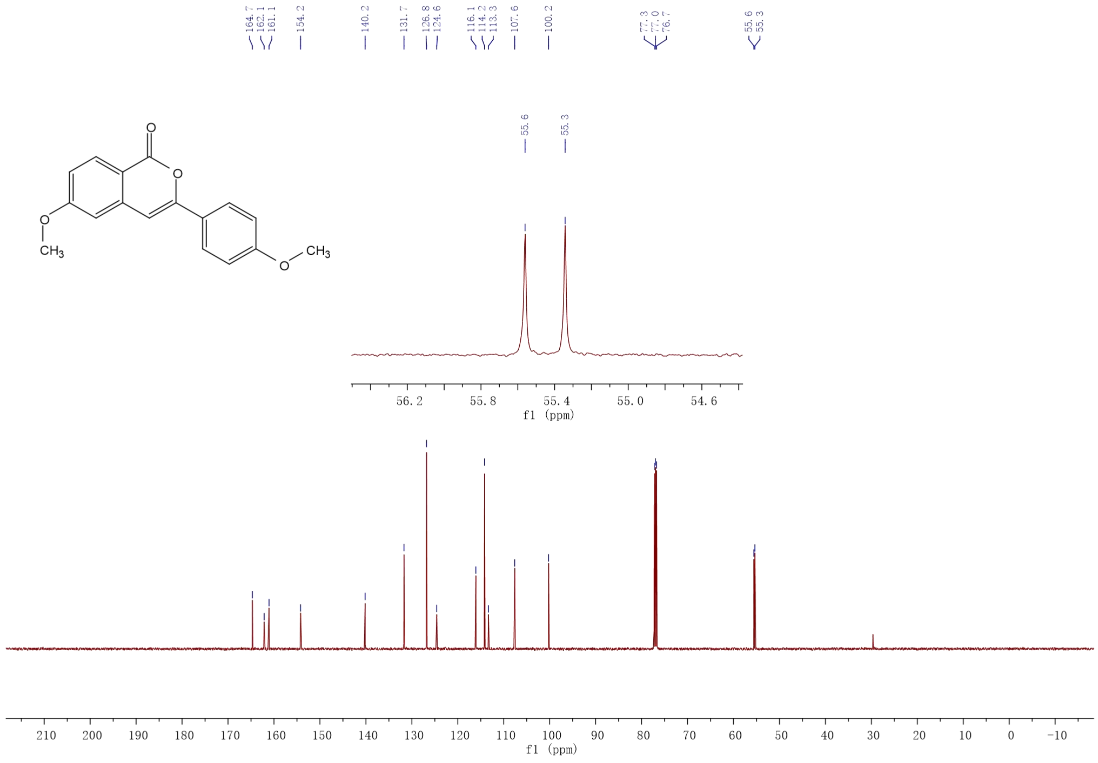

^{13}C NMR (500 Hz, $CDCl_3$): **2e**

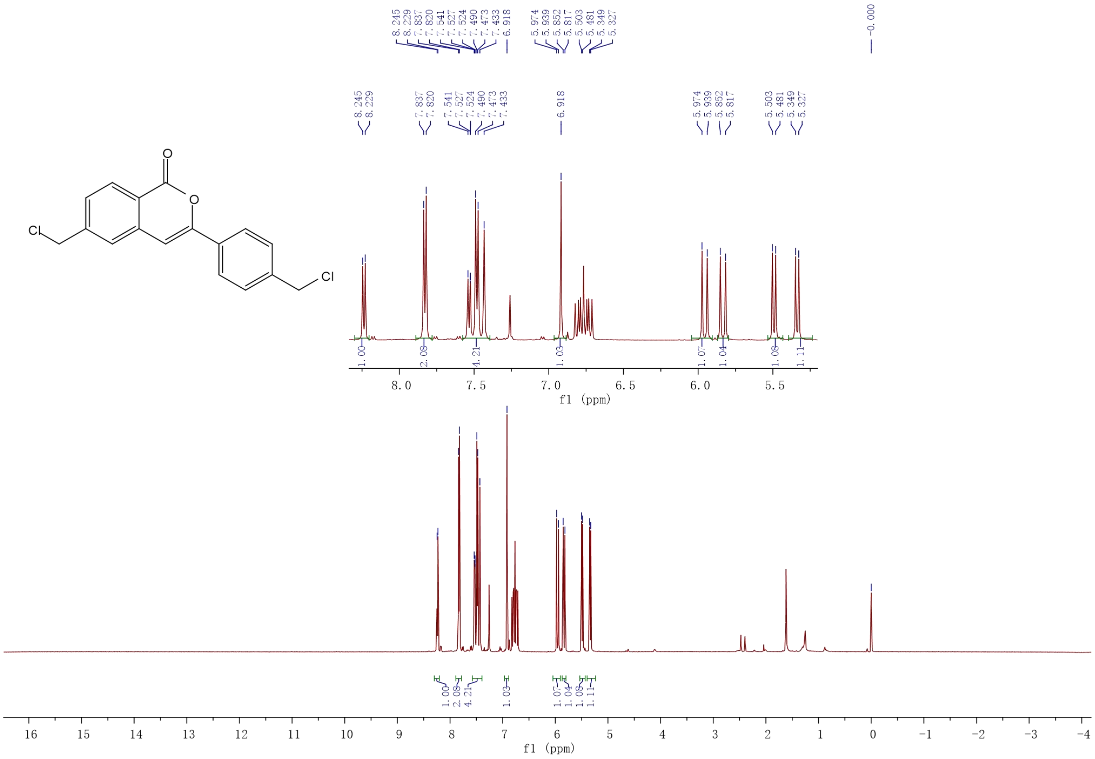

^{1}H NMR (500 Hz, $CDCl_3$): **2f**

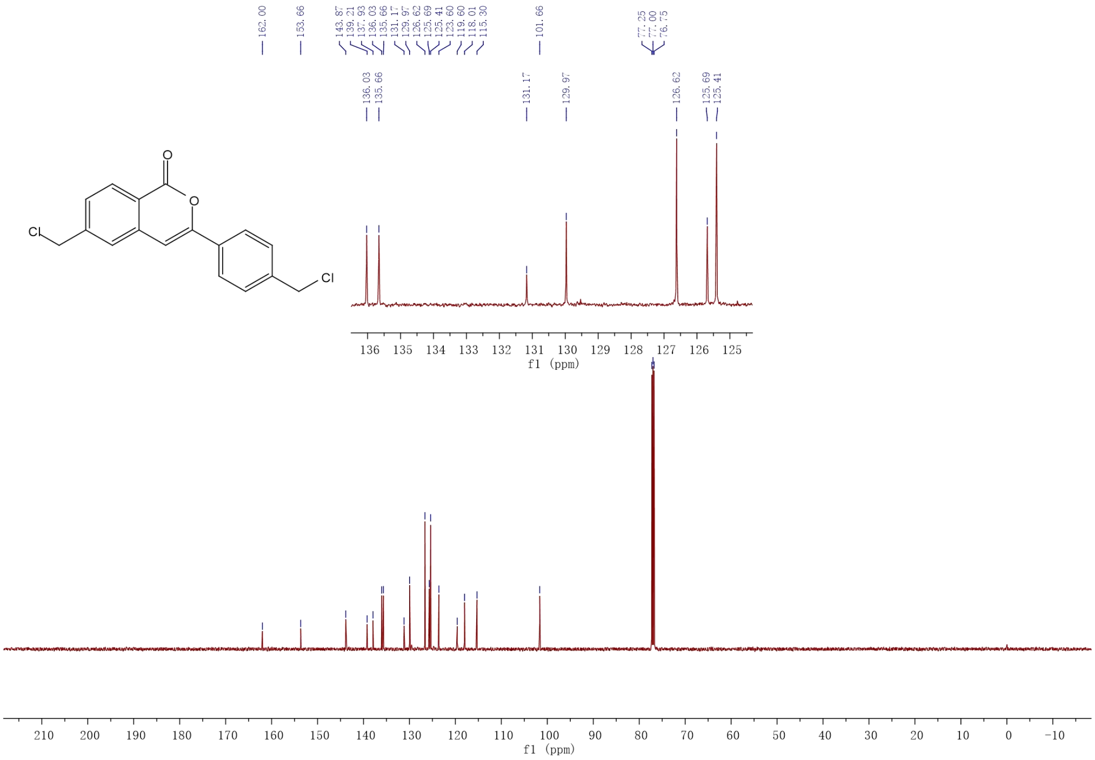

^{13}C NMR (500 Hz, $CDCl_3$): **2f**

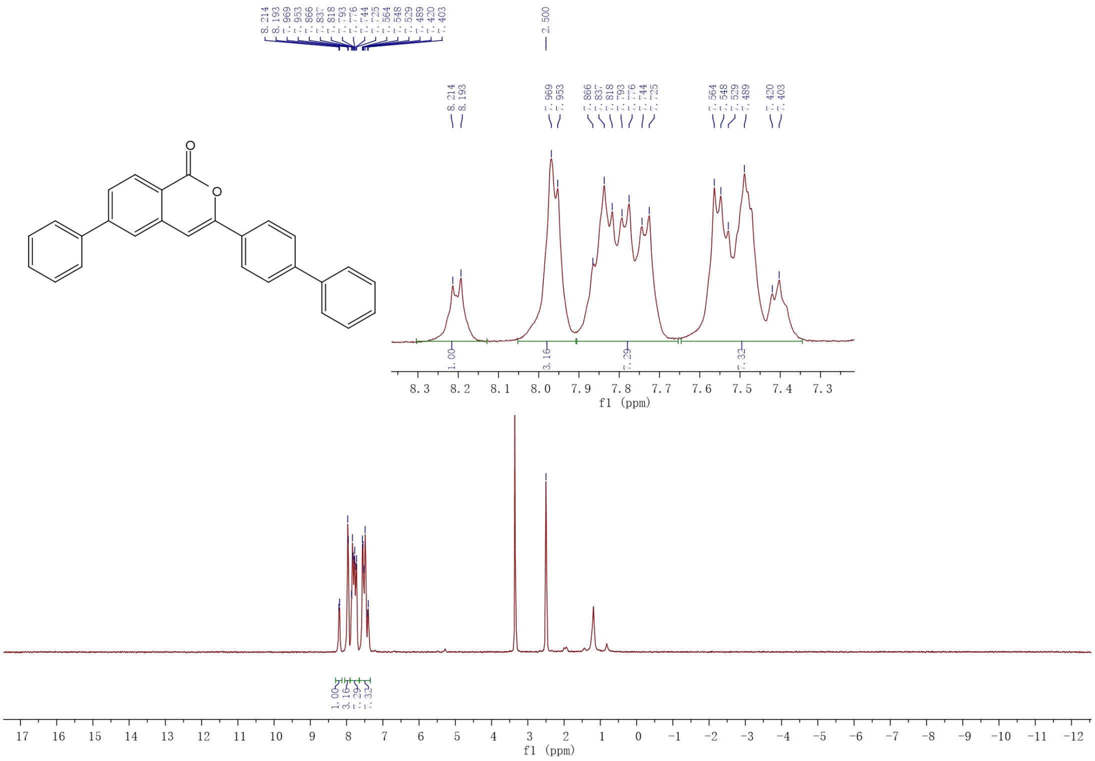

^{1}H NMR (400 Hz, DMSO): **2g**

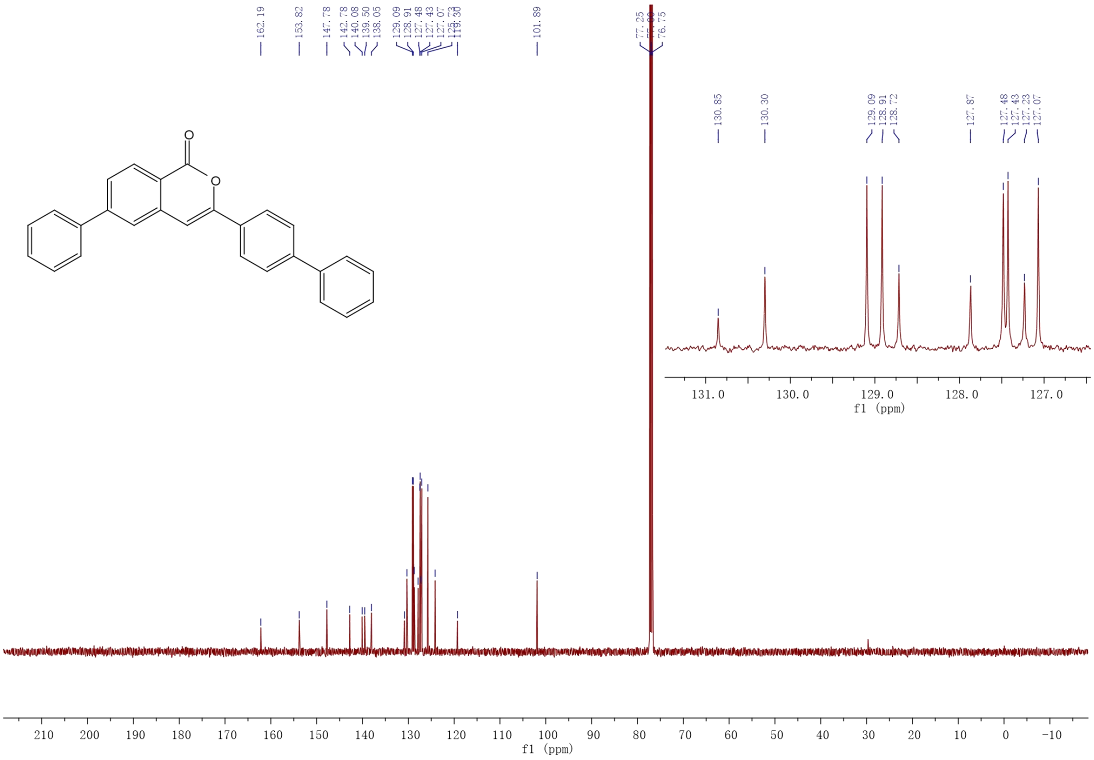

^{13}C NMR (500 Hz, $CDCl_3$): **2g**

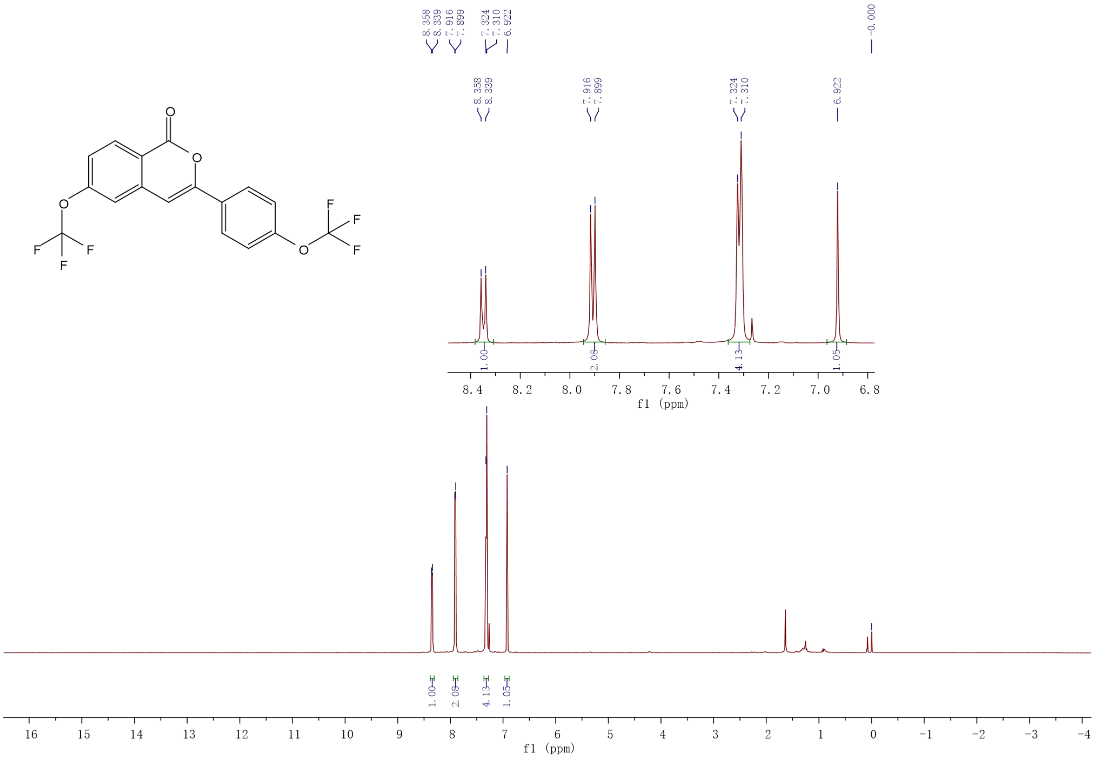

^{1}H NMR (500 Hz, $CDCl_3$): **2h**

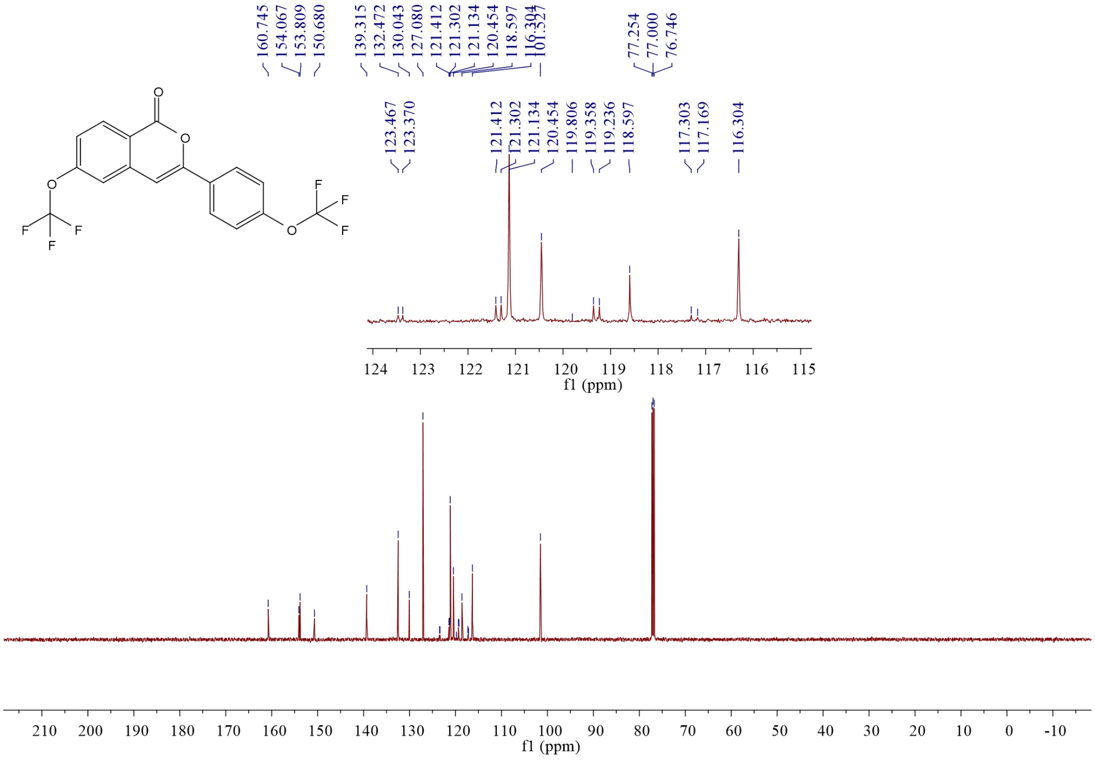

^{13}C NMR (500 Hz, $CDCl_3$): **2h**

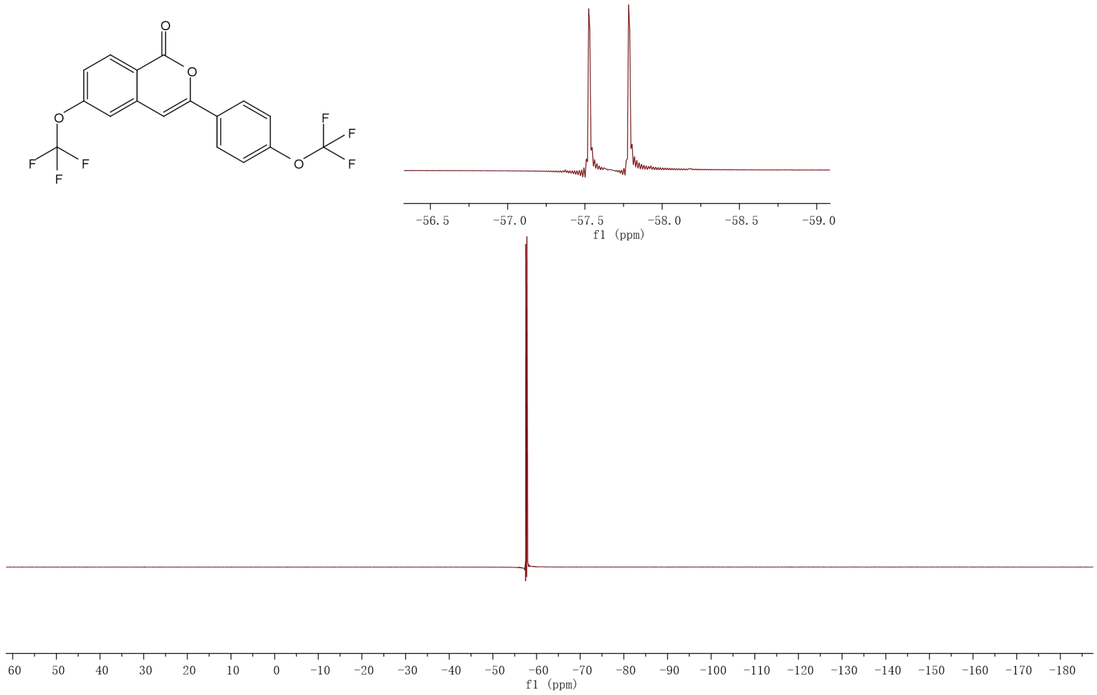

^{19}F NMR (500 Hz, $CDCl_3$): **2h**

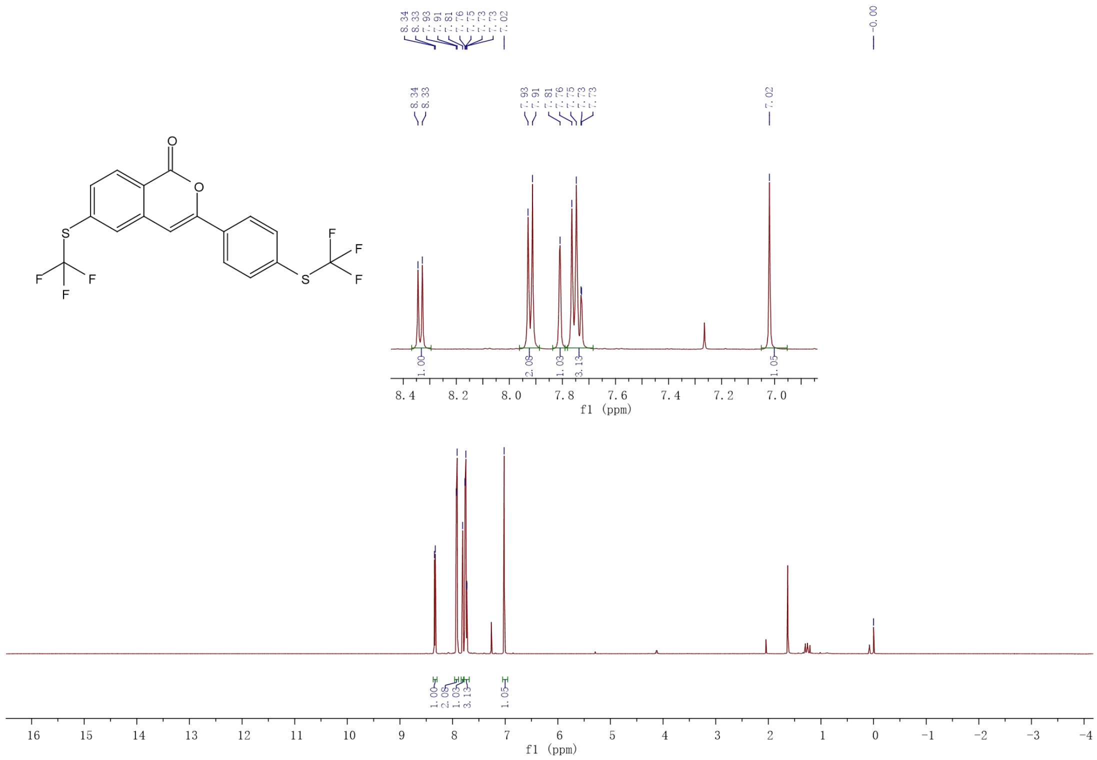

^{1}H NMR (500 Hz, $CDCl_3$): **2i**

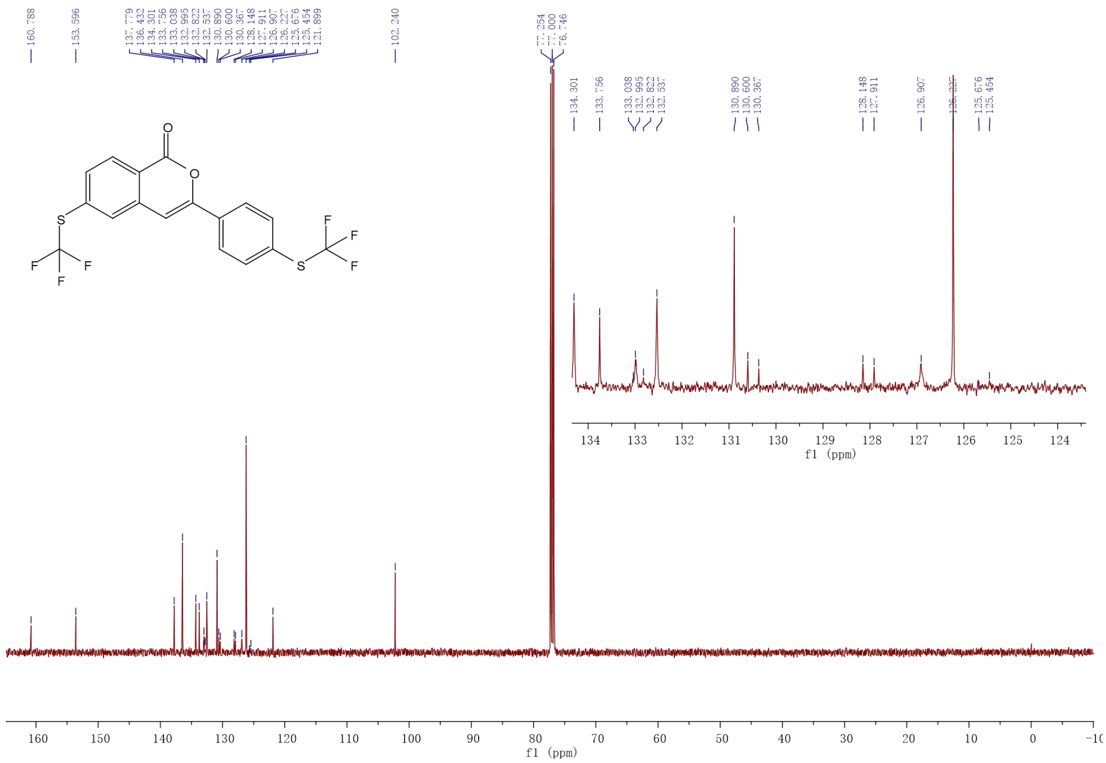

^{13}C NMR (500 Hz, $CDCl_3$): **2i**

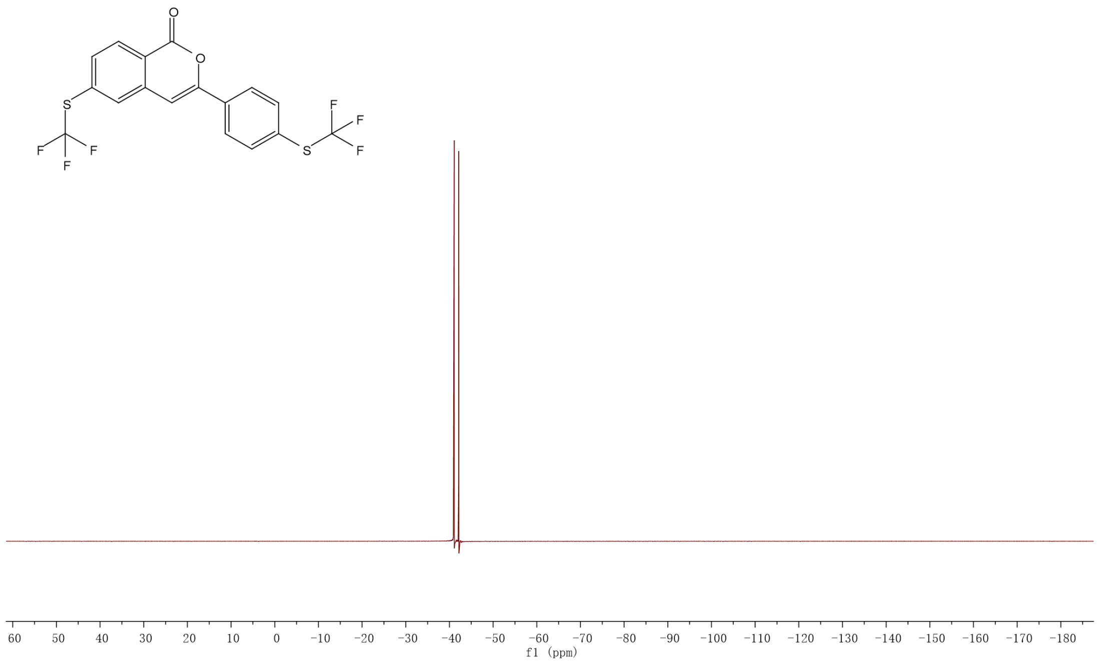

^{19}F NMR (500 Hz, $CDCl_3$): **2i**

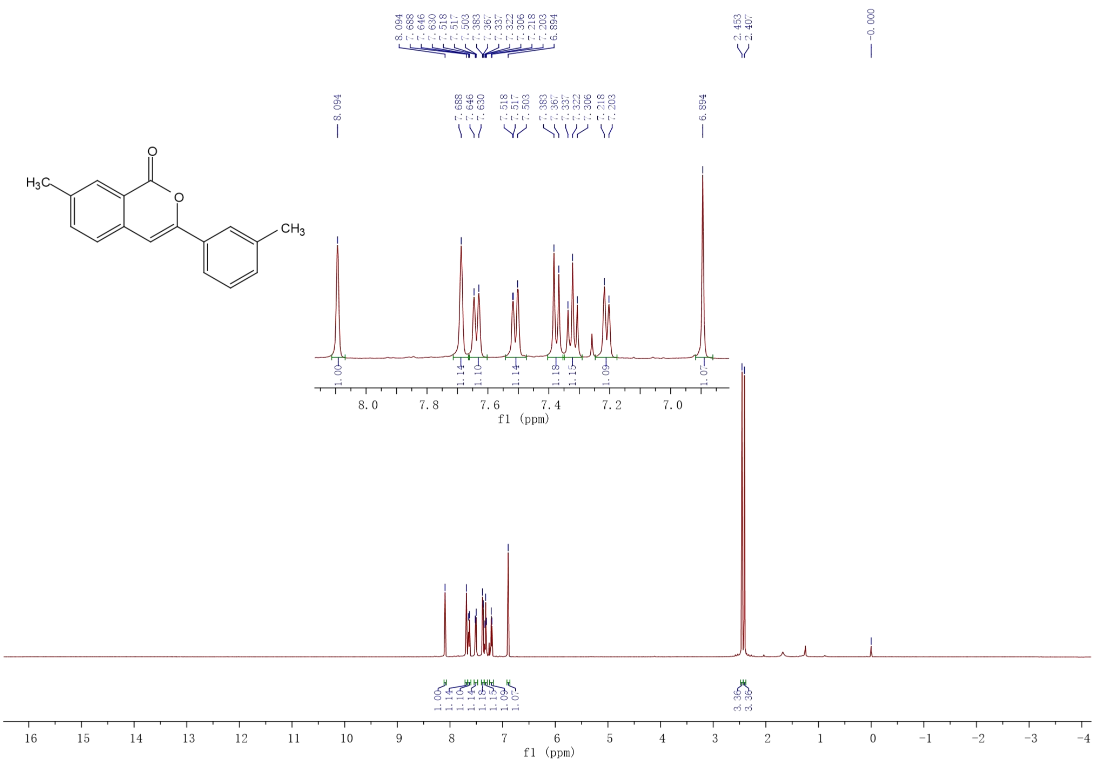

^{1}H NMR (500 Hz, CDCl$_3$): **2j**

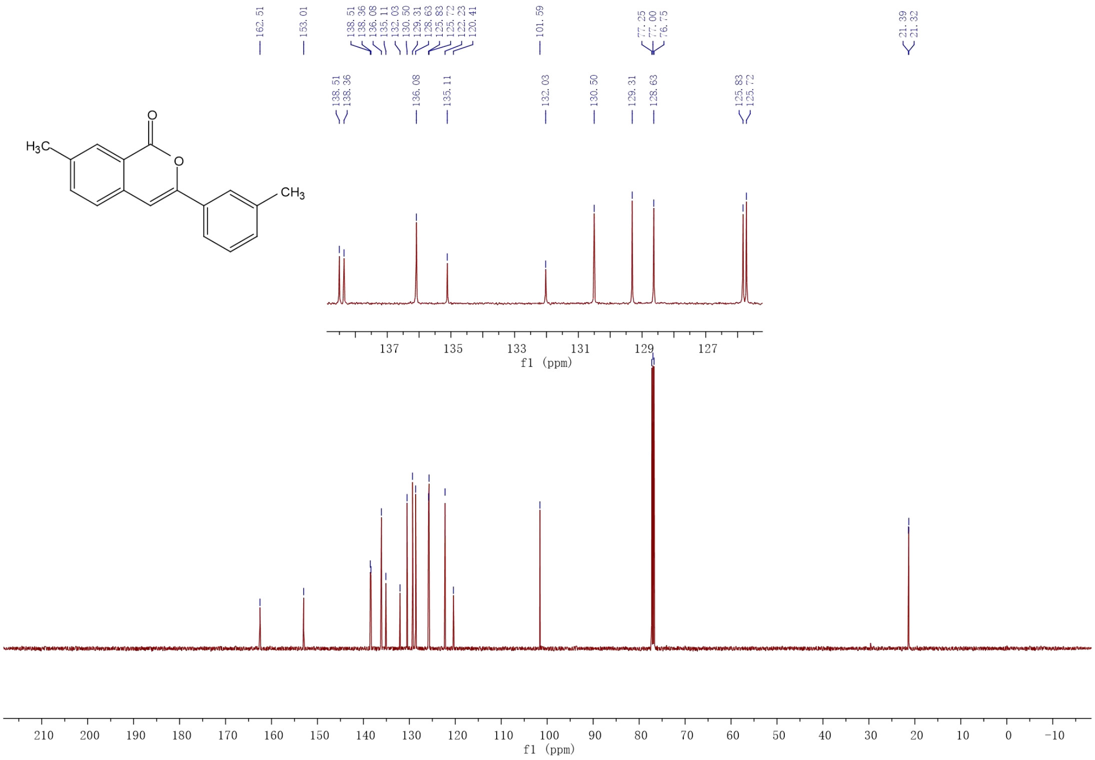

^{13}C NMR (500 Hz, CDCl$_3$): **2j**

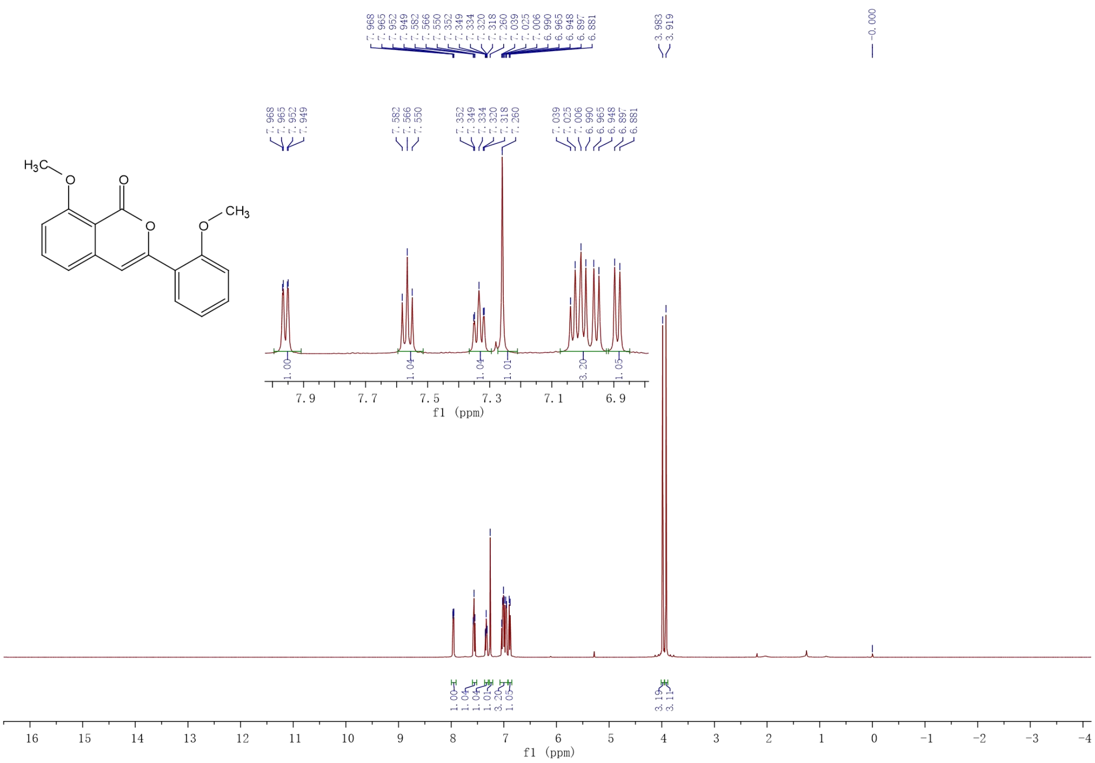

^{1}H NMR (500 Hz, $CDCl_3$): **2k**

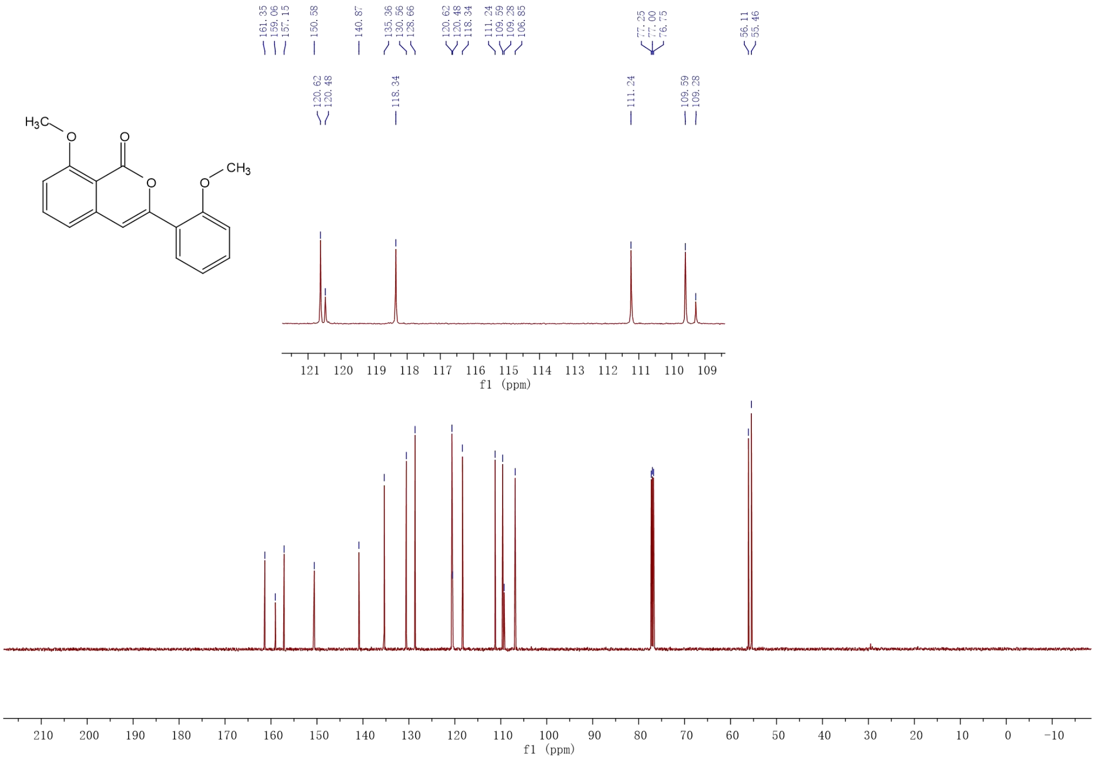

^{13}C NMR (500 Hz, $CDCl_3$): **2k**

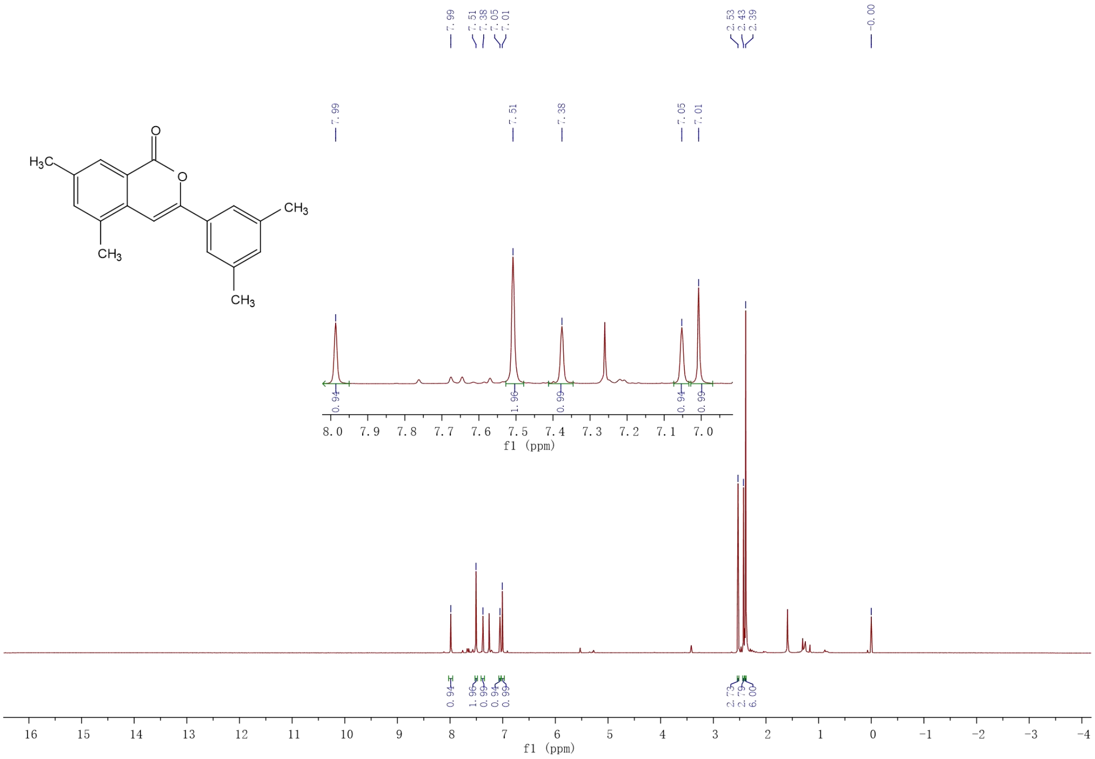

^{1}H NMR (500 Hz, $CDCl_3$): **2l**

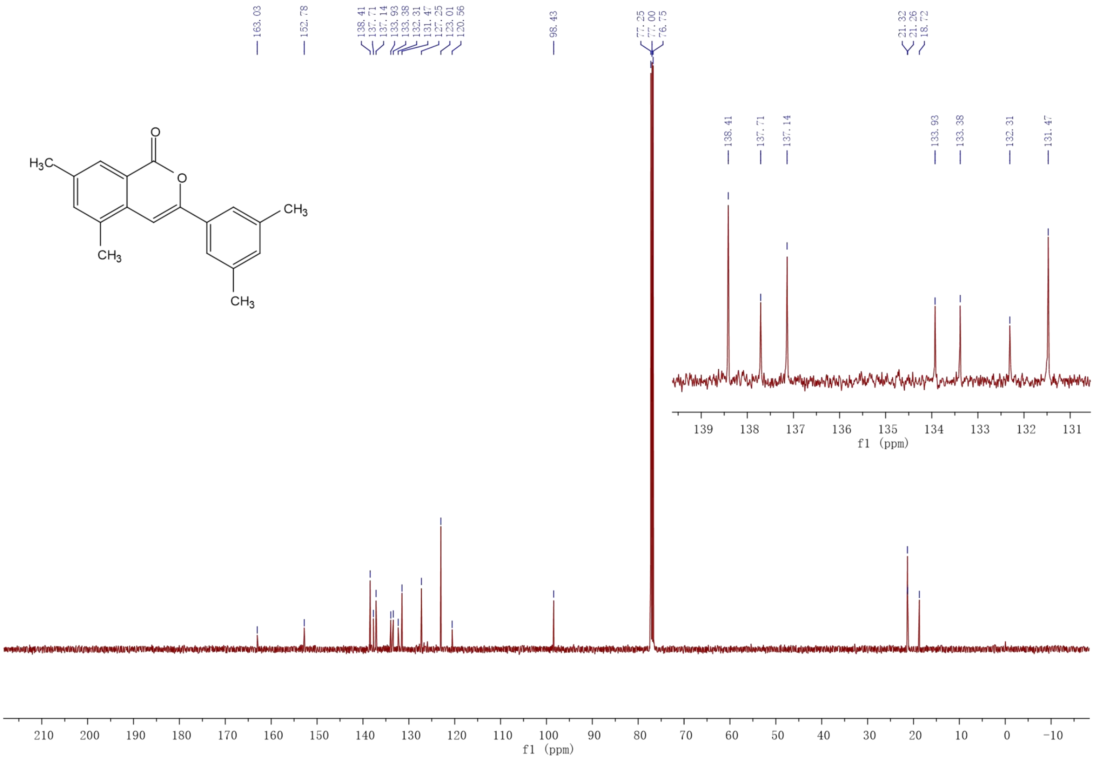

^{13}C NMR (500 Hz, $CDCl_3$): **2l**

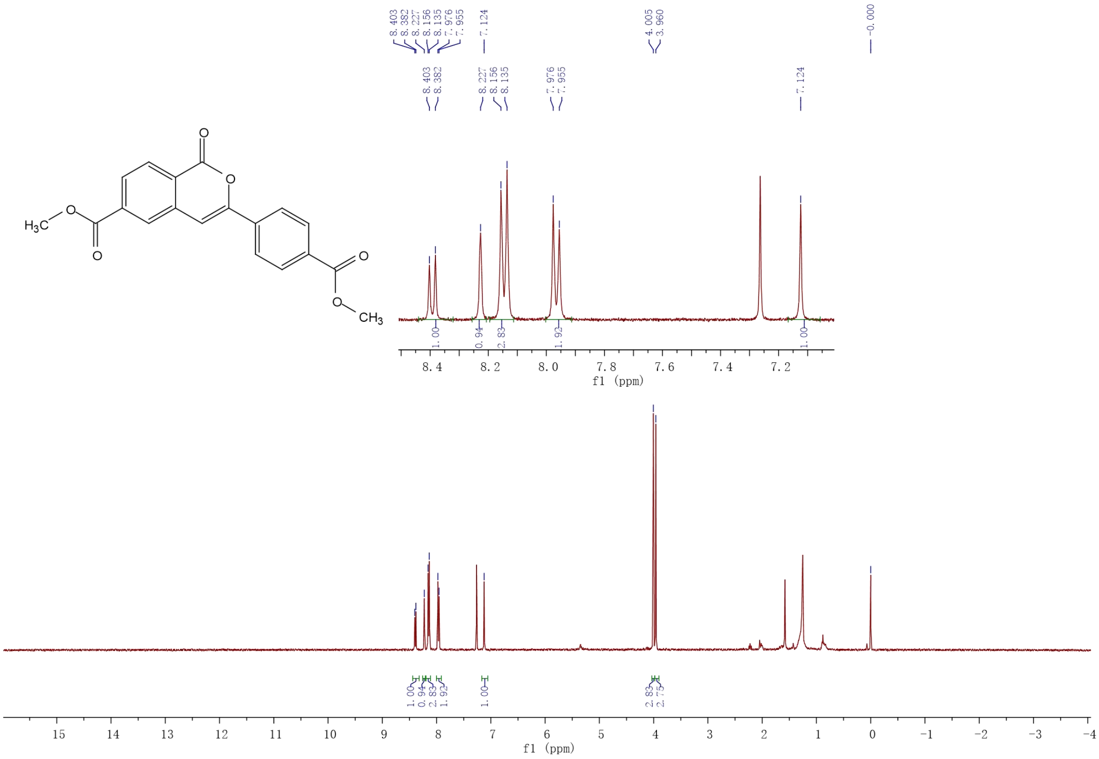

^{1}H NMR (400 Hz, $CDCl_3$): **2m**

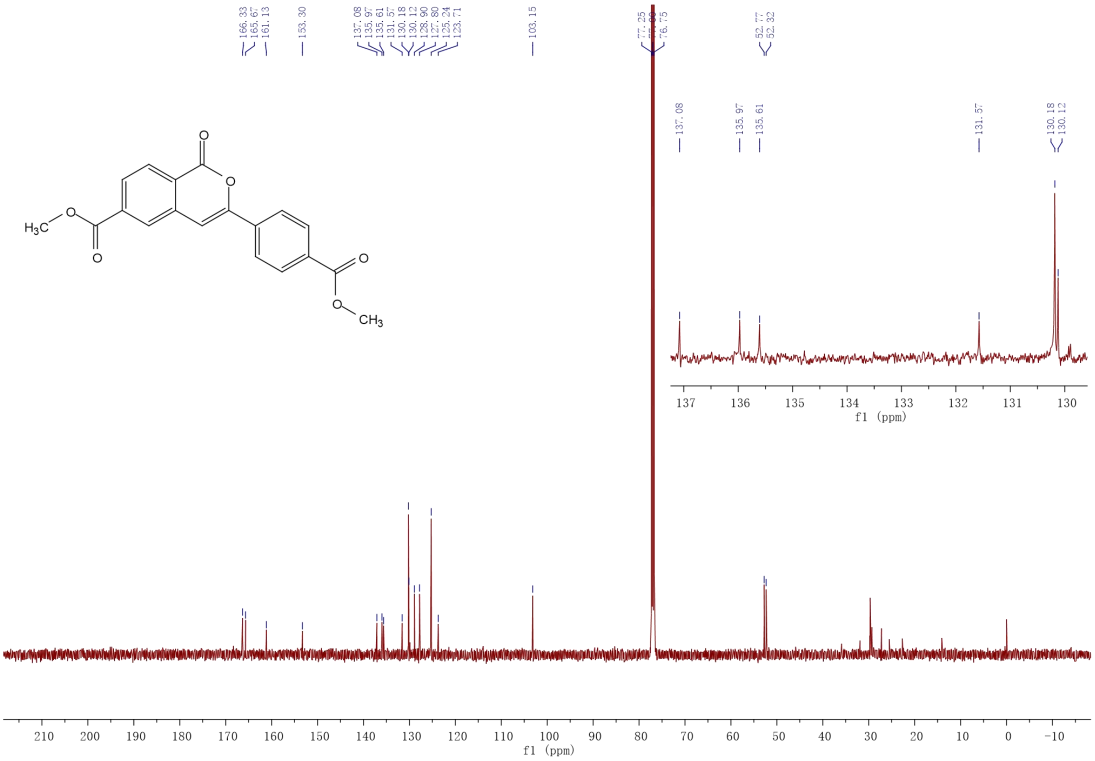

^{13}C NMR (500 Hz, $CDCl_3$): **2m**

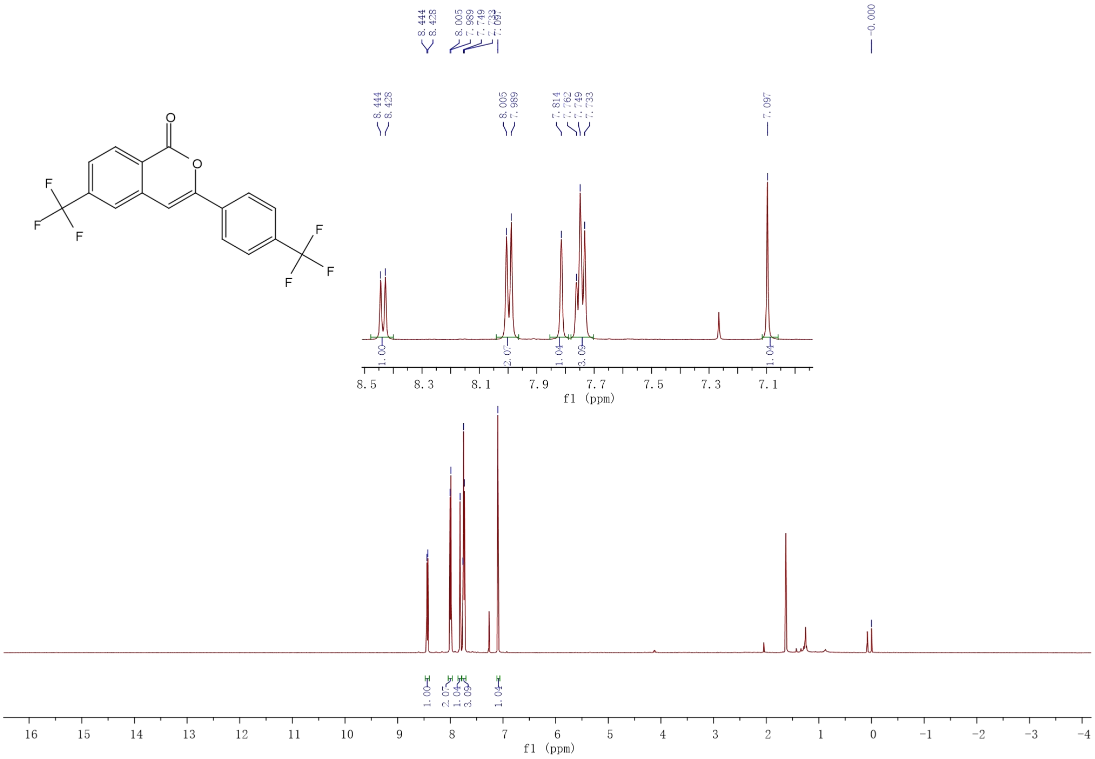

^{1}H NMR (500 Hz, $CDCl_3$): **2n**

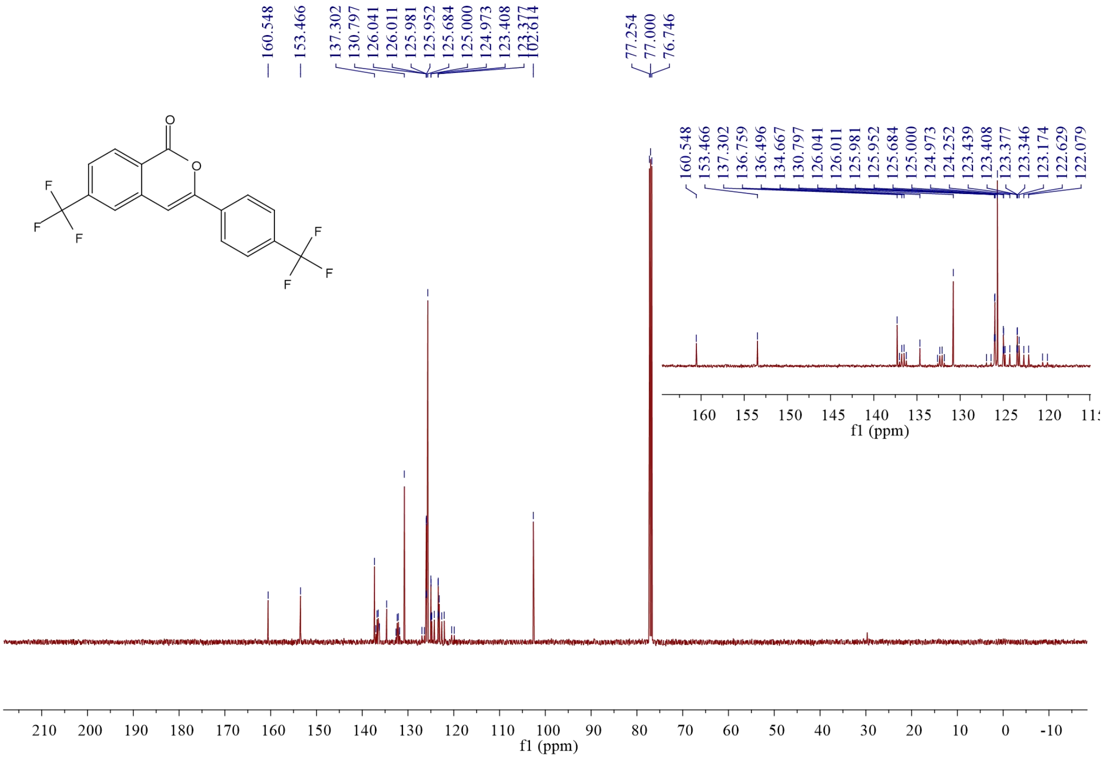

^{13}C NMR (500 Hz, $CDCl_3$): **2n**

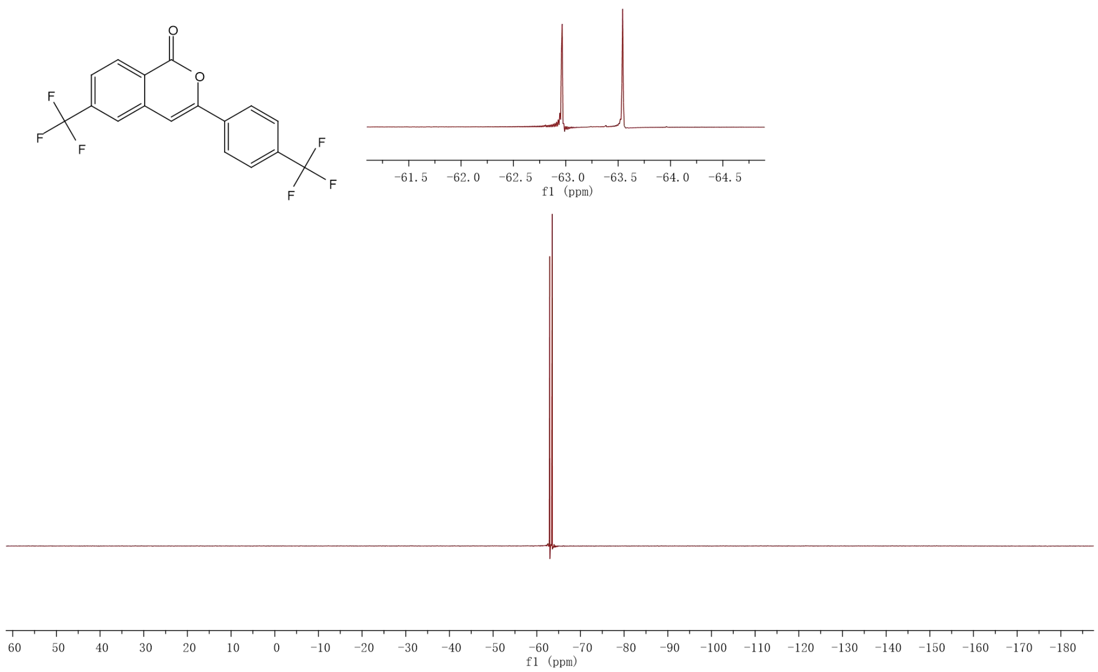

^{19}F NMR (500 Hz, $CDCl_3$): **2n**

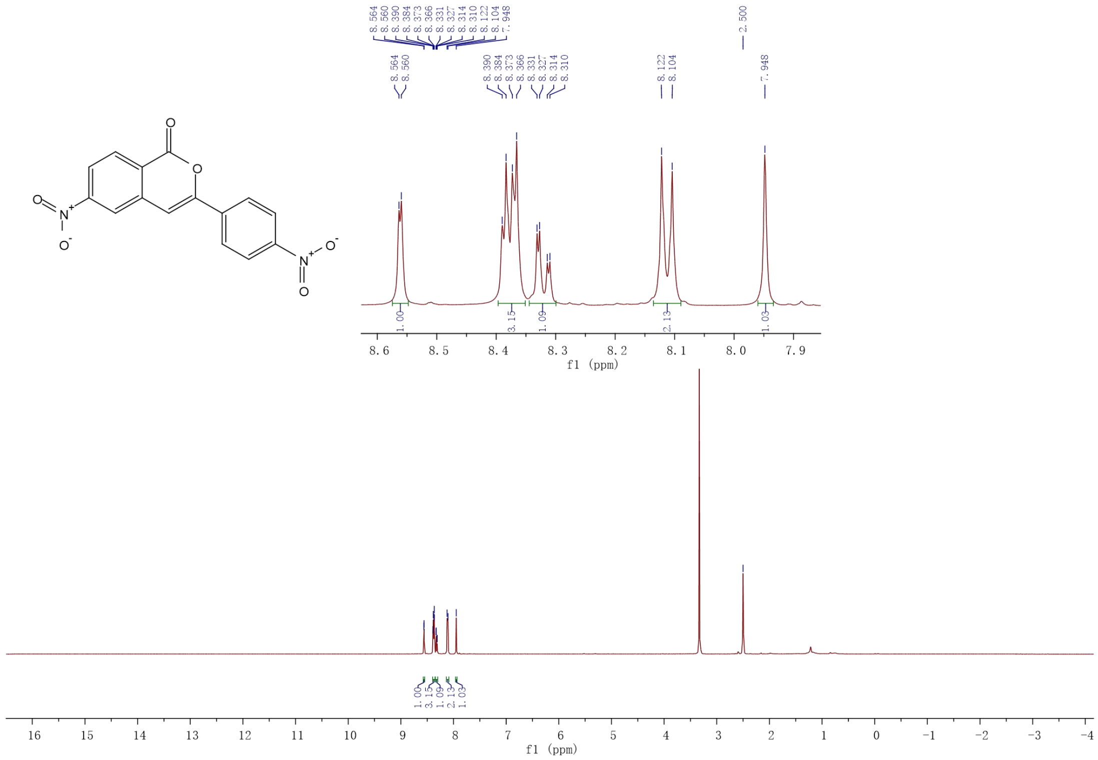

^{1}H NMR (500 Hz, DMSO): **2o**

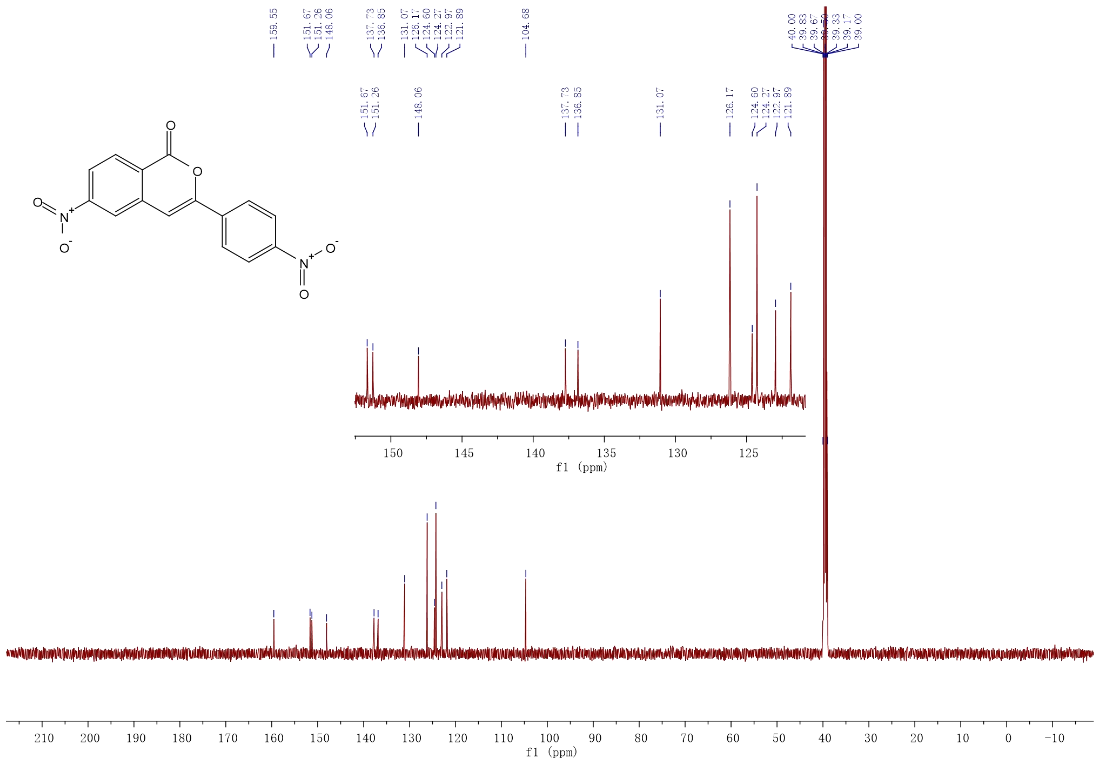

^{13}C NMR (500 Hz, DMSO): **2o**

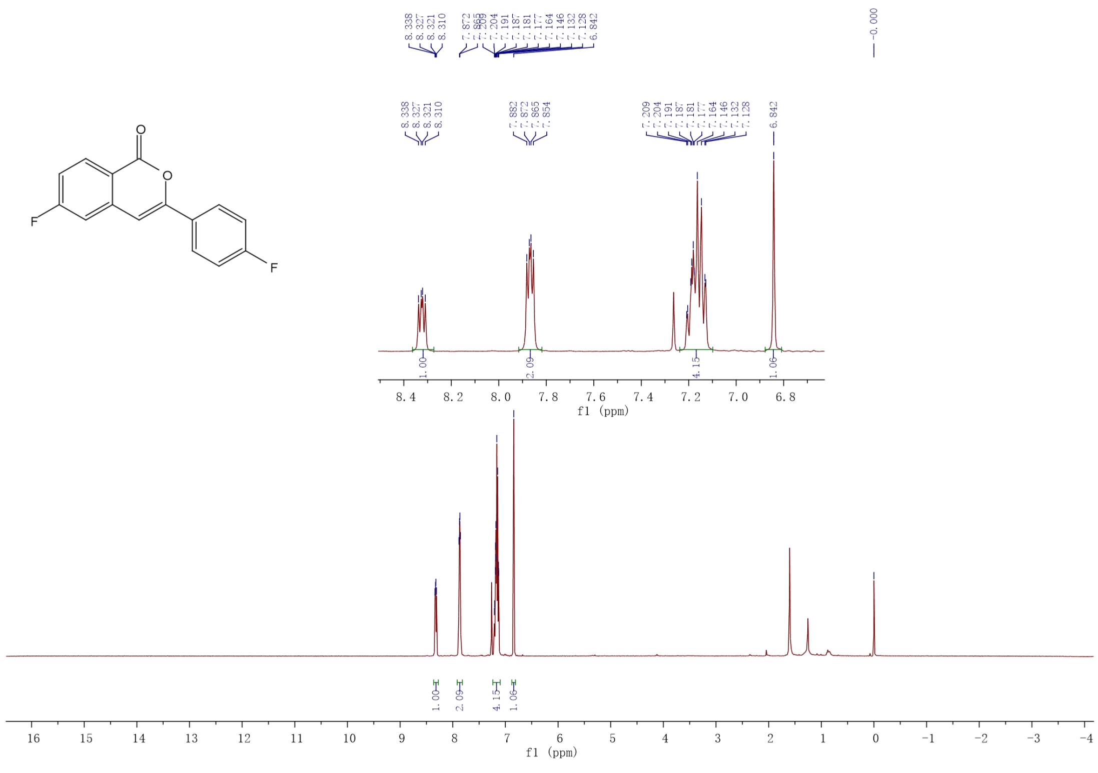

^{1}H NMR (500 Hz, $CDCl_3$): **2p**

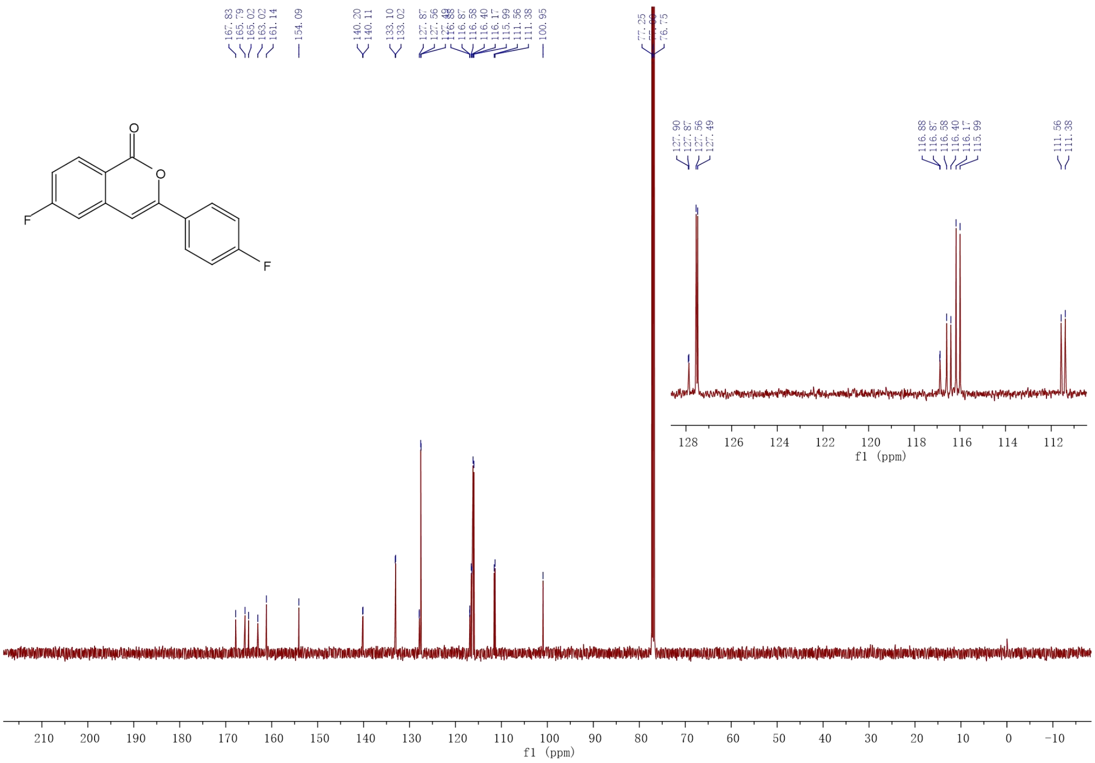

^{13}C NMR (500 Hz, $CDCl_3$): **2p**

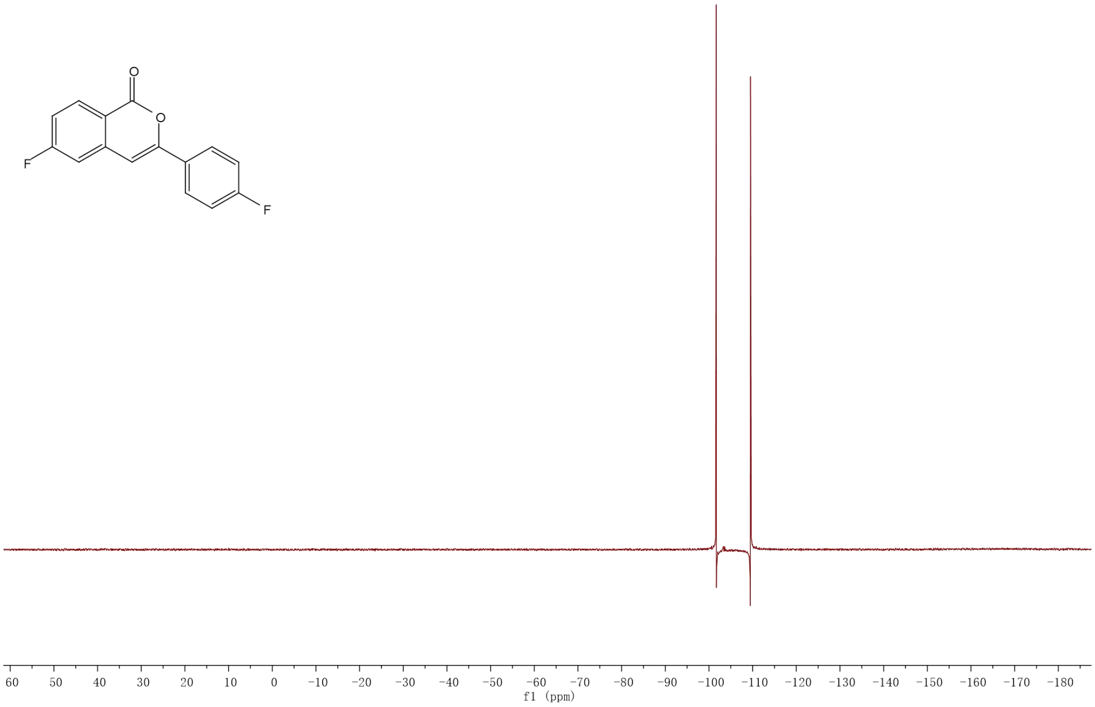

^{19}F NMR (500 Hz, $CDCl_3$): **2p**

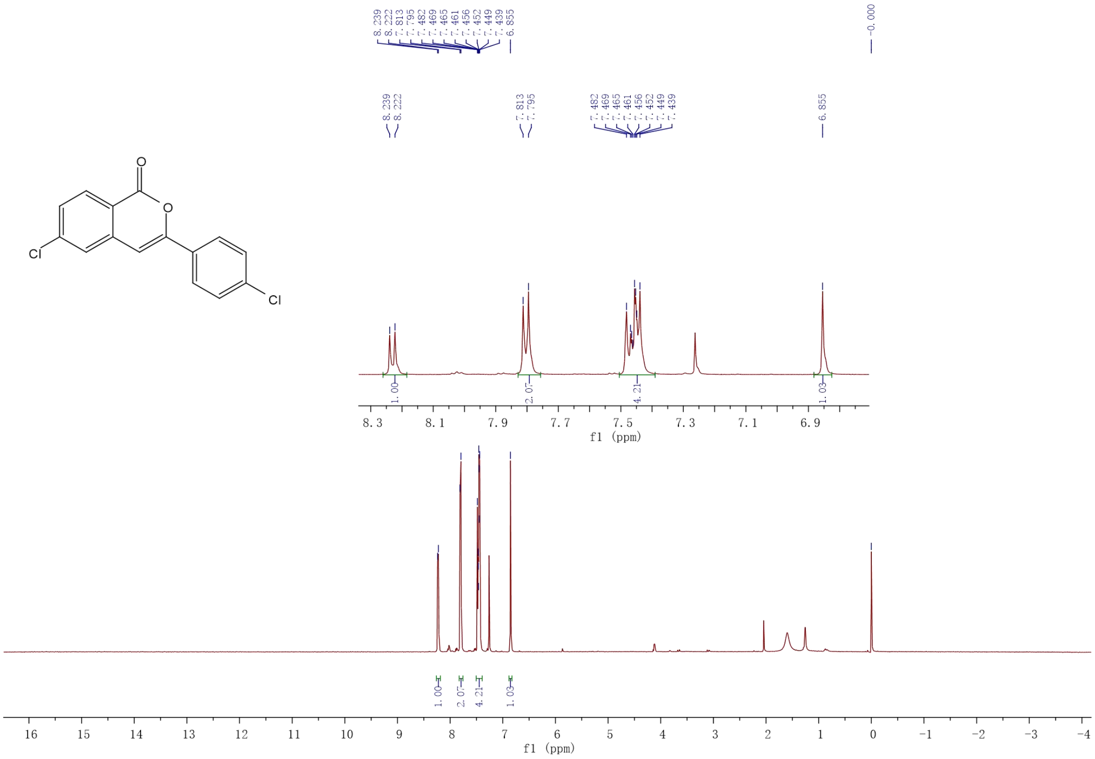

^{1}H NMR (500 Hz, $CDCl_3$): **2q**

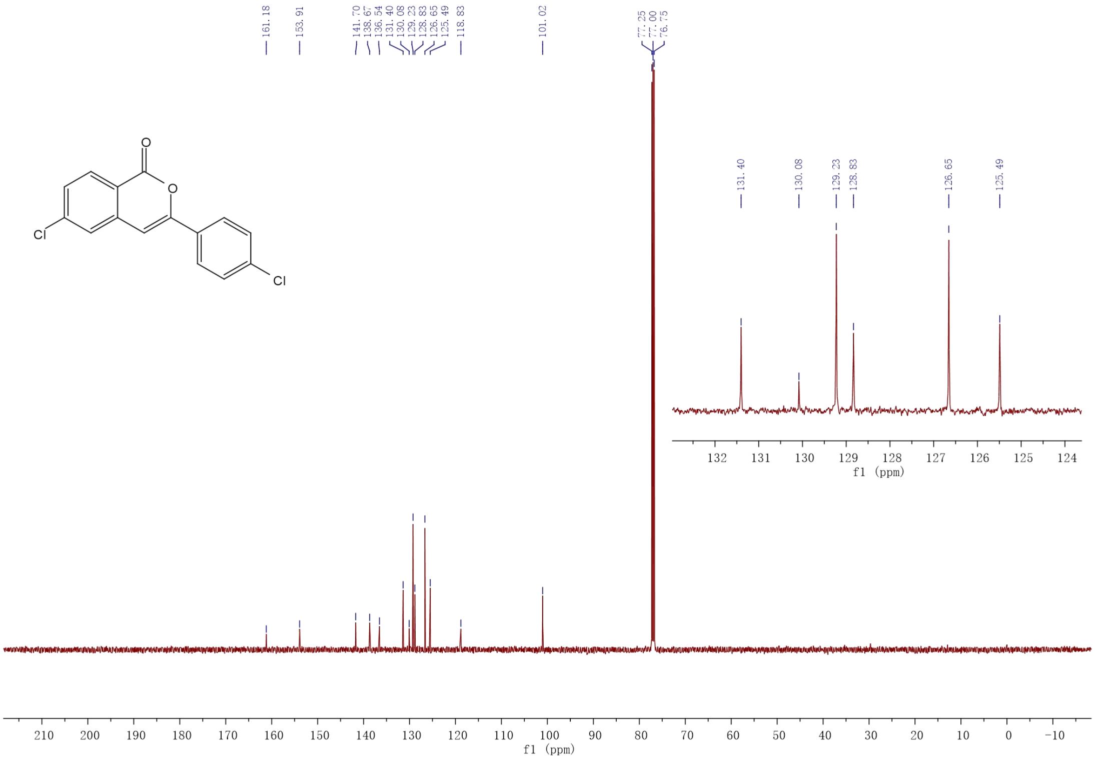

^{13}C NMR (500 Hz, $CDCl_3$): **2q**

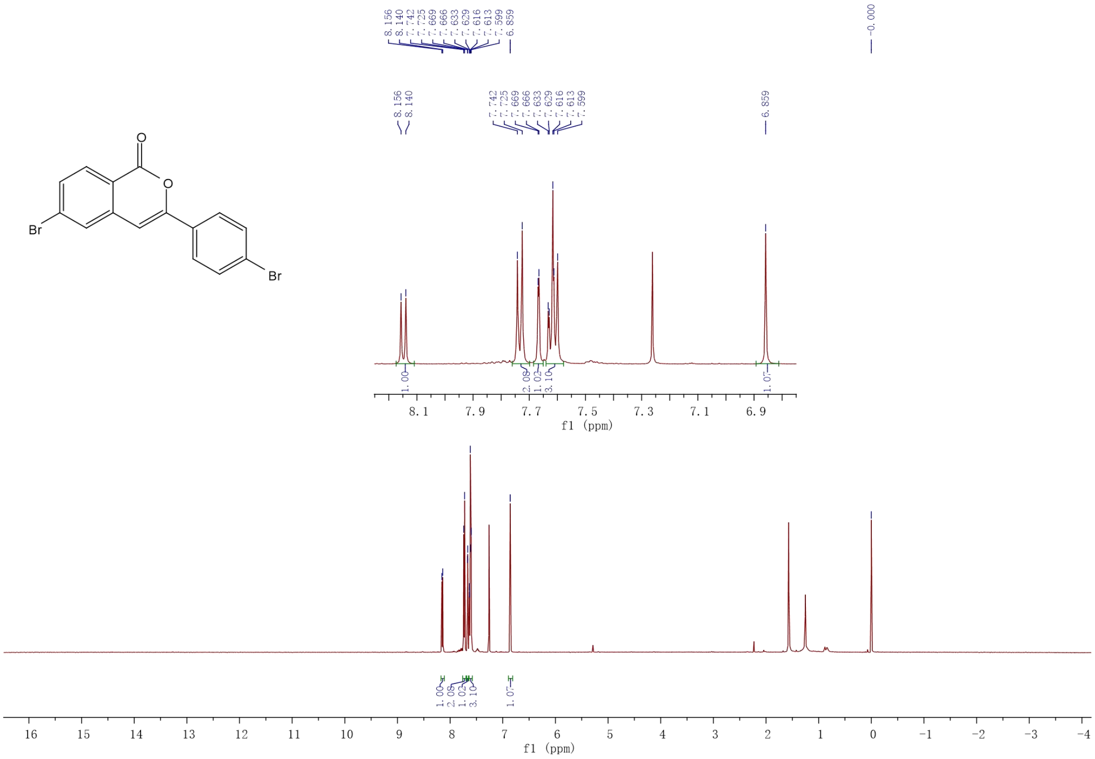

^{1}H NMR (500 Hz, $CDCl_3$): **2r**

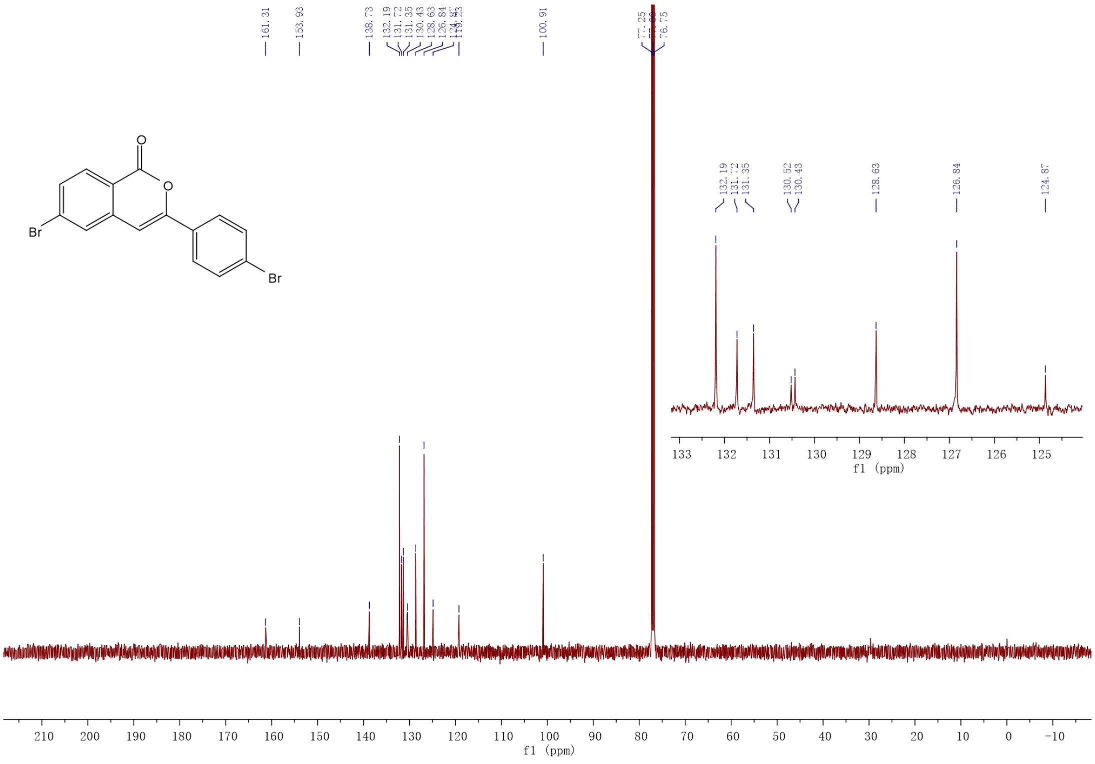

^{13}C NMR (500 Hz, $CDCl_3$): **2r**

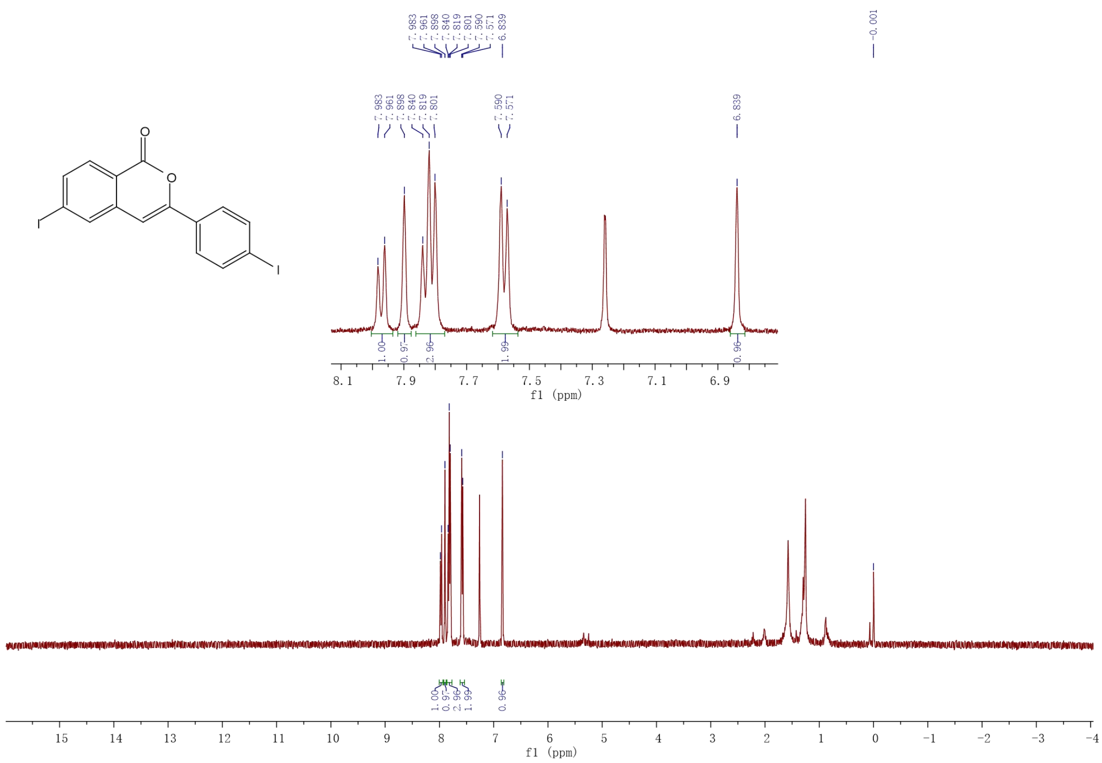

^{1}H NMR (400 Hz, $CDCl_3$): **2s**

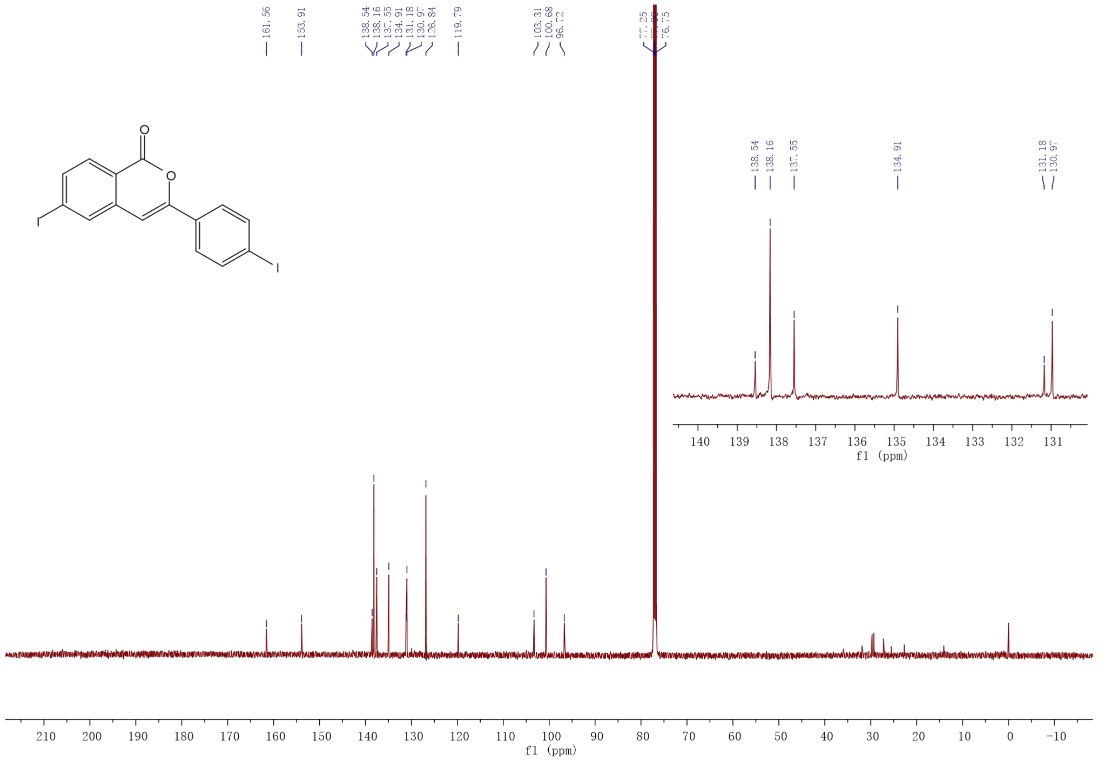

^{13}C NMR (500 Hz, $CDCl_3$): **2s**

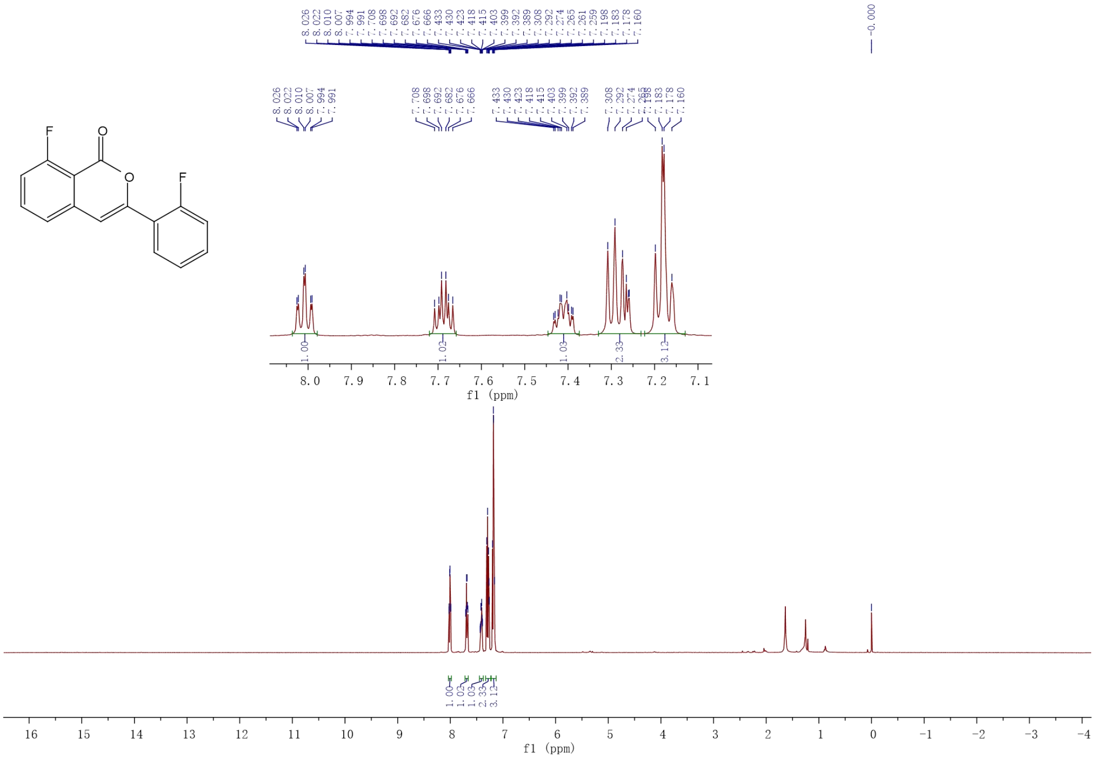

^{1}H NMR (500 Hz, CDCl₃): **2t**

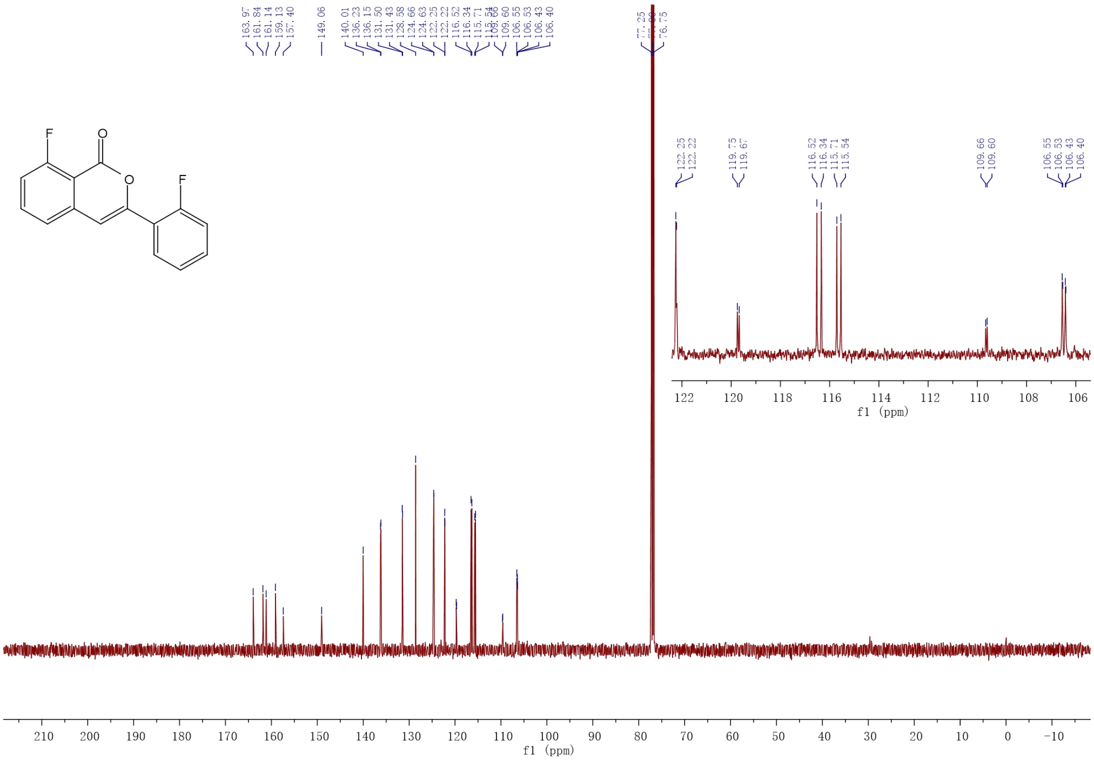

^{13}C NMR (500 Hz, CDCl₃): **2t**

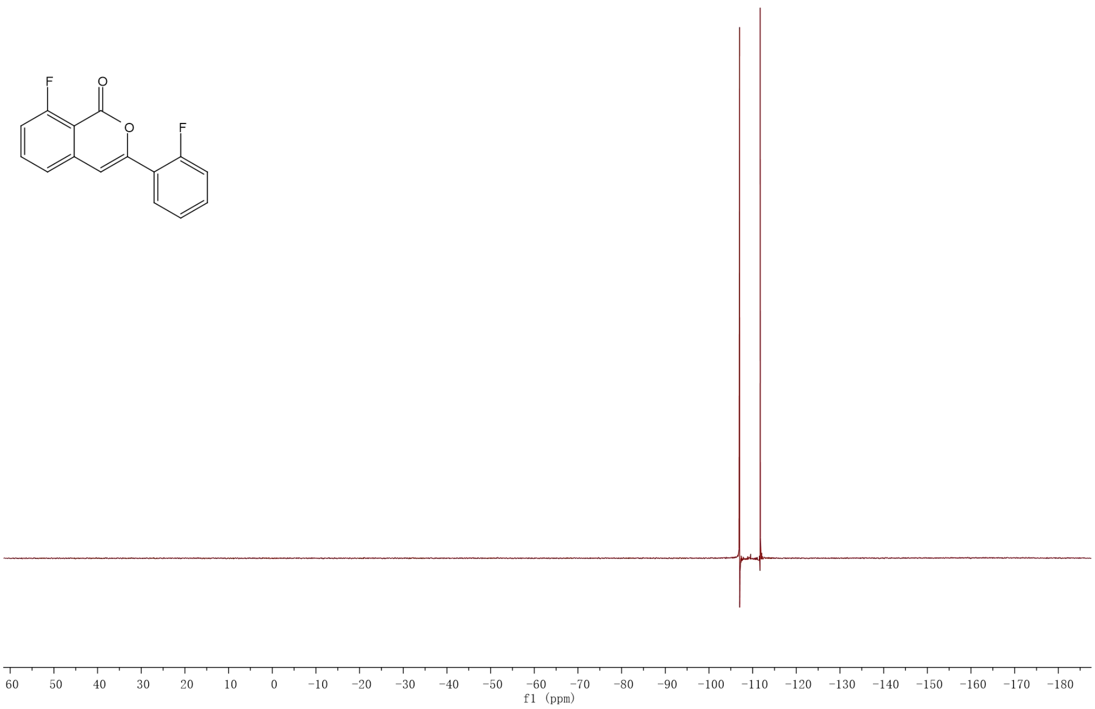

^{19}F NMR (500 Hz, $CDCl_3$): **2t**

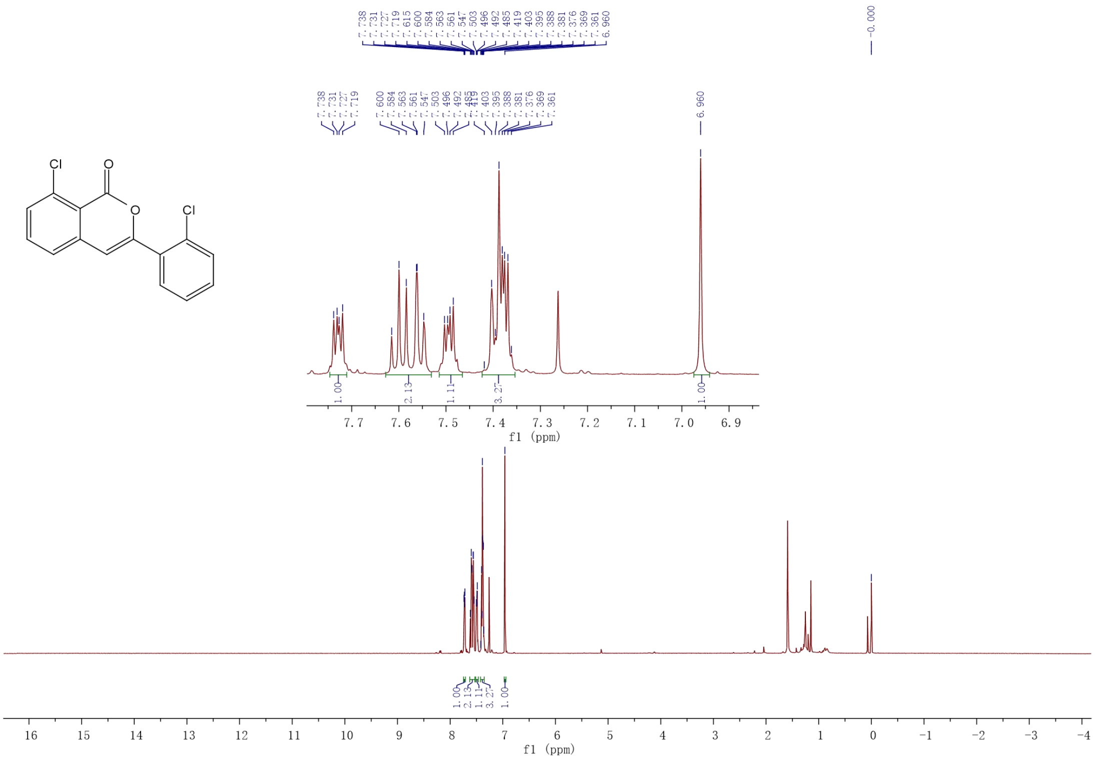

^{1}H NMR (500 Hz, $CDCl_3$): **2u**

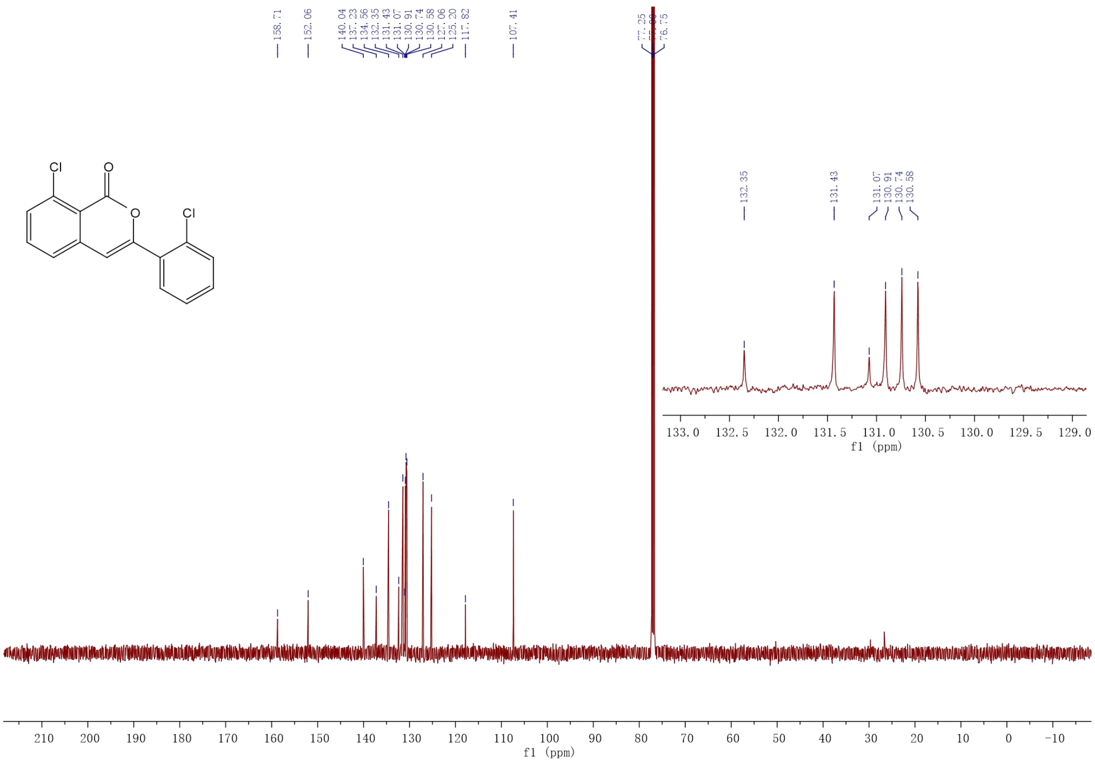

^{13}C NMR (500 Hz, $CDCl_3$): **2u**

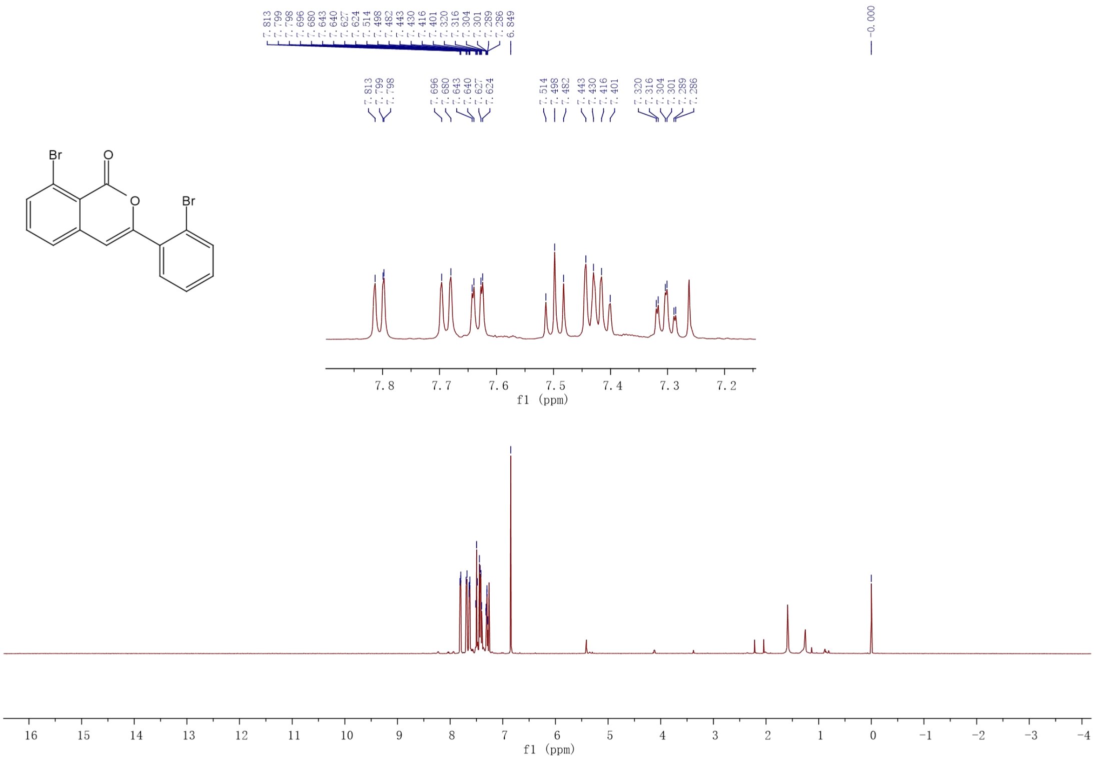

^{1}H NMR (500 Hz, $CDCl_3$):

2v

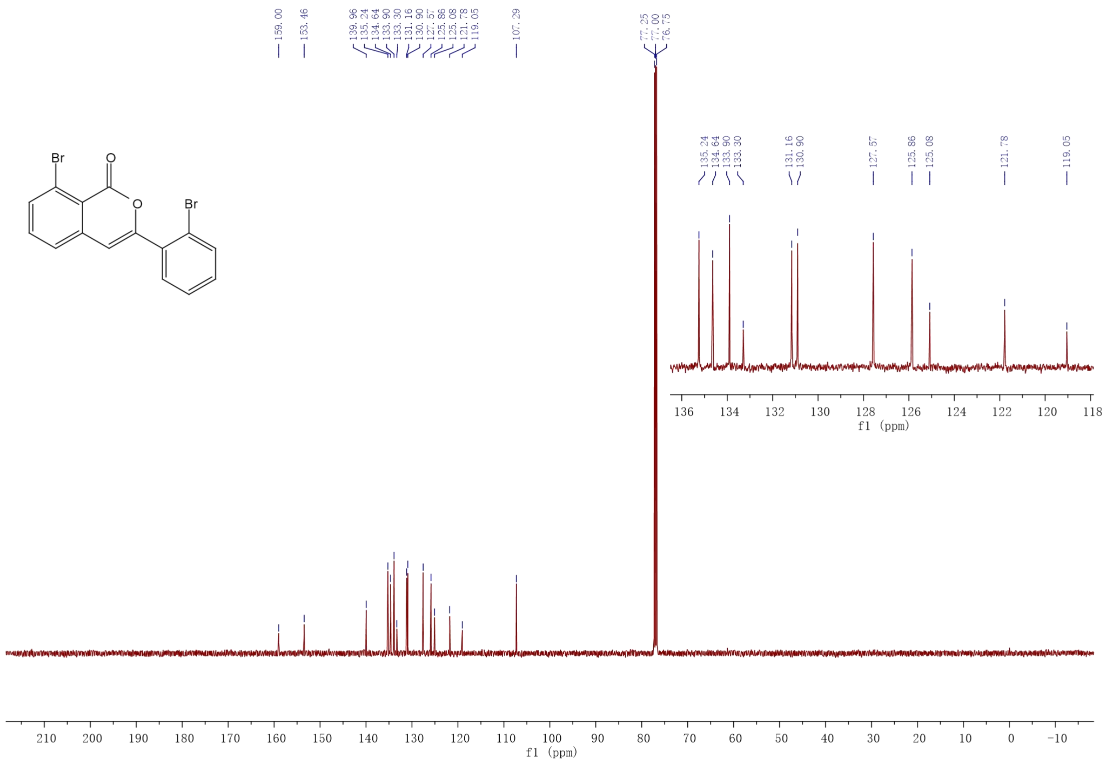

^{13}C NMR (500 Hz, $CDCl_3$): **2v**

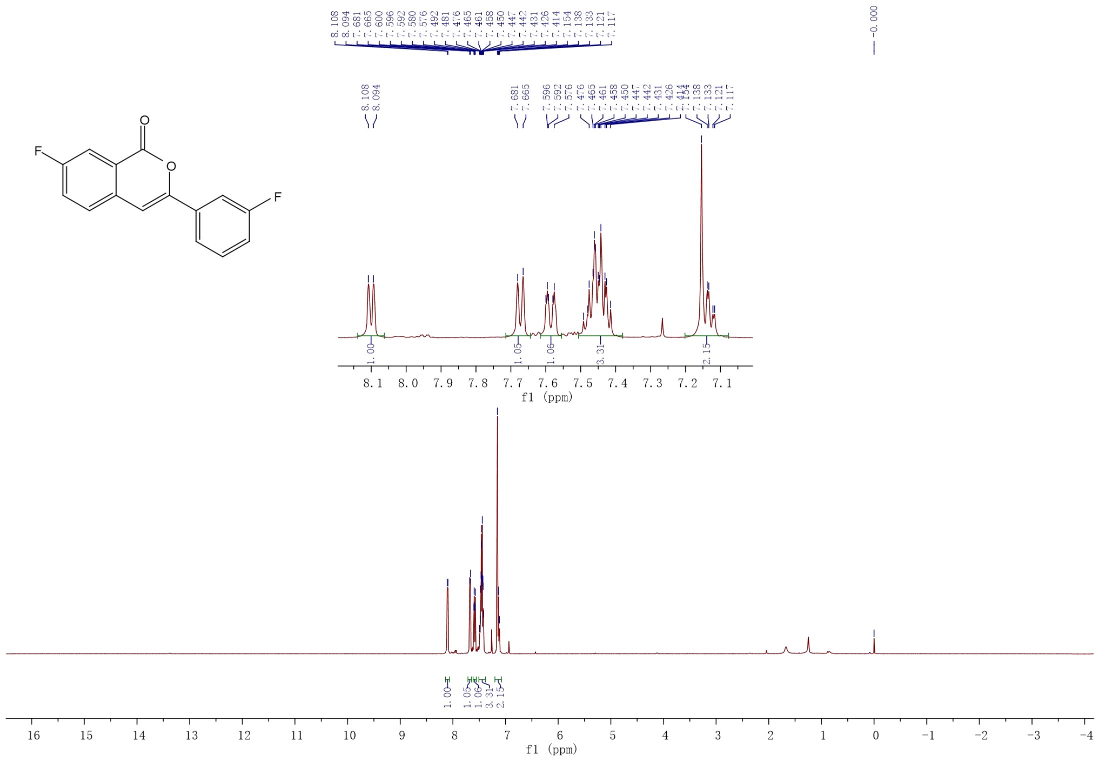

^{1}H NMR (500 Hz, $CDCl_3$): **2w**

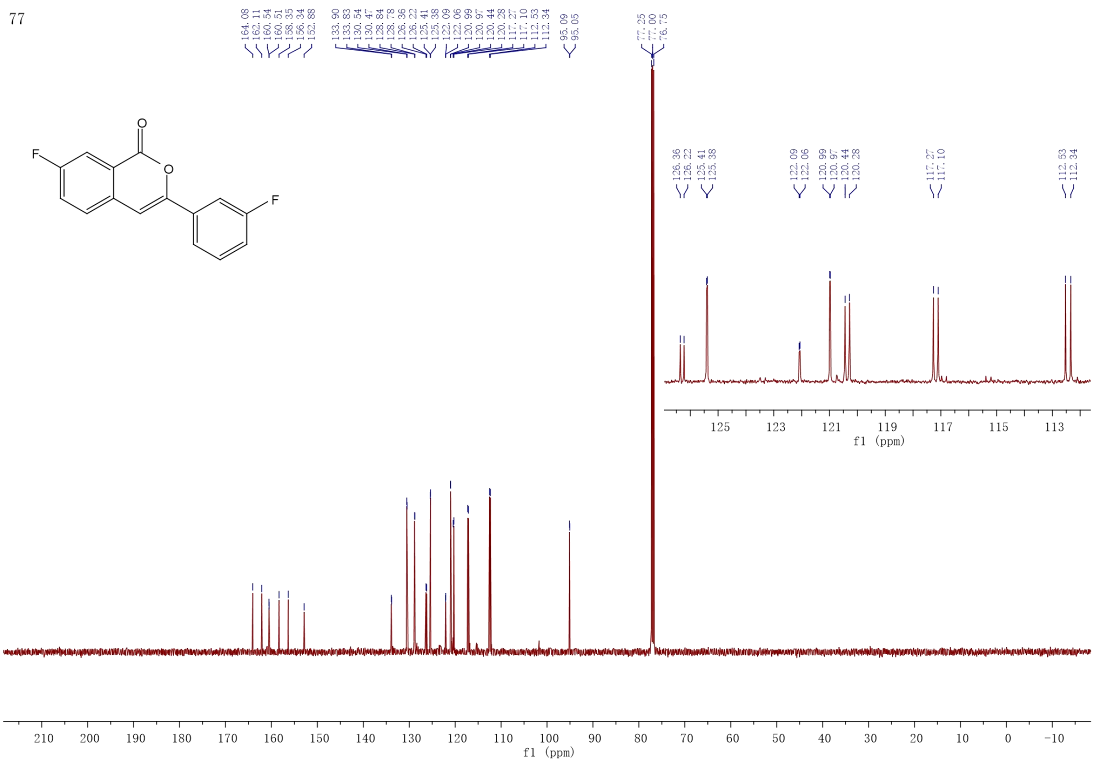

^{13}C NMR (500 Hz, $CDCl_3$): **2w**

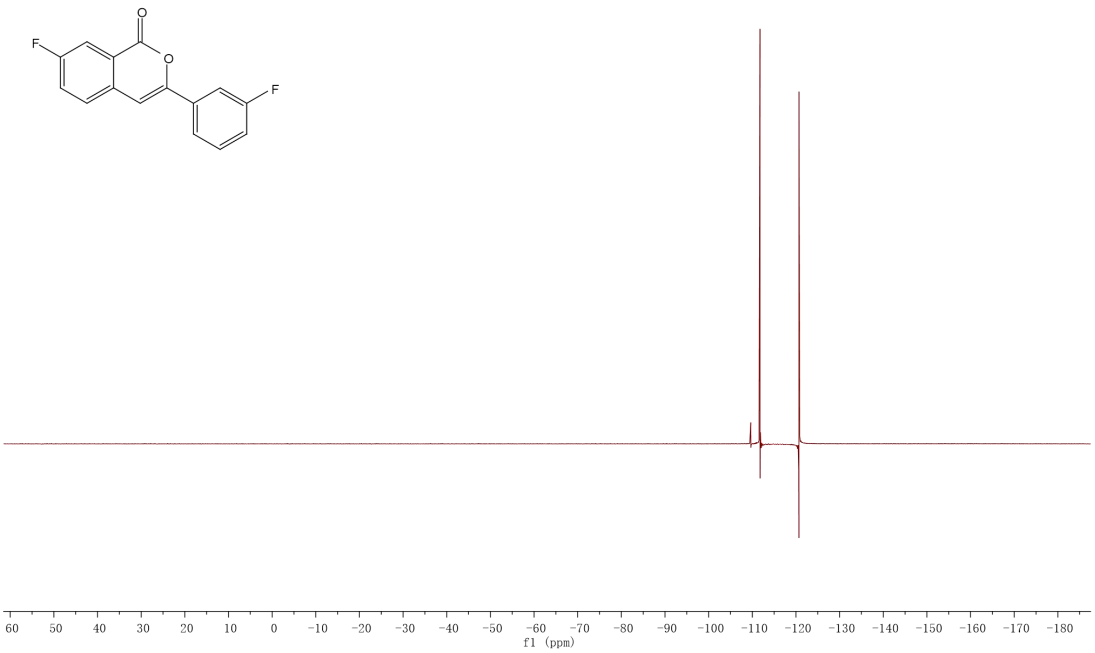

^{19}F NMR (500 Hz, $CDCl_3$): **2w**

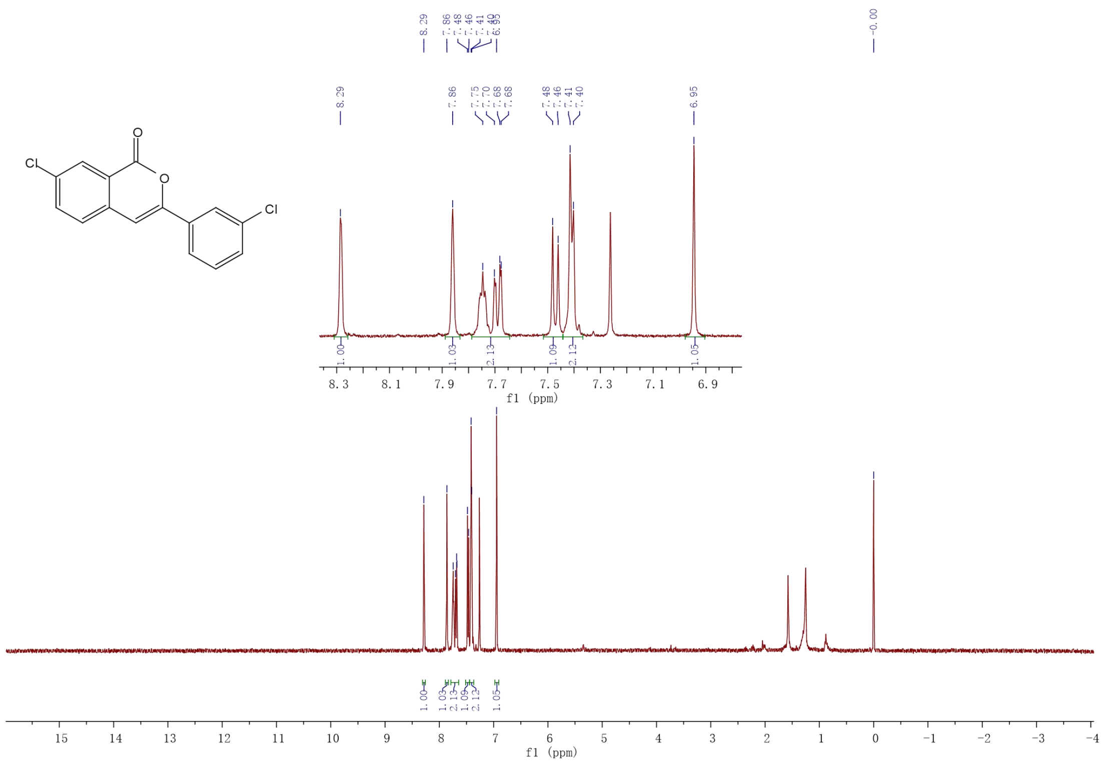

^{1}H NMR (400 Hz, $CDCl_3$): **2x-*p***

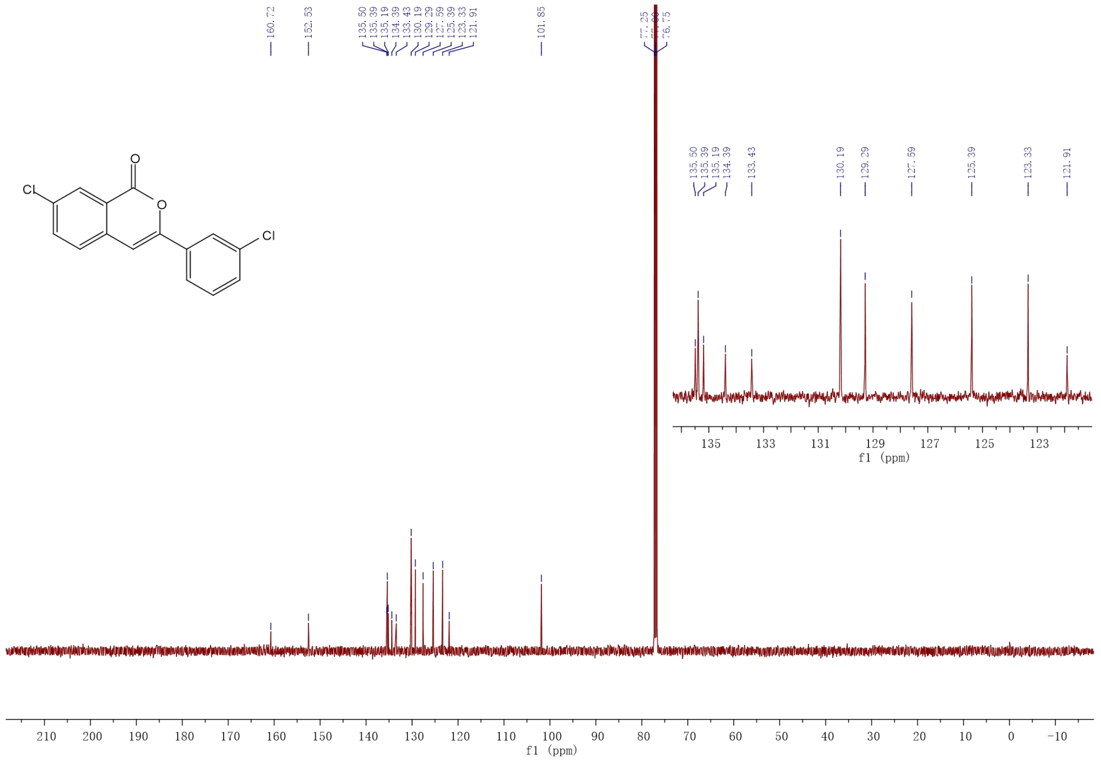

^{13}C NMR (500 Hz, $CDCl_3$): **2x-*p***

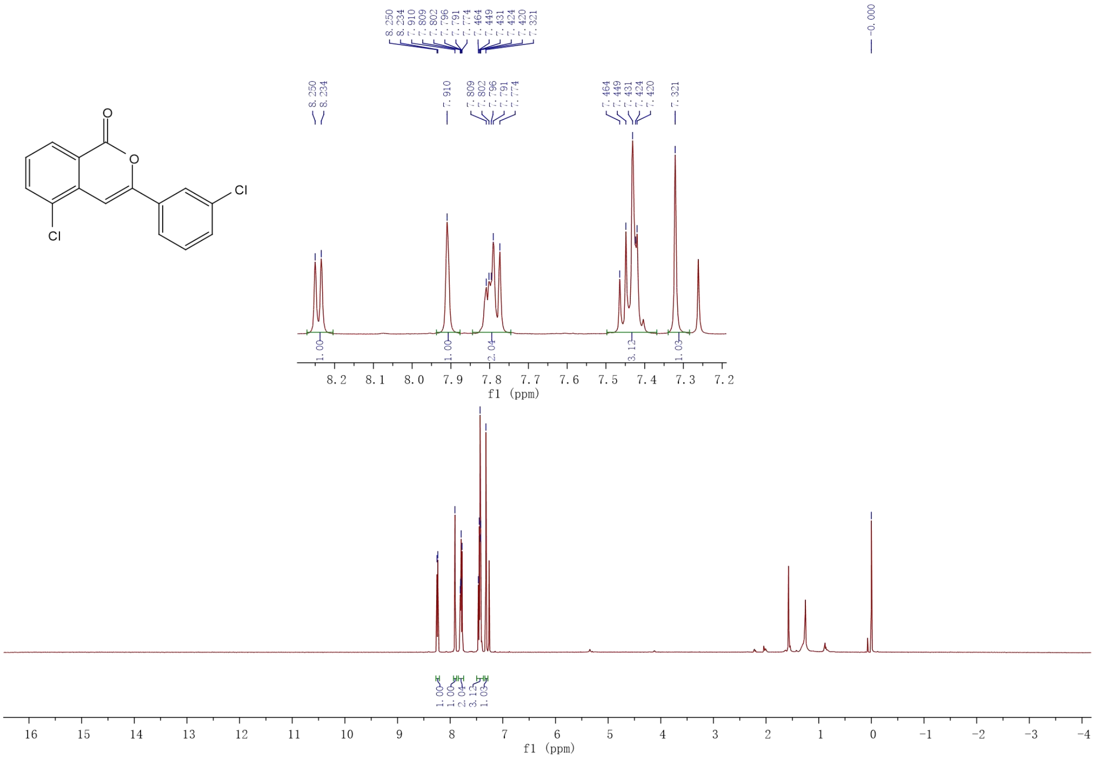

^{1}H NMR (500 Hz, $CDCl_3$): **2x-*o***

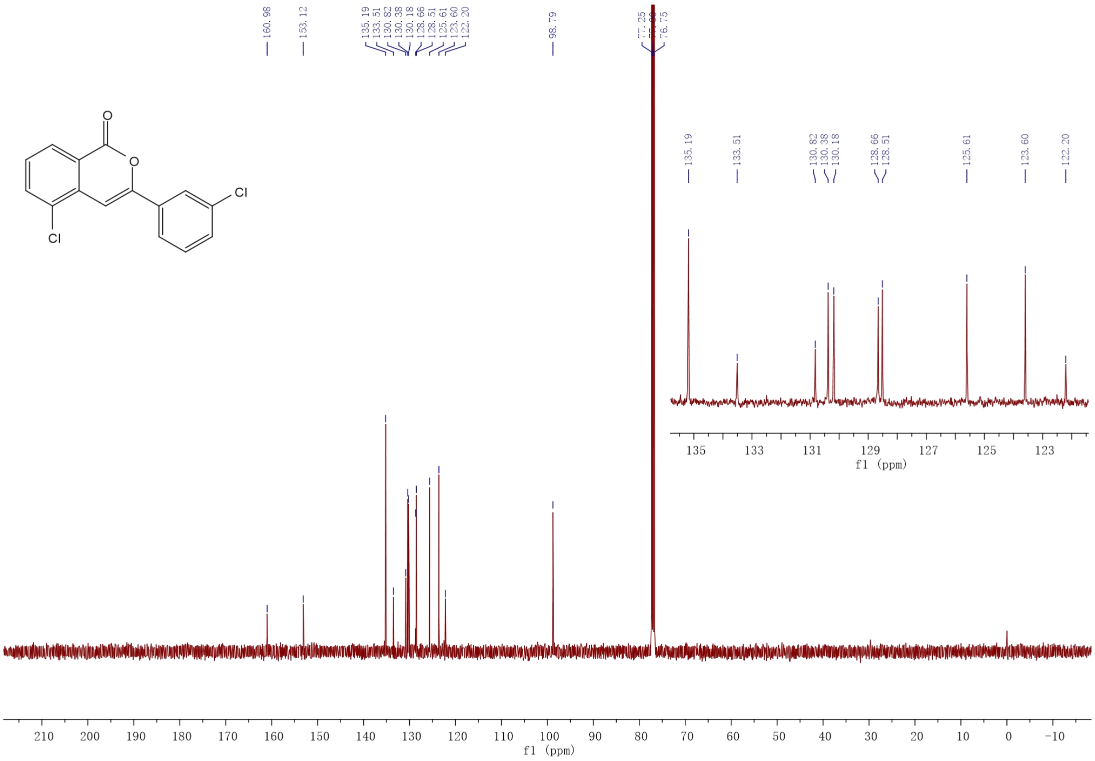

^{13}C NMR (500 Hz, $CDCl_3$): **2x-*o***

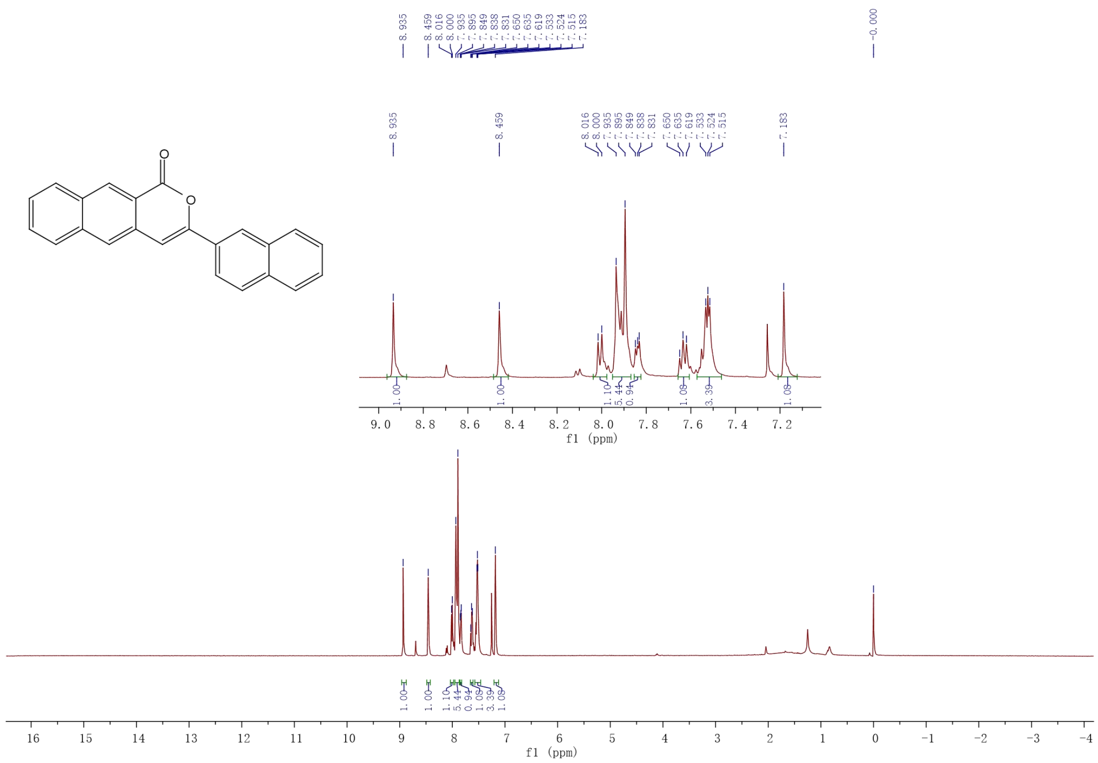

^{1}H NMR (500 Hz, $CDCl_3$): **2y**

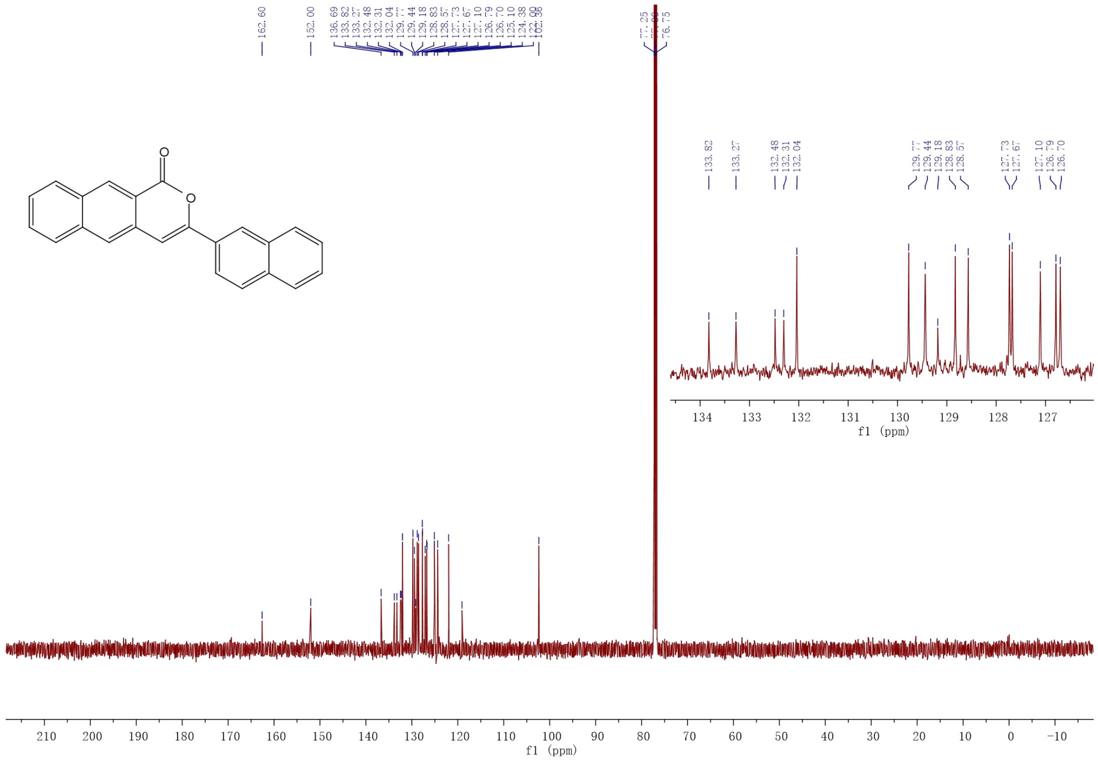

^{13}C NMR (500 Hz, $CDCl_3$): **2y**
